# Genome sequence and analysis of a broad-host range lytic bacteriophage that infects the *Bacillus cereus* group

**DOI:** 10.1186/1743-422X-10-48

**Published:** 2013-02-07

**Authors:** Tarek F El-Arabi, Mansel W Griffiths, Yi-Min She, Andre Villegas, Erika J Lingohr, Andrew M Kropinski

**Affiliations:** 1 Department of Agricultural Microbiology, Faculty of Agriculture, Ain Shams University, Cairo, Egypt; 2 Canadian Research Institute for Food Safety, University of Guelph, Guelph, ON, N1G 2W1, Canada; 3 Department of Food Science, University of Guelph, Guelph, ON, N1G 2W1, Canada; 4 Centre for Vaccine Evaluation, Biologics and Genetic Therapies Directorate, Health Canada, Ottawa, ON, K1A 0K9, Canada; 5 Public Health Agency of Canada, Laboratory for Foodborne Zoonoses, Guelph, ON, N1G 3W4, Canada; 6 Department of Molecular and Cellular Biology, University of Guelph, Guelph, ON, N1G 2W1, Canada

**Keywords:** *Bacillus cereus*, Bacteriophage, *Myoviridae*, Host range, Genome, Proteome, tRNAs

## Abstract

**Background:**

Comparatively little information is available on members of the *Myoviridae* infecting low G+C content, Gram-positive host bacteria of the family Firmicutes. While numerous *Bacillus* phages have been isolated up till now only very few *Bacillus cereus* phages have been characterized in detail.

**Results:**

Here we present data on the large, virulent, broad-host-range *B. cereus* phage vB_BceM_Bc431v3 (Bc431v3). Bc431v3 features a 158,618 bp dsDNA genome, encompassing 239 putative open reading frames (ORFs) and, 20 tRNA genes encoding 17 different amino acids. Since pulsed-field gel electrophoresis indicated that the genome of this phage has a mass of 155-158 kb Bc431v3 DNA appears not to contain long terminal repeats that are found in the genome of *Bacillus* phage SPO1.

**Conclusions:**

Bc431v3 displays significant sequence similarity, at the protein level, to *B. cereus* phage BCP78, *Listeria* phage A511 and *Enterococcus* phage ØEF24C and other morphologically related phages infecting Firmicutes such as *Staphylococcus* phage K and *Lactobacillus* phage LP65. Based on these data we suggest that Bc431v3 should be included as a member of the *Spounavirinae;* however, because of all the diverse taxonomical information has been addressed recently, it is difficult to determine the genus. The Bc431v3 phage contains some highly unusual genes such as gp143 encoding putative tRNA^His^ guanylyltransferase. In addition, it carries some genes that appear to be related to the host sporulation regulators. These are: gp098, which encodes a putative segregation protein related to FstK/SpoIIIE DNA transporters; gp105, a putative segregation protein; gp108, RNA polymerase sigma factor F/B; and, gp109 encoding RNA polymerase sigma factor G.

## Background

The genus *Bacillus* can be subdivided into two groups: the *Bacillus subtilis* group and the *Bacillus cereus* group [1]. Members of the latter subdivision include *B. anthracis*, the causative agent of the fatal human and animal disease, anthrax [2], *B. cereus*, *B. weihenstephanensis, B. thuringiensis*, *B. mycoides* and *B. pseudomycoides. B. cereus* and *B. weihenstephanensis* are implicated in foodborne illnesses as well as food spoilages. Their psychrotrophic properties enable them to cause problems in foods stored at low temperatures especially dairy products [3-6]. *B. thuringiensis*, *B. mycoides* and *B. pseudomycoides* have also been noted to cause food spoilage [7]. In addition, there is some evidence to indicate they potentially can cause foodborne illnesses [1,8-11].

With the exception of phages AP50, AP50-04, AP50-11, AP50-23, AP50-26, AP50-27, and Bam35 which belong to the family of Tectiviridae [12,13], all other *Bacillus* phages belong to the three tailed phage families: Myoviridae, Siphoviridae and Podoviridae. They range from very large viruses such as the G, to very small phages such as ϕ29 and ϕ15. All of the *Bacillus* phages possess dsDNA, and some of them are quite distinct as they contain unusual bases in their DNA. In the case of phage SPO1, thymine residues are replaced with 5-(hydroxymethyl) uracil (HmUra); while in phage PBS2 DNA the thymine are totally replaced by uracil [14]. Among the myoviruses, the subfamily Spounavirinae (SPO plus “una”, Latin for “one”) is comprised of two genera, “SPO1-like viruses” and “Twort-like viruses”. Members of Spounavirinae feature isometric heads 87–94 nm in diameter and tails 140–219 nm long [15-17]. The dsDNA molecules of this subfamily are quite large (127–142 kb) and nonpermuted with 3.1 – 20 kb terminal redundancies. The genus “Spo1likevirus” contains at least ten *Bacillus* phages, Lactobacillus phage 222a as well as *Enterococcus faecalis* phage ØEF24C with only the genomes of SPO1 and ØEF24C phages having been sequenced [16,18-21]. The distinct criterion of their genomes (i.e., “Spo1likevirus”) is the substitution of thymine with HmUra, and the presence dUMP hydroxymethylase activity [16]. The “Twortlikevirus”, on the other hand, contains a group of virulent phages of *Staphylococcus* including Twort, G1 and K phages as well as *Listeria* phages A511 and P100 [22-25]. As far as we know the DNA of these phages is not modified.

It is worthwhile mentioning that because of the close relatedness between members of *B. cereus* group; most of the phages isolated against *B. anthracis* were also able to infect *B. cereus* and vice-versa. The first phage reported against *B. anthracis* was phage W, which was originally induced from a soil strain of *B. cereus*[26]. A lytic variant of W, phage γ, was isolated shortly after and it is now used as a standard protocol for *B. anthracis* identification by the Centers for Disease Control and Prevention and other public health laboratories in the United States [27,28].

Very few phages of *B. cereus* have been isolated and characterized in detail. Some temperate phages were characterized and utilized in the transductional analyses of *B. cereus* strains [25,29-33]. In addition, a number of lytic phages have been isolated and used in phage typing schemes for epidemiological studies [34]. It is noteworthy that phage typing could be considered as an efficient tool especially because of the close relatedness between *B. cereus*, *B. thuringiensis* and *B. anthracis*, providing a cheap, convenient and fairly accurate tool to identify such closely related species. Lytic phages have also been used in the control of *B. cereus* in mashed potatoes [25,35,36].

In the present study, we isolated a lytic phage, vB_BceM_Bc431v3, that showed a strong potential to control *B. cereus*. Prior to its application in food preservation, we needed to characterize it and confirm that it will not affect the pathogenicity of this bacterium. Therefore, the objectives of this study were to determine the genomic sequence of phage Bc431v3, annotate its genes and appraise its structural functions in conjunction with existing phages present in the scientific literature and in the GenBank.

## Results

### Isolation and morphology of Bc431v3

**Figure 1:**
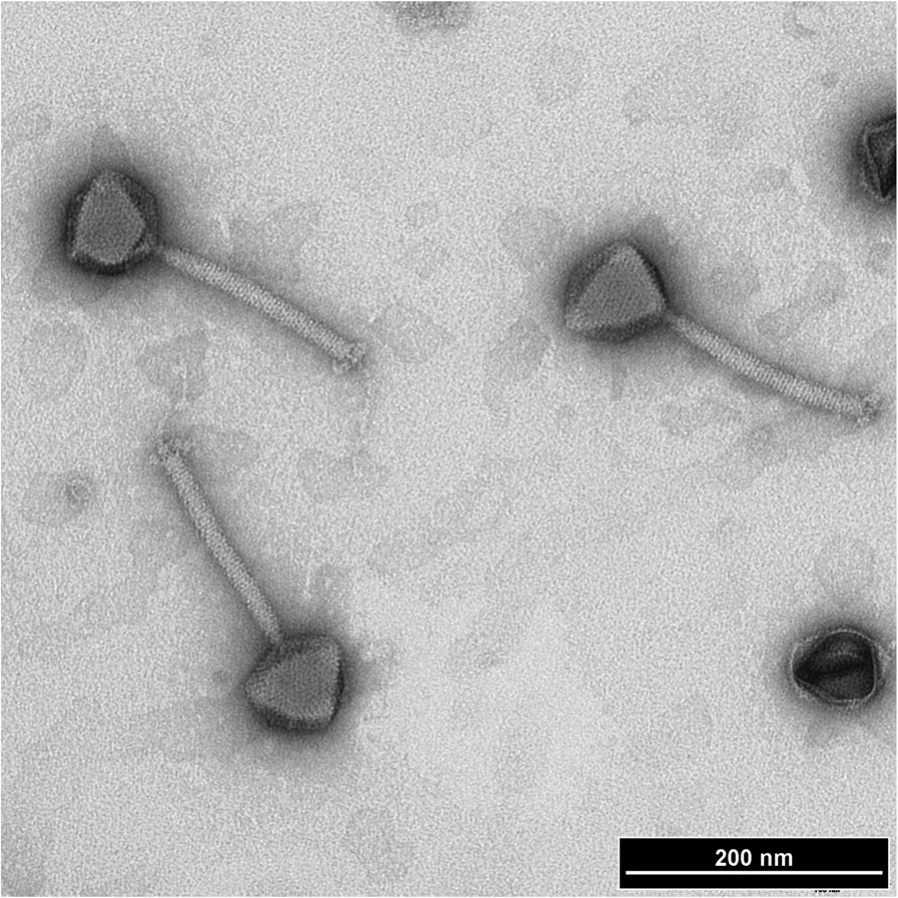
TEM images show the morphological features of the phage Bc431v3.

Phage Bc431v3 was isolated from sewage and produced small (1.8 mm) clear plaques with turbid borders on *B. cereus* strain LJH431. This virus was negatively stained with 2% uranyl acetate and examined by transmission electron microscopy. The micrographs show that this phage has isometric heads 85.4±3 nm in diameter with individual capsomers visible. The virus possesses a long contractile tail 180±3 nm in length by 12±4 nm in width. The base plate has a cluster of projections and what appears to be a central tail fibre. Collectively these features indicate that this virus belongs to the family *Myoviridae* (Figure 1).

### Host range

**Figure 2:**
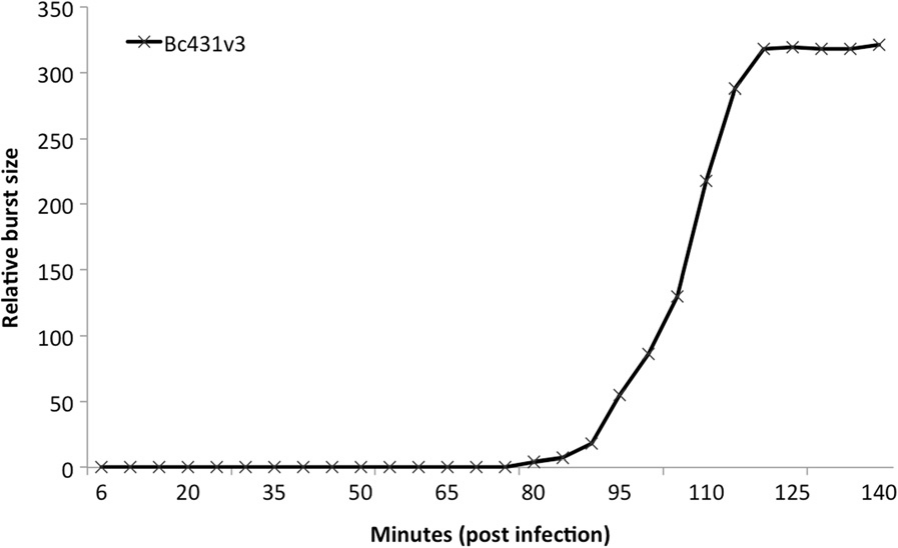
One-step growth curve of phage Bc431v3.

In a host range study, Bc431v3 was able to infect all tested *B. cereus, B. anthracis, B. licheniformis* and *B. weihenstephanensis* strains with different degrees of lysis (Table 1; Figure 2). Phage Bc431v3 also infected *B. thuringiensis, B. psychrosaccharolyticus* and *B. megaterium*, but was not able to lyse a strain of *B. subtilis*.

**Table 1:** Host range pattern of the isolated phages using Bioscreen C

**Host & strains**	Phage lytic pattern*
*Bacillus cereus*	
LJH431	++
LJH432	++
C1009	++
LJH183	++
LJH181	+
LJH180	++
A2	++
D2	++
NizoB434	++
NizoB435	++
NizoB436	++
1230-88	++
75-95	+
391-98	++
PAL-5	++
PAL-26	+
17	+
72	++
67-448	++
PAL-25	+
F4628/90	++
F450/90	++
PAL-18	++
*Bacillus anthracis*	
Δ Sterne	++
*Bacillus weihenstephanensis*	
WSBC 10204	++
WSBC 10207	++
WSBC 10295	++
*Bacillus thuringiensis*	
ATCC 10792	++
*Bacillus subtilis*	
C1004	-
ATCC 6051	-
*Bacillus licheniformis*	
C862	+
Bacillus psychrosaccharolyticus	
ATCC 23296	++
*Bacillus megaterium*	
ATCC 14581	++

* Lysis pattern was measured qualitatively as (++) for full lysis, (+) for partial lysis and (−) for no lysis.

### One-step growth curve

**Figure 3:**
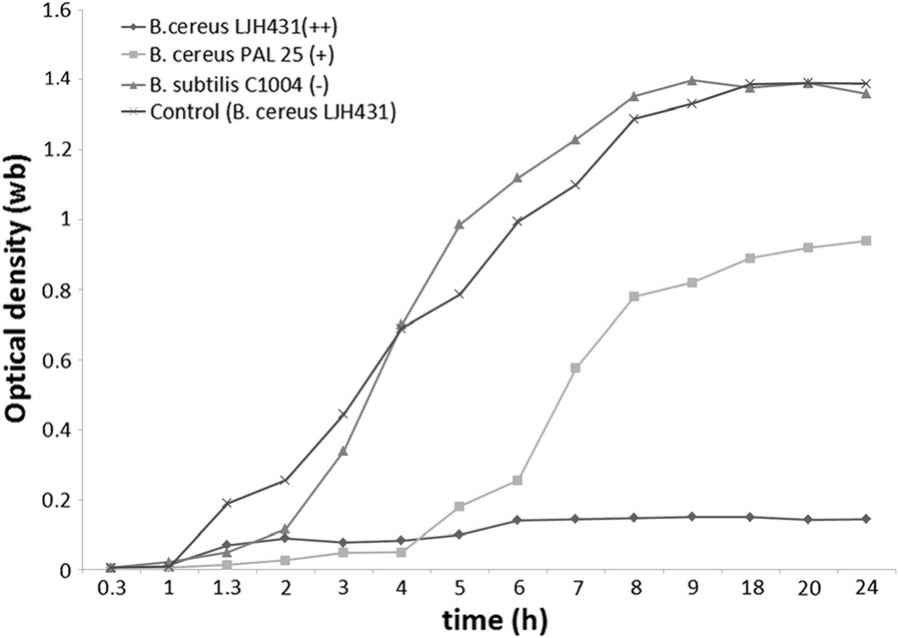
**Representative results from Bioscreen C after adding phage Bc431v3 to different strains of *Bacillus *spp.** This phage was able to completely inhibit the growth of *B. cereus* LJH431 (Full lysis; ++) while it only delayed the growth of *B. cereus* PAL 25 (Partial lysis; +). On the other hand, Bc431v3 could neither inhibit nor delay the growth of *B. subtilis* C1004. Control *B. cereus* LJH431 refers to the growth of *B. cereus* LJH431 in the absence of phage Bc431v3.

The growth characteristics of this virus in *B. cereus* strain LJH431 revealed a latent period of 85 ± 5 min and a burst size of 318 ± 5 (Figure 3).

### Genome of phage Bc431v3

The phage genome was sequenced to 19-fold coverage using 454 (pyrosequencing) technology. The genome is 158,618 bp long and possesses a G+C content of 39%. BLASTX analysis against the nonredundant NCBI database failed to reveal any frameshifts. Since pulsed-field gel electrophoresis indicated that the phage genome is a single linear DNA molecule of 155–158 kb it would suggest that this phage does not possess long terminally redundant ends such as are present on *Bacillus* phage SPO1.

### Identification and analysis of open reading frames (ORFs)

**Figure 4:**
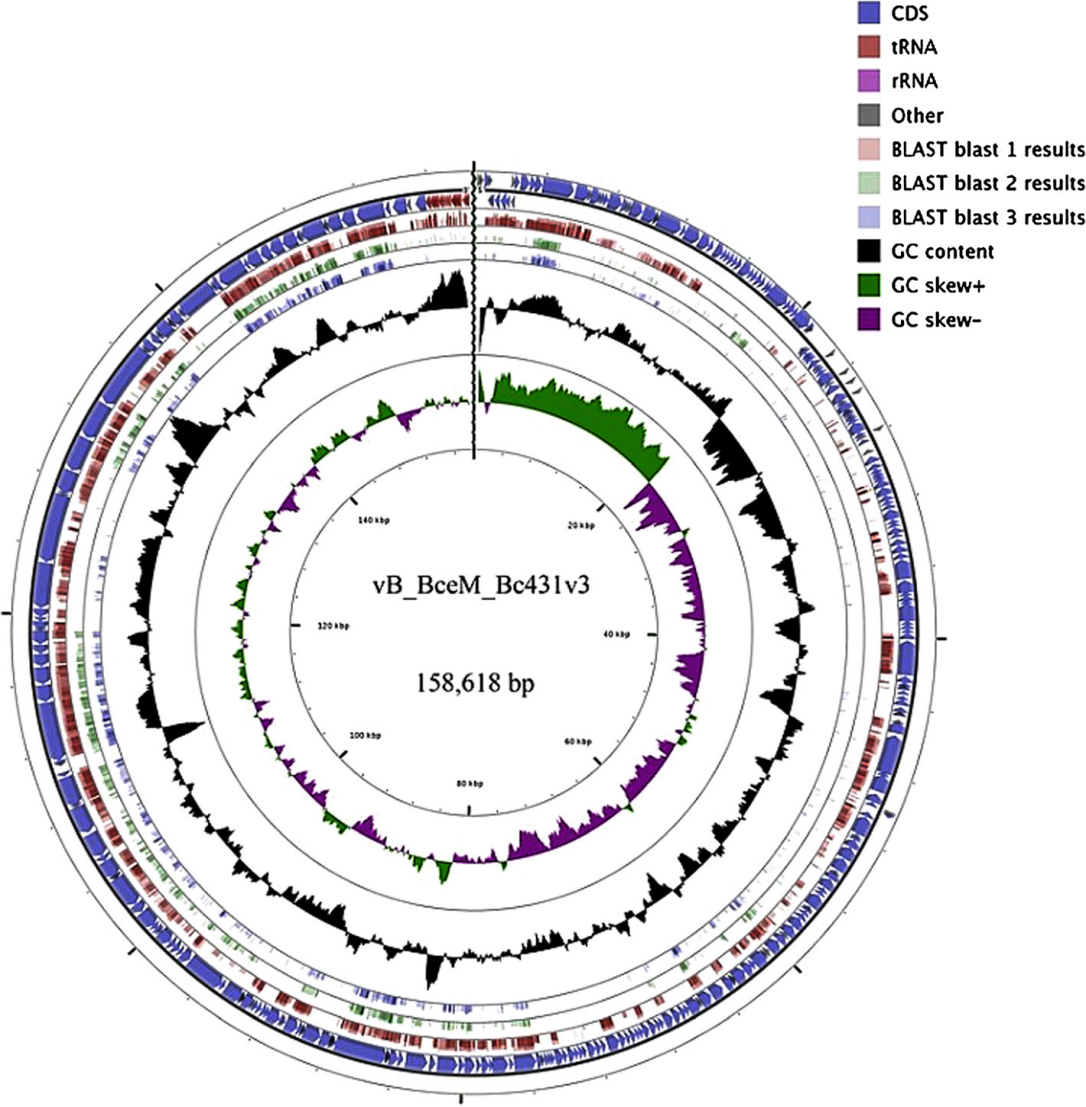
**Genetic and physical map of phage Bc431v3 prepared using CGview (Stothard and Wishart 2005).** Blast 1 shows the sequence homology between phage Bc431v3 proteins and those of *B. cereus* phage Bcp78. Blast 2 shows the homology between phage Bc341v3 and *Listeria* phage A511. Blast 3 shows the sequence homology between phage Bc431v3 and *Enterococcus* phage ØEF24C. In each of these cases TBLASTX was used to search for sequence similarity at the protein level.

The genome was initially analyzed using AutoFACT, with the ORF calls verified using Kodon and BLAST. A total of 239 putative ORFs were identified in the genome (Figure 4; Additional file 1: Table S1). A total of 143,817 bp nucleotides (90.7% of the genome) are involved in coding for putative proteins. Of these, 38 ORFs (15.9%) had identified functions, while 76 ORFs (31.8%) were identified as possessing homologs with proteins in the nonredundant NCBI database; however, their functions are undefined (conserved hypothetical proteins). A large percentage (125 ORFs; 52.3%) were considered to be hypothetical proteins unique to this phage. Using the homology approach introduced by Abbasifar *et. al.,*[37] no toxins were identified among the proteins specified by this phage. Three different start codons utilized in the genome; ATG, GTG and TTG were used at frequencies 88.7%, 5.9% and 5.5%, respectively.

The genome of this phage was exposed to DraI and SspI, EcoRI and NdeI which resulted in poor digestion, while HindIII and AccI failed to digest (data not shown). *In silico* restriction endonucleases digestion of phage Bc431v3 DNA revealed that the DNA possessed 200 AccI sites, 28 NdeI sites and 23 HindIII sites, suggesting that the DNA of this virus may be modified. Two C-5 cytosine-specific DNA methyltransferases were identified in the genome encoded by genes 172 and *173*, but their impact on the digestibility of the DNA is unknown.

In the following sections the properties of some of the genes will be discussed.

### Nucleotide metabolism and DNA replication

Sequence-based predictions identified many genes as being involved in Bc431v3 nucleotide metabolism and DNA synthesis. The former included thymidylate synthase (gp021), ribonucleotide reductase alpha (gp185) and beta subunits (gp183), dihydrofolate reductase (gp024), deoxyuridine 5^′^-triphosphate nucleotidohydrolase (gp190), an exonuclease I homolog (gp194), and a putative exonuclease II (gp195). The proteins involved in DNA replication included a DNA polymerase (gp170), DNA primase (gp192), and two DNA helicases (gp197 and gp200).

### DNA packaging and morphogenesis – genomic and proteomic analysis

Several genes encoding proteins directly involved in DNA packaging and morphogenesis were identified in Bc431v3 genome. Terminase small and large subunits homologs were determined to be the products of genes *010* and *011*, respectively. Genes encoding capsid morphogenesis were determined to be *232, 235* and *236* for major head, prohead protease, and portal vertex proteins, respectively. Tail proteins were expressed by a set of genes, which are *224* (tail core protein), *225* (tail sheath protein), *203* and *215* (putative tail proteins), *205* and *206* (baseplate proteins); and, *204* for putative tail fiber adapter protein. It is highly unusual for the terminase genes to be separated from the capsid-tail gene complex.

A cluster of genes starting from *orf203* to *orf230* carried the genes that are involved in morphogenesis, and their products were structurally similar to *B. cereus* phage Bcp78 and *Listeria* phage A511 [23,25]. Additionally, it was found that the product of *orf218* is a putative tail protein possessing endo-beta-N-acetylglucosaminidase activity that also contains three transmembrane domains. BLASTP homology showed that the gp218 has the motif of C-terminus similar to the putative tail lysin of the *Enterococcus* phage ØEF24C [19] as well as the pfam lysozyme sub-family 2. Additionally, gp218 also showed a homology with *Listeria* phage A511 gp97, a putative peptidase [23].

### Proteomic analysis

To achieve reliable protein identification, the purified phage were digested by two different enzymes (trypsin and chymotrypsin) and analyzed by high resolution UPLC and LTQ-FT mass spectrometry in duplicates. The combined dataset from Mascot database search identified 45 phage proteins, in which the top 22 proteins were present at high scores and sequence coverages (Additional file 2: Table S2). As expected, this short list included the highly abundant structural proteins of phage Bc431v3: major capsid membrane protein (orf232; sequence coverage 50%), tail protein (orf203; 13%), tail sheath protein (orf225; 63%), tail fiber protein (orf215; 14%), tail protein possessing endo-beta-N-acetylglucosaminidase activity (orf218; 35%), baseplate protein (orf205; 23%), and related structural protein (orf166; 39%). Other phage interacting proteins and enzymes were also detected such as N-acetylmuramoyl-L alanine amidase (orf012; coverage 87%) and prohead protease (orf235, 12%). Prohead protease is a proteolytic enzyme essential for phage capsid morphogenesis [38], and N-acetylmuramoyl-L alanine amidase plays an important role in DNA synthesis through cleavage of amide bonds between N-acetylmuramoyl and L–amino acids in the bacteriophage cell walls.

### Transcription

We know relatively little about the regulation of transcription in large *Bacillus* phages. Using a combination of two techniques, visual scanning for sequences similar to SigA promoters (Sigma43; TTGACA(−35)-N_14_-tgnTATAAT(−10); [39]) and MEME analysis we identified 27 putative promoters, 12 of which contained the extended −10 region (Additional file 3: Table S3). All of these sequences possessed typical AT-rich UTR sequences, but atypically the −35 region has the consensus TTGTTGAC, not TTGACA as would be expected for promoters recognized by SigA. The majority of putative promoters were located upstream of genes involved in nucleotide metabolism (e.g. tRNA^His^ guanylyltransferase, dihydrofolate reductase) or DNA synthesis (DNA polymerase, primase, polymerase) which, based upon analysis of other phages, correspond to middle or delayed early genes. Because of the lack of homologs we cannot define the early genes of this phage. One of the middle genes (*orf163*) specifies a protein which is related to proteins defined as sigma factors for *Enterococcus* phage phiEF24C (YP_001504175), *Staphylococcus* phage K (YP_024522), *Bacillus* phages SP10 (BAK53012) and SPO1 (YP_002300430). In the latter virus, gp34 is known to facilitate, along with gp33 late transcription. Based upon similarity to CGTTAGA(N_17-19_)GATATT [19] and allowing for 2 bp mismatch we identified four putative late promoters upstream of genes *056*, *162*, *190* and *198*. Examination product of Bc431v3 *orf164* using HHPred [40], failed to demonstrate homology to SPO1 gp33.

Interestingly, the Bc431v3 genome, like that of temperate phage Bcp1 [28], was found to carry two other genes encoding for bacterial-type sigma factors. These are the products of genes 108 and 109, which appear to be members of the RNA polymerase sigma F/B and sigma factor G subfamilies, respectively. MEME analysis using a variety of *Bacillus, Acyclobacillus*, *Geobacillus* and *Paenibacillus* sigma factors revealed that gp108 shared the following motif Wx(9)Ix(2)Lx(3)Ex(2)Ix(6)KDx(2)QSx (2)Ax(2)LGx(5)V with SigmaF-like proteins, while gp109 shared DDxFQxGxIGLx(3)Ix(2)FDx(6)FSTYAV with related SigG proteins in the *Bacillaceae.*

As with *Bacillus* phage SPO1 [19] the genome of Bc431v3 contains a large number of rho-independent transcriptional terminators (Additional file 3: Table S3).

### Lysis genes

The final stage of the phage lytic cycle is degradation of the bacterial cell wall and release of progeny phages. The lysis of the cell wall is typically induced by two phage-encoded proteins, holin and endolysin [41,42]. Holin forms a hole in the cell membrane, and endolysin passes through the hole and destroys the peptidoglycan structure [41,42]. Two gene products were determined to be the possible lysin, an N-acetylmuramoyl-L-alanine amidases encoded by gene 012 and/or L-alanoyl-D-glutamate peptidase encoded by gene 222. The former contains a MurNAc-LAA [cd02696], N-acetylmuramoyl-L-alanine amidase domain while gp222 contains a COG3584 (uncharacterized protein conserved in bacteria) domain. Both share homology to defined phage lysins. As for the holin, while ten proteins possessed two transmembrane domains, and two three domains, none possessed the small size generally associated with holins.

### Codon usage and tRNAs

The Bc431v3 genome was found to contain 20 tRNA genes for 17 amino acids (Additional file 4: Table S4). By comparing the codon usage pattern of the phage with that of its host (*Bacillus cereus*) we were able to see that in ten cases the phage-encoded tRNAs may significantly enhance translation of phage mRNAs (Additional file 5: Table S5). The products of two genes 143 and *177* appear to encode proteins, a tRNA^His^ guanylyltransferase [43] and predicted a nucleotidyltransferase, respectively, which may modify the phage or host tRNAs. As is quite common, this virus also includes a putative tRNAMet, which TFAM identified as a selenocysteinyl tRNA [44].

### Unusual genes

The phage’s Bc431v3 genome was found to contain several genes that have rarely been detected in other phages. In addition to sigma factors which are related to similar host proteins, we identified a putative segregation protein (gp098) related to FtsK-SpoIIIE, a DNA-binding protein (gp174) related to integration host factor (IHF), and a putative segregation protein gp105.

### Taxonomy

**Figure 5:**
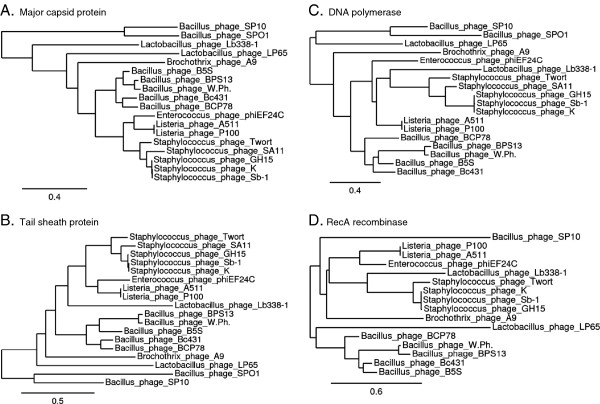
**Phylogenetic analysis of Bc431v3 proteins using “one click” at phylogeny.fr (http://www.phylogeny.fr/version2_cgi/simple_phylogeny.cgi): A. Major capsid protein, B. Tail sheath protein, C. DNA polymerase, and D. RecA recombinase.**

Using BLASTN the sequence of Bc431v3 showed significant sequence similarity to the terminases of *B. cereus* phage Bcp1 [45], and *B. anthracis* phages PlyM19 [46] and 1102^ϕ^3-1 [28]. At the protein level TBLASTX analysis (Figure 4) revealed similarity between Bc431v3 and *Listeria* phage A511 and *Enterococcus* phage ØEF24C localized to regions: 5^′^-end to 20 kb, 60–120 kb, and 130 kb - 3^′^-end. The 20-60 kb regions encode a wide range of hypothetical and conserved hypothetical proteins, plus the FtsK-SpoIIIE, and two sporulation-specific sigma factors, while the 120-130 kb region species a number of minor structural proteins. More detailed analysis using CoreGenes [47,48] showed that this phage shared almost 36.4% sequence homology with *Listeria* phage A511 and *Enterococcus* phage ØEF24C while it only showed 24.3% sequence homology with *Bacillus* phage SPO1. In a recent reclassification of the *Myoviridae*, the subfamily *Spounavirinae* was proposed [16] with two genera the “Spo1likevirus” [23] and the “Twortlikevirus.” Recently a considerable number of new complete genomes of *Bacillus* and *Staphylococcus* genomes [49] have been deposited in GenBank. Rather than verifying the ICTV taxonomy proposal (2009.009a-pB.A.v3) or the paper by Klumpp *et al.*[18] the new data, as shown through CoreGenes proteomic analyses and phylogeny (Figure 5) strongly suggests that the *Spounavirinae* are far more diverse than originally realized. It would, we think be premature to assign Bc431v3 to any genus in the *Spounavirinae*.

## Discussion

In this study we studied the biological, genomic and proteomic characteristics of Bc431v3, a strong lytic phage that is capable infecting a broad range of *Bacillus* species in general and members of *B. cereus* group in particular. This virus lacked repressor, site-specific integrase, virulence and antibiotic resistance determinants, which increase its potential application to the biocontrol of members of the *B. cereus* group.

One of the unique features of this virus is the presence of several rare or unique genes. Gene *174* encodes a putative DNA-binding protein related to bacteria integration host factor (IHF). The latter protein is nucleoid-associated and is implicated in a number of chromosomal functions including DNA compaction [45]. Bc431v3 also carries several genes directly related to host sporulation regulators, such as gene *105* encoding a putative segregation protein related to the FstK/SpoIIIE family of DNA transporters (DNA translocase). DNA transporters are responsible for the translocation of chromosomes after the completion of genome division. In *Bacillus*, the DNA translocase SpoIIIE is involved in transporting miss-segregated DNA during vegetative growth [50,51] and also plays an essential role in chromosome segregation during the process of sporulation [52]. Additionally, genes *108* and *109* encode sigma factor F/B σ^F/B^ and sigma factor σ^G^, respectively. After verification of protein motifs with MEME software, we found that gp*108* displays >70% sequence similarity to *Bacillus* sigma factors F and B while gp109 *is >*80% related to *Bacillus* sigma factor G. These proteins are both forespore transcription factors functioning respectively early and late in sporulation [50]. Occurrence of sporulation-related transcriptional factors on the phage Bc431v3 genome raises intriguing questions about their role in phage transcription. Schuch and Fischetti noted similar sigma factors F and G genes on the temperate phage Bcp1 genome that infects *B. anthracis*[30].

## Materials and methods

### Bacterial strains and culture media

*Bacillus cereus* strain LJH431 used in this study was obtained from the Canadian Research Institute for Food Safety culture collection (CRIFS, University of Guelph, Guelph, ON, Canada). Another fifteen strains of *B. cereus* were provided generously by T. Abee (Laboratory of Food Microbiology, Wageningen University, Netherlands). Strains A2 and D2 of *B. cereus* were kindly provided by A. Hudson (Christchurch Science Centre, Institute of Environmental Science and Research, New Zealand). Strains of *B. weihenstephanensis* were kindly provided by S. Scherer (Department of Biosciences, Wissenschaftszentrum Weihenstephan, Germany). Finally *B. anthracis* ΔSterne was obtained from K. Amoako (Canadian Food Inspection Agency, Ottawa, ON).

*B. cereus* strain LJH431 was grown in half-strength media of Tryptic Soy Broth (TSB; Difco Laboratories, Detroit, MI), half-strength Tryptic Soy Agar (TSA: TSB plus 1.5% agar) and half-strength Tryptic Soy Soft Agar (TSB + 0.5% agar) at 30°C for 24 h.

### Transmission electron microscopy

The morphology of the phage Bc431v3 was examined by transmission electron microscopy as described [53]. Briefly, 1 ml of each phage (~ 9 log_10_ PFU ml^-1^) was centrifuged at 16,000 × g for 1 h at 4°C (Beckman J-20 centrifuge, Beckman Coulter Inc., Mississauga, ON), pellets were washed once using CM buffer (2.5 g/L MgSO_4_.7H_2_O; 0.05 g/L gelatin; 6 ml/L 1 M Tris buffer; 0.735 g/L CaCl_2_; pH 7.5) and then resuspended in 300 μl of CM buffer. Five microliters of the phage suspension were applied onto 300-mesh copper grids coated with formvar and allowed to stand for 1 min. The excess liquid was removed by filter paper and grids were stained with 2% uranyl acetate for 30 s then carefully blotted with filter paper to remove excess stain solution. Negatively stained phages were examined with a LEO 912AB transmission electron microscope (Energy filtered TEM [EFTEM], LEO 912AB model at 100kv, Zeiss, Germany).

### Phage isolation and purification and, extraction of DNA

Phage vB_BceM_Bc431v3 was isolated from sludge samples collected from a local wastewater management plant, and plaque purified. Lysates were prepared using the solid propagation method as described elsewhere [54], clarified from bacterial cell debris by centrifugation (5,400× g, 15 min) and filtered through 0.45 μm mixed cellulose ester (MCE) Fisher brand syringe filters (Fisher Scientific Company, Ottawa, ON, Canada). DNase I and RNase A (Sigma-Aldrich, Oakville, ON, Canada) were added to 10 μg/ml and held at 37°C for 30 min. Subsequent DNA purification steps were carried on according to the handbook of QIAGEN® Lambda midi Kit (Qiagen Inc., Mississauga, ON, Canada). DNA concentration was determined by absorbance at 260 nm using a Nanodrop™ 1000 spectrophotometer (Thermo Scientific, Ottawa, ON, Canada).

### Determination of host specificity using Bioscreen C technology

The host range of the phage Bc431v3 on the selected strains of *Bacillus* spp. was determined using the spot test technique and also by measuring the optical density (OD) in liquid medium of the tested bacterium in the presence of phage using the Bioscreen C Microbiology Plate Reader (Labsystems, Helsinki, Finland) as described elsewhere [53]. The following settings were used: wide band (wb) wavelength; 25°C incubation temperature; 10 min preheating time; kinetic measurement; measurement time 24 h at time intervals of 30 min with medium intensity shaking for 10 s before and after each measurement. Fifty microliters of each phage lysate were transferred to each of the 100 wells of the sterilized honeycomb plates of the Bioscreen C reader (Fischer Scientific), and then each of the wells was inoculated with 125 μl of an overnight bacterial culture at final concentrations of 3 log_10_ CFU ml^-1^. The multiplicity of infection (M.O.I) used to determine the host range of the phage was around 10^3^. In this experimental design, three types of control samples were used: phage only in broth, bacteria only and sterile medium. The absorbance data were analyzed using the Bioscreen C data processing software version 5.26 (Labsystems) to determine the detection time (time required for each test well to increase by 0.3 OD units). Detection times (h:min) were converted to decimal values, averaged and the mean control detection time was subtracted from all test data for each isolate tested and expressed as detection time difference (DT diff.). Based on the host specificity, phages were divided into three groups: (++), when phages completely inhibited the growth of the host bacterium; (+), indicating that phages delayed the growth of the host bacterium; and (−), indicating that the phage had no effect on bacterial growth.

### One-step growth curve

Burst sizes and latent periods of the selected phages were determined by a one-step growth experiment as described by Anany [53]. Phages were added to its host bacterium at an MOI of around 0.1 and incubated in a water bath at 30°C for 5 min. One ml was removed and added to 100 μl of chloroform and mixed well. One hundred microliters of this mixture were added to 100 μl of an overnight culture of the host bacterium and mixed with 4 ml of overlay media and poured onto TSA agar plates to determine the degree of adsorption of the phage to bacterial cells. After an additional 30 s at 30°C, 100 μl were transferred to a tube containing 9.9 ml of fresh TSB and then diluted 10 times in fresh TSB (0.1 log_10_ ml^-1^). One ml of the 10^-1^ dilution tube was additionally diluted 10 times in fresh TSB (0.01 log_10_ ml^-1^) and all three tubes (the original plus 10^-1^ and 10^-2^ dilutions) were incubated in a water bath at 30°C. After 6 min, samples were collected every 5 min for 3 h and phages were titrated in each respective sample as previously described. The relative burst size was determined according to the equation:

Relative burst size = [(Final titre – Initial titre)/ Initial titre]

The relative burst size at different times was plotted against time to determine the latent period.

### Determination of phage genome size using PFGE

Phage genome size was determined by pulsed-field gel electrophoresis (PFGE) as described elsewhere [55]. Phage particles were embedded in 1% Seakem Gold agarose (Mandel Scientific, Guelph, ON) and subjected to electrophoresis in 0.5X TBE buffer (5X: 20 ml of 0.5 M EDTA [pH 8.0], 53 g/l Tris base, and 27.5 g/l boric acid) at 14°C for 18 h, using a CHEF DR-III Mapper electrophoresis system (Bio-Rad, Mississauga, ON) with pulse times of 2.2-54.2 s, at 6 V/cm. Low range DNA marker and phage lambda DNA concatemers (New England Biolabs) were used as size standards. The gels stained with ethidium bromide and DNA bands were visualized under UV transillumination. PFGE results were analyzed using BioNumerics software (Applied Maths Inc., Austin, TX).

### Genome sequencing and annotation

The sequencing of phage Bc431v3 DNA was carried out at the McGill University Genome Quebec Innovation Centre (Montreal, QC, Canada) using pyrosequencing (454 technology). AutoFACT automated annotation software [56] was initially used for genome annotation and then all open reading frames (ORFs) were confirmed using Kodon version 2.0 (Applied Maths). The individual proteins were analyzed using BLASTP against the protein databases at NCBI (http://www.ncbi.nlm.nih.gov). Protein motifs structures were identified using Pfam 24 (http://pfam.sanger.ac.uk/) [57]. In the case of the phage sigma factors a motif analysis was carried out using MEME v.4.5.0 [8]. Phage-encoded tRNA genes were identified with Aragorn [58] and tRNAscan-SE v.1.21 [59] using the default parameters. DNAMAN (Lynnon Corp., Vaudreuil-Dorion, QC, Canada) was used to determine the codon usage information of both Bc431v3 and its bacterial host *Bacillus cereus*, which also provided information on the GC content and presence of direct repeats in Bc431v3 genome. Putative promoters were identified by visual inspection for sequence similarity to TTGACA-N_15-18_-TATAAT, and by MEME analysis on the 5^′^ sequences extracted using extractUpStreamDNA (http://lfz.corefacility.ca/extractUpStreamDNA/). Rho-independent terminators were determined by ARNold and TransTerm software [60,61] and verified by examining the secondary structure of the DNA adjacent to polyT sequences using Mfold [62]. The physicochemical parameters of the gene products were determined using Molecule Weight and Isoelectric Point Finder (http://greengene.uml.edu/programs/FindMW.html). Transmembrane domains were predicted using TMHMM v2.0 and Phobius or Split 4.0 [63-65]. Sequences of bacterial sigma factors and sigma-factor binding sites were identified through DBTBS database (http://dbtbs.hgc.jp) [39].

Genomic comparisons at the proteomic level were made using CoreGenes [47,48]. For alignments of multiple genomes and defining sequence homology percentage with related phages, progressive Mauve was used [66]. For genomic map visualization and annotation pipelines, the CGview software was used (http://wishart.biology.ualberta. ca/cgview/) [67].

### GenBank accession number

The sequence of genome of this phage has been deposited with GenBank under accession number JX094431.

### Proteomic analysis

Phage Bc431v3 purified through CsCl gradients, was reduced with 10 mM dithiothreitol (56°C, 1 hr) and alkylated by 55 mM iodoacetamide (room temperature, dark, 1 hr), and then dialyzed against 10 mM NH_4_HCO_3_, and dried by SpeedVac concentrator (Savant, Fisher scientific, Nepean, Ontario). Enzymatic digestions were performed on ~20 μg of the purified protein using either sequencing grade trypsin or chymotrypsin (100 ng, Roche Diagnostics GmbH, Indianapolis, IN) for 4 hours. The digests were subsequently diluted by 0.2% formic acid and analyzed by online nanoAcquity ultra-performance liquid chromatography (UPLC, Waters, Milford, MA ) coupled with linear ion-trap Fourier transform ion cyclotron resonance (LTQ-FT ICR, Thermo Fisher, San Jose, CA) mass spectrometry. Peptides were trapped by a RP Symmetry C18 column (180 μm i.d. × 20 mm length, 5 μm) at 5 μl/min, and subsequently separated on a C18 analytical column (100 μm i.d. × 100 mm, 1.7 μm, BEH 130) at 400 nl/min. Peptide elution was achieved using mobile phases consisting of solvent A (0.1% FA) and solvent B (acetonitrile/0.1% FA) at a linear gradient from 5% to 30%, and then 85% of solvent B (65 min run). FT-MS scans were acquired with high resolution (100,000) at the mass range of m/z 300 to 2000, and low resolution MS/MS measurements in linear ion-trap mode were obtained by data-dependent scans of the top eight most intense precursor ions at multiply charged states of 2+, 3+, and 4+. Dynamic exclusion was enabled for a period of 180 S.

Protein identification was performed using an in-house Mascot Server (version 2.3.0, Matrix Science, London, UK), and the raw data were searched against the Bc431v3 protein database. The parameter settings allowed specific trypsin digestion for maximum 2 missed cleavage sites, and non-specific digestion of chymotrypsin. Cystein carbamidomethylation was designated as a fixed modification of peptides, and deamidation of asparagine and glutamine, methionine oxidation, pyro-Glu of Gln conversion at the N-terminus were considered as variable modifications. Mass tolerances were set up to 10 ppm for the FT MS ions and 1 Da for ion trap MS/MS fragment ions. Peptide assignments were filtered by an ion score cut off of 20, and the significance threshold was adjusted to 0.001 to achieve a false discovery rate (FDR) of less than 3% [38].

## Abbreviations

BLAST: Basic Local Alignment Search Tool; dsDNA: Double-stranded deoxyribonucleic acid; EDTA: Ethylenediaminetetraacetic acid; HmUra: 5-Hydroxymethyluracil; MOI: Multiplicity of infection (ratio of phage to bacteria); NCBI: National Center for Biotechnology Information; ORFs: Open reading frames; PFGE: Pulsed-field gel electrophoresis; PFU: Plaque forming unit; TBE: Tris borate EDTA; TSB: Tryptic Soy Broth; TMHMM: TransMembrane prediction using Hidden Markov Models; PEG: Polyethylene Glycol.

## Competing interests

The authors have no competing interests to disclose.

## Authors’ contributions

The bulk of the research and writing was conducted by TFEA, under the supervision of MWG. YMS carried out the proteomic studies. The initial annotation was performed by AV, and then extended by AMK and TFEA. EJL carried out the PFGE analyses. All authors read and approved the final manuscript.

## Authors’ information

TFEA is currently a post-doctoral fellow in Canadian Research Institute for Food Safety, Food Science Department, University of Guelph, Ontario, Canada.

## Supplementary Material

Additional file 1: Table S1 General features of putative ORFs of Bc431v3 and homology to proteins in the database. In addition, protein motifs including transmembrane domains are included. Click here for file

Additional file 2: Table S2 Protein identification of phage Bc431v3 by UPLC LTQ-FT MS/MS analyses. Click here for file

Additional file 3: Table S3 Potential promoters and rho-independent terminators in vB_BceM_Bc431v3 with WebLogo of the consensus. Click here for file

Additional file 4: Table S4 tRNAs discovered in the sequence of Bc341v3 using tRNAScan-SE. Click here for file

Additional file 5: Table S5 Comparison of codon usage in Bc431v3 genes with that of *Bacillus cereus* Q1. Those codons which are 50% more common in the phage are coloured orange. Click here for file
