# Supplementary material for: Genome sequence and analysis of a broad-host range lytic bacteriophage that infects the Bacillus cereus group
Source: Virol J. 2013 Feb 7;10:48. doi: 10.1186/1743-422X-10-48 (PMC3601020; doi:10.1186/1743-422X-10-48)
Supplement: Additional file 1: Table S1 — General features of putative ORFs of Bc431v3 and homology to proteins in the database. In addition, protein motifs including transmembrane domains are included. [file 1743-422X-10-48-S1.doc]

**Additional file 1, Table S1. G**eneral features of putative ORFs of vB_BceM_Bc431v3 and homology to proteins in the database. In addition, protein motifs including transmembrane domains are included.

| **Coordinates**  **(bp)** | **Strand** | **Length** | **Gene**  **(ORF)** | **Mass** | **pI** | **# AA residues** | **Function** | **Homologs and Motifs** |
| --- | --- | --- | --- | --- | --- | --- | --- | --- |
| 594..911 | + | 318 | orf001 | 12194 | 9.6 | 105 | putative regulatory protein | Sequence similarity to: YP_241088.1 ORF187 [Staphylococcus phage G1]; Motifs: HTH_XRE[cd00093], Helix-turn-helix XRE-family like proteins |
| 945..1178 | - | 234 | orf002 | 8541 | 8.0 | 77 | hypothetical protein |  |
| 1198..1518 | - | 321 | orf003 | 12269 | 4.8 | 106 | conserved hypothetical membrane protein | Sequence similarity to: YP_001504329.1| hypothetical protein EFP_gp220 [Enterococcus phage phiEF24C]; Motifs: one transmembrane domain shown using TMHMM and Phobius |
| 1537..1893 | - | 357 | orf005 | 13901 | 5.3 | 118 | conserved hypothetical protein | Sequence similarity to: YP_001468638.1| gp68 [Listeria phage A511] |
| 1874..2080 | - | 207 | orf006 | 7998 | 8.1 | 68 | hypothetical membrane protein | Motifs: two transmembrane domains shown using Phobius and SPLIT |
| 2298..2579 | + | 282 | orf007 | 10663 | 9.6 | 93 | hypothetical membrane protein | Motifs: two transmembrane domains shown using TMHMM and Phobius |
| 2610..3140 | + | 531 | orf008 | 20460 | 4.1 | 176 | hypothetical protein |  |
| 3140..3478 | + | 339 | orf009 | 13087 | 9.6 | 112 | conserved hypothetical protein | Sequence similarity to: YP_001504111.1| hypothetical protein EFP_gp002 [Enterococcus phage phiEF24C] |
| 3423..3836 | + | 414 | orf010 | 15358 | 4.6 | 137 | conserved hypothetical protein | Sequence similarity to: YP_001504112.1| hypothetical protein EFP_gp003 [Enterococcus phage phiEF24C] |
| 3836..5632 | + | 1797 | orf011 | 68433 | 6.5 | 598 | terminase, large subunit | Sequence similarity to: AAS45252.1| large terminase [Bacillus phage 1102phi1-3] |
| 5698..6516 | + | 819 | orf012 | 29365 | 6.4 | 272 | N-acetylmuramoyl-L-alanine amidase | Sequence similarity to: ADF97544.1| PlyM19 [uncultured phage] & NP_844822.1| N-acetylmuramoyl-L-alanine amidase [Bacillus anthracis str. Ames] |
| 6583..7143 | + | 561 | orf013 | 19427 | 5.3 | 186 | conserved hypothetical protein | Sequence similarity to: CAA72265.1| hypothetical protein [Bacillus phage Bastille] |
| 7228..7404 | + | 177 | orf014 | 6698 | 8.0 | 58 | hypothetical protein |  |
| 7404..7583 | + | 180 | orf014A | 6798 | 8.1 | 59 | hypothetical membrane protein | Motifs: two transmembrane domains shown using TMHMM and Phobius |
| 7601..8389 | + | 789 | orf015 | 29759 | 4.6 | 262 | conserved hypothetical membrane protein | Sequence similarity to: YP_001504116.1| hypothetical protein EFP_gp007 [Enterococcus phage phiEF24C]; Motifs: one transmembrane domain shown using TMHMM and Phobius |
| 8389..8703 | + | 315 | orf016 | 11914 | 7.9 | 104 | hypothetical protein |  |
| 8905..9624 | + | 720 | orf017 | 26378 | 7.0 | 239 | PhoH family protein | Sequence similarity to: YP_001857155.1| PhoH family protein [Burkholderia phymatum STM815] |
| 9648..10439 | + | 792 | orf018 | 29926 | 4.4 | 263 | hypothetical protein |  |
| 10544..11956 | + | 1413 | orf019 | 55268 | 8.9 | 470 | conserved hypothetical protein | Sequence similarity to: YP_001504189.1| hypothetical protein EFP_gp080 [Enterococcus phage phiEF24C] |
| 12008..12247 | + | 240 | orf020 | 9220 | 8.4 | 79 | conserved hypothetical protein | Sequence similarity to: YP_238719.1| ORF180 [Staphylococcus phage Twort] |
| 12240..13109 | + | 870 | orf021 | 33067 | 5.8 | 289 | thymidylate synthase | Sequence similarity to: BAI49171.1| hypothetical protein [Bacillus phage phiNIT1]; Motifs: one transmembrane domain shown using TMHMM and SPLIT |
| 13145..13771 | + | 627 | orf022 | 24254 | 5.8 | 208 | conserved hypothetical protein | Sequence similarity to: BAI49172.1| hypothetical protein [Bacillus phage phiNIT1] |
| 13771..14043 | + | 273 | orf023 | 10593 | 5.4 | 90 | hypothetical membrane protein | Motifs: one transmembrane domain shown using TMHMM and Phobius |
| 14154..14654 | + | 501 | orf024 | 19347 | 7.7 | 166 | dihydrofolate reductase | Sequence similarity to: BAI49173.1| hypothetical protein [Bacillus phage phiNIT1] & YP_002505644.1| dihydrofolate reductase region [Clostridium cellulolyticum H10] |
| 14654..14893 | + | 240 | orf025 | 9095 | 4.2 | 79 | conserved hypothetical protein | Sequence similarity to: ZP_07076149.1| predicted protein [Listeria monocytogenes FSL N1-017] & YP_003413444.1| hypothetical protein LM5578_1332 [Listeria monocytogenes 08-5578] |
| 14893..15096 | + | 204 | orf026 | 8018 | 8.9 | 67 | hypothetical protein |  |
| 15108..15374 | + | 267 | orf027 | 10076 | 7.8 | 88 | conserved hypothetical protein | Sequence similarity to: ZP_04189511.1| hypothetical protein bcere0028_56130 [Bacillus cereus AH1271] & ADH03132.1| gp51 [Brochothrix phage BL3] |
| 15371..15619 | + | 249 | orf028 | 9608 | 4.8 | 82 | hypothetical protein |  |
| 15621..15893 | + | 273 | orf029 | 10574 | 5.2 | 90 | conserved hypothetical protein | Sequence similarity to: ZP_04100578.1| hypothetical protein bthur0008_6270 [Bacillus thuringiensis serovar berliner ATCC 10792] & YP_002300464.1| gp34.65 [Bacillus phage SPO1] |
| 15927..16505 | + | 579 | orf030 | 21846 | 5.5 | 192 | conserved hypothetical protein | Sequence similarity to: ZP_00241333.1| conserved hypothetical protein protein [Bacillus cereus G9241] |
| 16524..16721 | + | 198 | orf031 | 7632 | 9.5 | 65 | hypothetical protein |  |
| 16732..16992 | + | 261 | orf032 | 9948 | 4.8 | 86 | hypothetical protein |  |
| 16995..17297 | + | 303 | orf033 | 11934 | 8.1 | 100 | conserved hypothetical protein | Sequence similarity to: YP_002425598.1| hypothetical protein AFE_1126 [Acidithiobacillus ferrooxidans ATCC 23270] |
| 17299..17508 | + | 210 | orf034 | 8160 | 7.7 | 69 | hypothetical protein |  |
| 17610..17798 | + | 189 | orf035 | 7143 | 9.3 | 62 | hypothetical membrane protein | Motifs: one transmembrane domain shown using TMHMM and Phobius |
| 17798..17968 | + | 171 | orf036 | 6482 | 5.7 | 56 | hypothetical protein |  |
| 17968..19155 | + | 1188 | orf037 | 44358 | 6.8 | 395 | conserved hypothetical protein | Sequence similarity to: ZP_04223180.1| hypothetical protein bcere0021_27880 [Bacillus cereus Rock3-42] & YP_003792713.1| hypothetical protein BACI_c29530 [Bacillus anthracis CI] & YP_001468622.1| gp52 [Listeria phage A511] |
| 19167..19385 | + | 219 | orf038 | 8633 | 8.1 | 72 | hypothetical protein |  |
| 19387..19749 | + | 363 | orf039 | 14034 | 4.1 | 120 | hypothetical protein |  |
| 19761..19961 | + | 201 | orf040 | 7605 | 9.1 | 66 | hypothetical protein |  |
| 19961..20890 | + | 930 | orf041 | 35797 | 6.1 | 309 | conserved hypothetical protein | Sequence similarity to: C-terminus of ZP_03227722.1| hypothetical protein Bcoam_18025 [Bacillus coahuilensis m4-4] & YP_078165.1| hypothetical protein BL03189 [Bacillus licheniformis ATCC 14580] |
| 20890..21132 | + | 243 | orf042 | 9355 | 10 | 80 | hypothetical protein |  |
| 21652..21936 | - | 285 | orf043 | 11224 | 4.2 | 94 | hypothetical protein |  |
| 21963..22145 | - | 183 | orf044 | 7328 | 4.7 | 60 | hypothetical protein |  |
| 22150..22251 | - | 102 | orf045A | 3679 | 9.7 | 33 | hypothetical membrane protein | Motifs: one transmembrane domain shown using TMHMM and Phobius |
| 22248..22460 | - | 213 | orf045 | 8211 | 4.3 | 70 | hypothetical protein |  |
| 22489..22848 | - | 360 | orf046 | 13551 | 3.6 | 119 | hypothetical protein |  |
| 23003..23251 | - | 249 | orf047 | 9650 | 5.5 | 82 | hypothetical protein |  |
| 23253..23441 | - | 189 | orf048 | 7233 | 5.9 | 62 | hypothetical protein |  |
| 23434..24108 | - | 675 | orf049 | 25930 | 4.0 | 224 | hypothetical protein |  |
| 24302..24745 | - | 444 | orf050 | 17096 | 4.3 | 147 | hypothetical protein |  |
| 24905..25429 | - | 525 | orf051 | 20056 | 4.2 | 174 | conserved hypothetical protein | Sequence similarity to: YP_551773.1| hypothetical protein Bpro_5003 [Polaromonas sp. JS666] & YP_516054.1| hypothetical protein Rfer_4370 [Rhodoferax ferrireducens T118] |
| 25516..26064 | - | 549 | orf052 | 21102 | 4.1 | 182 | hypothetical protein |  |
| 26214..26333 | - | 120 | orf053A | 4744 | 4.2 | 39 | hypothetical protein |  |
| 26504..26650 | - | 147 | orf053 | 5414 | 6.0 | 48 | hypothetical membrane protein | Motifs: one transmembrane domain shown using TMHMM and Phobius |
| 26644..26874 | - | 231 | orf054 | 8790 | 6.1 | 76 | hypothetical protein |  |
| 26874..27539 | - | 666 | orf055 | 25164 | 5.4 | 221 | conserved hypothetical protein | Sequence similarity to: YP_085030.1| group-specific protein [Bacillus cereus E33L] & ZP_05182902.1| hypothetical protein BantA1_01480 [Bacillus anthracis str. A1055] |
| 27541..27771 | - | 231 | orf056 | 8546 | 9.7 | 76 | hypothetical protein |  |
| 28019..28216 | - | 198 | orf057 | 7226 | 4.7 | 65 | hypothetical protein |  |
| 29079..29486 | - | 408 | orf058 | 15282 | 4.9 | 135 | hypothetical protein |  |
| 29522..29845 | - | 324 | orf059 | 12377 | 6.9 | 107 | hypothetical protein |  |
| 29849..30064 | - | 216 | orf060 | 7778 | 9.7 | 71 | hypothetical membrane protein | Motifs: two transmembrane domains shown using TMHMM and Phobius |
| 30064..30237 | - | 174 | orf061 | 6505 | 9.3 | 57 | hypothetical membrane protein | Motifs: one transmembrane domain shown using TMHMM and Phobius |
| 30234..30401 | - | 168 | orf062 | 6677 | 6.2 | 55 | hypothetical protein |  |
| 30414..30563 | - | 150 | orf063 | 5881 | 4.7 | 49 | hypothetical protein |  |
| 30560..30997 | - | 438 | orf064 | 16534 | 5.3 | 145 | hypothetical membrane protein | Motifs: two transmembrane domains shown using TMHMM and Phobius |
| 30994..31497 | - | 504 | orf065 | 19334 | 8.2 | 167 | hypothetical protein |  |
| 31485..31826 | - | 342 | orf066 | 13612 | 5.3 | 113 | hypothetical protein |  |
| 31838..32011 | - | 174 | orf067 | 7262 | 8.8 | 57 | hypothetical membrane protein | Motifs: one transmembrane domain shown using TMHMM and Phobius |
| 32024..32326 | - | 303 | orf068 | 11442 | 7.9 | 100 | hypothetical protein |  |
| 32345..32956 | - | 612 | orf070 | 22276 | 6.6 | 203 | hypothetical membrane protein | Motifs: one transmembrane domain shown using TMHMM and Phobius |
| 32956..33219 | - | 264 | orf071 | 10286 | 10.2 | 87 | conserved hypothetical protein | Sequence similarity to: YP_024542.1| hypothetical protein KgORF114 [Staphylococcus phage K] & ZP_04007984.1| hypothetical protein HMPREF0528_1516 [Lactobacillus johnsoni ATCC 33200] |
| 33200..33403 | - | 204 | orf072 | 7864 | 5.1 | 67 | hypothetical protein |  |
| 33437..33829 | - | 393 | orf073 | 13319 | 4.6 | 130 | conserved hypothetical protein | Sequence similarity to: YP_003965790.1| hypothetical protein PPSC2_p0331 [Paenibacillus polymyxa SC2] & YP_002408622.1| hypothetical protein ECIAI39_2683 [Escherichia coli IAI39] & NP_899386.1| hypothetical protein KVP40.0139 [Vibrio phage KVP40] |
| 33831..34013 | - | 183 | orf074 | 6746 | 4.4 | 60 | hypothetical protein |  |
| 34125..34523 | - | 399 | orf075 | 15740 | 5.5 | 132 | hypothetical protein |  |
| 34530..34964 | - | 435 | orf076 | 16074 | 6.3 | 144 | hypothetical protein |  |
| 34966..35325 | - | 360 | orf077 | 13778 | 6.6 | 119 | conserved hypothetical membrane protein | Sequence similarity to: ZP_04231038.1| hypothetical protein bcere0020_53370 [Bacillus cereus Rock3-29] & ZP_04237035.1| hypothetical protein bcere0019_56010 [Bacillus cereus Rock3-28] & YP_001331320.1| hypothetical protein NWMN_0286 [Staphylococcus aureus subsp. aureus str. Newman] & ABF73263.1| hypothetical protein [Staphylococcus aureus phage phiNM4]; Motifs: one transmembrane domain shown using TMHMM and Phobius |
| 35340..36530 | - | 1191 | orf078 | 47139 | 5.8 | 396 | conserved hypothetical protein | Sequence similarity to: ZP_05399400.1| hypothetical protein CdifQCD_20116 [Clostridium difficile QCD-37x79] |
| 36558..37334 | - | 777 | orf079 | 29581 | 5.0 | 258 | metal-dependent hydrolase | Sequence similarity to: ZP_04068850.1| Metal-dependent hydrolase [Bacillus thuringiensis IBL 4222] & YP_176632.1| metal-dependent hydrolase [Bacillus clausii KSM-K16] & YP_176632.1| metal-dependent hydrolase [Bacillus clausii KSM-K16]; Motifs:Lactamase_B[smart00849], Metallo-beta-lactamase superfamily & Lactamase_B[pfam00753], Metallo-beta-lactamase superfamily |
| 37327..37464 | - | 138 | orf080 | 5239 | 4.1 | 45 | hypothetical protein |  |
| 37477..37806 | - | 330 | orf081 | 12327 | 5.4 | 109 | hypothetical protein |  |
| 37806..37889 | - | 84 | orf082A | 2958 | 3.8 | 27 | hypothetical protein |  |
| 37905..38993 | - | 1089 | orf082 | 42879 | 5.7 | 362 | conserved hypothetical protein | Sequence similarity to: ZP_06842980.1| hypothetical protein BCh11DRAFT_4247 [Burkholderia sp. Ch1-1] |
| 38993..39166 | - | 174 | orf083 | 6723 | 5.3 | 57 | hypothetical protein |  |
| 39163..39456 | - | 294 | orf084 | 11190 | 6.4 | 97 | hypothetical protein |  |
| 39453..39662 | - | 210 | orf085 | 8034 | 5.7 | 69 | hypothetical protein |  |
| 39665..40060 | - | 396 | orf086 | 14965 | 5.4 | 131 | conserved hypothetical protein | Sequence similarity to: ZP_05332268.1| hypothetical protein CdifQCD-6_20935 [Clostridium difficile QCD-63q42] & YP_003424038.1| hypothetical protein mru_1296 [Methanobrevibacter ruminantium M1] |
| 40100..42136 | - | 2037 | orf087 | 77295 | 6.4 | 678 | conserved hypothetical protein | Sequence similarity to: ZP_05332267.1| hypothetical protein CdifQCD-6_20930 [Clostridium difficile QCD-63q42] & YP_003424039.1| hypothetical protein mru_1297 [Methanobrevibacter ruminantium M1] & YP_003358621.1| hypothetical protein [Shigella phage phiSboM-AG3] |
| 42175..42573 | - | 399 | orf088 | 15934 | 4.3 | 132 | conserved hypothetical protein | Sequence similarity to: hypothetical protein bthur0006_2390 [Bacillus thuringiensis serovar kurstaki str. T03a001] |
| 42573..42749 | - | 177 | orf089 | 6571 | 4.5 | 58 | hypothetical protein |  |
| 42773..42985 | - | 213 | orf090 | 8470 | 9.0 | 70 | conserved hypothetical protein | Sequence similarity to: hypothetical protein bthur0002_60280 [Bacillus thuringiensis Bt407] |
| 43064..43240 | - | 177 | orf091 | 6478 | 8.0 | 58 | hypothetical membrane protein | Motifs: two transmembrane domains shown using TMHMM and Phobius |
| 43397..43513 | - | 117 | orf093 | 4507 | 6.5 | 38 | hypothetical protein |  |
| 43545..43922 | - | 378 | orf094 | 14139 | 4.7 | 125 | hypothetical membrane protein | Motifs: two transmembrane domains shown using TMHMM and Phobius |
| 44002..44313 | - | 312 | orf095 | 11653 | 5.2 | 103 | hypothetical protein |  |
| 44330..44515 | - | 186 | orf096 | 6997 | 7.9 | 61 | hypothetical protein |  |
| 44896..45579 | - | 684 | orf097 | 24694 | 8.4 | 227 | hypothetical protein |  |
| 45687..48008 | - | 2322 | orf098 | 87236 | 5.8 | 773 | FtsK_SpoIIIE-family protein | Sequence similarity to: ZP_03115151.1| DNA translocase stage III sporulation protein [Bacillus cereus 03BB108] & YP_003702382.1 cell division protein FtsK/SpoIIIE [Syntrophothermus lipocalidus DSM 12680]; Motifs: FtsK_SpoIIIE[pfam01580], FtsK/SpoIIIE family & FtsK[COG1674], DNA segregation ATPase FtsK/SpoIIIE and related proteins |
| 47998..48114 | - | 117 | orf099 | 4296 | 9.7 | 38 | hypothetical protein |  |
| 48126..49142 | - | 1017 | orf100 | 37946 | 9.8 | 338 | conserved hypothetical membrane protein | Sequence similarity to: ZP_04254516.1| hypothetical protein bcere0016_56800 [Bacillus cereus 95/8201] & ZP_04242683.1| hypothetical protein bcere0018_53970 [Bacillus cereus Rock1-15]; Motifs: 5-7 transmembrane domains shown using TMHMM and Phobius |
| 49280..49813 | - | 534 | orf101 | 19203 | 9.9 | 177 | hypothetical membrane protein | Motifs: three transmembrane domains shown using TMHMM and Phobius |
| 49846..50031 | - | 186 | orf102 | 6508 | 10.0 | 61 | hypothetical protein |  |
| 50117..50278 | + | 162 | orf103 | 6664 | 5.5 | 53 | hypothetical protein |  |
| 50442..50756 | - | 315 | orf104 | 12042 | 9.7 | 104 | hypothetical protein |  |
| 50768..51865 | - | 1098 | orf105 | 40969 | 7.1 | 365 | conservered hypothetical protein; putative segregation protein | Sequence similarity to: ZP_04861681.1| conserved hypothetical protein [Clostridium botulinum D str. 1873] & YP_879207.1| hypothetical protein NT01CX_0741 [Clostridium novyi NT] & ZP_02622065.1| conserved hypothetical protein [Clostridium botulinum C str.Eklund] |
| 52008..52241 | - | 234 | orf106 | 8731 | 5.2 | 77 | hypothetical protein |  |
| 52243..52743 | - | 501 | orf107 | 18426 | 9.7 | 166 | hypothetical protein |  |
| 52745..53497 | - | 753 | orf108 | 29030 | 9.0 | 250 | RNA polymerase sigma-70 factor, sigma-B/F/G subfamily | Sequence similarity to: ACL00355.1| Bcp25 [Bacillus phage Bcp1] & YP_001112465.1 FliA/WhiG family RNA polymerase sigma factor [Desulfotomaculum reducens MI-1]; Motifs: spore_sigF[TIGR02885], RNA polymerase sigma-F factor & FliA[COG1191], DNA-directed RNA polymerase specialized sigma subunit & PRK07408[PRK07408], RNA polymerase sigma factor SigF |
| 53514..54284 | - | 771 | orf109 | 29451 | 5.5 | 256 | putative RNA polymerase sigma factor G | Sequence similarity to: ACL00354.1| Bcp26 [Bacillus phage Bcp1] & YP_003840016.1 RNA polymerase, sigma 28 subunit, FliA/WhiG subfamily [Caldicellulosiruptor obsidiansis OB47]; Motifs: PRK05572[PRK05572], sporulation sigma factor SigF & SigBFG[TIGR02980], RNA polymerase sigma-70 factor, sigma-B/F/G subfamily & PRK08215[PRK08215], sporulation sigma factor SigG |
| 54452..54931 | - | 480 | orf110 | 17754 | 4.6 | 159 | hypothetical protein |  |
| 54933..55547 | - | 615 | orf111 | 23184 | 4.8 | 204 | hypothetical protein |  |
| 55562..55810 | - | 249 | orf112 | 9445 | 6.6 | 82 | hypothetical protein |  |
| 55810..56019 | - | 210 | orf113 | 7696 | 4.8 | 69 | hypothetical protein |  |
| 56066..56893 | - | 828 | orf114 | 30483 | 5.3 | 275 | hypothetical protein |  |
| 57046..57258 | - | 213 | orf115 | 7618 | 9.5 | 70 | hypothetical membrane protein | Motifs: two transmembrane domains shown using TMHMM and Phobius |
| 57270..57515 | - | 246 | orf116 | 9204 | 8.8 | 81 | hypothetical membrane protein | Motifs: two transmembrane domains shown using TMHMM and Phobius |
| 57512..57832 | - | 321 | orf117 | 12331 | 5.4 | 106 | hypothetical protein |  |
| 57829..58458 | - | 630 | orf118 | 24919 | 5.2 | 209 | hypothetical protein |  |
| 58460..59056 | - | 597 | orf119 | 22428 | 7.0 | 198 | conserved hypothetical protein | Sequence similarity to: C-terminus of YP_001468547.1| gp167 [Listeria phage A511] & YP_001504192.1| hypothetical protein EFP_gp083 [Enterococcus phage phiEF24C] |
| 59175..59654 | - | 480 | orf120 | 18717 | 4.9 | 159 | conserved hypothetical protein | Sequence similarity to: AAY53400.1| gp97 [Listeria phage P100] & YP_001468546.1| gp166 [Listeria phage A511] |
| 59669..60391 | - | 723 | orf121 | 26997 | 4.9 | 240 | conserved hypothetical protein | Sequence similarity to: YP_001504190.1| hypothetical protein EFP_gp081 [Enterococcus phage phiEF24C] & AAY53399.1| gp96 [Listeria phage P100] |
| 60718..61572 | - | 855 | orf122 | 32235 | 4.8 | 284 | conserved hypothetical protein | Sequence similarity to: YP_001468534.1| gp154 [Listeria phage A511] & AAY53389.1| gp86 [Listeria phage P100] |
| 61572..61766 | - | 195 | orf123 | 7581 | 4.3 | 64 | hypothetical protein |  |
| 61766..62305 | - | 540 | orf124 | 20380 | 10.6 | 179 | hypothetical protein | Sequence similarity to: YP_001468533.1| gp153 [Listeria phage A511] & YP_001504185.1| hypothetical protein EFP_gp076 [Enterococcus phage phiEF24C] |
| 62298..63065 | - | 768 | orf125 | 29635 | 8.9 | 255 | conserved hypothetical protein | Sequence similarity to: YP_224229.1| hypothetical protein LPPPVgp01 [Listonella phage phiHSIC] & ZP_07135017.1| conserved domain protein [Escherichia coli MS 115-1] |
| 63171..63722 | - | 552 | orf126 | 21164 | 5.3 | 183 | hypothetical protein |  |
| 63902..64672 | - | 771 | orf127 | 30126 | 8.6 | 256 | conserved hypothetical protein | Sequence similarity to: YP_224229.1| hypothetical protein LPPPVgp01 [Listonella phage phiHSIC] & ZP_04670588.1| conserved hypothetical protein [Clostridiales bacterium 1_7_47_FAA] |
| 64711..65427 | - | 717 | orf128 | 27423 | 9.2 | 238 | conserved hypothetical protein | Sequence similarity to: YP_001504184.1| hypothetical protein EFP_gp075 [Enterococcus phage phiEF24C] & AAY53387.1| gp84 [Listeria phage P100] |
| 65482..66234 | - | 753 | orf129 | 29161 | 6.7 | 250 | hypothetical protein |  |
| 66237..66446 | - | 210 | orf130 | 7871 | 9.2 | 69 | hypothetical membrane protein | Motifs: two transmembrane domains shown using TMHMM and Phobius |
| 66436..66795 | - | 360 | orf131 | 13404 | 9.6 | 119 | hypothetical membrane protein | Motifs: four transmembrane domains shown using TMHMM and Phobius |
| 66797..67252 | - | 456 | orf132 | 17387 | 7.8 | 151 | conserved hypothetical protein | Sequence similarity to: NP_943990.1| hypothetical protein Aeh1p112 [Aeromonas phage Aeh1] |
| 67275..67832 | - | 558 | orf133 | 20713 | 9.0 | 185 | conserved hypothetical protein | Sequence similarity to: YP_001468530.1| gp150 [Listeria phage A511] & AAY53385.1| gp82 [Listeria phage P100] |
| 67913..68077 | - | 165 | orf134 | 6472 | 4.4 | 54 | conserved hypothetical protein | Sequence similarity to: YP_002449542.1| hypothetical protein BCAH820_0569 [Bacillus cereus AH820] |
| 68079..68369 | - | 291 | orf135 | 11115 | 4.9 | 96 | hypothetical protein |  |
| 68366..68773 | - | 408 | orf136 | 15952 | 4.8 | 135 | hypothetical protein |  |
| 68794..69192 | - | 399 | orf137 | 14569 | 6.7 | 132 | conserved hypothetical protein | Sequence similarity to: ZP_04075859.1| hypothetical protein bthur0013_62310 [Bacillus thuringiensis IBL 200] & YP_002300321.1| gp1.2 [Bacillus phage SPO1] |
| 69194..69406 | - | 213 | orf138 | 7762 | 9.1 | 70 | conserved hypothetical protein | Sequence similarity to: ZP_04167552.1| hypothetical protein bmyco0001_8070 [Bacillus mycoides DSM 2048] |
| 69419..69634 | - | 216 | orf139 | 7952 | 6.6 | 71 | hypothetical protein |  |
| 69650..69991 | - | 342 | orf140 | 13162 | 4.4 | 113 | hypothetical protein |  |
| 69993..70271 | - | 279 | orf141 | 11108 | 5.1 | 92 | hypothetical protein |  |
| 70285..70671 | - | 387 | orf142 | 14863 | 5.4 | 128 | hypothetical protein |  |
| 70673..71407 | - | 735 | orf143 | 28282 | 8.8 | 244 | conserved hypothetical protein, putative tRNAHis guanylyltransferase | Sequence similarity to: ZP_00738534.1| Hypothetical protein RBTH_06728 [Bacillus thuringiensis serovar israelensis ATCC 35646] & YP_003947904.1| protein [Paenibacillus polymyxa SC2]; Motifs: COG4021[COG4021], Uncharacterized conserved protein & Thg1[pfam04446], tRNAHis guanylyltransferase |
| 71487..71777 | - | 291 | orf144 | 11701 | 4.9 | 96 | hypothetical protein |  |
| 71779..72132 | - | 354 | orf145 | 13674 | 5.2 | 117 | hypothetical protein |  |
| 72134..72613 | - | 480 | orf146 | 18096 | 5.1 | 159 | hypothetical protein |  |
| 72711..72854 | - | 144 | orf147 | 5733 | 9.4 | 47 | hypothetical protein |  |
| 72856..73140 | - | 285 | orf148 | 10978 | 4.8 | 94 | hypothetical protein |  |
| 73155..73358 | - | 204 | orf149 | 7872 | 5.1 | 67 | hypothetical protein |  |
| 73361..73786 | - | 426 | orf150 | 16621 | 5.4 | 141 | hypothetical protein |  |
| 73808..74077 | - | 270 | orf152 | 10809 | 5.2 | 89 | hypothetical protein |  |
| 74038..74544 | - | 507 | orf153 | 20018 | 7.7 | 168 | predicted methyltransferase | Sequence similarity to: YP_686174.1| SAM-dependent methyltransferase [uncultured methanogenic archaeon RC-I] & ZP_06232159.1| Methyltransferase type 11 [Desulfovibrio aespoeensis Aspo-2]; Motifs: Methyltransf_11[pfam08241], Methyltransferase domain & PLN02336[PLN02336], phosphoethanolamine N-methyltransferase |
| 74570..74761 | - | 192 | orf154 | 7642 | 9.7 | 63 | hypothetical protein |  |
| 74758..74940 | - | 183 | orf155 | 7327 | 6.3 | 60 | hypothetical protein |  |
| 74942..75136 | - | 195 | orf156 | 7180 | 4.1 | 64 | hypothetical protein |  |
| 75250..75633 | - | 384 | orf157 | 14305 | 9.0 | 127 | hypothetical membrane protein | Motifs: three transmembrane domains shown using TMHMM and Phobius |
| 75647..76954 | - | 1308 | orf158 | 49418 | 5.9 | 435 | conserved hypothetical protein | Sequence similarity to: AAY53383.1| gp80 [Listeria phage P100] & YP_001468528.1| gp148 [Listeria phage A511] |
| 76977..77231 | - | 255 | orf159 | 9300 | 5.3 | 84 | hypothetical protein |  |
| 77283..78242 | - | 960 | orf160 | 35668 | 5.3 | 319 | conserved hypothetical protein | Sequence similarity to: AAY53382.1| gp79 [Listeria phage P100] & YP_001468527.1| gp147 [Listeria phage A511] |
| 78203..78376 | - | 174 | orf161A | 6699 | 9.1 | 57 | hypothetical protein |  |
| 78401..78877 | - | 477 | orf161 | 17195 | 7.0 | 158 | hypothetical protein |  |
| 78877..79176 | - | 300 | orf162 | 11006 | 6.0 | 99 | hypothetical membrane protein | Motifs: two transmembrane domains shown using TMHMM and Phobius |
| 79228..79866 | - | 639 | orf163 | 25292 | 5.4 | 212 | putative sigma factor | Sequence similarity to: YP_001468523.1| gp143 [Listeria phage A511] & AAY53377.1| gp74 [Listeria phage P100] & YP_001504175.1| Gene info linked to YP_001504175.1 putative sigma factor [Enterococcus phage phiEF24C] |
| 79859..80209 | - | 351 | orf164 | 13465 | 6.3 | 116 | conserved hypothetical protein | Sequence similarity to: YP_240963.1| ORF121 [Staphylococcus phage G1] & YP_001504174.1| hypothetical protein EFP_gp065 [Enterococcus phage phiEF24C] |
| 80253..81464 | - | 1212 | orf165 | 44084 | 5.6 | 403 | putative recA-like recombinase | Sequence similarity to: ACU27400.1| recombination repair protein [Bacillus phage Bcp1] & YP_001468521.1| gp141 [Listeria phage A511] & AAY53375.1| gp72 [Listeria phage P100] |
| 81527..81907 | - | 381 | orf166 | 14435 | 6.2 | 126 | conserved hypothetical protein; putative structural protein | Sequence similarity to: ZP_04271191.1| hypothetical protein bcere0013_57720 [Bacillus cereus BDRD-ST26] & YP_001828719.1| structural protein [Lactococcus phage 1706] |
| 82004..83302 | - | 1299 | orf167 | 46317 | 5.2 | 432 | conserved hypothetical protein | Sequence similarity to: YP_001468520.1| gp140 [Listeria phage A511] & AAY53374.1| gp71 [Listeria phage P100] & YP_001504172.1| hypothetical protein EFP_gp063 [Enterococcus phage phiEF24C] |
| 83409..83744 | - | 336 | orf168 | 12252 | 4.2 | 111 | hypothetical protein |  |
| 83744..84280 | - | 537 | orf169 | 20982 | 5.2 | 178 | conserved hypothetical protein | Sequence similarity to: YP_001468519.1| gp139 [Listeria phage A511] & AAY53373.1| gp70 [Listeria phage P100] & YP_001504171.1| hypothetical protein EFP_gp062 [Enterococcus phage phiEF24C] |
| 84385..87501 | - | 3117 | orf170 | 120391 | 6.4 | 1038 | DNA polymerase | Sequence similarity to:YP_001504170.1| putative DNA polymerase [Enterococcus phage phiEF24C] & YP_024516.1| putative DNA polymerase [Staphylococcus phage K] & YP_238608.1| ORF004 [Staphylococcus phage Twort] |
| 87517..87675 | - | 159 | orf171 | 5922 | 4.7 | 52 | hypothetical membrane protein | Motifs: one transmembrane domain shown using TMHMM and Phobius |
| 87785..88450 | - | 666 | orf172 | 25105 | 8.2 | 221 | C-5 cytosine-specific DNA methylase I | Sequence similarity to:Motifs: Cyt_C5_DNA_methylase[cd00315], Cytosine-C5 specific DNA methylases & Dcm[COG0270], Site-specific DNA methylase ; YP_001885133.1| DNA methyltransferase [Clostridium botulinum B str. Eklund 17B] |
| 88452..89474 | - | 1023 | orf173 | 38257 | 5.4 | 340 | C-5 cytosine-specific DNA methylase II | Sequence similarity to: ZP_07461376.1| DNA (cytosine-5-)-methyltransferase [Streptococcus pyogenes ATCC 0782] & YP_003307635.1| DNA-cytosine methyltransferase [Sebaldella termitidis ATCC 33386]; Motifs: Cyt_C5_DNA_methylase |
| 89576..89875 | - | 300 | orf174 | 11388 | 9.4 | 99 | putative DNA-binding protein | Sequence similarity to:YP_001504169.1| putative integration host factor [Enterococcus phage phiEF24C] & YP_024515.1| putative integration host factor [Staphylococcus phage K; Motifs: Bac_DNA_binding[pfam00216], Bacterial DNA-binding protein & HU_IHF[cd00591], Integration host factor (IHF) and HU |
| 89877..90635 | - | 759 | orf175 | 30067 | 8.2 | 252 | conserved hypothetical protein | Sequence similarity to:YP_001468515.1| gp135 [Listeria phage A511] |
| 90786..91028 | - | 243 | orf176 | 9166 | 9.8 | 80 | hypothetical protein |  |
| 91028..91780 | - | 753 | orf177 | 28377 | 5.7 | 250 | conserved hypothetical protein, predicted nucleotidyltransferase | Sequence similarity to: ZP_00738350.1| hypothetical protein RBTH_07153 [Bacillus thuringiensis serovar israelensis ATCC 35646] & YP_120176.1| hypothetical protein nfa39640 [Nocardia farcinica IFM 10152] ; Motifs: Nuc-transf[pfam10127] |
| 91782..91904 | - | 123 | orf178 | 4818 | 5.3 | 40 | hypothetical protein |  |
| 91906..92220 | - | 315 | orf179 | 11777 | 4.2 | 104 | hypothetical protein |  |
| 92233..92511 | - | 279 | orf180 | 10393 | 8.6 | 92 | hypothetical protein |  |
| 92513..92737 | - | 225 | orf181 | 8189 | 4.9 | 74 | hypothetical protein |  |
| 92809..93456 | - | 648 | orf182 | 25437 | 9.4 | 215 | hypothetical protein |  |
| 93557..94687 | - | 1131 | orf183 | 43460 | 5.3 | 376 | ribonucleotide reductase, beta subunit; putative transmembrane protein | Sequence similarity to: YP_002314759.1| ribonucleotide-diphosphate reductase subunit beta [Anoxybacillus flavithermus WK1] & YP_001814761.1| ribonucleotide-diphosphate reductase subunit beta [Exiguobacterium sibiricum 255-15]; Motifs: one transmembrane domain shown using TMHMM and Phobius |
| 94770..94958 | - | 189 | orf184 | 7523 | 5.7 | 62 | hypothetical protein |  |
| 94958..97297 | - | 2340 | orf185 | 88231 | 5.6 | 779 | ribonucleoside-diphosphate reductase, alpha subunit | Sequence similarity to: ZP_06811026.1| ribonucleoside-diphosphate reductase, alpha subunit [Geobacillus thermoglucosidasius C56-YS93] & YP_002948760.1| ribonucleoside-diphosphate reductase, alpha subunit [Geobacillus sp. WCH70] |
| 97329..97898 | - | 570 | orf186 | 21460 | 7.8 | 189 | conserved hypothetical protein | Sequence similarity to: YP_240945.1| ORF064 [Staphylococcus phage G1] |
| 97898..98161 | - | 264 | orf187 | 10119 | 4.6 | 87 | conserved hypothetical protein | Sequence similarity to: YP_002736883.1| hypothetical protein SPJ_1865 [Streptococcus pneumoniae JJA] & ZP_07341708.1| hypothetical protein CGSSpBS455_09202 [Streptococcus pneumoniae BS455] |
| 98161..98541 | - | 381 | orf188 | 14388 | 5.0 | 126 | hypothetical protein |  |
| 98525..98863 | - | 339 | orf189 | 12682 | 4.5 | 112 | hypothetical protein |  |
| 98869..99681 | - | 813 | orf190 | 30253 | 5.2 | 270 | putative deoxyuridine 5'-triphosphate nucleotidohydrolase | Sequence similarity to: ZP_07327522.1| deoxyuridine 5'-triphosphate nucleotidohydrolase Dut [Acetivibrio cellulolyticus CD2] ; Motifs:dut[PRK00601] |
| 99751..100023 | - | 273 | orf191 | 10888 | 4.4 | 90 | conserved hypothetical protein | Sequence similarity to: ZP_04112593.1| hypothetical protein bthur0007_64890 [Bacillus thuringiensis monterrey BGSC 4AJ1] |
| 100038..100190 | - | 153 | orf192A | 6065 | 9.5 | 50 | hypothetical membrane protein | Motifs: one transmembrane domain shown using TMHMM and Phobius |
| 100197..101279 | - | 1083 | orf192 | 41843 | 5.1 | 360 | DNA primase | Sequence similarity to: YP_001468497.1 gp117 [Listeria phage A511] & YP_001504158.1 putative primase [Enterococcus phage phiEF24C]; Motif: dnaG[TIGR01391], DNA primase & DnaG[COG0358], DNA primase |
| 101292..101864 | - | 573 | orf193 | 22351 | 5.7 | 190 | conserved hypothetical protein | Sequence similarity to: YP_238590.1| ORF065 [Staphylococcus phage Twort] & YP_002790821.1| hypothetical protein lb338_phage_142 [Lactobacillus phage Lb338-1] |
| 101861..103753 | - | 1893 | orf194 | 71873 | 6.7 | 630 | putative exonuclease I | Sequence similarity to: YP_001468495.1| gp115 [Listeria phage A511] & YP_024504.1| putative exonuclease [Staphylococcus phage K] |
| 103862..104950 | - | 1089 | orf195 | 41640 | 5.2 | 362 | putative exonuclease II | Sequence similarity to: YP_001468494.1| gp114 [Listeria phage A511] & YP_001504155.1| putative exonuclease [Enterococcus phage phiEF24C] |
| 104940..105293 | - | 354 | orf196 | 13564 | 4.0 | 117 | hypothetical protein |  |
| 105298..106761 | - | 1464 | orf197 | 55503 | 6.3 | 487 | DNA helicase I | Sequence similarity to: YP_001468493.1| gp113 [Listeria phage A511] & YP_001504154.1| putative helicase [Enterococcus phage phiEF24C]; Motifs: DnaB_C[cd00984], DnaB helicase & DnaB_C[pfam03796], DnaB-like helicase |
| 106842..108512 | - | 1671 | orf198 | 63949 | 6.0 | 556 | putative transcriptional regulator | Sequence similarity to: ADJ53148.1| gp114 [Brochothrix phage A9] & AAY53346.1| gp43 [Listeria phage P100] & YP_001468492.1 gp112 [Listeria phage A511] |
| 108591..110363 | - | 1773 | orf200 | 67956 | 8.9 | 590 | DNA helicase II | Sequence similarity to: AAY53345.1| gp42 [Listeria phage P100]& YP_001468491.1| gp111 [Listeria phage A511] & YP_001504152.1| putative helicase [Enterococcus phage phiEF24C]; Motifs: Helicase_C[pfam00271] |
| 110385..110669 | - | 285 | orf201 | 10720 | 5.1 | 94 | conserved hypothetical protein | Sequence similarity to: ZP_00738311.1| hypothetical protein RBTH_07102 [Bacillus thuringiensis serovar israelensis ATCC 35646] |
| 111181..111372 | - | 192 | orf202 | 7014 | 7.9 | 63 | hypothetical protein | Sequence similarity to: |
| 111385..114879 | - | 3495 | orf203 | 129400 | 5.2 | 1164 | putative tail protein | Sequence similarity to: YP_001504150.1| putative adsorption associated tail protein [Enterococcus phage phiEF24C] & AAY53340.1| gp37 [Listeria phage P100]; Postulated structural protein by homology with Listeria phage A511 structural proteins |
| 114894..115919 | - | 1026 | orf204 | 38232 | 8.3 | 341 | conserved hypothetical protein, putative structural protein | Sequence similarity to: YP_001468484.1| gp104 [Listeria phage A511] 7 YP_238576.1| ORF005 [Staphylococcus phage Twort]; Postulated structural protein by homology with Listeria phage A511 structural proteins; Postulated structural protein by homology with Listeria phage A511 structural proteins |
| 115930..116976 | - | 1047 | orf205 | 38864 | 6.0 | 348 | conserved hypothetical protein, putative baseplate protein | Sequence similarity to: YP_001468483.1| gp103 [Listeria phage A511] & AAY53337.1| gp34 [Listeria phage P100] & YP_001504147.1| putative structural protein [Enterococcus phage phiEF24C]; ; Motifs: XkdT[COG3299], Uncharacterized homolog of phage Mu protein gp47 & Baseplate_J[pfam04865], Baseplate J-like protein; Postulated structural protein by homology with Listeria phage A511 structural proteins |
| 116990..117739 | - | 750 | orf206 | 28107 | 5.1 | 249 | conserved hypothetical protein, putative baseplate protein | Sequence similarity to: AAY53336.1| gp33 [Listeria phage P100] & YP_001468482.1| gp102 [Listeria phage A511] & YP_024491.1| putative bacteriophage baseplate protein [Staphylococcus phage K]; Postulated structural protein by homology with Listeria phage A511 structural proteins |
| 117739..118263 | - | 525 | orf207 | 20020 | 6.3 | 174 | conserved hypothetical membrane protein | Sequence similarity to: AAY53335.1| gp32 [Listeria phage P100] & YP_001468481.1| gp101 [Listeria phage A511] & YP_001504145.1| hypothetical protein EFP_gp036 [Enterococcus phage phiEF24C]; Motifs: one transmembrane domain shown using Phobius and SPLIT |
| 118268..119095 | - | 828 | orf208 | 29773 | 6.0 | 275 | conserved hypothetical protein | Sequence similarity to: YP_001468480.1| gp100 [Listeria phage A511] & AAY53334.1| gp31 [Listeria phage P100] & YP_001504144.1| hypothetical protein EFP_gp035 [Enterococcus phage phiEF24C] |
| 119147..119632 | - | 486 | orf209 | 18969 | 7.8 | 161 | conserved hypothetical protein | Sequence similarity to: ZP_03598292.1| hypothetical protein BsubsN3_22509 [Bacillus subtilis subsp. subtilis str. NCIB 3610] & YP_001642664.1| hypothetical protein BcerKBAB4_5738 [Bacillus weihenstephanensis KBAB4] |
| 119648..119833 | - | 186 | orf210 | 7045 | 4.8 | 61 | hypothetical protein |  |
| 119858..120373 | - | 516 | orf211 | 19650 | 6.2 | 171 | conserved hypothetical protein | Sequence similarity to: ZP_03598341.1| hypothetical protein BsubsN3_22754 [Bacillus subtilis subsp. subtilis str. NCIB 3610] & YP_001642410.1| hypothetical protein BcerKBAB4_5443 [Bacillus weihenstephanensis KBAB4] |
| 120388..123072 | - | 2685 | orf212 | 96651 | 5.7 | 894 | putative minor structural protein | Sequence similarity to: YP_001504141.1| putative minor structural protein [Enterococcus phage phiEF24C] & ZP_03777394.1| hypothetical protein CLOHYLEM_04446 [Clostridium hylemonae DSM 15053] |
| 123094..127161 | - | 4068 | orf213 | 149850 | 5.6 | 1355 | phage minor structural protein | Sequence similarity to: YP_001504140.1| putative tail fiber [Enterococcus phage phiEF24C] |
| 127250..128653 | - | 1404 | orf214 | 48939 | 5.3 | 467 | conserved hypothetical protein | Sequence similarity to: ZP_04227935.1| hypothetical protein bcere0020_22130 [Bacillus cereus Rock3-29] & YP_001642407.1| hypothetical protein BcerKBAB4_5440 [Bacillus weihenstephanensis KBAB4] |
| 128674..130710 | - | 2037 | orf215 | 75788 | 4.9 | 678 | phage minor structural protein, putative tail fiber | Sequence similarity to: YP_001504140.1| putative tail fiber [Enterococcus phage phiEF24C] & AAY53333.1| gp30 [Listeria phage P100] |
| 130750..132987 | - | 2238 | orf216 | 82139 | 5.9 | 745 | conserved hypothetical protein | Sequence similarity to: YP_001468478.1| gp98 [Listeria phage A511] & AAY53332.1| gp29 [Listeria phage P100] |
| 133030..136881 | - | 3852 | orf218 | 136343 | 9.5 | 1283 | putative tail protein possessing endo-beta-N-acetylglucosaminidase activity; putative membrane protein | Sequence similarity to: AAY53331.1| gp28 [Listeria phage P100] & YP_001468477.1| gp97 [Listeria phage A511] & YP_001504138.1| putative tail lysin [Enterococcus phage phiEF24C]; Motifs: C-terminus LytD[COG4193], Beta- N-acetylglucosaminidase & LYZ2[smart00047], Lysozyme subfamily 2 & Glucosaminidase[pfam01832], Mannosyl-glycoprotein endo-beta-N-acetylglucosaminidase domains; Motifs: three transmembrane domains shown using TMHMM and SPLIT |
| 136936..137487 | - | 552 | orf219 | 20905 | 4.5 | 183 | conserved hypothetical protein | Sequence similarity to:YP_001468476.1| gp96 [Listeria phage A511] & AAY53330.1| gp27 [Listeria phage P100] & YP_001504137.1| putative RNA polymerase [Enterococcus phage phiEF24C] |
| 137555..137974 | - | 420 | orf220 | 16127 | 4.9 | 139 | conserved hypothetical protein | Sequence similarity to: YP_001504136.1| hypothetical protein EFP_gp027 [Enterococcus phage phiEF24C] & YP_001468475.1| gp95 [Listeria phage A511] |
| 138083..138988 | - | 906 | orf221 | 34364 | 5.0 | 301 | hypothetical protein |  |
| 139125..139787 | - | 663 | orf222 | 23676 | 8.7 | 220 | L-alanoyl-D-glutamate peptidase | Sequence similarity to: YP_001376959.1| 3D domain-containing protein [Bacillus cereus subsp. cytotoxis NVH 391-98]; Motifs: COG3584[COG3584], Uncharacterized protein conserved in bacteria & 3D[pfam06725], 3D domain & MltA[COG2821], Membrane-bound lytic murein transglycosylase |
| 139923..142256 | - | 2334 | orf223 | 85055 | 6.2 | 777 | conserved hypothetical protein | Sequence similarity to: ZP_04296147.1| hypothetical protein bcere0007_33800 [Bacillus cereus AH621] & ZP_04208583.1| hypothetical protein bcere0024_34060 [Bacillus cereus Rock4-18] |
| 142337..142765 | - | 429 | orf224 | 15921 | 5.0 | 142 | conserved hypothetical protein, putative tail protein | Sequence similarity to: YP_001468474.1| gp94 [Listeria phage A511]; Postulated structural protein by homology with Listeria phage A511 structural proteins |
| 142820..144526 | - | 1707 | orf225 | 61121 | 5.4 | 568 | tail sheath protein | Sequence similarity to: YP_001469620.1| tail sheath protein [Listeria phage A511] & YP_001468473.1| tail sheath protein precursor [Listeria phage A511] |
| 144556..144771 | - | 216 | orf226 | 8108 | 6.1 | 71 | conserved hypothetical protein | Sequence similarity to: YP_001504131.1| hypothetical protein EFP_gp022 [Enterococcus phage phiEF24C] & YP_001468472.1| gp92 [Listeria phage A511] |
| 144774..145631 | - | 858 | orf227 | 32598 | 4.6 | 285 | conserved hypothetical protein | Sequence similarity to: AAY53325.1| gp22 [Listeria phage P100] & YP_001468471.1| gp91 [Listeria phage A511] |
| 145655..146284 | - | 630 | orf228 | 24143 | 10.4 | 209 | conserved hypothetical protein | Sequence similarity to: YP_001468470.1| gp90 [Listeria phage A511] |
| 146284..147078 | - | 795 | orf229 | 29975 | 7.6 | 264 | conserved hypothetical protein | Sequence similarity to:YP_001468469.1| gp89 [Listeria phage A511] |
| 147092..147967 | - | 876 | orf230 | 32970 | 5.5 | 291 | conserved hypothetical protein, putative structural protein | Sequence similarity to: YP_001504127.1| hypothetical protein EFP_gp018 [Enterococcus phage phiEF24C] & YP_001468468.1| gp88 [Listeria phage A511]; Postulated structural protein by homology with Listeria phage A511 structural proteins |
| 147988..148194 | - | 207 | orf231 | 7837 | 5.9 | 68 | hypothetical protein |  |
| 148285..149727 | - | 1443 | orf232 | 52545 | 5.3 | 480 | Major capsid precursor; putative membrane protein | Sequence similarity to: YP_001468466.1| major capsid protein precursor [Listeria phage A511] & YP_001469619.1| major capsid protein [Listeria phage A511]; Motifs: one transmembrane domain shown using TMHMM and Phobius |
| 149892..150770 | - | 879 | orf233 | 33033 | 4.4 | 292 | hypothetical protein |  |
| 150790..151593 | - | 804 | orf235 | 29887 | 4.9 | 267 | phage prohead protease | Sequence similarity to: YP_001468464.1| gp84 [Listeria phage A511] & CAA62538.1| ORF1 [Listeria phage A511]; Motifs: Peptidase_U35[pfam04586], Caudovirus prohead protease & proheadase_HK97[TIGR01543], phage prohead protease, HK97 family & MEROPS Family/Subfamily = U35 |
| 151697..153373 | - | 1677 | orf236 | 62775 | 8.7 | 558 | Phage portal protein | Sequence similarity to: YP_001504121.1| putative portal protein [Enterococcus phage phiEF24C] & YP_001468463.1| gp83 [Listeria phage A511] ; Motifs: Phage_portal[pfam04860], Phage portal protein & portal_HK97[TIGR01537], phage portal protein, HK97 family |
| 153400..153699 | - | 300 | orf237 | 11660 | 6.3 | 99 | conserved hypothetical protein | Sequence similarity to: YP_001468462.1| gp82 [Listeria phage A511] |
| 153852..154691 | - | 840 | orf238 | 30650 | 6.0 | 279 | conserved hypothetical protein | Sequence similarity to: ZP_04174859.1| hypothetical protein bcere0030_25170 [Bacillus cereus AH1273] & ZP_04262340.1| hypothetical protein bcere0014_24320 [Bacillus cereus BDRD-ST196] |
| 155194..155823 | - | 630 | orf239 | 23519 | 5.9 | 209 | conserved hypothetical protein | Sequence similarity to: YP_002770778.1| hypothetical protein BBR47_12970 [Brevibacillus brevis NBRC 100599] & YP_079177.1| phage-like protein [Bacillus licheniformis ATCC 14580] |
