# Supplementary material for: Genome sequence and analysis of a broad-host range lytic bacteriophage that infects the Bacillus cereus group
Source: Virol J. 2013 Feb 7;10:48. doi: 10.1186/1743-422X-10-48 (PMC3601020; doi:10.1186/1743-422X-10-48)
Supplement: Additional file 2: Table S2 — Protein identification of phage Bc431v3 by UPLC LTQ-FT MS/MS analyses. [file 1743-422X-10-48-S2.doc]

**Additional file 2, Table S2. Protein identification of phage Bc431v3 by UPLC LTQ-FT MS/MS analyses**

**_____________________________________________________________________________________________________________________________________________**

1. [orf225|tail](http://10.139.25.109/mascot/cgi/protein_view.pl?file=../data/20120608/F008231.dat&hit=orf225|tail&db_idx=1&px=1&ave_thresh=38&_ignoreionsscorebelow=20&report=0&_sigthreshold=0.001&_msresflags=1089&_msresflags2=2&percolate=0&percolate_rt=0) sheath protein|[vB_BceM_Bc431v3] **Mass:** 61082  **Score:** 1705  **emPAI:** 11.26

Sequence coverage: **63%;** Matched peptides shown in **Bold Red**

**1** MVSYGHDRKR PHTEITLNAS GLGSANARSE KPLVLIGSAT GGQPK**VPVEL TNFAQAR**DFF RGGELLDAIE MAWNPSPNTR GAGKIYAIRA DDAKQGTK**TS**

**101 GGLTVTSKLY GADANEIQYA LDDNTLTQSK RFSVYFTKER YEQVYDNIGN IFSIKYKGAL AYGGVEVK**VD ATSK**LATQLI LKAGADKATA TVVRTY**TLGT

**201** GVYQNVN**VLI NDISNLPDFE VVTNSLGGNK NVETQFLDAL VETDVK**ATA**K MLTAIGADLV NQTDTDPYVK LSYDPKTAIP ATIPVTNL**TG GSTTAPGTSW

**301** AELFTAVADL GAYYIVPLTD KESIHGELSQ FLRDESGAGN QLRG**FVGGGL KDTFDK**LKAR QAGLRNPRVS L**VGNSGTRRM SDGRVYNYPA YMGAALIGGI**

**401 AS**GIAVGEPV TYK**KLNVEAL DIKFTGDQLD QL**DGAGVVMV EFVRTRASSY **FRIVSDPTTY** N**TASEPVQNR VSLGEVSDFL TTELRTMLDE QFIGTR**IR**NT**

**501 SASIIKNAVE SFLDNQKNVD GLIVDYNPDD VQVVITGNSA RINITVQPAR GLDDITVGIN YVDNKLTA**

**Start - End Observed Mr(expt) Mr(calc) ppm Miss Sequence**

**46 - 57 672.8671 1343.7196 1343.7197 0 0 K.VPVELTNFAQAR.D**  ([Ions score 105](http://10.139.25.109/mascot/cgi/peptide_view.pl?file=../data/20120608/F008231.dat&query=5156&hit=1&index=orf225|tail&px=1&section=5&ave_thresh=38&_ignoreionsscorebelow=20&report=0&_sigthreshold=0.001&_msresflags=1089&_msresflags2=2&percolate=0&percolate_rt=0))

**99 - 108 475.7613 949.5081 949.5080 0 0 K.TSGGLTVTSK.L**  ([Ions score 45](http://10.139.25.109/mascot/cgi/peptide_view.pl?file=../data/20120608/F008231.dat&query=892&hit=1&index=orf225|tail&px=1&section=5&ave_thresh=38&_ignoreionsscorebelow=20&report=0&_sigthreshold=0.001&_msresflags=1089&_msresflags2=2&percolate=0&percolate_rt=0))

**104 - 110 406.2317 810.4489 810.4487 0 0 L.TVTSKLY.G**  ([Ions score 30](http://10.139.25.109/mascot/cgi/peptide_view.pl?file=../data/20120608/F008231.dat&query=323&hit=1&index=orf225|tail&px=1&section=5&ave_thresh=38&_ignoreionsscorebelow=20&report=0&_sigthreshold=0.001&_msresflags=1089&_msresflags2=2&percolate=0&percolate_rt=0))

**109 - 130 815.0593 2442.1545 2442.1551 0 0 K.LYGADANEIQYALDDNTLTQSK.R** ([Ions score 45](http://10.139.25.109/mascot/cgi/peptide_view.pl?file=../data/20120608/F008231.dat&query=11338&hit=1&index=orf225|tail&px=1&section=5&ave_thresh=38&_ignoreionsscorebelow=20&report=0&_sigthreshold=0.001&_msresflags=1089&_msresflags2=2&percolate=0&percolate_rt=0))

**109 - 130 1222.0862 2442.1567 2442.1551 1 0 K.LYGADANEIQYALDDNTLTQSK.R** ([Ions score 110](http://10.139.25.109/mascot/cgi/peptide_view.pl?file=../data/20120608/F008231.dat&query=11339&hit=1&index=orf225|tail&px=1&section=5&ave_thresh=38&_ignoreionsscorebelow=20&report=0&_sigthreshold=0.001&_msresflags=1089&_msresflags2=2&percolate=0&percolate_rt=0))

**109 - 130 815.0597 2442.1556 2442.1551 0 0 K.LYGADANEIQYALDDNTLTQSK.R** ([Ions score 20](http://10.139.25.109/mascot/cgi/peptide_view.pl?file=../data/20120608/F008231.dat&query=11340&hit=1&index=orf225|tail&px=1&section=5&ave_thresh=38&_ignoreionsscorebelow=20&report=0&_sigthreshold=0.001&_msresflags=1089&_msresflags2=2&percolate=0&percolate_rt=0))

**111 - 132 824.0673 2469.1784 2469.1772 1 0 Y.GADANEIQYALDDNTLTQSKRF.S** ([Ions score 60](http://10.139.25.109/mascot/cgi/peptide_view.pl?file=../data/20120608/F008231.dat&query=11380&hit=1&index=orf225|tail&px=1&section=5&ave_thresh=38&_ignoreionsscorebelow=20&report=0&_sigthreshold=0.001&_msresflags=1089&_msresflags2=2&percolate=0&percolate_rt=0))

**111 - 132 824.0674 2470.1787 2469.1772 1 0 Y.GADANEIQYALDDNTLTQSKRF.S** ([Ions score 49](http://10.139.25.109/mascot/cgi/peptide_view.pl?file=../data/20120608/F008231.dat&query=11381&hit=1&index=orf225|tail&px=1&section=5&ave_thresh=38&_ignoreionsscorebelow=20&report=0&_sigthreshold=0.001&_msresflags=1089&_msresflags2=2&percolate=0&percolate_rt=0))

**120 - 132 754.8886 1507.7627 1507.7631 0 0 Y.ALDDNTLTQSKRF.S**  ([Ions score 61](http://10.139.25.109/mascot/cgi/peptide_view.pl?file=../data/20120608/F008231.dat&query=6331&hit=1&index=orf225|tail&px=1&section=5&ave_thresh=38&_ignoreionsscorebelow=20&report=0&_sigthreshold=0.001&_msresflags=1089&_msresflags2=2&percolate=0&percolate_rt=0))

**120 - 132 754.8922 1507.7699 1507.7631 5 0 Y.ALDDNTLTQSKRF.S**  ([Ions score 67](http://10.139.25.109/mascot/cgi/peptide_view.pl?file=../data/20120608/F008231.dat&query=6333&hit=1&index=orf225|tail&px=1&section=5&ave_thresh=38&_ignoreionsscorebelow=20&report=0&_sigthreshold=0.001&_msresflags=1089&_msresflags2=2&percolate=0&percolate_rt=0))

**131 - 138 524.2845 1046.5544 1046.5549 0 0 K.RFSVYFTK.E**  ([Ions score 30](http://10.139.25.109/mascot/cgi/peptide_view.pl?file=../data/20120608/F008231.dat&query=1650&hit=1&index=orf225|tail&px=1&section=5&ave_thresh=38&_ignoreionsscorebelow=20&report=0&_sigthreshold=0.001&_msresflags=1089&_msresflags2=2&percolate=0&percolate_rt=0))

**131 - 138 524.2851 1046.5556 1046.5549 1 0 K.RFSVYFTK.E**  ([Ions score 27](http://10.139.25.109/mascot/cgi/peptide_view.pl?file=../data/20120608/F008231.dat&query=1651&hit=1&index=orf225|tail&px=1&section=5&ave_thresh=38&_ignoreionsscorebelow=20&report=0&_sigthreshold=0.001&_msresflags=1089&_msresflags2=2&percolate=0&percolate_rt=0))

**132 - 138 446.2338 890.4530 890.4538 -1 0 R.FSVYFTK.E**  ([Ions score 43](http://10.139.25.109/mascot/cgi/peptide_view.pl?file=../data/20120608/F008231.dat&query=659&hit=1&index=orf225|tail&px=1&section=5&ave_thresh=38&_ignoreionsscorebelow=20&report=0&_sigthreshold=0.001&_msresflags=1089&_msresflags2=2&percolate=0&percolate_rt=0))

**136 - 152 712.6855 2135.0330 2135.0324 0 0 Y.FTKERYEQVYDNIGNIF.S**  ([Ions score 42](http://10.139.25.109/mascot/cgi/peptide_view.pl?file=../data/20120608/F008231.dat&query=10364&hit=1&index=orf225|tail&px=1&section=5&ave_thresh=38&_ignoreionsscorebelow=20&report=0&_sigthreshold=0.001&_msresflags=1089&_msresflags2=2&percolate=0&percolate_rt=0))

**136 - 152 713.6855 2135.0330 2135.0324 0 0 Y.FTKERYEQVYDNIGNIF.S**  ([Ions score 45](http://10.139.25.109/mascot/cgi/peptide_view.pl?file=../data/20120608/F008231.dat&query=10365&hit=1&index=orf225|tail&px=1&section=5&ave_thresh=38&_ignoreionsscorebelow=20&report=0&_sigthreshold=0.001&_msresflags=1089&_msresflags2=2&percolate=0&percolate_rt=0))

**137 - 152 663.6619 1987.9638 1987.9639 0 0 F.TKERYEQVYDNIGNIF.S**  ([Ions score 36](http://10.139.25.109/mascot/cgi/peptide_view.pl?file=../data/20120608/F008231.dat&query=9473&hit=1&index=orf225|tail&px=1&section=5&ave_thresh=38&_ignoreionsscorebelow=20&report=0&_sigthreshold=0.001&_msresflags=1089&_msresflags2=2&percolate=0&percolate_rt=0))

**139 - 155 696.6868 2087.0385 2087.0323 3 0 K.ERYEQVYDNIGNIFSIK.Y**  ([Ions score 27](http://10.139.25.109/mascot/cgi/peptide_view.pl?file=../data/20120608/F008231.dat&query=10086&hit=1&index=orf225|tail&px=1&section=5&ave_thresh=38&_ignoreionsscorebelow=20&report=0&_sigthreshold=0.001&_msresflags=1089&_msresflags2=2&percolate=0&percolate_rt=0))

**139 - 155 696.6855 2087.0330 2088.0323 0 0 K.ERYEQVYDNIGNIFSIK.Y**  ([Ions score 61](http://10.139.25.109/mascot/cgi/peptide_view.pl?file=../data/20120608/F008231.dat&query=10098&hit=1&index=orf225|tail&px=1&section=5&ave_thresh=38&_ignoreionsscorebelow=20&report=0&_sigthreshold=0.001&_msresflags=1089&_msresflags2=2&percolate=0&percolate_rt=0))

**141 - 155 901.9502 1801.8858 1801.8886 -2 0 R.YEQVYDNIGNIFSIK.Y**  ([Ions score 88](http://10.139.25.109/mascot/cgi/peptide_view.pl?file=../data/20120608/F008231.dat&query=8528&hit=1&index=orf225|tail&px=1&section=5&ave_thresh=38&_ignoreionsscorebelow=20&report=0&_sigthreshold=0.001&_msresflags=1089&_msresflags2=2&percolate=0&percolate_rt=0))

**142 - 152 656.3149 1310.6153 1310.6143 1 0 Y.EQVYDNIGNIF.S**  ([Ions score 54](http://10.139.25.109/mascot/cgi/peptide_view.pl?file=../data/20120608/F008231.dat&query=4834&hit=1&index=orf225|tail&px=1&section=5&ave_thresh=38&_ignoreionsscorebelow=20&report=0&_sigthreshold=0.001&_msresflags=1089&_msresflags2=2&percolate=0&percolate_rt=0))

**142 - 152 656.3154 1310.6163 1310.6143 2 0 Y.EQVYDNIGNIF.S**  ([Ions score 30](http://10.139.25.109/mascot/cgi/peptide_view.pl?file=../data/20120608/F008231.dat&query=4835&hit=1&index=orf225|tail&px=1&section=5&ave_thresh=38&_ignoreionsscorebelow=20&report=0&_sigthreshold=0.001&_msresflags=1089&_msresflags2=2&percolate=0&percolate_rt=0))

**142 - 155 820.4211 1638.8276 1638.8253 1 0 Y.EQVYDNIGNIFSIK.Y**  ([Ions score 76](http://10.139.25.109/mascot/cgi/peptide_view.pl?file=../data/20120608/F008231.dat&query=7375&hit=1&index=orf225|tail&px=1&section=5&ave_thresh=38&_ignoreionsscorebelow=20&report=0&_sigthreshold=0.001&_msresflags=1089&_msresflags2=2&percolate=0&percolate_rt=0))

**142 - 155 820.4236 1638.8327 1638.8253 5 0 Y.EQVYDNIGNIFSIK.Y**  ([Ions score 68](http://10.139.25.109/mascot/cgi/peptide_view.pl?file=../data/20120608/F008231.dat&query=7376&hit=1&index=orf225|tail&px=1&section=5&ave_thresh=38&_ignoreionsscorebelow=20&report=0&_sigthreshold=0.001&_msresflags=1089&_msresflags2=2&percolate=0&percolate_rt=0))

**143 - 155 755.8957 1509.7768 1509.7827 -4 0 E.QVYDNIGNIFSIK.Y**  ([Ions score 60](http://10.139.25.109/mascot/cgi/peptide_view.pl?file=../data/20120608/F008231.dat&query=6343&hit=1&index=orf225|tail&px=1&section=5&ave_thresh=38&_ignoreionsscorebelow=20&report=0&_sigthreshold=0.001&_msresflags=1089&_msresflags2=2&percolate=0&percolate_rt=0))

**143 - 155 755.8998 1509.7850 1509.7827 2 0 E.QVYDNIGNIFSIK.Y**  ([Ions score 58](http://10.139.25.109/mascot/cgi/peptide_view.pl?file=../data/20120608/F008231.dat&query=6344&hit=1&index=orf225|tail&px=1&section=5&ave_thresh=38&_ignoreionsscorebelow=20&report=0&_sigthreshold=0.001&_msresflags=1089&_msresflags2=2&percolate=0&percolate_rt=0))

**144 - 152 527.7642 1053.5138 1053.5131 1 0 Q.VYDNIGNIF.S**  ([Ions score 34](http://10.139.25.109/mascot/cgi/peptide_view.pl?file=../data/20120608/F008231.dat&query=1721&hit=1&index=orf225|tail&px=1&section=5&ave_thresh=38&_ignoreionsscorebelow=20&report=0&_sigthreshold=0.001&_msresflags=1089&_msresflags2=2&percolate=0&percolate_rt=0))

**144 - 152 527.7643 1053.5140 1053.5131 1 0 Q.VYDNIGNIF.S**  ([Ions score 24](http://10.139.25.109/mascot/cgi/peptide_view.pl?file=../data/20120608/F008231.dat&query=1722&hit=1&index=orf225|tail&px=1&section=5&ave_thresh=38&_ignoreionsscorebelow=20&report=0&_sigthreshold=0.001&_msresflags=1089&_msresflags2=2&percolate=0&percolate_rt=0))

**144 - 155 691.8696 1381.7246 1381.7242 0 0 Q.VYDNIGNIFSIK.Y**  ([Ions score 58](http://10.139.25.109/mascot/cgi/peptide_view.pl?file=../data/20120608/F008231.dat&query=5448&hit=1&index=orf225|tail&px=1&section=5&ave_thresh=38&_ignoreionsscorebelow=20&report=0&_sigthreshold=0.001&_msresflags=1089&_msresflags2=2&percolate=0&percolate_rt=0))

**144 - 155 691.8705 1381.7265 1381.7242 2 0 Q.VYDNIGNIFSIK.Y**  ([Ions score 45](http://10.139.25.109/mascot/cgi/peptide_view.pl?file=../data/20120608/F008231.dat&query=5449&hit=1&index=orf225|tail&px=1&section=5&ave_thresh=38&_ignoreionsscorebelow=20&report=0&_sigthreshold=0.001&_msresflags=1089&_msresflags2=2&percolate=0&percolate_rt=0))

**156 - 168 677.8709 1353.7271 1353.7292 -2 0 K.YKGALAYGGVEVK.V**  ([Ions score 50](http://10.139.25.109/mascot/cgi/peptide_view.pl?file=../data/20120608/F008231.dat&query=5210&hit=1&index=orf225|tail&px=1&section=5&ave_thresh=38&_ignoreionsscorebelow=20&report=0&_sigthreshold=0.001&_msresflags=1089&_msresflags2=2&percolate=0&percolate_rt=0))

**156 - 168 452.2506 1353.7300 1353.7292 1 0 K.YKGALAYGGVEVK.V**  ([Ions score 51](http://10.139.25.109/mascot/cgi/peptide_view.pl?file=../data/20120608/F008231.dat&query=5211&hit=1&index=orf225|tail&px=1&section=5&ave_thresh=38&_ignoreionsscorebelow=20&report=0&_sigthreshold=0.001&_msresflags=1089&_msresflags2=2&percolate=0&percolate_rt=0))

**158 - 168 532.2931 1062.5716 1062.5710 1 0 K.GALAYGGVEVK.V**  ([Ions score 44](http://10.139.25.109/mascot/cgi/peptide_view.pl?file=../data/20120608/F008231.dat&query=1812&hit=1&index=orf225|tail&px=1&section=5&ave_thresh=38&_ignoreionsscorebelow=20&report=0&_sigthreshold=0.001&_msresflags=1089&_msresflags2=2&percolate=0&percolate_rt=0))

**175 - 182 450.2998 898.5851 898.5851 0 0 K.LATQLILK.A**  ([Ions score 59](http://10.139.25.109/mascot/cgi/peptide_view.pl?file=../data/20120608/F008231.dat&query=696&hit=1&index=orf225|tail&px=1&section=5&ave_thresh=38&_ignoreionsscorebelow=20&report=0&_sigthreshold=0.001&_msresflags=1089&_msresflags2=2&percolate=0&percolate_rt=0))

**180 - 196 445.2601 1777.0112 1777.0098 1 0 L.ILKAGADKATATVVRTY.T**  ([Ions score 35](http://10.139.25.109/mascot/cgi/peptide_view.pl?file=../data/20120608/F008231.dat&query=8361&hit=1&index=orf225|tail&px=1&section=5&ave_thresh=38&_ignoreionsscorebelow=20&report=0&_sigthreshold=0.001&_msresflags=1089&_msresflags2=2&percolate=0&percolate_rt=0))

**182 - 196 517.9545 1550.8416 1550.8416 0 0 L.KAGADKATATVVRTY.T**  ([Ions score 42](http://10.139.25.109/mascot/cgi/peptide_view.pl?file=../data/20120608/F008231.dat&query=6690&hit=1&index=orf225|tail&px=1&section=5&ave_thresh=38&_ignoreionsscorebelow=20&report=0&_sigthreshold=0.001&_msresflags=1089&_msresflags2=2&percolate=0&percolate_rt=0))

**182 - 196 517.9547 1550.8421 1550.8416 0 0 L.KAGADKATATVVRTY.T**  ([Ions score 34](http://10.139.25.109/mascot/cgi/peptide_view.pl?file=../data/20120608/F008231.dat&query=6691&hit=1&index=orf225|tail&px=1&section=5&ave_thresh=38&_ignoreionsscorebelow=20&report=0&_sigthreshold=0.001&_msresflags=1089&_msresflags2=2&percolate=0&percolate_rt=0))

**188 - 194 359.2169 716.4192 716.4181 2 0 K.ATATVVR.T**  ([Ions score 46](http://10.139.25.109/mascot/cgi/peptide_view.pl?file=../data/20120608/F008231.dat&query=29&hit=1&index=orf225|tail&px=1&section=5&ave_thresh=38&_ignoreionsscorebelow=20&report=0&_sigthreshold=0.001&_msresflags=1089&_msresflags2=2&percolate=0&percolate_rt=0))

**191 - 196 369.7107 737.4069 737.4072 0 0 A.TVVRTY.T**  ([Ions score 21](http://10.139.25.109/mascot/cgi/peptide_view.pl?file=../data/20120608/F008231.dat&query=83&hit=1&index=orf225|tail&px=1&section=5&ave_thresh=38&_ignoreionsscorebelow=20&report=0&_sigthreshold=0.001&_msresflags=1089&_msresflags2=2&percolate=0&percolate_rt=0))

**208 - 224 951.4963 1900.9770 1900.9782 -1 0 N.VLINDISNLPDFEVVTN.S** ([Ions score 23](http://10.139.25.109/mascot/cgi/peptide_view.pl?file=../data/20120608/F008231.dat&query=9096&hit=1&index=orf225|tail&px=1&section=5&ave_thresh=38&_ignoreionsscorebelow=20&report=0&_sigthreshold=0.001&_msresflags=1089&_msresflags2=2&percolate=0&percolate_rt=0))

**224 - 234 566.7807 1131.5458 1131.5520 -6 0 T.NSLGGNKNVET.Q**  ([Ions score 21](http://10.139.25.109/mascot/cgi/peptide_view.pl?file=../data/20120608/F008231.dat&query=2652&hit=1&index=orf225|tail&px=1&section=5&ave_thresh=38&_ignoreionsscorebelow=20&report=0&_sigthreshold=0.001&_msresflags=1089&_msresflags2=2&percolate=0&percolate_rt=0))

**224 - 236 704.3406 1406.6666 1406.6790 -9 0 T.NSLGGNKNVETQF.L**  ([Ions score 24](http://10.139.25.109/mascot/cgi/peptide_view.pl?file=../data/20120608/F008231.dat&query=5625&hit=1&index=orf225|tail&px=1&section=5&ave_thresh=38&_ignoreionsscorebelow=20&report=0&_sigthreshold=0.001&_msresflags=1089&_msresflags2=2&percolate=0&percolate_rt=0))

**225 - 237 704.3418 1407.6768 1407.6882 -8 0 N.SLGGNKNVETQFL.D**  2 Deamidated (NQ) ([Ions score 20](http://10.139.25.109/mascot/cgi/peptide_view.pl?file=../data/20120608/F008231.dat&query=5629&hit=1&index=orf225|tail&px=1&section=5&ave_thresh=38&_ignoreionsscorebelow=20&report=0&_sigthreshold=0.001&_msresflags=1089&_msresflags2=2&percolate=0&percolate_rt=0))

**231 - 246 910.9693 1819.9240 1819.9204 2 0 K.NVETQFLDALVETDVK.A**  ([Ions score 110](http://10.139.25.109/mascot/cgi/peptide_view.pl?file=../data/20120608/F008231.dat&query=8637&hit=1&index=orf225|tail&px=1&section=5&ave_thresh=38&_ignoreionsscorebelow=20&report=0&_sigthreshold=0.001&_msresflags=1089&_msresflags2=2&percolate=0&percolate_rt=0))

**250 - 266 903.4580 1804.9015 1804.8877 8 0 A.KMLTAIGADLVNQTDTD.P**  ([Ions score 20](http://10.139.25.109/mascot/cgi/peptide_view.pl?file=../data/20120608/F008231.dat&query=8559&hit=1&index=orf225|tail&px=1&section=5&ave_thresh=38&_ignoreionsscorebelow=20&report=0&_sigthreshold=0.001&_msresflags=1089&_msresflags2=2&percolate=0&percolate_rt=0))

**251 - 270 722.3654 2164.0744 2164.0722 1 0 K.MLTAIGADLVNQTDTDPYVK.L**  ([Ions score 58](http://10.139.25.109/mascot/cgi/peptide_view.pl?file=../data/20120608/F008231.dat&query=10478&hit=1&index=orf225|tail&px=1&section=5&ave_thresh=38&_ignoreionsscorebelow=20&report=0&_sigthreshold=0.001&_msresflags=1089&_msresflags2=2&percolate=0&percolate_rt=0))

**251 - 270 727.6969 2180.0689 2180.0671 1 0 K.MLTAIGADLVNQTDTDPYVK.L**  Oxidation (M) ([Ions score 21](http://10.139.25.109/mascot/cgi/peptide_view.pl?file=../data/20120608/F008231.dat&query=10564&hit=1&index=orf225|tail&px=1&section=5&ave_thresh=38&_ignoreionsscorebelow=20&report=0&_sigthreshold=0.001&_msresflags=1089&_msresflags2=2&percolate=0&percolate_rt=0))

**251 - 276 956.8165 2867.4260 2867.4263 0 0 K.MLTAIGADLVNQTDTDPYVKLSYDPK.T** [Ions score74](http://10.139.25.109/mascot/cgi/peptide_view.pl?file=../data/20120608/F008231.dat&query=11877&hit=1&index=orf225|tail&px=1&section=5&ave_thresh=38&_ignoreionsscorebelow=20&report=0&_sigthreshold=0.001&_msresflags=1089&_msresflags2=2&percolate=0&percolate_rt=0))

**251 - 276 956.8170 2867.4275 2867.4263 0 0 K.MLTAIGADLVNQTDTDPYVKLSYDPK.T** [Ions score98](http://10.139.25.109/mascot/cgi/peptide_view.pl?file=../data/20120608/F008231.dat&query=11878&hit=1&index=orf225|tail&px=1&section=5&ave_thresh=38&_ignoreionsscorebelow=20&report=0&_sigthreshold=0.001&_msresflags=1089&_msresflags2=2&percolate=0&percolate_rt=0))

**252 - 270 1017.5277 2033.0408 2033.0317 4 0 M.LTAIGADLVNQTDTDPYVK.L**  ([Ions score 59](http://10.139.25.109/mascot/cgi/peptide_view.pl?file=../data/20120608/F008231.dat&query=9751&hit=1&index=orf225|tail&px=1&section=5&ave_thresh=38&_ignoreionsscorebelow=20&report=0&_sigthreshold=0.001&_msresflags=1089&_msresflags2=2&percolate=0&percolate_rt=0))

**252 - 270 1017.5319 2033.0492 2033.0317 3 0 M.LTAIGADLVNQTDTDPYVK.L**  ([Ions score 45](http://10.139.25.109/mascot/cgi/peptide_view.pl?file=../data/20120608/F008231.dat&query=9752&hit=1&index=orf225|tail&px=1&section=5&ave_thresh=38&_ignoreionsscorebelow=20&report=0&_sigthreshold=0.001&_msresflags=1089&_msresflags2=2&percolate=0&percolate_rt=0))

**253 - 268 847.4022 1692.7898 1692.7843 3 0 L.TAIGADLVNQTDTDPY.V**  ([Ions score 35](http://10.139.25.109/mascot/cgi/peptide_view.pl?file=../data/20120608/F008231.dat&query=7837&hit=1&index=orf225|tail&px=1&section=5&ave_thresh=38&_ignoreionsscorebelow=20&report=0&_sigthreshold=0.001&_msresflags=1089&_msresflags2=2&percolate=0&percolate_rt=0))

**253 - 268 847.4032 1692.7918 1692.7843 4 0 L.TAIGADLVNQTDTDPY.V**  ([Ions score 25](http://10.139.25.109/mascot/cgi/peptide_view.pl?file=../data/20120608/F008231.dat&query=7838&hit=1&index=orf225|tail&px=1&section=5&ave_thresh=38&_ignoreionsscorebelow=20&report=0&_sigthreshold=0.001&_msresflags=1089&_msresflags2=2&percolate=0&percolate_rt=0))

**253 - 270 960.9818 1919.9491 1919.9476 1 0 L.TAIGADLVNQTDTDPYVK.L**  ([Ions score 105](http://10.139.25.109/mascot/cgi/peptide_view.pl?file=../data/20120608/F008231.dat&query=9208&hit=1&index=orf225|tail&px=1&section=5&ave_thresh=38&_ignoreionsscorebelow=20&report=0&_sigthreshold=0.001&_msresflags=1089&_msresflags2=2&percolate=0&percolate_rt=0))

**253 - 270 960.9825 1919.9493 1919.9476 1 0 L.TAIGADLVNQTDTDPYVK.L**  ([Ions score 72](http://10.139.25.109/mascot/cgi/peptide_view.pl?file=../data/20120608/F008231.dat&query=9217&hit=1&index=orf225|tail&px=1&section=5&ave_thresh=38&_ignoreionsscorebelow=20&report=0&_sigthreshold=0.001&_msresflags=1089&_msresflags2=2&percolate=0&percolate_rt=0))

**253 - 271 678.6838 2033.0297 2033.0317 -1 0 L.TAIGADLVNQTDTDPYVKL.S**  ([Ions score 43](http://10.139.25.109/mascot/cgi/peptide_view.pl?file=../data/20120608/F008231.dat&query=9750&hit=1&index=orf225|tail&px=1&section=5&ave_thresh=38&_ignoreionsscorebelow=20&report=0&_sigthreshold=0.001&_msresflags=1089&_msresflags2=2&percolate=0&percolate_rt=0))

**253 - 271 1017.5243 2033.0329 2033.0317 1 0 L.TAIGADLVNQTDTDPYVKL.S**  Deamidated (NQ) ([Ions score 25](http://10.139.25.109/mascot/cgi/peptide_view.pl?file=../data/20120608/F008231.dat&query=9764&hit=1&index=orf225|tail&px=1&section=5&ave_thresh=38&_ignoreionsscorebelow=20&report=0&_sigthreshold=0.001&_msresflags=1089&_msresflags2=2&percolate=0&percolate_rt=0))

**269 - 287 676.7171 2027.1278 2027.1303 1 0 Y.VKLSYDPKTAIPATIPVTN.L**  Deamidated (NQ) ([Ions score 25](http://10.139.25.109/mascot/cgi/peptide_view.pl?file=../data/20120608/F008231.dat&query=9722&hit=1&index=orf225|tail&px=1&section=5&ave_thresh=38&_ignoreionsscorebelow=20&report=0&_sigthreshold=0.001&_msresflags=1089&_msresflags2=2&percolate=0&percolate_rt=0))

**274 - 288 775.9423 1549.8701 1549.8716 -1 0 Y.DPKTAIPATIPVTNL.T**  ([Ions score 27](http://10.139.25.109/mascot/cgi/peptide_view.pl?file=../data/20120608/F008231.dat&query=6675&hit=1&index=orf225|tail&px=1&section=5&ave_thresh=38&_ignoreionsscorebelow=20&report=0&_sigthreshold=0.001&_msresflags=1089&_msresflags2=2&percolate=0&percolate_rt=0))

**345 - 356 642.3386 1282.6626 1282.6558 5 0 G.FVGGGLKDTFDK.L**  ([Ions score 45](http://10.139.25.109/mascot/cgi/peptide_view.pl?file=../data/20120608/F008231.dat&query=4522&hit=1&index=orf225|tail&px=1&section=5&ave_thresh=38&_ignoreionsscorebelow=20&report=0&_sigthreshold=0.001&_msresflags=1089&_msresflags2=2&percolate=0&percolate_rt=0))

**372 - 402 1079.5271 3235.5595 3235.5502 3 0 L.VGNSGTRRMSDGRVYNYPAYMGAALIGGIAS.G**  ([Ions score 22](http://10.139.25.109/mascot/cgi/peptide_view.pl?file=../data/20120608/F008231.dat&query=12092&hit=1&index=orf225|tail&px=1&section=5&ave_thresh=38&_ignoreionsscorebelow=20&report=0&_sigthreshold=0.001&_msresflags=1089&_msresflags2=2&percolate=0&percolate_rt=0))

**414 - 423 571.8421 1141.6696 1141.6706 -1 0 K.KLNVEALDIK.F**  ([Ions score 50](http://10.139.25.109/mascot/cgi/peptide_view.pl?file=../data/20120608/F008231.dat&query=2784&hit=1&index=orf225|tail&px=1&section=5&ave_thresh=38&_ignoreionsscorebelow=20&report=0&_sigthreshold=0.001&_msresflags=1089&_msresflags2=2&percolate=0&percolate_rt=0))

**421 - 432 697.8403 1393.6661 1393.6613 3 0 L.DIKFTGDQLDQL.D**  2 Deamidated (NQ) ([Ions score 22](http://10.139.25.109/mascot/cgi/peptide_view.pl?file=../data/20120608/F008231.dat&query=5542&hit=3&index=orf225|tail&px=1&section=5&ave_thresh=38&_ignoreionsscorebelow=20&report=0&_sigthreshold=0.001&_msresflags=1089&_msresflags2=2&percolate=0&percolate_rt=0))

**451 - 460 599.8089 1197.6032 1197.6030 0 0 Y.FRIVSDPTTY.N**  ([Ions score 31](http://10.139.25.109/mascot/cgi/peptide_view.pl?file=../data/20120608/F008231.dat&query=3536&hit=1&index=orf225|tail&px=1&section=5&ave_thresh=38&_ignoreionsscorebelow=20&report=0&_sigthreshold=0.001&_msresflags=1089&_msresflags2=2&percolate=0&percolate_rt=0))

**451 - 460 599.8104 1197.6062 1197.6030 3 0 Y.FRIVSDPTTY.N**  ([Ions score 26](http://10.139.25.109/mascot/cgi/peptide_view.pl?file=../data/20120608/F008231.dat&query=3537&hit=1&index=orf225|tail&px=1&section=5&ave_thresh=38&_ignoreionsscorebelow=20&report=0&_sigthreshold=0.001&_msresflags=1089&_msresflags2=2&percolate=0&percolate_rt=0))

**462 - 480 1024.5204 2047.0262 2047.0222 2 0 N.TASEPVQNRVSLGEVSDFL.T**  ([Ions score 48](http://10.139.25.109/mascot/cgi/peptide_view.pl?file=../data/20120608/F008231.dat&query=9862&hit=1&index=orf225|tail&px=1&section=5&ave_thresh=38&_ignoreionsscorebelow=20&report=0&_sigthreshold=0.001&_msresflags=1089&_msresflags2=2&percolate=0&percolate_rt=0))

**463 - 480 973.9959 1945.9771 1945.9745 1 0 T.ASEPVQNRVSLGEVSDFL.T**  ([Ions score 52](http://10.139.25.109/mascot/cgi/peptide_view.pl?file=../data/20120608/F008231.dat&query=9313&hit=1&index=orf225|tail&px=1&section=5&ave_thresh=38&_ignoreionsscorebelow=20&report=0&_sigthreshold=0.001&_msresflags=1089&_msresflags2=2&percolate=0&percolate_rt=0))

**467 - 480 781.9131 1561.8117 1561.8100 1 0 P.VQNRVSLGEVSDFL.T**  ([Ions score 59](http://10.139.25.109/mascot/cgi/peptide_view.pl?file=../data/20120608/F008231.dat&query=6775&hit=1&index=orf225|tail&px=1&section=5&ave_thresh=38&_ignoreionsscorebelow=20&report=0&_sigthreshold=0.001&_msresflags=1089&_msresflags2=2&percolate=0&percolate_rt=0))

**471 - 485 833.4405 1664.8664 1664.8621 3 0 R.VSLGEVSDFLTTELR.T**  ([Ions score 104](http://10.139.25.109/mascot/cgi/peptide_view.pl?file=../data/20120608/F008231.dat&query=7598&hit=1&index=orf225|tail&px=1&section=5&ave_thresh=38&_ignoreionsscorebelow=20&report=0&_sigthreshold=0.001&_msresflags=1089&_msresflags2=2&percolate=0&percolate_rt=0))

**471 - 485 833.4407 1664.8668 1664.8621 3 0 R.VSLGEVSDFLTTELR.T**  ([Ions score 124](http://10.139.25.109/mascot/cgi/peptide_view.pl?file=../data/20120608/F008231.dat&query=7599&hit=1&index=orf225|tail&px=1&section=5&ave_thresh=38&_ignoreionsscorebelow=20&report=0&_sigthreshold=0.001&_msresflags=1089&_msresflags2=2&percolate=0&percolate_rt=0))

**473 - 485 740.3890 1478.7634 1478.7617 1 0 S.LGEVSDFLTTELR.T**  ([Ions score 90](http://10.139.25.109/mascot/cgi/peptide_view.pl?file=../data/20120608/F008231.dat&query=6124&hit=1&index=orf225|tail&px=1&section=5&ave_thresh=38&_ignoreionsscorebelow=20&report=0&_sigthreshold=0.001&_msresflags=1089&_msresflags2=2&percolate=0&percolate_rt=0))

**473 - 485 740.3890 1478.7634 1478.7617 1 0 S.LGEVSDFLTTELR.T**  ([Ions score 90](http://10.139.25.109/mascot/cgi/peptide_view.pl?file=../data/20120608/F008231.dat&query=6127&hit=1&index=orf225|tail&px=1&section=5&ave_thresh=38&_ignoreionsscorebelow=20&report=0&_sigthreshold=0.001&_msresflags=1089&_msresflags2=2&percolate=0&percolate_rt=0))

**481 - 487 426.2188 850.4230 850.4218 1 0 L.TTELRTM.L**  ([Ions score 21](http://10.139.25.109/mascot/cgi/peptide_view.pl?file=../data/20120608/F008231.dat&query=505&hit=1&index=orf225|tail&px=1&section=5&ave_thresh=38&_ignoreionsscorebelow=20&report=0&_sigthreshold=0.001&_msresflags=1089&_msresflags2=2&percolate=0&percolate_rt=0))

**481 - 492 742.3589 1482.7032 1482.7024 1 0 L.TTELRTMLDEQF.I**  ([Ions score 76](http://10.139.25.109/mascot/cgi/peptide_view.pl?file=../data/20120608/F008231.dat&query=6145&hit=1&index=orf225|tail&px=1&section=5&ave_thresh=38&_ignoreionsscorebelow=20&report=0&_sigthreshold=0.001&_msresflags=1089&_msresflags2=2&percolate=0&percolate_rt=0))

**481 - 492 742.3594 1482.7042 1482.7024 1 0 L.TTELRTMLDEQF.I**  ([Ions score 55](http://10.139.25.109/mascot/cgi/peptide_view.pl?file=../data/20120608/F008231.dat&query=6146&hit=1&index=orf225|tail&px=1&section=5&ave_thresh=38&_ignoreionsscorebelow=20&report=0&_sigthreshold=0.001&_msresflags=1089&_msresflags2=2&percolate=0&percolate_rt=0))

**485 - 492 520.2473 1038.4801 1038.4804 0 0 L.RTMLDEQF.I**  ([Ions score 43](http://10.139.25.109/mascot/cgi/peptide_view.pl?file=../data/20120608/F008231.dat&query=1561&hit=1&index=orf225|tail&px=1&section=5&ave_thresh=38&_ignoreionsscorebelow=20&report=0&_sigthreshold=0.001&_msresflags=1089&_msresflags2=2&percolate=0&percolate_rt=0))

**485 - 492 520.2476 1038.4806 1038.4804 0 0 L.RTMLDEQF.I**  ([Ions score 36](http://10.139.25.109/mascot/cgi/peptide_view.pl?file=../data/20120608/F008231.dat&query=1562&hit=1&index=orf225|tail&px=1&section=5&ave_thresh=38&_ignoreionsscorebelow=20&report=0&_sigthreshold=0.001&_msresflags=1089&_msresflags2=2&percolate=0&percolate_rt=0))

**486 - 496 655.8247 1309.6349 1309.6336 1 0 R.TMLDEQFIGTR.I**  ([Ions score 67](http://10.139.25.109/mascot/cgi/peptide_view.pl?file=../data/20120608/F008231.dat&query=4825&hit=1&index=orf225|tail&px=1&section=5&ave_thresh=38&_ignoreionsscorebelow=20&report=0&_sigthreshold=0.001&_msresflags=1089&_msresflags2=2&percolate=0&percolate_rt=0))

**486 - 496 663.8206 1325.6267 1325.6286 -1 0 R.TMLDEQFIGTR.I**  Oxidation (M) ([Ions score 68](http://10.139.25.109/mascot/cgi/peptide_view.pl?file=../data/20120608/F008231.dat&query=4991&hit=1&index=orf225|tail&px=1&section=5&ave_thresh=38&_ignoreionsscorebelow=20&report=0&_sigthreshold=0.001&_msresflags=1089&_msresflags2=2&percolate=0&percolate_rt=0))

**486 - 496 663.8222 1325.6298 1325.6286 1 0 R.TMLDEQFIGTR.I**  Oxidation (M) ([Ions score 82](http://10.139.25.109/mascot/cgi/peptide_view.pl?file=../data/20120608/F008231.dat&query=4992&hit=1&index=orf225|tail&px=1&section=5&ave_thresh=38&_ignoreionsscorebelow=20&report=0&_sigthreshold=0.001&_msresflags=1089&_msresflags2=2&percolate=0&percolate_rt=0))

**499 - 506 417.2404 832.4662 832.4654 1 0 R.NTSASIIK.N**  ([Ions score 39](http://10.139.25.109/mascot/cgi/peptide_view.pl?file=../data/20120608/F008231.dat&query=429&hit=1&index=orf225|tail&px=1&section=5&ave_thresh=38&_ignoreionsscorebelow=20&report=0&_sigthreshold=0.001&_msresflags=1089&_msresflags2=2&percolate=0&percolate_rt=0))

**500 - 512 683.8644 1365.7142 1365.7140 0 0 N.TSASIIKNAVESF.L**  ([Ions score 45](http://10.139.25.109/mascot/cgi/peptide_view.pl?file=../data/20120608/F008231.dat&query=5314&hit=1&index=orf225|tail&px=1&section=5&ave_thresh=38&_ignoreionsscorebelow=20&report=0&_sigthreshold=0.001&_msresflags=1089&_msresflags2=2&percolate=0&percolate_rt=0))

**500 - 512 683.8646 1365.7146 1365.7140 0 0 N.TSASIIKNAVESF.L**  ([Ions score 59](http://10.139.25.109/mascot/cgi/peptide_view.pl?file=../data/20120608/F008231.dat&query=5315&hit=1&index=orf225|tail&px=1&section=5&ave_thresh=38&_ignoreionsscorebelow=20&report=0&_sigthreshold=0.001&_msresflags=1089&_msresflags2=2&percolate=0&percolate_rt=0))

**507 - 517 632.8133 1263.6120 1263.6095 2 0 K.NAVESFLDNQK.N**  ([Ions score 68](http://10.139.25.109/mascot/cgi/peptide_view.pl?file=../data/20120608/F008231.dat&query=4284&hit=1&index=orf225|tail&px=1&section=5&ave_thresh=38&_ignoreionsscorebelow=20&report=0&_sigthreshold=0.001&_msresflags=1089&_msresflags2=2&percolate=0&percolate_rt=0))

**507 - 517 632.8140 1263.6134 1263.6095 3 0 K.NAVESFLDNQK.N**  ([Ions score 61](http://10.139.25.109/mascot/cgi/peptide_view.pl?file=../data/20120608/F008231.dat&query=4286&hit=1&index=orf225|tail&px=1&section=5&ave_thresh=38&_ignoreionsscorebelow=20&report=0&_sigthreshold=0.001&_msresflags=1089&_msresflags2=2&percolate=0&percolate_rt=0))

**518 - 541 858.4332 2572.2761 2572.2769 0 0 K.NVDGLIVDYNPDDVQVVITGNSAR.I** [Ions score 89](http://10.139.25.109/mascot/cgi/peptide_view.pl?file=../data/20120608/F008231.dat&query=11547&hit=1&index=orf225|tail&px=1&section=5&ave_thresh=38&_ignoreionsscorebelow=20&report=0&_sigthreshold=0.001&_msresflags=1089&_msresflags2=2&percolate=0&percolate_rt=0))

**518 - 541 1287.1467 2572.2778 2572.2769 0 0 K.NVDGLIVDYNPDDVQVVITGNSAR.I** [Ions score 133](http://10.139.25.109/mascot/cgi/peptide_view.pl?file=../data/20120608/F008231.dat&query=11548&hit=1&index=orf225|tail&px=1&section=5&ave_thresh=38&_ignoreionsscorebelow=20&report=0&_sigthreshold=0.001&_msresflags=1089&_msresflags2=2&percolate=0&percolate_rt=0))

**542 - 550 506.3008 1010.5871 1010.5873 0 0 R.INITVQPAR.G**  ([Ions score 54](http://10.139.25.109/mascot/cgi/peptide_view.pl?file=../data/20120608/F008231.dat&query=1264&hit=1&index=orf225|tail&px=1&section=5&ave_thresh=38&_ignoreionsscorebelow=20&report=0&_sigthreshold=0.001&_msresflags=1089&_msresflags2=2&percolate=0&percolate_rt=0))

**542 - 550 506.3019 1010.5891 1010.5873 2 0 R.INITVQPAR.G**  ([Ions score 57](http://10.139.25.109/mascot/cgi/peptide_view.pl?file=../data/20120608/F008231.dat&query=1266&hit=1&index=orf225|tail&px=1&section=5&ave_thresh=38&_ignoreionsscorebelow=20&report=0&_sigthreshold=0.001&_msresflags=1089&_msresflags2=2&percolate=0&percolate_rt=0))

**551 - 565 818.4144 1634.8142 1634.8152 -1 0 R.GLDDITVGINYVDNK.L**  ([Ions score 90](http://10.139.25.109/mascot/cgi/peptide_view.pl?file=../data/20120608/F008231.dat&query=7332&hit=1&index=orf225|tail&px=1&section=5&ave_thresh=38&_ignoreionsscorebelow=20&report=0&_sigthreshold=0.001&_msresflags=1089&_msresflags2=2&percolate=0&percolate_rt=0))

**551 - 565 818.4147 1634.8148 1634.8152 0 0 R.GLDDITVGINYVDNK.L**  ([Ions score 48](http://10.139.25.109/mascot/cgi/peptide_view.pl?file=../data/20120608/F008231.dat&query=7333&hit=1&index=orf225|tail&px=1&section=5&ave_thresh=38&_ignoreionsscorebelow=20&report=0&_sigthreshold=0.001&_msresflags=1089&_msresflags2=2&percolate=0&percolate_rt=0))

**551 - 565 545.9456 1634.8150 1634.8152 0 0 R.GLDDITVGINYVDNK.L**  ([Ions score 48](http://10.139.25.109/mascot/cgi/peptide_view.pl?file=../data/20120608/F008231.dat&query=7334&hit=1&index=orf225|tail&px=1&section=5&ave_thresh=38&_ignoreionsscorebelow=20&report=0&_sigthreshold=0.001&_msresflags=1089&_msresflags2=2&percolate=0&percolate_rt=0))

**551 - 568 641.0023 1919.9850 1919.9840 0 0 R.GLDDITVGINYVDNKLTA.-**  ([Ions score 22](http://10.139.25.109/mascot/cgi/peptide_view.pl?file=../data/20120608/F008231.dat&query=9210&hit=1&index=orf225|tail&px=1&section=5&ave_thresh=38&_ignoreionsscorebelow=20&report=0&_sigthreshold=0.001&_msresflags=1089&_msresflags2=2&percolate=0&percolate_rt=0))

**551 - 568 641.0025 1919.9857 1919.9840 1 0 R.GLDDITVGINYVDNKLTA.-**  ([Ions score 36](http://10.139.25.109/mascot/cgi/peptide_view.pl?file=../data/20120608/F008231.dat&query=9211&hit=1&index=orf225|tail&px=1&section=5&ave_thresh=38&_ignoreionsscorebelow=20&report=0&_sigthreshold=0.001&_msresflags=1089&_msresflags2=2&percolate=0&percolate_rt=0))

**562 - 568 380.7135 759.4124 759.4127 0 0 Y.VDNKLTA.-**  ([Ions score 40](http://10.139.25.109/mascot/cgi/peptide_view.pl?file=../data/20120608/F008231.dat&query=143&hit=1&index=orf225|tail&px=1&section=5&ave_thresh=38&_ignoreionsscorebelow=20&report=0&_sigthreshold=0.001&_msresflags=1089&_msresflags2=2&percolate=0&percolate_rt=0))

2. [orf012|N-acetylmuramoyl-L alanine](http://10.139.25.109/mascot/cgi/protein_view.pl?file=../data/20120608/F008231.dat&hit=orf012|N-acetylmuramoyl-L-alanine&db_idx=1&px=1&ave_thresh=38&_ignoreionsscorebelow=20&report=0&_sigthreshold=0.001&_msresflags=1089&_msresflags2=2&percolate=0&percolate_rt=0) amidase|[vB_BceM_Bc431v3] **Mass:** 29574 **Score:** 1100  **emPAI:** 44.94

Sequence coverage: **87%**; Matched peptides shown in **Bold Red**

**1** **MGTYNVHGGH NGIVQGANYG NR**KEH**IMDRQ VKDALISKL**R **SLGHTVYDCT DETGSTQSAN LRNIVAKCN**A HRVDLDISLH **LNAFNGSANG VEVCYYDQQA**

**101 LAAKVSKQLS DDIGWSNR**GA KVRTDLYVLN **TTSAPAILIE LGFIDNESDM AKWNVDKIAD SICYAITGQR TGSGGNTGGG STGGSTGGGG YDSSWFTPQN**

**201 GVFTANTTIK VRSEPSVNAE HIRTL**ESGK**S YTYSSFGMER DGYVWIKGVD GTYLATGETR** DGK**RISYWGT FQ**

**Start - End Observed Mr(expt) Mr(calc) ppm Miss Sequence**

**1 - 22 579.7741 2315.0651 2315.0614 2 0 -.MGTYNVHGGHNGIVQGANYGNR.K**  ([Ions score 25](http://10.139.25.109/mascot/cgi/peptide_view.pl?file=../data/20120608/F008231.dat&query=11029&hit=1&index=orf012|N-acetylmuramoyl-L-alanine&px=1&section=5&ave_thresh=38&_ignoreionsscorebelow=20&report=0&_sigthreshold=0.001&_msresflags=1089&_msresflags2=2&percolate=0&percolate_rt=0))

**2 - 22 729.0149 2184.0212 2185.0209 0 0 M.GTYNVHGGHNGIVQGANYGNR.K**  ([Ions score 85](http://10.139.25.109/mascot/cgi/peptide_view.pl?file=../data/20120608/F008231.dat&query=10576&hit=1&index=orf012|N-acetylmuramoyl-L-alanine&px=1&section=5&ave_thresh=38&_ignoreionsscorebelow=20&report=0&_sigthreshold=0.001&_msresflags=1089&_msresflags2=2&percolate=0&percolate_rt=0))

**2 - 22 547.2638 2185.0239 2185.0209 1 0 M.GTYNVHGGHNGIVQGANYGNR.K**  ([Ions score 25](http://10.139.25.109/mascot/cgi/peptide_view.pl?file=../data/20120608/F008231.dat&query=10578&hit=1&index=orf012|N-acetylmuramoyl-L-alanine&px=1&section=5&ave_thresh=38&_ignoreionsscorebelow=20&report=0&_sigthreshold=0.001&_msresflags=1089&_msresflags2=2&percolate=0&percolate_rt=0))

**5 - 19 768.8683 1535.7221 1535.7229 -1 0 Y.NVHGGHNGIVQGANY.G**  ([Ions score 59](http://10.139.25.109/mascot/cgi/peptide_view.pl?file=../data/20120608/F008231.dat&query=6567&hit=1&index=orf012|N-acetylmuramoyl-L-alanine&px=1&section=5&ave_thresh=38&_ignoreionsscorebelow=20&report=0&_sigthreshold=0.001&_msresflags=1089&_msresflags2=2&percolate=0&percolate_rt=0))

**5 - 19 768.8688 1535.7230 1535.7229 0 0 Y.NVHGGHNGIVQGANY.G**  ([Ions score 53](http://10.139.25.109/mascot/cgi/peptide_view.pl?file=../data/20120608/F008231.dat&query=6568&hit=1&index=orf012|N-acetylmuramoyl-L-alanine&px=1&section=5&ave_thresh=38&_ignoreionsscorebelow=20&report=0&_sigthreshold=0.001&_msresflags=1089&_msresflags2=2&percolate=0&percolate_rt=0))

**5 - 19 512.9152 1535.7238 1535.7229 1 0 Y.NVHGGHNGIVQGANY.G**  ([Ions score 20](http://10.139.25.109/mascot/cgi/peptide_view.pl?file=../data/20120608/F008231.dat&query=6569&hit=1&index=orf012|N-acetylmuramoyl-L-alanine&px=1&section=5&ave_thresh=38&_ignoreionsscorebelow=20&report=0&_sigthreshold=0.001&_msresflags=1089&_msresflags2=2&percolate=0&percolate_rt=0))

**26 - 35 594.8242 1187.6338 1187.6332 0 0 H.IMDRQVKDAL.I**  ([Ions score 35](http://10.139.25.109/mascot/cgi/peptide_view.pl?file=../data/20120608/F008231.dat&query=3395&hit=1&index=orf012|N-acetylmuramoyl-L-alanine&px=1&section=5&ave_thresh=38&_ignoreionsscorebelow=20&report=0&_sigthreshold=0.001&_msresflags=1089&_msresflags2=2&percolate=0&percolate_rt=0))

**26 - 39 543.9836 1628.9291 1628.9283 0 0 H.IMDRQVKDALISKL.R**  ([Ions score 36](http://10.139.25.109/mascot/cgi/peptide_view.pl?file=../data/20120608/F008231.dat&query=7290&hit=1&index=orf012|N-acetylmuramoyl-L-alanine&px=1&section=5&ave_thresh=38&_ignoreionsscorebelow=20&report=0&_sigthreshold=0.001&_msresflags=1089&_msresflags2=2&percolate=0&percolate_rt=0))

**26 - 39 549.3151 1644.9235 1644.9232 0 0 H.IMDRQVKDALISKL.R**  Oxidation (M) ([Ions score 35](http://10.139.25.109/mascot/cgi/peptide_view.pl?file=../data/20120608/F008231.dat&query=7422&hit=1&index=orf012|N-acetylmuramoyl-L-alanine&px=1&section=5&ave_thresh=38&_ignoreionsscorebelow=20&report=0&_sigthreshold=0.001&_msresflags=1089&_msresflags2=2&percolate=0&percolate_rt=0))

**26 - 39 549.6482 1645.9227 1645.9073 9 0 H.IMDRQVKDALISKL.R**  Oxidation (M) ([Ions score 35](http://10.139.25.109/mascot/cgi/peptide_view.pl?file=../data/20120608/F008231.dat&query=7431&hit=1&index=orf012|N-acetylmuramoyl-L-alanine&px=1&section=5&ave_thresh=38&_ignoreionsscorebelow=20&report=0&_sigthreshold=0.001&_msresflags=1089&_msresflags2=2&percolate=0&percolate_rt=0))

**30 - 39 557.8445 1113.6745 1113.6757 -1 0 R.QVKDALISKL.R**  ([Ions score 30](http://10.139.25.109/mascot/cgi/peptide_view.pl?file=../data/20120608/F008231.dat&query=2430&hit=1&index=orf012|N-acetylmuramoyl-L-alanine&px=1&section=5&ave_thresh=38&_ignoreionsscorebelow=20&report=0&_sigthreshold=0.001&_msresflags=1089&_msresflags2=2&percolate=0&percolate_rt=0))

**30 - 39 372.2324 1113.6755 1113.6757 0 0 R.QVKDALISKL.R**  ([Ions score 26](http://10.139.25.109/mascot/cgi/peptide_view.pl?file=../data/20120608/F008231.dat&query=2431&hit=1&index=orf012|N-acetylmuramoyl-L-alanine&px=1&section=5&ave_thresh=38&_ignoreionsscorebelow=20&report=0&_sigthreshold=0.001&_msresflags=1089&_msresflags2=2&percolate=0&percolate_rt=0))

**30 - 39 557.8451 1113.6756 1113.6757 0 0 R.QVKDALISKL.R**  ([Ions score 34](http://10.139.25.109/mascot/cgi/peptide_view.pl?file=../data/20120608/F008231.dat&query=2432&hit=1&index=orf012|N-acetylmuramoyl-L-alanine&px=1&section=5&ave_thresh=38&_ignoreionsscorebelow=20&report=0&_sigthreshold=0.001&_msresflags=1089&_msresflags2=2&percolate=0&percolate_rt=0))

**41 - 62 1206.5418 2411.0680 2411.0659 1 0 R.SLGHTVYDCTDETGSTQSANLR.N**  ([Ions score 91](http://10.139.25.109/mascot/cgi/peptide_view.pl?file=../data/20120608/F008231.dat&query=11269&hit=1&index=orf012|N-acetylmuramoyl-L-alanine&px=1&section=5&ave_thresh=38&_ignoreionsscorebelow=20&report=0&_sigthreshold=0.001&_msresflags=1089&_msresflags2=2&percolate=0&percolate_rt=0))

**41 - 62 804.6965 2411.0660 2411.0659 0 0 R.SLGHTVYDCTDETGSTQSANLR.N**  Deamidated (NQ) ([Ions score 67](http://10.139.25.109/mascot/cgi/peptide_view.pl?file=../data/20120608/F008231.dat&query=11270&hit=1&index=orf012|N-acetylmuramoyl-L-alanine&px=1&section=5&ave_thresh=38&_ignoreionsscorebelow=20&report=0&_sigthreshold=0.001&_msresflags=1089&_msresflags2=2&percolate=0&percolate_rt=0))

**45 - 62 1009.4464 2016.8693 2016.8695 0 0 H.TVYDCTDETGSTQSANLR.N**  ([Ions score 100](http://10.139.25.109/mascot/cgi/peptide_view.pl?file=../data/20120608/F008231.dat&query=9649&hit=1&index=orf012|N-acetylmuramoyl-L-alanine&px=1&section=5&ave_thresh=38&_ignoreionsscorebelow=20&report=0&_sigthreshold=0.001&_msresflags=1089&_msresflags2=2&percolate=0&percolate_rt=0))

**48 - 61 749.8028 1497.5910 1497.5889 1 0 Y.DCTDETGSTQSANL.R**  ([Ions score 61](http://10.139.25.109/mascot/cgi/peptide_view.pl?file=../data/20120608/F008231.dat&query=6241&hit=1&index=orf012|N-acetylmuramoyl-L-alanine&px=1&section=5&ave_thresh=38&_ignoreionsscorebelow=20&report=0&_sigthreshold=0.001&_msresflags=1089&_msresflags2=2&percolate=0&percolate_rt=0))

**48 - 61 749.8029 1497.5912 1497.5889 1 0 Y.DCTDETGSTQSANL.R**  ([Ions score 79](http://10.139.25.109/mascot/cgi/peptide_view.pl?file=../data/20120608/F008231.dat&query=6242&hit=1&index=orf012|N-acetylmuramoyl-L-alanine&px=1&section=5&ave_thresh=38&_ignoreionsscorebelow=20&report=0&_sigthreshold=0.001&_msresflags=1089&_msresflags2=2&percolate=0&percolate_rt=0))

**48 - 69 818.7054 2453.0927 2453.0911 1 0 Y.DCTDETGSTQSANLRNIVAKCN.A**  ([Ions score 38](http://10.139.25.109/mascot/cgi/peptide_view.pl?file=../data/20120608/F008231.dat&query=11352&hit=1&index=orf012|N-acetylmuramoyl-L-alanine&px=1&section=5&ave_thresh=38&_ignoreionsscorebelow=20&report=0&_sigthreshold=0.001&_msresflags=1089&_msresflags2=2&percolate=0&percolate_rt=0))

**48 - 69 818.7052 2453.0921 2453.0911 0 0 Y.DCTDETGSTQSANLRNIVAKCN.A**  ([Ions score 30](http://10.139.25.109/mascot/cgi/peptide_view.pl?file=../data/20120608/F008231.dat&query=11353&hit=1&index=orf012|N-acetylmuramoyl-L-alanine&px=1&section=5&ave_thresh=38&_ignoreionsscorebelow=20&report=0&_sigthreshold=0.001&_msresflags=1089&_msresflags2=2&percolate=0&percolate_rt=0))

**81 - 96 889.3980 1776.7813 1776.7777 2 0 H.LNAFNGSANGVEVCYY.D**  ([Ions score 24](http://10.139.25.109/mascot/cgi/peptide_view.pl?file=../data/20120608/F008231.dat&query=8350&hit=1&index=orf012|N-acetylmuramoyl-L-alanine&px=1&section=5&ave_thresh=38&_ignoreionsscorebelow=20&report=0&_sigthreshold=0.001&_msresflags=1089&_msresflags2=2&percolate=0&percolate_rt=0))

**81 - 101 1167.5286 2333.0426 2333.0270 7 0 H.LNAFNGSANGVEVCYYDQQAL.A**  Deamidated (NQ) ([Ions score 50](http://10.139.25.109/mascot/cgi/peptide_view.pl?file=../data/20120608/F008231.dat&query=11078&hit=1&index=orf012|N-acetylmuramoyl-L-alanine&px=1&section=5&ave_thresh=38&_ignoreionsscorebelow=20&report=0&_sigthreshold=0.001&_msresflags=1089&_msresflags2=2&percolate=0&percolate_rt=0))

**85 - 96 666.7806 1331.5467 1331.5452 1 0 F.NGSANGVEVCYY.D**  ([Ions score 24](http://10.139.25.109/mascot/cgi/peptide_view.pl?file=../data/20120608/F008231.dat&query=5045&hit=1&index=orf012|N-acetylmuramoyl-L-alanine&px=1&section=5&ave_thresh=38&_ignoreionsscorebelow=20&report=0&_sigthreshold=0.001&_msresflags=1089&_msresflags2=2&percolate=0&percolate_rt=0))

**85 - 96 666.7810 1331.5475 1331.5452 2 0 F.NGSANGVEVCYY.D**  ([Ions score 35](http://10.139.25.109/mascot/cgi/peptide_view.pl?file=../data/20120608/F008231.dat&query=5046&hit=1&index=orf012|N-acetylmuramoyl-L-alanine&px=1&section=5&ave_thresh=38&_ignoreionsscorebelow=20&report=0&_sigthreshold=0.001&_msresflags=1089&_msresflags2=2&percolate=0&percolate_rt=0))

**85 - 101 944.4081 1886.8016 1886.8105 -5 0 F.NGSANGVEVCYYDQQAL.A**  ([Ions score 24](http://10.139.25.109/mascot/cgi/peptide_view.pl?file=../data/20120608/F008231.dat&query=9028&hit=1&index=orf012|N-acetylmuramoyl-L-alanine&px=1&section=5&ave_thresh=38&_ignoreionsscorebelow=20&report=0&_sigthreshold=0.001&_msresflags=1089&_msresflags2=2&percolate=0&percolate_rt=0))

**98 - 109 643.3721 1284.7296 1284.7401 -8 0 D.QQALAAKVSKQL.S**  Deamidated (NQ) ([Ions score 22](http://10.139.25.109/mascot/cgi/peptide_view.pl?file=../data/20120608/F008231.dat&query=4541&hit=1&index=orf012|N-acetylmuramoyl-L-alanine&px=1&section=5&ave_thresh=38&_ignoreionsscorebelow=20&report=0&_sigthreshold=0.001&_msresflags=1089&_msresflags2=2&percolate=0&percolate_rt=0))

**102 - 115 759.3993 1516.7840 1516.7885 -3 0 L.AAKVSKQLSDDIGW.S**  ([Ions score 47](http://10.139.25.109/mascot/cgi/peptide_view.pl?file=../data/20120608/F008231.dat&query=6410&hit=1&index=orf012|N-acetylmuramoyl-L-alanine&px=1&section=5&ave_thresh=38&_ignoreionsscorebelow=20&report=0&_sigthreshold=0.001&_msresflags=1089&_msresflags2=2&percolate=0&percolate_rt=0))

**102 - 115 506.6038 1516.7895 1516.7885 1 0 L.AAKVSKQLSDDIGW.S**  ([Ions score 30](http://10.139.25.109/mascot/cgi/peptide_view.pl?file=../data/20120608/F008231.dat&query=6412&hit=1&index=orf012|N-acetylmuramoyl-L-alanine&px=1&section=5&ave_thresh=38&_ignoreionsscorebelow=20&report=0&_sigthreshold=0.001&_msresflags=1089&_msresflags2=2&percolate=0&percolate_rt=0))

**108 - 118 637.2958 1272.5770 1272.5735 3 0 K.QLSDDIGWSNR.G**  Gln->pyro-Glu (N-term Q) ([Ions score 56](http://10.139.25.109/mascot/cgi/peptide_view.pl?file=../data/20120608/F008231.dat&query=4383&hit=1&index=orf012|N-acetylmuramoyl-L-alanine&px=1&section=5&ave_thresh=38&_ignoreionsscorebelow=20&report=0&_sigthreshold=0.001&_msresflags=1089&_msresflags2=2&percolate=0&percolate_rt=0))

**108 - 118 645.8082 1289.6018 1289.6000 1 0 K.QLSDDIGWSNR.G**  ([Ions score 45](http://10.139.25.109/mascot/cgi/peptide_view.pl?file=../data/20120608/F008231.dat&query=4600&hit=1&index=orf012|N-acetylmuramoyl-L-alanine&px=1&section=5&ave_thresh=38&_ignoreionsscorebelow=20&report=0&_sigthreshold=0.001&_msresflags=1089&_msresflags2=2&percolate=0&percolate_rt=0))

**108 - 118 645.8090 1289.6034 1289.6000 3 0 K.QLSDDIGWSNR.G**  ([Ions score 47](http://10.139.25.109/mascot/cgi/peptide_view.pl?file=../data/20120608/F008231.dat&query=4601&hit=1&index=orf012|N-acetylmuramoyl-L-alanine&px=1&section=5&ave_thresh=38&_ignoreionsscorebelow=20&report=0&_sigthreshold=0.001&_msresflags=1089&_msresflags2=2&percolate=0&percolate_rt=0))

**131 - 146 837.9534 1673.8923 1673.8876 3 0 N.TTSAPAILIELGFIDN.E**  ([Ions score 23](http://10.139.25.109/mascot/cgi/peptide_view.pl?file=../data/20120608/F008231.dat&query=7677&hit=1&index=orf012|N-acetylmuramoyl-L-alanine&px=1&section=5&ave_thresh=38&_ignoreionsscorebelow=20&report=0&_sigthreshold=0.001&_msresflags=1089&_msresflags2=2&percolate=0&percolate_rt=0))

**142 - 153 706.8113 1411.6081 1411.6078 0 0 L.GFIDNESDMAKW.N**  ([Ions score 64](http://10.139.25.109/mascot/cgi/peptide_view.pl?file=../data/20120608/F008231.dat&query=5658&hit=1&index=orf012|N-acetylmuramoyl-L-alanine&px=1&section=5&ave_thresh=38&_ignoreionsscorebelow=20&report=0&_sigthreshold=0.001&_msresflags=1089&_msresflags2=2&percolate=0&percolate_rt=0))

**153 - 164 742.3446 1482.6746 1482.6813 -4 0 K.WNVDKIADSICY.A**  ([Ions score 73](http://10.139.25.109/mascot/cgi/peptide_view.pl?file=../data/20120608/F008231.dat&query=6143&hit=1&index=orf012|N-acetylmuramoyl-L-alanine&px=1&section=5&ave_thresh=38&_ignoreionsscorebelow=20&report=0&_sigthreshold=0.001&_msresflags=1089&_msresflags2=2&percolate=0&percolate_rt=0))

**153 - 164 742.3456 1482.6767 1482.6813 -3 0 K.WNVDKIADSICY.A**  ([Ions score 57](http://10.139.25.109/mascot/cgi/peptide_view.pl?file=../data/20120608/F008231.dat&query=6144&hit=1&index=orf012|N-acetylmuramoyl-L-alanine&px=1&section=5&ave_thresh=38&_ignoreionsscorebelow=20&report=0&_sigthreshold=0.001&_msresflags=1089&_msresflags2=2&percolate=0&percolate_rt=0))

**153 - 170 704.0181 2109.0308 2109.0313 0 0 K.WNVDKIADSICYAITGQR.T** ([Ions score 58](http://10.139.25.109/mascot/cgi/peptide_view.pl?file=../data/20120608/F008231.dat&query=10221&hit=1&index=orf012|N-acetylmuramoyl-L-alanine&px=1&section=5&ave_thresh=38&_ignoreionsscorebelow=20&report=0&_sigthreshold=0.001&_msresflags=1089&_msresflags2=2&percolate=0&percolate_rt=0))

**154 - 164 649.3083 1296.6020 1296.6020 0 0 W.NVDKIADSICY.A**  ([Ions score 52](http://10.139.25.109/mascot/cgi/peptide_view.pl?file=../data/20120608/F008231.dat&query=4692&hit=1&index=orf012|N-acetylmuramoyl-L-alanine&px=1&section=5&ave_thresh=38&_ignoreionsscorebelow=20&report=0&_sigthreshold=0.001&_msresflags=1089&_msresflags2=2&percolate=0&percolate_rt=0))

**154 - 164 649.3088 1296.6031 1296.6020 1 0 W.NVDKIADSICY.A**  ([Ions score 44](http://10.139.25.109/mascot/cgi/peptide_view.pl?file=../data/20120608/F008231.dat&query=4693&hit=1&index=orf012|N-acetylmuramoyl-L-alanine&px=1&section=5&ave_thresh=38&_ignoreionsscorebelow=20&report=0&_sigthreshold=0.001&_msresflags=1089&_msresflags2=2&percolate=0&percolate_rt=0))

**158 - 170 734.3671 1466.7197 1466.7188 1 0 K.IADSICYAITGQR.T**  ([Ions score 84](http://10.139.25.109/mascot/cgi/peptide_view.pl?file=../data/20120608/F008231.dat&query=6034&hit=1&index=orf012|N-acetylmuramoyl-L-alanine&px=1&section=5&ave_thresh=38&_ignoreionsscorebelow=20&report=0&_sigthreshold=0.001&_msresflags=1089&_msresflags2=2&percolate=0&percolate_rt=0))

**158 - 170 734.3695 1466.7243 1466.7188 4 0 K.IADSICYAITGQR.T**  ([Ions score 74](http://10.139.25.109/mascot/cgi/peptide_view.pl?file=../data/20120608/F008231.dat&query=6035&hit=1&index=orf012|N-acetylmuramoyl-L-alanine&px=1&section=5&ave_thresh=38&_ignoreionsscorebelow=20&report=0&_sigthreshold=0.001&_msresflags=1089&_msresflags2=2&percolate=0&percolate_rt=0))

**165 - 195 916.7338 2747.1795 2747.1656 4 0 Y.AITGQRTGSGGNTGGGSTGGSTGGGGYDSSW.F**  ([Ions score 66](http://10.139.25.109/mascot/cgi/peptide_view.pl?file=../data/20120608/F008231.dat&query=11787&hit=1&index=orf012|N-acetylmuramoyl-L-alanine&px=1&section=5&ave_thresh=38&_ignoreionsscorebelow=20&report=0&_sigthreshold=0.001&_msresflags=1089&_msresflags2=2&percolate=0&percolate_rt=0))

**165 - 196 965.4250 2893.2515 2893.2499 1 0 Y.AITGQRTGSGGNTGGGSTGGSTGGGGYDSSWF.T** ([Ions score 90](http://10.139.25.109/mascot/cgi/peptide_view.pl?file=../data/20120608/F008231.dat&query=11894&hit=1&index=orf012|N-acetylmuramoyl-L-alanine&px=1&section=5&ave_thresh=38&_ignoreionsscorebelow=20&report=0&_sigthreshold=0.001&_msresflags=1089&_msresflags2=2&percolate=0&percolate_rt=0))

**165 - 196 965.4255 2893.2530 2893.2499 1 0 Y.AITGQRTGSGGNTGGGSTGGSTGGGGYDSSWF.T** ([Ions score 86](http://10.139.25.109/mascot/cgi/peptide_view.pl?file=../data/20120608/F008231.dat&query=11895&hit=1&index=orf012|N-acetylmuramoyl-L-alanine&px=1&section=5&ave_thresh=38&_ignoreionsscorebelow=20&report=0&_sigthreshold=0.001&_msresflags=1089&_msresflags2=2&percolate=0&percolate_rt=0))

**165 - 196 1447.6324 2893.2492 2893.2499 0 0 Y.AITGQRTGSGGNTGGGSTGGSTGGGGYDSSWF.T** ([Ions score 95](http://10.139.25.109/mascot/cgi/peptide_view.pl?file=../data/20120608/F008231.dat&query=11896&hit=1&index=orf012|N-acetylmuramoyl-L-alanine&px=1&section=5&ave_thresh=38&_ignoreionsscorebelow=20&report=0&_sigthreshold=0.001&_msresflags=1089&_msresflags2=2&percolate=0&percolate_rt=0))

**171 - 196 1134.4581 2266.9006 2266.8999 0 0 R.TGSGGNTGGGSTGGSTGGGGYDSSWF.T**  ([Ions score 65](http://10.139.25.109/mascot/cgi/peptide_view.pl?file=../data/20120608/F008231.dat&query=10871&hit=1&index=orf012|N-acetylmuramoyl-L-alanine&px=1&section=5&ave_thresh=38&_ignoreionsscorebelow=20&report=0&_sigthreshold=0.001&_msresflags=1089&_msresflags2=2&percolate=0&percolate_rt=0))

**171 - 210 935.9235 3739.6627 3739.6623 0 0 R.TGSGGNTGGGSTGGSTGGGGYDSSWFTPQNGVFTANTTIK.V** ([Ions score 31](http://10.139.25.109/mascot/cgi/peptide_view.pl?file=../data/20120608/F008231.dat&query=12242&hit=1&index=orf012|N-acetylmuramoyl-L-alanine&px=1&section=5&ave_thresh=38&_ignoreionsscorebelow=20&report=0&_sigthreshold=0.001&_msresflags=1089&_msresflags2=2&percolate=0&percolate_rt=0))

**171 - 210 1247.5618 3739.6619 3739.6623 0 0 R.TGSGGNTGGGSTGGSTGGGGYDSSWFTPQNGVFTANTTIK.V** ([Ions score 47](http://10.139.25.109/mascot/cgi/peptide_view.pl?file=../data/20120608/F008231.dat&query=12243&hit=1&index=orf012|N-acetylmuramoyl-L-alanine&px=1&section=5&ave_thresh=38&_ignoreionsscorebelow=20&report=0&_sigthreshold=0.001&_msresflags=1089&_msresflags2=2&percolate=0&percolate_rt=0))

**204 - 225 610.0851 2436.3091 2436.3085 0 0 F.TANTTIKVRSEPSVNAEHIRTL.E**  ([Ions score 48](http://10.139.25.109/mascot/cgi/peptide_view.pl?file=../data/20120608/F008231.dat&query=11319&hit=1&index=orf012|N-acetylmuramoyl-L-alanine&px=1&section=5&ave_thresh=38&_ignoreionsscorebelow=20&report=0&_sigthreshold=0.001&_msresflags=1089&_msresflags2=2&percolate=0&percolate_rt=0))

**204 - 225 610.0853 2436.3099 2436.3085 1 0 F.TANTTIKVRSEPSVNAEHIRTL.E**  ([Ions score 34](http://10.139.25.109/mascot/cgi/peptide_view.pl?file=../data/20120608/F008231.dat&query=11321&hit=1&index=orf012|N-acetylmuramoyl-L-alanine&px=1&section=5&ave_thresh=38&_ignoreionsscorebelow=20&report=0&_sigthreshold=0.001&_msresflags=1089&_msresflags2=2&percolate=0&percolate_rt=0))

**211 - 223 374.2009 1492.7745 1492.7746 0 0 K.VRSEPSVNAEHIR.T**  ([Ions score 36](http://10.139.25.109/mascot/cgi/peptide_view.pl?file=../data/20120608/F008231.dat&query=6216&hit=1&index=orf012|N-acetylmuramoyl-L-alanine&px=1&section=5&ave_thresh=38&_ignoreionsscorebelow=20&report=0&_sigthreshold=0.001&_msresflags=1089&_msresflags2=2&percolate=0&percolate_rt=0))

**213 - 223 413.5425 1237.6056 1237.6051 0 0 R.SEPSVNAEHIR.T**  ([Ions score 25](http://10.139.25.109/mascot/cgi/peptide_view.pl?file=../data/20120608/F008231.dat&query=4005&hit=1&index=orf012|N-acetylmuramoyl-L-alanine&px=1&section=5&ave_thresh=38&_ignoreionsscorebelow=20&report=0&_sigthreshold=0.001&_msresflags=1089&_msresflags2=2&percolate=0&percolate_rt=0))

**213 - 223 619.8102 1237.6058 1237.6051 1 0 R.SEPSVNAEHIR.T**  ([Ions score 48](http://10.139.25.109/mascot/cgi/peptide_view.pl?file=../data/20120608/F008231.dat&query=4006&hit=1&index=orf012|N-acetylmuramoyl-L-alanine&px=1&section=5&ave_thresh=38&_ignoreionsscorebelow=20&report=0&_sigthreshold=0.001&_msresflags=1089&_msresflags2=2&percolate=0&percolate_rt=0))

**213 - 223 619.8109 1237.6073 1237.6051 2 0 R.SEPSVNAEHIR.T**  ([Ions score 45](http://10.139.25.109/mascot/cgi/peptide_view.pl?file=../data/20120608/F008231.dat&query=4007&hit=1&index=orf012|N-acetylmuramoyl-L-alanine&px=1&section=5&ave_thresh=38&_ignoreionsscorebelow=20&report=0&_sigthreshold=0.001&_msresflags=1089&_msresflags2=2&percolate=0&percolate_rt=0))

**230 - 240 664.2853 1326.5561 1326.5550 1 0 K.SYTYSSFGMER.D**  ([Ions score 70](http://10.139.25.109/mascot/cgi/peptide_view.pl?file=../data/20120608/F008231.dat&query=4996&hit=1&index=orf012|N-acetylmuramoyl-L-alanine&px=1&section=5&ave_thresh=38&_ignoreionsscorebelow=20&report=0&_sigthreshold=0.001&_msresflags=1089&_msresflags2=2&percolate=0&percolate_rt=0))

**230 - 240 664.2871 1326.5595 1326.5550 3 0 K.SYTYSSFGMER.D**  ([Ions score 51](http://10.139.25.109/mascot/cgi/peptide_view.pl?file=../data/20120608/F008231.dat&query=4997&hit=1&index=orf012|N-acetylmuramoyl-L-alanine&px=1&section=5&ave_thresh=38&_ignoreionsscorebelow=20&report=0&_sigthreshold=0.001&_msresflags=1089&_msresflags2=2&percolate=0&percolate_rt=0))

**230 - 240 672.2829 1342.5512 1342.5500 1 0 K.SYTYSSFGMER.D**  Oxidation (M) ([Ions score 42](http://10.139.25.109/mascot/cgi/peptide_view.pl?file=../data/20120608/F008231.dat&query=5149&hit=1&index=orf012|N-acetylmuramoyl-L-alanine&px=1&section=5&ave_thresh=38&_ignoreionsscorebelow=20&report=0&_sigthreshold=0.001&_msresflags=1089&_msresflags2=2&percolate=0&percolate_rt=0))

**231 - 240 620.7698 1239.5250 1239.5230 2 0 S.YTYSSFGMER.D**  ([Ions score 29](http://10.139.25.109/mascot/cgi/peptide_view.pl?file=../data/20120608/F008231.dat&query=4023&hit=1&index=orf012|N-acetylmuramoyl-L-alanine&px=1&section=5&ave_thresh=38&_ignoreionsscorebelow=20&report=0&_sigthreshold=0.001&_msresflags=1089&_msresflags2=2&percolate=0&percolate_rt=0))

**234 - 243 574.7378 1147.4610 1147.4604 1 0 Y.SSFGMERDGY.V**  ([Ions score 36](http://10.139.25.109/mascot/cgi/peptide_view.pl?file=../data/20120608/F008231.dat&query=2852&hit=1&index=orf012|N-acetylmuramoyl-L-alanine&px=1&section=5&ave_thresh=38&_ignoreionsscorebelow=20&report=0&_sigthreshold=0.001&_msresflags=1089&_msresflags2=2&percolate=0&percolate_rt=0))

**234 - 243 574.7380 1147.4615 1147.4604 1 0 Y.SSFGMERDGY.V**  ([Ions score 70](http://10.139.25.109/mascot/cgi/peptide_view.pl?file=../data/20120608/F008231.dat&query=2853&hit=1&index=orf012|N-acetylmuramoyl-L-alanine&px=1&section=5&ave_thresh=38&_ignoreionsscorebelow=20&report=0&_sigthreshold=0.001&_msresflags=1089&_msresflags2=2&percolate=0&percolate_rt=0))

**234 - 243 582.7343 1163.4541 1163.4553 -1 0 Y.SSFGMERDGY.V**  Oxidation (M) ([Ions score 45](http://10.139.25.109/mascot/cgi/peptide_view.pl?file=../data/20120608/F008231.dat&query=3090&hit=1&index=orf012|N-acetylmuramoyl-L-alanine&px=1&section=5&ave_thresh=38&_ignoreionsscorebelow=20&report=0&_sigthreshold=0.001&_msresflags=1089&_msresflags2=2&percolate=0&percolate_rt=0))

**234 - 245 717.3123 1432.6100 1432.6082 1 0 Y.SSFGMERDGYVW.I**  ([Ions score 34](http://10.139.25.109/mascot/cgi/peptide_view.pl?file=../data/20120608/F008231.dat&query=5802&hit=1&index=orf012|N-acetylmuramoyl-L-alanine&px=1&section=5&ave_thresh=38&_ignoreionsscorebelow=20&report=0&_sigthreshold=0.001&_msresflags=1089&_msresflags2=2&percolate=0&percolate_rt=0))

**237 - 243 414.1714 826.3282 826.3279 0 0 F.GMERDGY.V**  ([Ions score 41](http://10.139.25.109/mascot/cgi/peptide_view.pl?file=../data/20120608/F008231.dat&query=386&hit=1&index=orf012|N-acetylmuramoyl-L-alanine&px=1&section=5&ave_thresh=38&_ignoreionsscorebelow=20&report=0&_sigthreshold=0.001&_msresflags=1089&_msresflags2=2&percolate=0&percolate_rt=0))

**237 - 245 556.7455 1111.4765 1111.4757 1 0 F.GMERDGYVW.I**  ([Ions score 21](http://10.139.25.109/mascot/cgi/peptide_view.pl?file=../data/20120608/F008231.dat&query=2391&hit=1&index=orf012|N-acetylmuramoyl-L-alanine&px=1&section=5&ave_thresh=38&_ignoreionsscorebelow=20&report=0&_sigthreshold=0.001&_msresflags=1089&_msresflags2=2&percolate=0&percolate_rt=0))

**237 - 245 556.7456 1111.4767 1111.4757 1 0 F.GMERDGYVW.I**  ([Ions score 20](http://10.139.25.109/mascot/cgi/peptide_view.pl?file=../data/20120608/F008231.dat&query=2392&hit=1&index=orf012|N-acetylmuramoyl-L-alanine&px=1&section=5&ave_thresh=38&_ignoreionsscorebelow=20&report=0&_sigthreshold=0.001&_msresflags=1089&_msresflags2=2&percolate=0&percolate_rt=0))

**241 - 247 440.7317 879.4489 879.4491 0 0 R.DGYVWIK.G**  ([Ions score 36](http://10.139.25.109/mascot/cgi/peptide_view.pl?file=../data/20120608/F008231.dat&query=616&hit=1&index=orf012|N-acetylmuramoyl-L-alanine&px=1&section=5&ave_thresh=38&_ignoreionsscorebelow=20&report=0&_sigthreshold=0.001&_msresflags=1089&_msresflags2=2&percolate=0&percolate_rt=0))

**244 - 254 625.8422 1249.6699 1249.6707 -1 0 Y.VWIKGVDGTYL.A**  ([Ions score 37](http://10.139.25.109/mascot/cgi/peptide_view.pl?file=../data/20120608/F008231.dat&query=4140&hit=1&index=orf012|N-acetylmuramoyl-L-alanine&px=1&section=5&ave_thresh=38&_ignoreionsscorebelow=20&report=0&_sigthreshold=0.001&_msresflags=1089&_msresflags2=2&percolate=0&percolate_rt=0))

**246 - 253 426.7270 851.4394 851.4389 1 0 W.IKGVDGTY.L**  ([Ions score 32](http://10.139.25.109/mascot/cgi/peptide_view.pl?file=../data/20120608/F008231.dat&query=512&hit=1&index=orf012|N-acetylmuramoyl-L-alanine&px=1&section=5&ave_thresh=38&_ignoreionsscorebelow=20&report=0&_sigthreshold=0.001&_msresflags=1089&_msresflags2=2&percolate=0&percolate_rt=0))

**246 - 254 483.2697 964.5249 964.5229 2 0 W.IKGVDGTYL.A**  ([Ions score 26](http://10.139.25.109/mascot/cgi/peptide_view.pl?file=../data/20120608/F008231.dat&query=958&hit=1&index=orf012|N-acetylmuramoyl-L-alanine&px=1&section=5&ave_thresh=38&_ignoreionsscorebelow=20&report=0&_sigthreshold=0.001&_msresflags=1089&_msresflags2=2&percolate=0&percolate_rt=0))

**248 - 260 670.3281 1338.6417 1338.6416 0 0 K.GVDGTYLATGETR.D**  ([Ions score 97](http://10.139.25.109/mascot/cgi/peptide_view.pl?file=../data/20120608/F008231.dat&query=5099&hit=1&index=orf012|N-acetylmuramoyl-L-alanine&px=1&section=5&ave_thresh=38&_ignoreionsscorebelow=20&report=0&_sigthreshold=0.001&_msresflags=1089&_msresflags2=2&percolate=0&percolate_rt=0))

**248 - 260 670.3292 1338.6439 1338.6416 2 0 K.GVDGTYLATGETR.D**  ([Ions score 88](http://10.139.25.109/mascot/cgi/peptide_view.pl?file=../data/20120608/F008231.dat&query=5100&hit=1&index=orf012|N-acetylmuramoyl-L-alanine&px=1&section=5&ave_thresh=38&_ignoreionsscorebelow=20&report=0&_sigthreshold=0.001&_msresflags=1089&_msresflags2=2&percolate=0&percolate_rt=0))

**264 - 272 579.2909 1156.5673 1156.5665 1 0 K.RISYWGTFQ.-**  ([Ions score 49](http://10.139.25.109/mascot/cgi/peptide_view.pl?file=../data/20120608/F008231.dat&query=2971&hit=1&index=orf012|N-acetylmuramoyl-L-alanine&px=1&section=5&ave_thresh=38&_ignoreionsscorebelow=20&report=0&_sigthreshold=0.001&_msresflags=1089&_msresflags2=2&percolate=0&percolate_rt=0))

**265 - 272 501.2398 1000.4651 1000.4654 0 0 R.ISYWGTFQ.-**  ([Ions score 47](http://10.139.25.109/mascot/cgi/peptide_view.pl?file=../data/20120608/F008231.dat&query=1138&hit=1&index=orf012|N-acetylmuramoyl-L-alanine&px=1&section=5&ave_thresh=38&_ignoreionsscorebelow=20&report=0&_sigthreshold=0.001&_msresflags=1089&_msresflags2=2&percolate=0&percolate_rt=0))

3. [orf168|hypothetical](http://10.139.25.109/mascot/cgi/protein_view.pl?file=../data/20120608/F008231.dat&hit=orf168|hypothetical&db_idx=1&px=1&ave_thresh=38&_ignoreionsscorebelow=20&report=0&_sigthreshold=0.001&_msresflags=1089&_msresflags2=2&percolate=0&percolate_rt=0) protein|[vB_BceM_Bc431v3]  **Mass:** 12243    **Score:** 562,  **emPAI:** 371.46

Sequence Coverage: **88%** Matched peptides shown in **Bold Red**

**1** MLEGIKK**AFT ATSYEPEQAP VEVNPIDDAV AAKLGYKVAE GQYKELQIDL ETGDVF**VLDE LL**VATPPEFS KDIFLVNLMA DFANANGIQL PKWTNEPLKI**

**101 AKAVADWEPQ N**

**Start - End Observed Mr(expt) Mr(calc) ppm Miss Sequence**

**8 - 33 911.7819 2732.3222 2732.3181 2 0 K.AFTATSYEPEQAPVEVNPIDDAVAAK.L**  [Ions score 28](http://10.139.25.109/mascot/cgi/peptide_view.pl?file=../data/20120608/F008231.dat&query=11780&hit=1&index=orf168|hypothetical&px=1&section=5&ave_thresh=38&_ignoreionsscorebelow=20&report=0&_sigthreshold=0.001&_msresflags=1089&_msresflags2=2&percolate=0&percolate_rt=0))

**8 - 36 1022.8376 3065.4893 3065.4870 1 0 K.AFTATSYEPEQAPVEVNPIDDAVAAKLGY.K**  ([Ions score 35](http://10.139.25.109/mascot/cgi/peptide_view.pl?file=../data/20120608/F008231.dat&query=11993&hit=1&index=orf168|hypothetical&px=1&section=5&ave_thresh=38&_ignoreionsscorebelow=20&report=0&_sigthreshold=0.001&_msresflags=1089&_msresflags2=2&percolate=0&percolate_rt=0))

**9 - 33 1331.6489 2661.2822 2661.2810 0 0 A.FTATSYEPEQAPVEVNPIDDAVAAK.L** ([Ions score 53](http://10.139.25.109/mascot/cgi/peptide_view.pl?file=../data/20120608/F008231.dat&query=11690&hit=1&index=orf168|hypothetical&px=1&section=5&ave_thresh=38&_ignoreionsscorebelow=20&report=0&_sigthreshold=0.001&_msresflags=1089&_msresflags2=2&percolate=0&percolate_rt=0))

**9 - 34 1388.1890 2774.3624 2774.3651 -1 0 A.FTATSYEPEQAPVEVNPIDDAVAAKL.G** ([Ions score 63](http://10.139.25.109/mascot/cgi/peptide_view.pl?file=../data/20120608/F008231.dat&query=11821&hit=1&index=orf168|hypothetical&px=1&section=5&ave_thresh=38&_ignoreionsscorebelow=20&report=0&_sigthreshold=0.001&_msresflags=1089&_msresflags2=2&percolate=0&percolate_rt=0))

**9 - 34 1388.1893 2774.3630 2774.3651 -1 0 A.FTATSYEPEQAPVEVNPIDDAVAAKL.G** ([Ions score 41](http://10.139.25.109/mascot/cgi/peptide_view.pl?file=../data/20120608/F008231.dat&query=11824&hit=1&index=orf168|hypothetical&px=1&section=5&ave_thresh=38&_ignoreionsscorebelow=20&report=0&_sigthreshold=0.001&_msresflags=1089&_msresflags2=2&percolate=0&percolate_rt=0))

**11 - 34 1264.6306 2527.2466 2527.2329 5 0 T.ATSYEPEQAPVEVNPIDDAVAAKL.G**  Deamidated (NQ) ([Ions score 60](http://10.139.25.109/mascot/cgi/peptide_view.pl?file=../data/20120608/F008231.dat&query=11463&hit=1&index=orf168|hypothetical&px=1&section=5&ave_thresh=38&_ignoreionsscorebelow=20&report=0&_sigthreshold=0.001&_msresflags=1089&_msresflags2=2&percolate=0&percolate_rt=0))

**15 - 33 996.5007 1990.9869 1990.9847 1 0 Y.EPEQAPVEVNPIDDAVAAK.L**  ([Ions score 84](http://10.139.25.109/mascot/cgi/peptide_view.pl?file=../data/20120608/F008231.dat&query=9488&hit=1&index=orf168|hypothetical&px=1&section=5&ave_thresh=38&_ignoreionsscorebelow=20&report=0&_sigthreshold=0.001&_msresflags=1089&_msresflags2=2&percolate=0&percolate_rt=0))

**15 - 33 665.0033 1991.9881 1991.9687 10 0 Y.EPEQAPVEVNPIDDAVAAK.L**  Deamidated (NQ) ([Ions score 57](http://10.139.25.109/mascot/cgi/peptide_view.pl?file=../data/20120608/F008231.dat&query=9493&hit=1&index=orf168|hypothetical&px=1&section=5&ave_thresh=38&_ignoreionsscorebelow=20&report=0&_sigthreshold=0.001&_msresflags=1089&_msresflags2=2&percolate=0&percolate_rt=0))

**15 - 33 665.0034 1991.9883 1991.9687 10 0 Y.EPEQAPVEVNPIDDAVAAK.L**  Deamidated (NQ) ([Ions score 60](http://10.139.25.109/mascot/cgi/peptide_view.pl?file=../data/20120608/F008231.dat&query=9494&hit=1&index=orf168|hypothetical&px=1&section=5&ave_thresh=38&_ignoreionsscorebelow=20&report=0&_sigthreshold=0.001&_msresflags=1089&_msresflags2=2&percolate=0&percolate_rt=0))

**15 - 33 997.0015 1991.9885 1991.9687 10 0 Y.EPEQAPVEVNPIDDAVAAK.L**  Deamidated (NQ) ([Ions score 65](http://10.139.25.109/mascot/cgi/peptide_view.pl?file=../data/20120608/F008231.dat&query=9495&hit=1&index=orf168|hypothetical&px=1&section=5&ave_thresh=38&_ignoreionsscorebelow=20&report=0&_sigthreshold=0.001&_msresflags=1089&_msresflags2=2&percolate=0&percolate_rt=0))

**15 - 34 1053.0447 2104.0748 2104.0688 3 0 Y.EPEQAPVEVNPIDDAVAAKL.G**  ([Ions score 66](http://10.139.25.109/mascot/cgi/peptide_view.pl?file=../data/20120608/F008231.dat&query=10191&hit=1&index=orf168|hypothetical&px=1&section=5&ave_thresh=38&_ignoreionsscorebelow=20&report=0&_sigthreshold=0.001&_msresflags=1089&_msresflags2=2&percolate=0&percolate_rt=0))

**15 - 34 702.6985 2105.0738 2105.0528 10 0 Y.EPEQAPVEVNPIDDAVAAKL.G**  Deamidated (NQ) ([Ions score 36](http://10.139.25.109/mascot/cgi/peptide_view.pl?file=../data/20120608/F008231.dat&query=10194&hit=1&index=orf168|hypothetical&px=1&section=5&ave_thresh=38&_ignoreionsscorebelow=20&report=0&_sigthreshold=0.001&_msresflags=1089&_msresflags2=2&percolate=0&percolate_rt=0))

**15 - 34 1053.5442 2105.0738 2105.0528 10 0 Y.EPEQAPVEVNPIDDAVAAKL.G**  Deamidated (NQ) ([Ions score 64](http://10.139.25.109/mascot/cgi/peptide_view.pl?file=../data/20120608/F008231.dat&query=10195&hit=1&index=orf168|hypothetical&px=1&section=5&ave_thresh=38&_ignoreionsscorebelow=20&report=0&_sigthreshold=0.001&_msresflags=1089&_msresflags2=2&percolate=0&percolate_rt=0))

**17 - 33 883.4514 1764.8883 1764.8894 -1 0 P.EQAPVEVNPIDDAVAAK.L**  ([Ions score 39](http://10.139.25.109/mascot/cgi/peptide_view.pl?file=../data/20120608/F008231.dat&query=8262&hit=1&index=orf168|hypothetical&px=1&section=5&ave_thresh=38&_ignoreionsscorebelow=20&report=0&_sigthreshold=0.001&_msresflags=1089&_msresflags2=2&percolate=0&percolate_rt=0))

**17 - 34 939.9963 1877.9781 1877.9734 2 0 P.EQAPVEVNPIDDAVAAKL.G**  ([Ions score 34](http://10.139.25.109/mascot/cgi/peptide_view.pl?file=../data/20120608/F008231.dat&query=8984&hit=1&index=orf168|hypothetical&px=1&section=5&ave_thresh=38&_ignoreionsscorebelow=20&report=0&_sigthreshold=0.001&_msresflags=1089&_msresflags2=2&percolate=0&percolate_rt=0))

**19 - 34 811.4435 1620.8724 1620.8723 0 0 Q.APVEVNPIDDAVAAKL.G**  ([Ions score 68](http://10.139.25.109/mascot/cgi/peptide_view.pl?file=../data/20120608/F008231.dat&query=7236&hit=1&index=orf168|hypothetical&px=1&section=5&ave_thresh=38&_ignoreionsscorebelow=20&report=0&_sigthreshold=0.001&_msresflags=1089&_msresflags2=2&percolate=0&percolate_rt=0))

**19 - 34 811.4437 1620.8729 1620.8723 0 0 Q.APVEVNPIDDAVAAKL.G**  ([Ions score 70](http://10.139.25.109/mascot/cgi/peptide_view.pl?file=../data/20120608/F008231.dat&query=7237&hit=1&index=orf168|hypothetical&px=1&section=5&ave_thresh=38&_ignoreionsscorebelow=20&report=0&_sigthreshold=0.001&_msresflags=1089&_msresflags2=2&percolate=0&percolate_rt=0))

**19 - 36 921.4815 1840.9485 1840.9571 -5 0 Q.APVEVNPIDDAVAAKLGY.K**  ([Ions score 70](http://10.139.25.109/mascot/cgi/peptide_view.pl?file=../data/20120608/F008231.dat&query=8782&hit=1&index=orf168|hypothetical&px=1&section=5&ave_thresh=38&_ignoreionsscorebelow=20&report=0&_sigthreshold=0.001&_msresflags=1089&_msresflags2=2&percolate=0&percolate_rt=0))

**19 - 36 614.6600 1840.9581 1840.9571 1 0 Q.APVEVNPIDDAVAAKLGY.K**  ([Ions score 35](http://10.139.25.109/mascot/cgi/peptide_view.pl?file=../data/20120608/F008231.dat&query=8784&hit=1&index=orf168|hypothetical&px=1&section=5&ave_thresh=38&_ignoreionsscorebelow=20&report=0&_sigthreshold=0.001&_msresflags=1089&_msresflags2=2&percolate=0&percolate_rt=0))

**21 - 33 670.8568 1339.6989 1339.6983 0 0 P.VEVNPIDDAVAAK.L**  ([Ions score 71](http://10.139.25.109/mascot/cgi/peptide_view.pl?file=../data/20120608/F008231.dat&query=5118&hit=1&index=orf168|hypothetical&px=1&section=5&ave_thresh=38&_ignoreionsscorebelow=20&report=0&_sigthreshold=0.001&_msresflags=1089&_msresflags2=2&percolate=0&percolate_rt=0))

**21 - 33 670.8573 1339.7000 1339.6983 1 0 P.VEVNPIDDAVAAK.L**  ([Ions score 60](http://10.139.25.109/mascot/cgi/peptide_view.pl?file=../data/20120608/F008231.dat&query=5119&hit=1&index=orf168|hypothetical&px=1&section=5&ave_thresh=38&_ignoreionsscorebelow=20&report=0&_sigthreshold=0.001&_msresflags=1089&_msresflags2=2&percolate=0&percolate_rt=0))

**21 - 34 727.3958 1452.7769 1452.7824 -4 0 P.VEVNPIDDAVAAKL.G**  ([Ions score 53](http://10.139.25.109/mascot/cgi/peptide_view.pl?file=../data/20120608/F008231.dat&query=5931&hit=1&index=orf168|hypothetical&px=1&section=5&ave_thresh=38&_ignoreionsscorebelow=20&report=0&_sigthreshold=0.001&_msresflags=1089&_msresflags2=2&percolate=0&percolate_rt=0))

**21 - 34 727.3980 1452.7815 1452.7824 -1 0 P.VEVNPIDDAVAAKL.G**  ([Ions score 60](http://10.139.25.109/mascot/cgi/peptide_view.pl?file=../data/20120608/F008231.dat&query=5932&hit=1&index=orf168|hypothetical&px=1&section=5&ave_thresh=38&_ignoreionsscorebelow=20&report=0&_sigthreshold=0.001&_msresflags=1089&_msresflags2=2&percolate=0&percolate_rt=0))

**21 - 36 837.4416 1672.8686 1672.8672 1 0 P.VEVNPIDDAVAAKLGY.K**  ([Ions score 62](http://10.139.25.109/mascot/cgi/peptide_view.pl?file=../data/20120608/F008231.dat&query=7666&hit=1&index=orf168|hypothetical&px=1&section=5&ave_thresh=38&_ignoreionsscorebelow=20&report=0&_sigthreshold=0.001&_msresflags=1089&_msresflags2=2&percolate=0&percolate_rt=0))

**23 - 34 613.3428 1224.6711 1224.6714 -0 0 E.VNPIDDAVAAKL.G**  ([Ions score 41](http://10.139.25.109/mascot/cgi/peptide_view.pl?file=../data/20120608/F008231.dat&query=3853&hit=1&index=orf168|hypothetical&px=1&section=5&ave_thresh=38&_ignoreionsscorebelow=20&report=0&_sigthreshold=0.001&_msresflags=1089&_msresflags2=2&percolate=0&percolate_rt=0))

**23 - 34 613.3442 1224.6738 1224.6714 2 0 E.VNPIDDAVAAKL.G**  ([Ions score 20](http://10.139.25.109/mascot/cgi/peptide_view.pl?file=../data/20120608/F008231.dat&query=3854&hit=1&index=orf168|hypothetical&px=1&section=5&ave_thresh=38&_ignoreionsscorebelow=20&report=0&_sigthreshold=0.001&_msresflags=1089&_msresflags2=2&percolate=0&percolate_rt=0))

**37 - 43 397.7056 793.3966 793.3970 -0 0 Y.KVAEGQY.K**  ([Ions score 26](http://10.139.25.109/mascot/cgi/peptide_view.pl?file=../data/20120608/F008231.dat&query=255&hit=1&index=orf168|hypothetical&px=1&section=5&ave_thresh=38&_ignoreionsscorebelow=20&report=0&_sigthreshold=0.001&_msresflags=1089&_msresflags2=2&percolate=0&percolate_rt=0))

**38 - 44 397.7059 793.3972 793.3970 0 0 K.VAEGQYK.E**  ([Ions score 39](http://10.139.25.109/mascot/cgi/peptide_view.pl?file=../data/20120608/F008231.dat&query=256&hit=1&index=orf168|hypothetical&px=1&section=5&ave_thresh=38&_ignoreionsscorebelow=20&report=0&_sigthreshold=0.001&_msresflags=1089&_msresflags2=2&percolate=0&percolate_rt=0))

**44 - 56 753.8881 1505.7617 1505.7613 0 0 Y.KELQIDLETGDVF.V**  ([Ions score 63](http://10.139.25.109/mascot/cgi/peptide_view.pl?file=../data/20120608/F008231.dat&query=6309&hit=1&index=orf168|hypothetical&px=1&section=5&ave_thresh=38&_ignoreionsscorebelow=20&report=0&_sigthreshold=0.001&_msresflags=1089&_msresflags2=2&percolate=0&percolate_rt=0))

**63 - 74 675.8507 1349.6869 1349.6867 0 0 L.VATPPEFSKDIF.L**  ([Ions score 38](http://10.139.25.109/mascot/cgi/peptide_view.pl?file=../data/20120608/F008231.dat&query=5185&hit=1&index=orf168|hypothetical&px=1&section=5&ave_thresh=38&_ignoreionsscorebelow=20&report=0&_sigthreshold=0.001&_msresflags=1089&_msresflags2=2&percolate=0&percolate_rt=0))

**63 - 74 675.8519 1349.6893 1349.6867 2 0 L.VATPPEFSKDIF.L**  ([Ions score 43](http://10.139.25.109/mascot/cgi/peptide_view.pl?file=../data/20120608/F008231.dat&query=5186&hit=1&index=orf168|hypothetical&px=1&section=5&ave_thresh=38&_ignoreionsscorebelow=20&report=0&_sigthreshold=0.001&_msresflags=1089&_msresflags2=2&percolate=0&percolate_rt=0))

**66 - 79 555.9532 1664.8377 1664.8484 -6 0 T.PPEFSKDIFLVNLM.A**  Oxidation (M) ([Ions score 27](http://10.139.25.109/mascot/cgi/peptide_view.pl?file=../data/20120608/F008231.dat&query=7597&hit=1&index=orf168|hypothetical&px=1&section=5&ave_thresh=38&_ignoreionsscorebelow=20&report=0&_sigthreshold=0.001&_msresflags=1089&_msresflags2=2&percolate=0&percolate_rt=0))

**79 - 98 1115.5511 2229.0876 2229.0888 -1 0 L.MADFANANGIQLPKWTNEPL.K**  ([Ions score 64](http://10.139.25.109/mascot/cgi/peptide_view.pl?file=../data/20120608/F008231.dat&query=10728&hit=1&index=orf168|hypothetical&px=1&section=5&ave_thresh=38&_ignoreionsscorebelow=20&report=0&_sigthreshold=0.001&_msresflags=1089&_msresflags2=2&percolate=0&percolate_rt=0))

**80 - 88 447.2038 892.3930 892.3926 0 0 M.ADFANANGI.Q**  Deamidated (NQ) ([Ions score 25](http://10.139.25.109/mascot/cgi/peptide_view.pl?file=../data/20120608/F008231.dat&query=665&hit=1&index=orf168|hypothetical&px=1&section=5&ave_thresh=38&_ignoreionsscorebelow=20&report=0&_sigthreshold=0.001&_msresflags=1089&_msresflags2=2&percolate=0&percolate_rt=0))

**80 - 98 1050.0321 2098.0496 2098.0483 1 0 M.ADFANANGIQLPKWTNEPL.K**  ([Ions score 56](http://10.139.25.109/mascot/cgi/peptide_view.pl?file=../data/20120608/F008231.dat&query=10148&hit=1&index=orf168|hypothetical&px=1&section=5&ave_thresh=38&_ignoreionsscorebelow=20&report=0&_sigthreshold=0.001&_msresflags=1089&_msresflags2=2&percolate=0&percolate_rt=0))

**83 - 98 883.4693 1764.9240 1764.9158 5 0 F.ANANGIQLPKWTNEPL.K**  ([Ions score 40](http://10.139.25.109/mascot/cgi/peptide_view.pl?file=../data/20120608/F008231.dat&query=8263&hit=1&index=orf168|hypothetical&px=1&section=5&ave_thresh=38&_ignoreionsscorebelow=20&report=0&_sigthreshold=0.001&_msresflags=1089&_msresflags2=2&percolate=0&percolate_rt=0))

**93 - 99 444.2348 886.4550 886.4548 0 0 K.WTNEPLK.I**  ([Ions score 49](http://10.139.25.109/mascot/cgi/peptide_view.pl?file=../data/20120608/F008231.dat&query=643&hit=1&index=orf168|hypothetical&px=1&section=5&ave_thresh=38&_ignoreionsscorebelow=20&report=0&_sigthreshold=0.001&_msresflags=1089&_msresflags2=2&percolate=0&percolate_rt=0))

**99 - 111 735.3874 1468.7602 1468.7674 -5 0 L.KIAKAVADWEPQN.-**  ([Ions score 47](http://10.139.25.109/mascot/cgi/peptide_view.pl?file=../data/20120608/F008231.dat&query=6047&hit=1&index=orf168|hypothetical&px=1&section=5&ave_thresh=38&_ignoreionsscorebelow=20&report=0&_sigthreshold=0.001&_msresflags=1089&_msresflags2=2&percolate=0&percolate_rt=0))

**99 - 111 735.3909 1468.7672 1468.7674 -0 0 L.KIAKAVADWEPQN.-**  ([Ions score 48](http://10.139.25.109/mascot/cgi/peptide_view.pl?file=../data/20120608/F008231.dat&query=6048&hit=1&index=orf168|hypothetical&px=1&section=5&ave_thresh=38&_ignoreionsscorebelow=20&report=0&_sigthreshold=0.001&_msresflags=1089&_msresflags2=2&percolate=0&percolate_rt=0))

**103 - 111 515.2344 1028.4543 1028.4563 -2 0 K.AVADWEPQN.-**  ([Ions score 21](http://10.139.25.109/mascot/cgi/peptide_view.pl?file=../data/20120608/F008231.dat&query=1446&hit=1&index=orf168|hypothetical&px=1&section=5&ave_thresh=38&_ignoreionsscorebelow=20&report=0&_sigthreshold=0.001&_msresflags=1089&_msresflags2=2&percolate=0&percolate_rt=0))

4. [orf239|conserved](http://10.139.25.109/mascot/cgi/protein_view.pl?file=../data/20120608/F008231.dat&hit=orf239|conserved&db_idx=1&px=1&ave_thresh=38&_ignoreionsscorebelow=20&report=0&_sigthreshold=0.001&_msresflags=1089&_msresflags2=2&percolate=0&percolate_rt=0) hypothetical protein|[vB_BceM_Bc431v3]    **Mass:** 23503  **Score:** 474   **emPAI:** 6.29

Sequence Coverage: **58%**; Matched peptides shown in **Bold Red**

**1** MEKM**SVQRGL MEL**KTLGNRI TR**ATQQAFVS FYVGDK**GAPQ GF**KTPDEFSS YAQGRYDSAT DLIKR**R**NAIK AAIIQSN**AVT **KVTVAGKQMT VAEAIDRK**DS

**101** IVH**EKVLLQQ LQSQFSEITR** R**VASQQQVLD AR**IDKVLEEE GGKDRKVDDA DHARIVKNAE SR**YKPNLVDP IGIR**KVIEQM EEDINSFELD VDASLSEINA

**201** R**TDIEFEVK**

**Start - End Observed Mr(expt) Mr(calc) ppm Miss Sequence**

**5 - 13 516.7777 1031.5408 1031.5433 -3 0 M.SVQRGLMEL.K**  ([Ions score 28](http://10.139.25.109/mascot/cgi/peptide_view.pl?file=../data/20120608/F008231.dat&query=1487&hit=1&index=orf239|conserved&px=1&section=5&ave_thresh=38&_ignoreionsscorebelow=20&report=0&_sigthreshold=0.001&_msresflags=1089&_msresflags2=2&percolate=0&percolate_rt=0))

**5 - 13 516.7778 1031.5411 1031.5433 -2 0 M.SVQRGLMEL.K**  ([Ions score 30](http://10.139.25.109/mascot/cgi/peptide_view.pl?file=../data/20120608/F008231.dat&query=1488&hit=1&index=orf239|conserved&px=1&section=5&ave_thresh=38&_ignoreionsscorebelow=20&report=0&_sigthreshold=0.001&_msresflags=1089&_msresflags2=2&percolate=0&percolate_rt=0))

**23 - 36 780.8888 1559.7630 1559.7620 1 0 R.ATQQAFVSFYVGDK.G**  ([Ions score 83](http://10.139.25.109/mascot/cgi/peptide_view.pl?file=../data/20120608/F008231.dat&query=6754&hit=1&index=orf239|conserved&px=1&section=5&ave_thresh=38&_ignoreionsscorebelow=20&report=0&_sigthreshold=0.001&_msresflags=1089&_msresflags2=2&percolate=0&percolate_rt=0))

**23 - 36 780.8899 1559.7652 1559.7620 2 0 R.ATQQAFVSFYVGDK.G**  ([Ions score 70](http://10.139.25.109/mascot/cgi/peptide_view.pl?file=../data/20120608/F008231.dat&query=6755&hit=1&index=orf239|conserved&px=1&section=5&ave_thresh=38&_ignoreionsscorebelow=20&report=0&_sigthreshold=0.001&_msresflags=1089&_msresflags2=2&percolate=0&percolate_rt=0))

**43 - 51 537.2437 1072.4727 1072.4713 1 0 F.KTPDEFSSY.A**  ([Ions score 38](http://10.139.25.109/mascot/cgi/peptide_view.pl?file=../data/20120608/F008231.dat&query=1923&hit=1&index=orf239|conserved&px=1&section=5&ave_thresh=38&_ignoreionsscorebelow=20&report=0&_sigthreshold=0.001&_msresflags=1089&_msresflags2=2&percolate=0&percolate_rt=0))

**43 - 51 537.2466 1072.4786 1072.4713 7 0 F.KTPDEFSSY.A**  ([Ions score 57](http://10.139.25.109/mascot/cgi/peptide_view.pl?file=../data/20120608/F008231.dat&query=1925&hit=1&index=orf239|conserved&px=1&section=5&ave_thresh=38&_ignoreionsscorebelow=20&report=0&_sigthreshold=0.001&_msresflags=1089&_msresflags2=2&percolate=0&percolate_rt=0))

**44 - 55 679.3045 1356.5944 1356.5946 -0 0 K.TPDEFSSYAQGR.Y**  ([Ions score 92](http://10.139.25.109/mascot/cgi/peptide_view.pl?file=../data/20120608/F008231.dat&query=5239&hit=1&index=orf239|conserved&px=1&section=5&ave_thresh=38&_ignoreionsscorebelow=20&report=0&_sigthreshold=0.001&_msresflags=1089&_msresflags2=2&percolate=0&percolate_rt=0))

**44 - 55 679.3056 1356.5966 1356.5946 2 0 K.TPDEFSSYAQGR.Y**  ([Ions score 86](http://10.139.25.109/mascot/cgi/peptide_view.pl?file=../data/20120608/F008231.dat&query=5240&hit=1&index=orf239|conserved&px=1&section=5&ave_thresh=38&_ignoreionsscorebelow=20&report=0&_sigthreshold=0.001&_msresflags=1089&_msresflags2=2&percolate=0&percolate_rt=0))

**44 - 64 789.0397 2364.0972 2364.0757 9 0 K.TPDEFSSYAQGRYDSATDLIK.R**  Deamidated (NQ) ([Ions score 65](http://10.139.25.109/mascot/cgi/peptide_view.pl?file=../data/20120608/F008231.dat&query=11158&hit=1&index=orf239|conserved&px=1&section=5&ave_thresh=38&_ignoreionsscorebelow=20&report=0&_sigthreshold=0.001&_msresflags=1089&_msresflags2=2&percolate=0&percolate_rt=0))

**44 - 64 789.0398 2364.0975 2364.0757 9 0 K.TPDEFSSYAQGRYDSATDLIK.R**  Deamidated (NQ) ([Ions score 40](http://10.139.25.109/mascot/cgi/peptide_view.pl?file=../data/20120608/F008231.dat&query=11159&hit=1&index=orf239|conserved&px=1&section=5&ave_thresh=38&_ignoreionsscorebelow=20&report=0&_sigthreshold=0.001&_msresflags=1089&_msresflags2=2&percolate=0&percolate_rt=0))

**56 - 64 513.2613 1024.5081 1024.5077 0 0 R.YDSATDLIK.R**  ([Ions score 43](http://10.139.25.109/mascot/cgi/peptide_view.pl?file=../data/20120608/F008231.dat&query=1416&hit=1&index=orf239|conserved&px=1&section=5&ave_thresh=38&_ignoreionsscorebelow=20&report=0&_sigthreshold=0.001&_msresflags=1089&_msresflags2=2&percolate=0&percolate_rt=0))

**56 - 64 513.2613 1024.5081 1024.5077 0 0 R.YDSATDLIK.R**  ([Ions score 36](http://10.139.25.109/mascot/cgi/peptide_view.pl?file=../data/20120608/F008231.dat&query=1417&hit=1&index=orf239|conserved&px=1&section=5&ave_thresh=38&_ignoreionsscorebelow=20&report=0&_sigthreshold=0.001&_msresflags=1089&_msresflags2=2&percolate=0&percolate_rt=0))

**56 - 65 394.5434 1180.6085 1180.6088 -0 0 R.YDSATDLIKR.R**  ([Ions score 21](http://10.139.25.109/mascot/cgi/peptide_view.pl?file=../data/20120608/F008231.dat&query=3308&hit=1&index=orf239|conserved&px=1&section=5&ave_thresh=38&_ignoreionsscorebelow=20&report=0&_sigthreshold=0.001&_msresflags=1089&_msresflags2=2&percolate=0&percolate_rt=0))

**67 - 75 471.2911 940.5676 940.5705 -3 0 R.NAIKAAIIQ.S**  ([Ions score 46](http://10.139.25.109/mascot/cgi/peptide_view.pl?file=../data/20120608/F008231.dat&query=869&hit=1&index=orf239|conserved&px=1&section=5&ave_thresh=38&_ignoreionsscorebelow=20&report=0&_sigthreshold=0.001&_msresflags=1089&_msresflags2=2&percolate=0&percolate_rt=0))

**67 - 77 571.8292 1141.6438 1141.6455 -1 0 R.NAIKAAIIQSN.A**  ([Ions score 28](http://10.139.25.109/mascot/cgi/peptide_view.pl?file=../data/20120608/F008231.dat&query=2780&hit=1&index=orf239|conserved&px=1&section=5&ave_thresh=38&_ignoreionsscorebelow=20&report=0&_sigthreshold=0.001&_msresflags=1089&_msresflags2=2&percolate=0&percolate_rt=0))

**68 - 75 414.2710 826.5275 826.5276 -0 0 N.AIKAAIIQ.S**  ([Ions score 20](http://10.139.25.109/mascot/cgi/peptide_view.pl?file=../data/20120608/F008231.dat&query=393&hit=1&index=orf239|conserved&px=1&section=5&ave_thresh=38&_ignoreionsscorebelow=20&report=0&_sigthreshold=0.001&_msresflags=1089&_msresflags2=2&percolate=0&percolate_rt=0))

**81 - 89 481.2786 960.5426 960.5426 -0 0 T.KVTVAGKQM.T**  ([Ions score 56](http://10.139.25.109/mascot/cgi/peptide_view.pl?file=../data/20120608/F008231.dat&query=938&hit=1&index=orf239|conserved&px=1&section=5&ave_thresh=38&_ignoreionsscorebelow=20&report=0&_sigthreshold=0.001&_msresflags=1089&_msresflags2=2&percolate=0&percolate_rt=0))

**88 - 98 622.8196 1243.6247 1243.6231 1 0 K.QMTVAEAIDRK.D**  Gln->pyro-Glu (N-term Q) ([Ions score 60](http://10.139.25.109/mascot/cgi/peptide_view.pl?file=../data/20120608/F008231.dat&query=4065&hit=1&index=orf239|conserved&px=1&section=5&ave_thresh=38&_ignoreionsscorebelow=20&report=0&_sigthreshold=0.001&_msresflags=1089&_msresflags2=2&percolate=0&percolate_rt=0))

**88 - 98 622.8201 1243.6256 1243.6231 2 0 K.QMTVAEAIDRK.D**  Gln->pyro-Glu (N-term Q) ([Ions score 60](http://10.139.25.109/mascot/cgi/peptide_view.pl?file=../data/20120608/F008231.dat&query=4066&hit=1&index=orf239|conserved&px=1&section=5&ave_thresh=38&_ignoreionsscorebelow=20&report=0&_sigthreshold=0.001&_msresflags=1089&_msresflags2=2&percolate=0&percolate_rt=0))

**88 - 98 631.3319 1260.6491 1260.6496 0 0 K.QMTVAEAIDRK.D**  ([Ions score 52](http://10.139.25.109/mascot/cgi/peptide_view.pl?file=../data/20120608/F008231.dat&query=4258&hit=1&index=orf239|conserved&px=1&section=5&ave_thresh=38&_ignoreionsscorebelow=20&report=0&_sigthreshold=0.001&_msresflags=1089&_msresflags2=2&percolate=0&percolate_rt=0))

**88 - 98 421.2241 1260.6504 1260.6496 1 0 K.QMTVAEAIDRK.D**  ([Ions score 32](http://10.139.25.109/mascot/cgi/peptide_view.pl?file=../data/20120608/F008231.dat&query=4259&hit=1&index=orf239|conserved&px=1&section=5&ave_thresh=38&_ignoreionsscorebelow=20&report=0&_sigthreshold=0.001&_msresflags=1089&_msresflags2=2&percolate=0&percolate_rt=0))

**88 - 98 631.3334 1260.6523 1260.6496 2 0 K.QMTVAEAIDRK.D**  ([Ions score 50](http://10.139.25.109/mascot/cgi/peptide_view.pl?file=../data/20120608/F008231.dat&query=4260&hit=1&index=orf239|conserved&px=1&section=5&ave_thresh=38&_ignoreionsscorebelow=20&report=0&_sigthreshold=0.001&_msresflags=1089&_msresflags2=2&percolate=0&percolate_rt=0))

**104 - 112 550.3269 1098.6392 1098.6284 10 0 H.EKVLLQQLQ.S**  Deamidated (NQ) ([Ions score 30](http://10.139.25.109/mascot/cgi/peptide_view.pl?file=../data/20120608/F008231.dat&query=2278&hit=2&index=orf239|conserved&px=1&section=5&ave_thresh=38&_ignoreionsscorebelow=20&report=0&_sigthreshold=0.001&_msresflags=1089&_msresflags2=2&percolate=0&percolate_rt=0))

**104 - 115 730.9093 1459.8040 1459.8035 0 0 H.EKVLLQQLQSQF.S**  ([Ions score 52](http://10.139.25.109/mascot/cgi/peptide_view.pl?file=../data/20120608/F008231.dat&query=5984&hit=1&index=orf239|conserved&px=1&section=5&ave_thresh=38&_ignoreionsscorebelow=20&report=0&_sigthreshold=0.001&_msresflags=1089&_msresflags2=2&percolate=0&percolate_rt=0))

**106 - 120 597.3328 1788.9765 1788.9734 2 0 K.VLLQQLQSQFSEITR.R**  ([Ions score 48](http://10.139.25.109/mascot/cgi/peptide_view.pl?file=../data/20120608/F008231.dat&query=8441&hit=1&index=orf239|conserved&px=1&section=5&ave_thresh=38&_ignoreionsscorebelow=20&report=0&_sigthreshold=0.001&_msresflags=1089&_msresflags2=2&percolate=0&percolate_rt=0))

**122 - 132 607.8281 1213.6416 1213.6415 0 0 R.VASQQQVLDAR.I**  ([Ions score 64](http://10.139.25.109/mascot/cgi/peptide_view.pl?file=../data/20120608/F008231.dat&query=3703&hit=1&index=orf239|conserved&px=1&section=5&ave_thresh=38&_ignoreionsscorebelow=20&report=0&_sigthreshold=0.001&_msresflags=1089&_msresflags2=2&percolate=0&percolate_rt=0))

**122 - 132 607.8290 1213.6435 1213.6415 2 0 R.VASQQQVLDAR.I**  ([Ions score 59](http://10.139.25.109/mascot/cgi/peptide_view.pl?file=../data/20120608/F008231.dat&query=3704&hit=1&index=orf239|conserved&px=1&section=5&ave_thresh=38&_ignoreionsscorebelow=20&report=0&_sigthreshold=0.001&_msresflags=1089&_msresflags2=2&percolate=0&percolate_rt=0))

**163 - 174 462.2700 1383.7882 1383.7874 1 0 R.YKPNLVDPIGIR.K**  ([Ions score 37](http://10.139.25.109/mascot/cgi/peptide_view.pl?file=../data/20120608/F008231.dat&query=5475&hit=1&index=orf239|conserved&px=1&section=5&ave_thresh=38&_ignoreionsscorebelow=20&report=0&_sigthreshold=0.001&_msresflags=1089&_msresflags2=2&percolate=0&percolate_rt=0))

**163 - 174 692.9020 1383.7894 1383.7874 1 0 R.YKPNLVDPIGIR.K**  ([Ions score 35](http://10.139.25.109/mascot/cgi/peptide_view.pl?file=../data/20120608/F008231.dat&query=5476&hit=1&index=orf239|conserved&px=1&section=5&ave_thresh=38&_ignoreionsscorebelow=20&report=0&_sigthreshold=0.001&_msresflags=1089&_msresflags2=2&percolate=0&percolate_rt=0))

**202 - 209 490.7503 979.4860 979.4862 0 0 R.TDIEFEVK.-**  ([Ions score 53](http://10.139.25.109/mascot/cgi/peptide_view.pl?file=../data/20120608/F008231.dat&query=1024&hit=1&index=orf239|conserved&px=1&section=5&ave_thresh=38&_ignoreionsscorebelow=20&report=0&_sigthreshold=0.001&_msresflags=1089&_msresflags2=2&percolate=0&percolate_rt=0))

5. [orf161|hypothetical](http://10.139.25.109/mascot/cgi/protein_view.pl?file=../data/20120608/F008231.dat&hit=orf161|hypothetical&db_idx=1&px=1&ave_thresh=38&_ignoreionsscorebelow=20&report=0&_sigthreshold=0.001&_msresflags=1089&_msresflags2=2&percolate=0&percolate_rt=0) protein|[vB_BceM_Bc431v3]   **Mass:** 17183   **Score:** 396   **emPAI:** 3.99

Sequence Coverage: **56%**; Matched peptides shown in **Bold Red**

**1** M**AKLADVLKQ ATVTLKVGDY LAAF**TRPEGY VPS**IKGTTSL HTGK**Y**LFKIV GVGTAEGDGS YKKMNIVPVV HK**ADQK**GYED KLKSPIVIVE PLK**VTHATGR

**101** NDNEYKDVYH GNSFHTHTVK AGEEGRSAL**L AFLEFTKTEY** SLTVNDFMVG GEELIAGA

**Start - End Observed Mr(expt) Mr(calc) ppm Miss Sequence**

**2 - 8 365.2292 728.4439 728.4432 1 0 M.AKLADVL.K**  ([Ions score 40](http://10.139.25.109/mascot/cgi/peptide_view.pl?file=../data/20120608/F008231.dat&query=63&hit=1&index=orf161|hypothetical&px=1&section=5&ave_thresh=38&_ignoreionsscorebelow=20&report=0&_sigthreshold=0.001&_msresflags=1089&_msresflags2=2&percolate=0&percolate_rt=0))

**9 - 15 380.7309 759.4472 759.4491 -2 0 L.KQATVTL.K**  ([Ions score 29](http://10.139.25.109/mascot/cgi/peptide_view.pl?file=../data/20120608/F008231.dat&query=144&hit=1&index=orf161|hypothetical&px=1&section=5&ave_thresh=38&_ignoreionsscorebelow=20&report=0&_sigthreshold=0.001&_msresflags=1089&_msresflags2=2&percolate=0&percolate_rt=0))

**10 - 16 380.7322 759.4499 759.4491 1 0 K.QATVTLK.V**  ([Ions score 50](http://10.139.25.109/mascot/cgi/peptide_view.pl?file=../data/20120608/F008231.dat&query=145&hit=1&index=orf161|hypothetical&px=1&section=5&ave_thresh=38&_ignoreionsscorebelow=20&report=0&_sigthreshold=0.001&_msresflags=1089&_msresflags2=2&percolate=0&percolate_rt=0))

**16 - 24 492.2660 982.5174 982.5124 5 0 L.KVGDYLAAF.T**  ([Ions score 32](http://10.139.25.109/mascot/cgi/peptide_view.pl?file=../data/20120608/F008231.dat&query=1035&hit=1&index=orf161|hypothetical&px=1&section=5&ave_thresh=38&_ignoreionsscorebelow=20&report=0&_sigthreshold=0.001&_msresflags=1089&_msresflags2=2&percolate=0&percolate_rt=0))

**34 - 44 571.8326 1141.6506 1141.6455 4 0 S.IKGTTSLHTGK.Y**  ([Ions score 24](http://10.139.25.109/mascot/cgi/peptide_view.pl?file=../data/20120608/F008231.dat&query=2782&hit=2&index=orf161|hypothetical&px=1&section=5&ave_thresh=38&_ignoreionsscorebelow=20&report=0&_sigthreshold=0.001&_msresflags=1089&_msresflags2=2&percolate=0&percolate_rt=0))

**46 - 61 806.9164 1611.8183 1611.8145 2 0 Y.LFKIVGVGTAEGDGSY.K**  ([Ions score 44](http://10.139.25.109/mascot/cgi/peptide_view.pl?file=../data/20120608/F008231.dat&query=7176&hit=1&index=orf161|hypothetical&px=1&section=5&ave_thresh=38&_ignoreionsscorebelow=20&report=0&_sigthreshold=0.001&_msresflags=1089&_msresflags2=2&percolate=0&percolate_rt=0))

**48 - 61 676.8378 1351.6610 1351.6620 -1 0 F.KIVGVGTAEGDGSY.K**  ([Ions score 82](http://10.139.25.109/mascot/cgi/peptide_view.pl?file=../data/20120608/F008231.dat&query=5190&hit=1&index=orf161|hypothetical&px=1&section=5&ave_thresh=38&_ignoreionsscorebelow=20&report=0&_sigthreshold=0.001&_msresflags=1089&_msresflags2=2&percolate=0&percolate_rt=0))

**48 - 61 676.8390 1351.6634 1351.6620 1 0 F.KIVGVGTAEGDGSY.K**  ([Ions score 76](http://10.139.25.109/mascot/cgi/peptide_view.pl?file=../data/20120608/F008231.dat&query=5192&hit=1&index=orf161|hypothetical&px=1&section=5&ave_thresh=38&_ignoreionsscorebelow=20&report=0&_sigthreshold=0.001&_msresflags=1089&_msresflags2=2&percolate=0&percolate_rt=0))

**49 - 62 676.8380 1351.6615 1351.6620 0 0 K.IVGVGTAEGDGSYK.K**  ([Ions score 57](http://10.139.25.109/mascot/cgi/peptide_view.pl?file=../data/20120608/F008231.dat&query=5191&hit=1&index=orf161|hypothetical&px=1&section=5&ave_thresh=38&_ignoreionsscorebelow=20&report=0&_sigthreshold=0.001&_msresflags=1089&_msresflags2=2&percolate=0&percolate_rt=0))

**49 - 62 676.8391 1351.6637 1351.6620 1 0 K.IVGVGTAEGDGSYK.K**  ([Ions score 67](http://10.139.25.109/mascot/cgi/peptide_view.pl?file=../data/20120608/F008231.dat&query=5193&hit=1&index=orf161|hypothetical&px=1&section=5&ave_thresh=38&_ignoreionsscorebelow=20&report=0&_sigthreshold=0.001&_msresflags=1089&_msresflags2=2&percolate=0&percolate_rt=0))

**49 - 63 494.2597 1479.7574 1479.7569 0 0 K.IVGVGTAEGDGSYKK.M**  ([Ions score 29](http://10.139.25.109/mascot/cgi/peptide_view.pl?file=../data/20120608/F008231.dat&query=6131&hit=1&index=orf161|hypothetical&px=1&section=5&ave_thresh=38&_ignoreionsscorebelow=20&report=0&_sigthreshold=0.001&_msresflags=1089&_msresflags2=2&percolate=0&percolate_rt=0))

**49 - 63 740.8869 1479.7592 1479.7569 2 0 K.IVGVGTAEGDGSYKK.M**  ([Ions score 48](http://10.139.25.109/mascot/cgi/peptide_view.pl?file=../data/20120608/F008231.dat&query=6132&hit=1&index=orf161|hypothetical&px=1&section=5&ave_thresh=38&_ignoreionsscorebelow=20&report=0&_sigthreshold=0.001&_msresflags=1089&_msresflags2=2&percolate=0&percolate_rt=0))

**49 - 63 740.8871 1479.7596 1479.7569 2 0 K.IVGVGTAEGDGSYKK.M**  ([Ions score 60](http://10.139.25.109/mascot/cgi/peptide_view.pl?file=../data/20120608/F008231.dat&query=6133&hit=1&index=orf161|hypothetical&px=1&section=5&ave_thresh=38&_ignoreionsscorebelow=20&report=0&_sigthreshold=0.001&_msresflags=1089&_msresflags2=2&percolate=0&percolate_rt=0))

**64 - 72 518.8022 1035.5898 1035.5899 0 0 K.MNIVPVVHK.A**  ([Ions score 41](http://10.139.25.109/mascot/cgi/peptide_view.pl?file=../data/20120608/F008231.dat&query=1535&hit=1&index=orf161|hypothetical&px=1&section=5&ave_thresh=38&_ignoreionsscorebelow=20&report=0&_sigthreshold=0.001&_msresflags=1089&_msresflags2=2&percolate=0&percolate_rt=0))

**64 - 72 518.8025 1035.5904 1035.5899 0 0 K.MNIVPVVHK.A**  ([Ions score 30](http://10.139.25.109/mascot/cgi/peptide_view.pl?file=../data/20120608/F008231.dat&query=1536&hit=1&index=orf161|hypothetical&px=1&section=5&ave_thresh=38&_ignoreionsscorebelow=20&report=0&_sigthreshold=0.001&_msresflags=1089&_msresflags2=2&percolate=0&percolate_rt=0))

**77 - 83 426.7270 851.4395 851.4389 1 0 K.GYEDKLK.S**  ([Ions score 21](http://10.139.25.109/mascot/cgi/peptide_view.pl?file=../data/20120608/F008231.dat&query=513&hit=1&index=orf161|hypothetical&px=1&section=5&ave_thresh=38&_ignoreionsscorebelow=20&report=0&_sigthreshold=0.001&_msresflags=1089&_msresflags2=2&percolate=0&percolate_rt=0))

**84 - 93 547.8447 1093.6749 1093.6747 0 0 K.SPIVIVEPLK.V**  ([Ions score 87](http://10.139.25.109/mascot/cgi/peptide_view.pl?file=../data/20120608/F008231.dat&query=2223&hit=1&index=orf161|hypothetical&px=1&section=5&ave_thresh=38&_ignoreionsscorebelow=20&report=0&_sigthreshold=0.001&_msresflags=1089&_msresflags2=2&percolate=0&percolate_rt=0))

**130 - 140 681.3538 1360.6931 1360.6915 1 0 L.LAFLEFTKTEY.S**  ([Ions score 61](http://10.139.25.109/mascot/cgi/peptide_view.pl?file=../data/20120608/F008231.dat&query=5279&hit=1&index=orf161|hypothetical&px=1&section=5&ave_thresh=38&_ignoreionsscorebelow=20&report=0&_sigthreshold=0.001&_msresflags=1089&_msresflags2=2&percolate=0&percolate_rt=0))

**130 - 140 681.3542 1360.6938 1360.6915 2 0 L.LAFLEFTKTEY.S**  ([Ions score 57](http://10.139.25.109/mascot/cgi/peptide_view.pl?file=../data/20120608/F008231.dat&query=5280&hit=1&index=orf161|hypothetical&px=1&section=5&ave_thresh=38&_ignoreionsscorebelow=20&report=0&_sigthreshold=0.001&_msresflags=1089&_msresflags2=2&percolate=0&percolate_rt=0))

**131 - 140 624.8109 1247.6073 1247.6074 0 0 L.AFLEFTKTEY.S**  ([Ions score 74](http://10.139.25.109/mascot/cgi/peptide_view.pl?file=../data/20120608/F008231.dat&query=4110&hit=1&index=orf161|hypothetical&px=1&section=5&ave_thresh=38&_ignoreionsscorebelow=20&report=0&_sigthreshold=0.001&_msresflags=1089&_msresflags2=2&percolate=0&percolate_rt=0))

**131 - 140 624.8118 1247.6090 1247.6074 1 0 L.AFLEFTKTEY.S**  ([Ions score 74](http://10.139.25.109/mascot/cgi/peptide_view.pl?file=../data/20120608/F008231.dat&query=4111&hit=1&index=orf161|hypothetical&px=1&section=5&ave_thresh=38&_ignoreionsscorebelow=20&report=0&_sigthreshold=0.001&_msresflags=1089&_msresflags2=2&percolate=0&percolate_rt=0))

6. [orf013|conserved](http://10.139.25.109/mascot/cgi/protein_view.pl?file=../data/20120608/F008231.dat&hit=orf013|conserved&db_idx=1&px=1&ave_thresh=38&_ignoreionsscorebelow=20&report=0&_sigthreshold=0.001&_msresflags=1089&_msresflags2=2&percolate=0&percolate_rt=0) hypothetical protein|[vB_BceM_Bc431v3]   **Mass:** 19414  **Score:** 341  **emPAI:** 3.19

Sequence Coverage: **66%**; Matched peptides shown in **Bold Red**

**1** **MDNNMPMIRN GVYQNNPDVL GKIGELDLAI KELGAGNTGD YATKEDIQGM VKQVNSTNPD TNGNVTLTGL VKKVNNTVPD ANGNVTITIP STAGMVKSVN**

**101 SNLPDANGNV TITI**PSTAGF VKKVNNVAPD ANGNVTINLF PSGTTAQRPS TGTVAGQYFF DTTLNKPLYR NATN**NGWVDG TGAP**VT

**Start - End Observed Mr(expt) Mr(calc) ppm Miss Sequence**

**1 - 9 561.2483 1120.4821 1120.4827 -1 0 -.MDNNMPMIR.N**  ([Ions score 57](http://10.139.25.109/mascot/cgi/peptide_view.pl?file=../data/20120608/F008231.dat&query=2493&hit=1&index=orf013|conserved&px=1&section=5&ave_thresh=38&_ignoreionsscorebelow=20&report=0&_sigthreshold=0.001&_msresflags=1089&_msresflags2=2&percolate=0&percolate_rt=0))

**10 - 22 709.3580 1416.7014 1416.6997 1 0 R.NGVYQNNPDVLGK.I**  ([Ions score 53](http://10.139.25.109/mascot/cgi/peptide_view.pl?file=../data/20120608/F008231.dat&query=5685&hit=1&index=orf013|conserved&px=1&section=5&ave_thresh=38&_ignoreionsscorebelow=20&report=0&_sigthreshold=0.001&_msresflags=1089&_msresflags2=2&percolate=0&percolate_rt=0))

**10 - 22 709.3582 1416.7017 1416.6997 1 0 R.NGVYQNNPDVLGK.I**  ([Ions score 68](http://10.139.25.109/mascot/cgi/peptide_view.pl?file=../data/20120608/F008231.dat&query=5686&hit=1&index=orf013|conserved&px=1&section=5&ave_thresh=38&_ignoreionsscorebelow=20&report=0&_sigthreshold=0.001&_msresflags=1089&_msresflags2=2&percolate=0&percolate_rt=0))

**10 - 22 709.8458 1417.6771 1417.6837 -5 0 R.NGVYQNNPDVLGK.I**  Deamidated (NQ) ([Ions score 68](http://10.139.25.109/mascot/cgi/peptide_view.pl?file=../data/20120608/F008231.dat&query=5699&hit=1&index=orf013|conserved&px=1&section=5&ave_thresh=38&_ignoreionsscorebelow=20&report=0&_sigthreshold=0.001&_msresflags=1089&_msresflags2=2&percolate=0&percolate_rt=0))

**10 - 22 709.8471 1417.6797 1417.6837 -3 0 R.NGVYQNNPDVLGK.I**  Deamidated (NQ) ([Ions score 45](http://10.139.25.109/mascot/cgi/peptide_view.pl?file=../data/20120608/F008231.dat&query=5700&hit=1&index=orf013|conserved&px=1&section=5&ave_thresh=38&_ignoreionsscorebelow=20&report=0&_sigthreshold=0.001&_msresflags=1089&_msresflags2=2&percolate=0&percolate_rt=0))

**23 - 31 486.2918 970.5691 970.5699 -1 0 K.IGELDLAIK.E**  ([Ions score 78](http://10.139.25.109/mascot/cgi/peptide_view.pl?file=../data/20120608/F008231.dat&query=984&hit=1&index=orf013|conserved&px=1&section=5&ave_thresh=38&_ignoreionsscorebelow=20&report=0&_sigthreshold=0.001&_msresflags=1089&_msresflags2=2&percolate=0&percolate_rt=0))

**32 - 52 733.0199 2196.0379 2196.0369 0 0 K.ELGAGNTGDYATKEDIQGMVK.Q**  ([Ions score 54](http://10.139.25.109/mascot/cgi/peptide_view.pl?file=../data/20120608/F008231.dat&query=10620&hit=1&index=orf013|conserved&px=1&section=5&ave_thresh=38&_ignoreionsscorebelow=20&report=0&_sigthreshold=0.001&_msresflags=1089&_msresflags2=2&percolate=0&percolate_rt=0))

**53 - 72 1037.0330 2072.0514 2072.0386 6 0 K.QVNSTNPDTNGNVTLTGLVK.K**  Deamidated (NQ) ([Ions score 95](http://10.139.25.109/mascot/cgi/peptide_view.pl?file=../data/20120608/F008231.dat&query=10003&hit=1&index=orf013|conserved&px=1&section=5&ave_thresh=38&_ignoreionsscorebelow=20&report=0&_sigthreshold=0.001&_msresflags=1089&_msresflags2=2&percolate=0&percolate_rt=0))

**53 - 72 691.6930 2072.0573 2072.0386 9 0 K.QVNSTNPDTNGNVTLTGLVK.K**  Deamidated (NQ) ([Ions score 45](http://10.139.25.109/mascot/cgi/peptide_view.pl?file=../data/20120608/F008231.dat&query=10004&hit=1&index=orf013|conserved&px=1&section=5&ave_thresh=38&_ignoreionsscorebelow=20&report=0&_sigthreshold=0.001&_msresflags=1089&_msresflags2=2&percolate=0&percolate_rt=0))

**53 - 72 1036.5348 2071.0539 2071.0546 0 0 K.QVNSTNPDTNGNVTLTGLVK.K**  ([Ions score 112](http://10.139.25.109/mascot/cgi/peptide_view.pl?file=../data/20120608/F008231.dat&query=10005&hit=1&index=orf013|conserved&px=1&section=5&ave_thresh=38&_ignoreionsscorebelow=20&report=0&_sigthreshold=0.001&_msresflags=1089&_msresflags2=2&percolate=0&percolate_rt=0))

**53 - 73 734.3917 2200.1533 2200.1335 9 0 K.QVNSTNPDTNGNVTLTGLVKK.V**  Deamidated (NQ) ([Ions score 42](http://10.139.25.109/mascot/cgi/peptide_view.pl?file=../data/20120608/F008231.dat&query=10638&hit=1&index=orf013|conserved&px=1&section=5&ave_thresh=38&_ignoreionsscorebelow=20&report=0&_sigthreshold=0.001&_msresflags=1089&_msresflags2=2&percolate=0&percolate_rt=0))

**53 - 73 734.3919 2200.1539 2200.1335 9 0 K.QVNSTNPDTNGNVTLTGLVKK.V**  Deamidated (NQ) ([Ions score 50](http://10.139.25.109/mascot/cgi/peptide_view.pl?file=../data/20120608/F008231.dat&query=10639&hit=1&index=orf013|conserved&px=1&section=5&ave_thresh=38&_ignoreionsscorebelow=20&report=0&_sigthreshold=0.001&_msresflags=1089&_msresflags2=2&percolate=0&percolate_rt=0))

**72 - 92 729.0486 2184.1239 2184.1274 -2 0 V.KKVNNTVPDANGNVTITIPST.A**  2 Deamidated (NQ) ([Ions score 21](http://10.139.25.109/mascot/cgi/peptide_view.pl?file=../data/20120608/F008231.dat&query=10574&hit=1&index=orf013|conserved&px=1&section=5&ave_thresh=38&_ignoreionsscorebelow=20&report=0&_sigthreshold=0.001&_msresflags=1089&_msresflags2=2&percolate=0&percolate_rt=0))

**80 - 90 557.7902 1113.5658 1113.5666 -1 0 P.DANGNVTITIP.S**  ([Ions score 27](http://10.139.25.109/mascot/cgi/peptide_view.pl?file=../data/20120608/F008231.dat&query=2417&hit=1&index=orf013|conserved&px=1&section=5&ave_thresh=38&_ignoreionsscorebelow=20&report=0&_sigthreshold=0.001&_msresflags=1089&_msresflags2=2&percolate=0&percolate_rt=0))

**87 - 114 712.3614 2845.4167 2845.4267 -3 0 T.ITIPSTAGMVKSVNSNLPDANGNVTITI.P** Oxidation (M)([Ions score 21](http://10.139.25.109/mascot/cgi/peptide_view.pl?file=../data/20120608/F008231.dat&query=11853&hit=1&index=orf013|conserved&px=1&section=5&ave_thresh=38&_ignoreionsscorebelow=20&report=0&_sigthreshold=0.001&_msresflags=1089&_msresflags2=2&percolate=0&percolate_rt=0))

**175 - 184 487.7129 973.4112 973.4141 -3 0 N.NGWVDGTGAP.V**  Deamidated (NQ) ([Ions score 31](http://10.139.25.109/mascot/cgi/peptide_view.pl?file=../data/20120608/F008231.dat&query=997&hit=1&index=orf013|conserved&px=1&section=5&ave_thresh=38&_ignoreionsscorebelow=20&report=0&_sigthreshold=0.001&_msresflags=1089&_msresflags2=2&percolate=0&percolate_rt=0))

**175 - 184 487.7130 973.4114 973.4141 -3 0 N.NGWVDGTGAP.V**  Deamidated (NQ) ([Ions score 35](http://10.139.25.109/mascot/cgi/peptide_view.pl?file=../data/20120608/F008231.dat&query=998&hit=1&index=orf013|conserved&px=1&section=5&ave_thresh=38&_ignoreionsscorebelow=20&report=0&_sigthreshold=0.001&_msresflags=1089&_msresflags2=2&percolate=0&percolate_rt=0))

7. [orf232|Major](http://10.139.25.109/mascot/cgi/protein_view.pl?file=../data/20120608/F008231.dat&hit=orf232|Major&db_idx=1&px=1&ave_thresh=38&_ignoreionsscorebelow=20&report=0&_sigthreshold=0.001&_msresflags=1089&_msresflags2=2&percolate=0&percolate_rt=0) capsid precursor; putative membrane protein|[vB_BceM_Bc431v3] **Mass:** 52511 **Score:** 340 **emPAI:** 0.83

Sequence Coverage: **50%**; Matched peptides shown in **Bold Red**

**1** MGAELNKDKQ VQAEPEVRKL PQAAEDKIAD LQK**SFTTGVG ITPDTQLDAA ALRREYLEDE VKMLTWDNSD FTIYPLIAKQ QISNTVAKYA VFNQHGR**TGH

**101** SR**FVSEIGVA SINDPNIRQK TVQM**KFISDT K**QQSIAAGLV NNISDPMTIL TEDAISVIAK SIE**WAIFYGD ASLSAESDQQ SGIEFDGLH**K LIDQKTNIID**

**201 LKGQSLSEAV LNK**AAVIVGK GYGKATDAFM PIGVQAEF**TN NLLDRQRVIQ PSNAGGFSTG FTINQFLSAR GAINLHGSTI MENDNVLVEN RLPQANAPLP**

**301 VK**TLKATVK**A ADKGGFTTED K**SLSYKVVVF SNEAESVASD AVTAALTDAT SSVTLEIELQ PIYQAQPQFV VVYRQGAQTG HYFQIARIPV AK**ASDLNVIT**

**401 FVDRNEIIPE TTDVF**VGEMN QNVLSLLELM PMMRLPLAQM NATYTFSVLW YGALALYAPK KWVRIKNVKY IPALAADVTL

**Start - End Observed Mr(expt) Mr(calc) ppm Miss Sequence**

**34 - 53 679.0216 2034.0430 2034.0270 8 0 K.SFTTGVGITPDTQLDAAALR.R**  Deamidated (NQ) ([Ions score 39](http://10.139.25.109/mascot/cgi/peptide_view.pl?file=../data/20120608/F008231.dat&query=9768&hit=1&index=orf232|Major&px=1&section=5&ave_thresh=38&_ignoreionsscorebelow=20&report=0&_sigthreshold=0.001&_msresflags=1089&_msresflags2=2&percolate=0&percolate_rt=0))

**34 - 54 731.0571 2190.1494 2190.1281 10 0 K.SFTTGVGITPDTQLDAAALRR.E**  Deamidated (NQ) ([Ions score 38](http://10.139.25.109/mascot/cgi/peptide_view.pl?file=../data/20120608/F008231.dat&query=10597&hit=1&index=orf232|Major&px=1&section=5&ave_thresh=38&_ignoreionsscorebelow=20&report=0&_sigthreshold=0.001&_msresflags=1089&_msresflags2=2&percolate=0&percolate_rt=0))

**34 - 54 731.0571 2190.1494 2190.1281 10 0 K.SFTTGVGITPDTQLDAAALRR.E**  Deamidated (NQ) ([Ions score 24](http://10.139.25.109/mascot/cgi/peptide_view.pl?file=../data/20120608/F008231.dat&query=10598&hit=1&index=orf232|Major&px=1&section=5&ave_thresh=38&_ignoreionsscorebelow=20&report=0&_sigthreshold=0.001&_msresflags=1089&_msresflags2=2&percolate=0&percolate_rt=0))

**55 - 62 512.7448 1023.4751 1023.4760 -1 0 R.EYLEDEVK.M**  ([Ions score 28](http://10.139.25.109/mascot/cgi/peptide_view.pl?file=../data/20120608/F008231.dat&query=1408&hit=1&index=orf232|Major&px=1&section=5&ave_thresh=38&_ignoreionsscorebelow=20&report=0&_sigthreshold=0.001&_msresflags=1089&_msresflags2=2&percolate=0&percolate_rt=0))

**55 - 62 512.7458 1023.4770 1023.4760 1 0 R.EYLEDEVK.M**  ([Ions score 23](http://10.139.25.109/mascot/cgi/peptide_view.pl?file=../data/20120608/F008231.dat&query=1409&hit=1&index=orf232|Major&px=1&section=5&ave_thresh=38&_ignoreionsscorebelow=20&report=0&_sigthreshold=0.001&_msresflags=1089&_msresflags2=2&percolate=0&percolate_rt=0))

**63 - 79 1014.5114 2027.0082 2027.0074 0 0 K.MLTWDNSDFTIYPLIAK.Q**  ([Ions score 73](http://10.139.25.109/mascot/cgi/peptide_view.pl?file=../data/20120608/F008231.dat&query=9707&hit=1&index=orf232|Major&px=1&section=5&ave_thresh=38&_ignoreionsscorebelow=20&report=0&_sigthreshold=0.001&_msresflags=1089&_msresflags2=2&percolate=0&percolate_rt=0))

**80 - 88 494.7748 987.5351 987.5349 0 0 K.QQISNTVAK.Y**  ([Ions score 28](http://10.139.25.109/mascot/cgi/peptide_view.pl?file=../data/20120608/F008231.dat&query=1062&hit=1&index=orf232|Major&px=1&section=5&ave_thresh=38&_ignoreionsscorebelow=20&report=0&_sigthreshold=0.001&_msresflags=1089&_msresflags2=2&percolate=0&percolate_rt=0))

**89 - 97 364.5179 1090.5318 1090.5308 1 0 K.YAVFNQHGR.T**  ([Ions score 23](http://10.139.25.109/mascot/cgi/peptide_view.pl?file=../data/20120608/F008231.dat&query=2176&hit=1&index=orf232|Major&px=1&section=5&ave_thresh=38&_ignoreionsscorebelow=20&report=0&_sigthreshold=0.001&_msresflags=1089&_msresflags2=2&percolate=0&percolate_rt=0))

**103 - 118 577.6405 1729.8997 1729.8999 0 0 R.FVSEIGVASINDPNIR.Q**  ([Ions score 42](http://10.139.25.109/mascot/cgi/peptide_view.pl?file=../data/20120608/F008231.dat&query=8053&hit=1&index=orf232|Major&px=1&section=5&ave_thresh=38&_ignoreionsscorebelow=20&report=0&_sigthreshold=0.001&_msresflags=1089&_msresflags2=2&percolate=0&percolate_rt=0))

**103 - 118 865.9574 1729.9002 1729.8999 0 0 R.FVSEIGVASINDPNIR.Q**  ([Ions score 56](http://10.139.25.109/mascot/cgi/peptide_view.pl?file=../data/20120608/F008231.dat&query=8054&hit=1&index=orf232|Major&px=1&section=5&ave_thresh=38&_ignoreionsscorebelow=20&report=0&_sigthreshold=0.001&_msresflags=1089&_msresflags2=2&percolate=0&percolate_rt=0))

**103 - 118 865.9591 1729.9035 1729.8999 2 0 R.FVSEIGVASINDPNIR.Q**  ([Ions score 97](http://10.139.25.109/mascot/cgi/peptide_view.pl?file=../data/20120608/F008231.dat&query=8055&hit=1&index=orf232|Major&px=1&section=5&ave_thresh=38&_ignoreionsscorebelow=20&report=0&_sigthreshold=0.001&_msresflags=1089&_msresflags2=2&percolate=0&percolate_rt=0))

**104 - 124 767.4085 2299.2037 2299.1842 8 0 F.VSEIGVASINDPNIRQKTVQM.K**  Deamidated (NQ) ([Ions score 41](http://10.139.25.109/mascot/cgi/peptide_view.pl?file=../data/20120608/F008231.dat&query=10969&hit=1&index=orf232|Major&px=1&section=5&ave_thresh=38&_ignoreionsscorebelow=20&report=0&_sigthreshold=0.001&_msresflags=1089&_msresflags2=2&percolate=0&percolate_rt=0))

**104 - 124 767.4089 2299.2050 2299.1842 9 0 F.VSEIGVASINDPNIRQKTVQM.K**  Deamidated (NQ) ([Ions score 49](http://10.139.25.109/mascot/cgi/peptide_view.pl?file=../data/20120608/F008231.dat&query=10970&hit=1&index=orf232|Major&px=1&section=5&ave_thresh=38&_ignoreionsscorebelow=20&report=0&_sigthreshold=0.001&_msresflags=1089&_msresflags2=2&percolate=0&percolate_rt=0))

**116 - 124 567.8028 1133.5910 1133.5863 4 0 P.NIRQKTVQM.K**  Deamidated (NQ); Oxidation (M)([Ions score 23](http://10.139.25.109/mascot/cgi/peptide_view.pl?file=../data/20120608/F008231.dat&query=2668&hit=1&index=orf232|Major&px=1&section=5&ave_thresh=38&_ignoreionsscorebelow=20&report=0&_sigthreshold=0.001&_msresflags=1089&_msresflags2=2&percolate=0&percolate_rt=0))

**132 - 140 443.7550 885.4954 885.4920 4 0 K.QQSIAAGLV.N**  ([Ions score 23](http://10.139.25.109/mascot/cgi/peptide_view.pl?file=../data/20120608/F008231.dat&query=640&hit=1&index=orf232|Major&px=1&section=5&ave_thresh=38&_ignoreionsscorebelow=20&report=0&_sigthreshold=0.001&_msresflags=1089&_msresflags2=2&percolate=0&percolate_rt=0))

**140 - 163 859.1078 2574.3015 2574.2986 1 0 L.VNNISDPMTILTEDAISVIAKSIE.W** 2Deamidated (NQ)([Ions score 22](http://10.139.25.109/mascot/cgi/peptide_view.pl?file=../data/20120608/F008231.dat&query=11552&hit=1&index=orf232|Major&px=1&section=5&ave_thresh=38&_ignoreionsscorebelow=20&report=0&_sigthreshold=0.001&_msresflags=1089&_msresflags2=2&percolate=0&percolate_rt=0))

**190 - 198 536.8239 1071.6333 1071.6288 4 0 H.KLIDQKTNI.I**  ([Ions score 21](http://10.139.25.109/mascot/cgi/peptide_view.pl?file=../data/20120608/F008231.dat&query=1920&hit=2&index=orf232|Major&px=1&section=5&ave_thresh=38&_ignoreionsscorebelow=20&report=0&_sigthreshold=0.001&_msresflags=1089&_msresflags2=2&percolate=0&percolate_rt=0))

**196 - 202 408.7454 815.4763 815.4752 1 0 K.TNIIDLK.G**  ([Ions score 45](http://10.139.25.109/mascot/cgi/peptide_view.pl?file=../data/20120608/F008231.dat&query=350&hit=1&index=orf232|Major&px=1&section=5&ave_thresh=38&_ignoreionsscorebelow=20&report=0&_sigthreshold=0.001&_msresflags=1089&_msresflags2=2&percolate=0&percolate_rt=0))

**203 - 213 573.3118 1144.6090 1144.6088 0 0 K.GQSLSEAVLNK.A**  ([Ions score 64](http://10.139.25.109/mascot/cgi/peptide_view.pl?file=../data/20120608/F008231.dat&query=2821&hit=1&index=orf232|Major&px=1&section=5&ave_thresh=38&_ignoreionsscorebelow=20&report=0&_sigthreshold=0.001&_msresflags=1089&_msresflags2=2&percolate=0&percolate_rt=0))

**203 - 213 573.3120 1144.6095 1144.6088 1 0 K.GQSLSEAVLNK.A**  ([Ions score 56](http://10.139.25.109/mascot/cgi/peptide_view.pl?file=../data/20120608/F008231.dat&query=2822&hit=1&index=orf232|Major&px=1&section=5&ave_thresh=38&_ignoreionsscorebelow=20&report=0&_sigthreshold=0.001&_msresflags=1089&_msresflags2=2&percolate=0&percolate_rt=0))

**239 - 257 701.0373 2100.0900 2100.0712 9 0 F.TNNLLDRQRVIQPSNAGGF.S**  Deamidated (NQ) ([Ions score 35](http://10.139.25.109/mascot/cgi/peptide_view.pl?file=../data/20120608/F008231.dat&query=10164&hit=1&index=orf232|Major&px=1&section=5&ave_thresh=38&_ignoreionsscorebelow=20&report=0&_sigthreshold=0.001&_msresflags=1089&_msresflags2=2&percolate=0&percolate_rt=0))

**239 - 261 831.7598 2492.2575 2492.2408 7 0 F.TNNLLDRQRVIQPSNAGGFSTGF.T**  Deamidated (NQ) ([Ions score 53](http://10.139.25.109/mascot/cgi/peptide_view.pl?file=../data/20120608/F008231.dat&query=11410&hit=1&index=orf232|Major&px=1&section=5&ave_thresh=38&_ignoreionsscorebelow=20&report=0&_sigthreshold=0.001&_msresflags=1089&_msresflags2=2&percolate=0&percolate_rt=0))

**248 - 270 805.0807 2412.2202 2412.2074 5 0 R.VIQPSNAGGFSTGFTINQFLSAR.G**  Deamidated (NQ) ([Ions score 41](http://10.139.25.109/mascot/cgi/peptide_view.pl?file=../data/20120608/F008231.dat&query=11275&hit=1&index=orf232|Major&px=1&section=5&ave_thresh=38&_ignoreionsscorebelow=20&report=0&_sigthreshold=0.001&_msresflags=1089&_msresflags2=2&percolate=0&percolate_rt=0))

**271 - 291 766.3843 2296.1312 2296.1117 8 0 R.GAINLHGSTIMENDNVLVENR.L**  Deamidated (NQ) ([Ions score 25](http://10.139.25.109/mascot/cgi/peptide_view.pl?file=../data/20120608/F008231.dat&query=10954&hit=1&index=orf232|Major&px=1&section=5&ave_thresh=38&_ignoreionsscorebelow=20&report=0&_sigthreshold=0.001&_msresflags=1089&_msresflags2=2&percolate=0&percolate_rt=0))

**273 - 281 501.2611 1000.5077 1000.5012 7 0 A.INLHGSTIM.E**  Oxidation (M) ([Ions score 22](http://10.139.25.109/mascot/cgi/peptide_view.pl?file=../data/20120608/F008231.dat&query=1145&hit=1&index=orf232|Major&px=1&section=5&ave_thresh=38&_ignoreionsscorebelow=20&report=0&_sigthreshold=0.001&_msresflags=1089&_msresflags2=2&percolate=0&percolate_rt=0))

**283 - 300 659.0161 1974.0265 1974.0170 5 0 E.NDNVLVENRLPQANAPLP.V**  Deamidated (NQ) ([Ions score 30](http://10.139.25.109/mascot/cgi/peptide_view.pl?file=../data/20120608/F008231.dat&query=9443&hit=1&index=orf232|Major&px=1&section=5&ave_thresh=38&_ignoreionsscorebelow=20&report=0&_sigthreshold=0.001&_msresflags=1089&_msresflags2=2&percolate=0&percolate_rt=0))

**292 - 302 574.3454 1146.6762 1146.6761 0 0 R.LPQANAPLPVK.T**  ([Ions score 37](http://10.139.25.109/mascot/cgi/peptide_view.pl?file=../data/20120608/F008231.dat&query=2844&hit=1&index=orf232|Major&px=1&section=5&ave_thresh=38&_ignoreionsscorebelow=20&report=0&_sigthreshold=0.001&_msresflags=1089&_msresflags2=2&percolate=0&percolate_rt=0))

**292 - 302 574.3456 1146.6767 1146.6761 1 0 R.LPQANAPLPVK.T**  ([Ions score 45](http://10.139.25.109/mascot/cgi/peptide_view.pl?file=../data/20120608/F008231.dat&query=2845&hit=1&index=orf232|Major&px=1&section=5&ave_thresh=38&_ignoreionsscorebelow=20&report=0&_sigthreshold=0.001&_msresflags=1089&_msresflags2=2&percolate=0&percolate_rt=0))

**310 - 321 413.8669 1238.5790 1238.5779 1 0 K.AADKGGFTTEDK.S**  ([Ions score 28](http://10.139.25.109/mascot/cgi/peptide_view.pl?file=../data/20120608/F008231.dat&query=4013&hit=1&index=orf232|Major&px=1&section=5&ave_thresh=38&_ignoreionsscorebelow=20&report=0&_sigthreshold=0.001&_msresflags=1089&_msresflags2=2&percolate=0&percolate_rt=0))

**393 - 404 675.3574 1348.7002 1348.6987 1 0 K.ASDLNVITFVDR.N**  ([Ions score 82](http://10.139.25.109/mascot/cgi/peptide_view.pl?file=../data/20120608/F008231.dat&query=5178&hit=1&index=orf232|Major&px=1&section=5&ave_thresh=38&_ignoreionsscorebelow=20&report=0&_sigthreshold=0.001&_msresflags=1089&_msresflags2=2&percolate=0&percolate_rt=0))

**393 - 404 675.3583 1348.7020 1348.6987 2 0 K.ASDLNVITFVDR.N**  ([Ions score 89](http://10.139.25.109/mascot/cgi/peptide_view.pl?file=../data/20120608/F008231.dat&query=5179&hit=1&index=orf232|Major&px=1&section=5&ave_thresh=38&_ignoreionsscorebelow=20&report=0&_sigthreshold=0.001&_msresflags=1089&_msresflags2=2&percolate=0&percolate_rt=0))

**402 - 415 824.4141 1646.8136 1646.8152 -1 0 F.VDRNEIIPETTDVF.V**  ([Ions score 27](http://10.139.25.109/mascot/cgi/peptide_view.pl?file=../data/20120608/F008231.dat&query=7435&hit=1&index=orf232|Major&px=1&section=5&ave_thresh=38&_ignoreionsscorebelow=20&report=0&_sigthreshold=0.001&_msresflags=1089&_msresflags2=2&percolate=0&percolate_rt=0))

8. [orf158|conserved](http://10.139.25.109/mascot/cgi/protein_view.pl?file=../data/20120608/F008231.dat&hit=orf158|conserved&db_idx=1&px=1&ave_thresh=38&_ignoreionsscorebelow=20&report=0&_sigthreshold=0.001&_msresflags=1089&_msresflags2=2&percolate=0&percolate_rt=0) hypothetical protein|[vB_BceM_Bc431v3] **Mass:** 49386 **Score:** 305  **emPAI:** 1.03

Sequence Coverage: **30%**; Matched peptides shown in **Bold Red**

**1** M**AKEKLTVQE QELIKQETGA Y**VVVMGYLKR ER**GQVAPSVF NKIIVELGYD KVKKNDLIAV AQKIEKNDVL ASLYKKSYLG EITIDDIPHA YEEESEVKEE**

**101 N**IFSYVTNYL MDKEDNTAKL REYRKLQKDG TLMALLMKDL KKHLVEELKG LPRAKYLTSA PYTPETGDKT LI**LALSDWHV G**FISHDMHTG DYNFERLQTS

**201** IQEIVSYTIR TVQERDIKEV HVLFLGDLVE NFAMRSTQSF DLEFTFAEQ**I AKGQQL**LIDV LLTLSKFVPV TFSMVAGNHD RFETDKKTAI FNNSVAYTVL

**301** EN**LIMLQEKM GQLPN**VTITD NRKDVYRFDV DIAGQGIAGA HGDHLAKSSE KIPAFMTHGR KVDILFTGHL HNFRIAQESF TRLHLQVGST IGENSYSR**QG**

**401 NYPTTTPSQQ IV**ILTEGSKI PELIPLWLGT DGKLL

**Start - End Observed Mr(expt) Mr(calc) ppm Miss Sequence**

**2 - 21 769.7481 2306.2223 2306.2005 9 0 M.AKEKLTVQEQELIKQETGAY.V**  Deamidated (NQ) ([Ions score 29](http://10.139.25.109/mascot/cgi/peptide_view.pl?file=../data/20120608/F008231.dat&query=10993&hit=1&index=orf158|conserved&px=1&section=5&ave_thresh=38&_ignoreionsscorebelow=20&report=0&_sigthreshold=0.001&_msresflags=1089&_msresflags2=2&percolate=0&percolate_rt=0))

**6 - 15 600.8458 1199.6770 1199.6761 1 0 K.LTVQEQELIK.Q**  ([Ions score 67](http://10.139.25.109/mascot/cgi/peptide_view.pl?file=../data/20120608/F008231.dat&query=3556&hit=1&index=orf158|conserved&px=1&section=5&ave_thresh=38&_ignoreionsscorebelow=20&report=0&_sigthreshold=0.001&_msresflags=1089&_msresflags2=2&percolate=0&percolate_rt=0))

**6 - 15 600.8471 1199.6797 1199.6761 3 0 K.LTVQEQELIK.Q**  ([Ions score 63](http://10.139.25.109/mascot/cgi/peptide_view.pl?file=../data/20120608/F008231.dat&query=3557&hit=1&index=orf158|conserved&px=1&section=5&ave_thresh=38&_ignoreionsscorebelow=20&report=0&_sigthreshold=0.001&_msresflags=1089&_msresflags2=2&percolate=0&percolate_rt=0))

**33 - 42 523.7853 1045.5560 1045.5556 0 0 R.GQVAPSVFNK.I**  ([Ions score 49](http://10.139.25.109/mascot/cgi/peptide_view.pl?file=../data/20120608/F008231.dat&query=1635&hit=1&index=orf158|conserved&px=1&section=5&ave_thresh=38&_ignoreionsscorebelow=20&report=0&_sigthreshold=0.001&_msresflags=1089&_msresflags2=2&percolate=0&percolate_rt=0))

**33 - 42 523.7853 1045.5560 1045.5556 0 0 R.GQVAPSVFNK.I**  ([Ions score 47](http://10.139.25.109/mascot/cgi/peptide_view.pl?file=../data/20120608/F008231.dat&query=1636&hit=1&index=orf158|conserved&px=1&section=5&ave_thresh=38&_ignoreionsscorebelow=20&report=0&_sigthreshold=0.001&_msresflags=1089&_msresflags2=2&percolate=0&percolate_rt=0))

**43 - 51 525.2970 1048.5794 1048.5804 -1 0 K.IIVELGYDK.V**  ([Ions score 46](http://10.139.25.109/mascot/cgi/peptide_view.pl?file=../data/20120608/F008231.dat&query=1679&hit=1&index=orf158|conserved&px=1&section=5&ave_thresh=38&_ignoreionsscorebelow=20&report=0&_sigthreshold=0.001&_msresflags=1089&_msresflags2=2&percolate=0&percolate_rt=0))

**43 - 51 525.2991 1048.5836 1048.5804 3 0 K.IIVELGYDK.V**  ([Ions score 59](http://10.139.25.109/mascot/cgi/peptide_view.pl?file=../data/20120608/F008231.dat&query=1680&hit=1&index=orf158|conserved&px=1&section=5&ave_thresh=38&_ignoreionsscorebelow=20&report=0&_sigthreshold=0.001&_msresflags=1089&_msresflags2=2&percolate=0&percolate_rt=0))

**43 - 53 426.2551 1275.7434 1275.7438 -0 0 K.IIVELGYDKVK.K**  ([Ions score 41](http://10.139.25.109/mascot/cgi/peptide_view.pl?file=../data/20120608/F008231.dat&query=4435&hit=1&index=orf158|conserved&px=1&section=5&ave_thresh=38&_ignoreionsscorebelow=20&report=0&_sigthreshold=0.001&_msresflags=1089&_msresflags2=2&percolate=0&percolate_rt=0))

**50 - 58 536.8259 1071.6373 1071.6288 8 0 Y.DKVKKNDLI.A**  ([Ions score 21](http://10.139.25.109/mascot/cgi/peptide_view.pl?file=../data/20120608/F008231.dat&query=1921&hit=2&index=orf158|conserved&px=1&section=5&ave_thresh=38&_ignoreionsscorebelow=20&report=0&_sigthreshold=0.001&_msresflags=1089&_msresflags2=2&percolate=0&percolate_rt=0))

**54 - 63 550.3269 1098.6392 1098.6397 -0 0 K.KNDLIAVAQK.I**  ([Ions score 64](http://10.139.25.109/mascot/cgi/peptide_view.pl?file=../data/20120608/F008231.dat&query=2278&hit=1&index=orf158|conserved&px=1&section=5&ave_thresh=38&_ignoreionsscorebelow=20&report=0&_sigthreshold=0.001&_msresflags=1089&_msresflags2=2&percolate=0&percolate_rt=0))

**54 - 63 367.2210 1098.6413 1098.6397 1 0 K.KNDLIAVAQK.I**  ([Ions score 25](http://10.139.25.109/mascot/cgi/peptide_view.pl?file=../data/20120608/F008231.dat&query=2279&hit=1&index=orf158|conserved&px=1&section=5&ave_thresh=38&_ignoreionsscorebelow=20&report=0&_sigthreshold=0.001&_msresflags=1089&_msresflags2=2&percolate=0&percolate_rt=0))

**55 - 71 928.4951 1854.9756 1854.9938 -10 0 K.NDLIAVAQKIEKNDVLA.S**  2 Deamidated (NQ) ([Ions score 20](http://10.139.25.109/mascot/cgi/peptide_view.pl?file=../data/20120608/F008231.dat&query=8861&hit=1&index=orf158|conserved&px=1&section=5&ave_thresh=38&_ignoreionsscorebelow=20&report=0&_sigthreshold=0.001&_msresflags=1089&_msresflags2=2&percolate=0&percolate_rt=0))

**63 - 74 696.8909 1391.7673 1391.7660 1 0 Q.KIEKNDVLASLY.K**  ([Ions score 49](http://10.139.25.109/mascot/cgi/peptide_view.pl?file=../data/20120608/F008231.dat&query=5528&hit=1&index=orf158|conserved&px=1&section=5&ave_thresh=38&_ignoreionsscorebelow=20&report=0&_sigthreshold=0.001&_msresflags=1089&_msresflags2=2&percolate=0&percolate_rt=0))

**63 - 74 696.8917 1391.7688 1391.7660 2 0 Q.KIEKNDVLASLY.K**  ([Ions score 38](http://10.139.25.109/mascot/cgi/peptide_view.pl?file=../data/20120608/F008231.dat&query=5530&hit=1&index=orf158|conserved&px=1&section=5&ave_thresh=38&_ignoreionsscorebelow=20&report=0&_sigthreshold=0.001&_msresflags=1089&_msresflags2=2&percolate=0&percolate_rt=0))

**64 - 75 464.9301 1391.7684 1391.7660 2 0 K.IEKNDVLASLYK.K**  ([Ions score 31](http://10.139.25.109/mascot/cgi/peptide_view.pl?file=../data/20120608/F008231.dat&query=5529&hit=1&index=orf158|conserved&px=1&section=5&ave_thresh=38&_ignoreionsscorebelow=20&report=0&_sigthreshold=0.001&_msresflags=1089&_msresflags2=2&percolate=0&percolate_rt=0))

**67 - 75 511.7797 1021.5449 1021.5444 0 0 K.NDVLASLYK.K**  ([Ions score 33](http://10.139.25.109/mascot/cgi/peptide_view.pl?file=../data/20120608/F008231.dat&query=1392&hit=1&index=orf158|conserved&px=1&section=5&ave_thresh=38&_ignoreionsscorebelow=20&report=0&_sigthreshold=0.001&_msresflags=1089&_msresflags2=2&percolate=0&percolate_rt=0))

**67 - 75 511.7803 1021.5461 1021.5444 2 0 K.NDVLASLYK.K**  ([Ions score 56](http://10.139.25.109/mascot/cgi/peptide_view.pl?file=../data/20120608/F008231.dat&query=1393&hit=1&index=orf158|conserved&px=1&section=5&ave_thresh=38&_ignoreionsscorebelow=20&report=0&_sigthreshold=0.001&_msresflags=1089&_msresflags2=2&percolate=0&percolate_rt=0))

**67 - 76 575.8270 1149.6395 1149.6393 0 0 K.NDVLASLYKK.S**  ([Ions score 36](http://10.139.25.109/mascot/cgi/peptide_view.pl?file=../data/20120608/F008231.dat&query=2880&hit=1&index=orf158|conserved&px=1&section=5&ave_thresh=38&_ignoreionsscorebelow=20&report=0&_sigthreshold=0.001&_msresflags=1089&_msresflags2=2&percolate=0&percolate_rt=0))

**77 - 95 1091.0031 2179.9916 2179.9797 5 0 K.SYLGEITIDDIPHAYEEES.E**  ([Ions score 32](http://10.139.25.109/mascot/cgi/peptide_view.pl?file=../data/20120608/F008231.dat&query=10562&hit=1&index=orf158|conserved&px=1&section=5&ave_thresh=38&_ignoreionsscorebelow=20&report=0&_sigthreshold=0.001&_msresflags=1089&_msresflags2=2&percolate=0&percolate_rt=0))

**77 - 101 970.7792 2909.3159 2909.2978 6 0 K.SYLGEITIDDIPHAYEEESEVKEEN.I** Deamidated (NQ)([Ions score 47](http://10.139.25.109/mascot/cgi/peptide_view.pl?file=../data/20120608/F008231.dat&query=11907&hit=1&index=orf158|conserved&px=1&section=5&ave_thresh=38&_ignoreionsscorebelow=20&report=0&_sigthreshold=0.001&_msresflags=1089&_msresflags2=2&percolate=0&percolate_rt=0))

**77 - 101 970.7817 2909.3234 2909.2978 9 0 K.SYLGEITIDDIPHAYEEESEVKEEN.I** Deamidated (NQ)([Ions score 56](http://10.139.25.109/mascot/cgi/peptide_view.pl?file=../data/20120608/F008231.dat&query=11908&hit=1&index=orf158|conserved&px=1&section=5&ave_thresh=38&_ignoreionsscorebelow=20&report=0&_sigthreshold=0.001&_msresflags=1089&_msresflags2=2&percolate=0&percolate_rt=0))

**79 - 96 1030.4696 2058.9246 2058.9269 -1 0 Y.LGEITIDDIPHAYEEESE.V**  ([Ions score 64](http://10.139.25.109/mascot/cgi/peptide_view.pl?file=../data/20120608/F008231.dat&query=9910&hit=1&index=orf158|conserved&px=1&section=5&ave_thresh=38&_ignoreionsscorebelow=20&report=0&_sigthreshold=0.001&_msresflags=1089&_msresflags2=2&percolate=0&percolate_rt=0))

**79 - 101 887.4130 2659.2172 2659.2024 6 0 Y.LGEITIDDIPHAYEEESEVKEEN.I**  Deamidated (NQ) ([Ions score 64](http://10.139.25.109/mascot/cgi/peptide_view.pl?file=../data/20120608/F008231.dat&query=11676&hit=1&index=orf158|conserved&px=1&section=5&ave_thresh=38&_ignoreionsscorebelow=20&report=0&_sigthreshold=0.001&_msresflags=1089&_msresflags2=2&percolate=0&percolate_rt=0))

**79 - 101 887.4133 2659.2180 2659.2024 6 0 Y.LGEITIDDIPHAYEEESEVKEEN.I**  Deamidated (NQ) ([Ions score 52](http://10.139.25.109/mascot/cgi/peptide_view.pl?file=../data/20120608/F008231.dat&query=11677&hit=1&index=orf158|conserved&px=1&section=5&ave_thresh=38&_ignoreionsscorebelow=20&report=0&_sigthreshold=0.001&_msresflags=1089&_msresflags2=2&percolate=0&percolate_rt=0))

**173 - 181 499.2592 996.5038 996.5029 1 0 I.LALSDWHVG.F**  ([Ions score 20](http://10.139.25.109/mascot/cgi/peptide_view.pl?file=../data/20120608/F008231.dat&query=1095&hit=1&index=orf158|conserved&px=1&section=5&ave_thresh=38&_ignoreionsscorebelow=20&report=0&_sigthreshold=0.001&_msresflags=1089&_msresflags2=2&percolate=0&percolate_rt=0))

**250 - 256 380.2158 758.4171 758.4174 0 0 Q.IAKGQQL.L**  2 Deamidated (NQ) ([Ions score 23](http://10.139.25.109/mascot/cgi/peptide_view.pl?file=../data/20120608/F008231.dat&query=141&hit=1&index=orf158|conserved&px=1&section=5&ave_thresh=38&_ignoreionsscorebelow=20&report=0&_sigthreshold=0.001&_msresflags=1089&_msresflags2=2&percolate=0&percolate_rt=0))

**303 - 315 773.8973 1545.7801 1545.7895 -6 0 N.LIMLQEKMGQLPN.V**  2 Oxidation (M) ([Ions score 20](http://10.139.25.109/mascot/cgi/peptide_view.pl?file=../data/20120608/F008231.dat&query=6646&hit=1&index=orf158|conserved&px=1&section=5&ave_thresh=38&_ignoreionsscorebelow=20&report=0&_sigthreshold=0.001&_msresflags=1089&_msresflags2=2&percolate=0&percolate_rt=0))

**399 - 412 760.3410 1518.6674 1518.6726 -3 0 R.QGNYPTTTPSQQIV.I**  Gln->pyro-Glu (N-term Q) ([Ions score 22](http://10.139.25.109/mascot/cgi/peptide_view.pl?file=../data/20120608/F008231.dat&query=6422&hit=1&index=orf158|conserved&px=1&section=5&ave_thresh=38&_ignoreionsscorebelow=20&report=0&_sigthreshold=0.001&_msresflags=1089&_msresflags2=2&percolate=0&percolate_rt=0))

9. [orf166|conserved](http://10.139.25.109/mascot/cgi/protein_view.pl?file=../data/20120608/F008231.dat&hit=orf166|conserved&db_idx=1&px=1&ave_thresh=38&_ignoreionsscorebelow=20&report=0&_sigthreshold=0.001&_msresflags=1089&_msresflags2=2&percolate=0&percolate_rt=0) hypothetical protein; putative structural protein|[vB_BceM_Bc431v3] **Mass:**14481 **Score:**222 **emPAI:**1.33

Sequence Coverage: **39%**; Matched peptides shown in **Bold Red**

**1** MEEVKMTKEM VKVEHD**LLTE RVTK**VYREKE SSFHAPHLFT VTSAEDDKTL AVIHFQEGA**L NVAGVNGVMN EDLLVMILTR LQGFQNSEFA CK**ENEMAITK

**101** **IEEALLWLR**K RTMGREKRGV LGTHTK

**Start - End Observed Mr(expt) Mr(calc) ppm Miss Sequence**

**17 - 24 480.2932 958.5717 958.5811 -10 0 D.LLTERVTK.V**  ([Ions score 22](http://10.139.25.109/mascot/cgi/peptide_view.pl?file=../data/20120608/F008231.dat&query=925&hit=1&index=orf166|conserved&px=1&section=5&ave_thresh=38&_ignoreionsscorebelow=20&report=0&_sigthreshold=0.001&_msresflags=1089&_msresflags2=2&percolate=0&percolate_rt=0))

**60 - 78 683.3541 2047.0404 2047.0217 9 0 A.LNVAGVNGVMNEDLLVMIL.T**  2 Oxidation (M) ([Ions score 26](http://10.139.25.109/mascot/cgi/peptide_view.pl?file=../data/20120608/F008231.dat&query=9863&hit=1&index=orf166|conserved&px=1&section=5&ave_thresh=38&_ignoreionsscorebelow=20&report=0&_sigthreshold=0.001&_msresflags=1089&_msresflags2=2&percolate=0&percolate_rt=0))

**77 - 89 776.9089 1551.8033 1551.8045 -1 0 M.ILTRLQGFQNSEF.A**  ([Ions score 47](http://10.139.25.109/mascot/cgi/peptide_view.pl?file=../data/20120608/F008231.dat&query=6696&hit=1&index=orf166|conserved&px=1&section=5&ave_thresh=38&_ignoreionsscorebelow=20&report=0&_sigthreshold=0.001&_msresflags=1089&_msresflags2=2&percolate=0&percolate_rt=0))

**77 - 89 776.9100 1551.8054 1551.8045 1 0 M.ILTRLQGFQNSEF.A**  ([Ions score 43](http://10.139.25.109/mascot/cgi/peptide_view.pl?file=../data/20120608/F008231.dat&query=6697&hit=1&index=orf166|conserved&px=1&section=5&ave_thresh=38&_ignoreionsscorebelow=20&report=0&_sigthreshold=0.001&_msresflags=1089&_msresflags2=2&percolate=0&percolate_rt=0))

**79 - 89 663.8251 1325.6357 1325.6364 -1 0 L.TRLQGFQNSEF.A**  ([Ions score 63](http://10.139.25.109/mascot/cgi/peptide_view.pl?file=../data/20120608/F008231.dat&query=4993&hit=1&index=orf166|conserved&px=1&section=5&ave_thresh=38&_ignoreionsscorebelow=20&report=0&_sigthreshold=0.001&_msresflags=1089&_msresflags2=2&percolate=0&percolate_rt=0))

**79 - 89 663.8266 1325.6386 1325.6364 2 0 L.TRLQGFQNSEF.A**  ([Ions score 68](http://10.139.25.109/mascot/cgi/peptide_view.pl?file=../data/20120608/F008231.dat&query=4994&hit=1&index=orf166|conserved&px=1&section=5&ave_thresh=38&_ignoreionsscorebelow=20&report=0&_sigthreshold=0.001&_msresflags=1089&_msresflags2=2&percolate=0&percolate_rt=0))

**81 - 92 714.8324 1427.6502 1427.6503 0 0 R.LQGFQNSEFACK.E**  ([Ions score 64](http://10.139.25.109/mascot/cgi/peptide_view.pl?file=../data/20120608/F008231.dat&query=5768&hit=1&index=orf166|conserved&px=1&section=5&ave_thresh=38&_ignoreionsscorebelow=20&report=0&_sigthreshold=0.001&_msresflags=1089&_msresflags2=2&percolate=0&percolate_rt=0))

**81 - 92 714.8336 1427.6526 1427.6503 2 0 R.LQGFQNSEFACK.E**  ([Ions score 61](http://10.139.25.109/mascot/cgi/peptide_view.pl?file=../data/20120608/F008231.dat&query=5769&hit=1&index=orf166|conserved&px=1&section=5&ave_thresh=38&_ignoreionsscorebelow=20&report=0&_sigthreshold=0.001&_msresflags=1089&_msresflags2=2&percolate=0&percolate_rt=0))

**101 - 109 571.8322 1141.6499 1141.6495 0 0 K.IEEALLWLR.K**  ([Ions score 66](http://10.139.25.109/mascot/cgi/peptide_view.pl?file=../data/20120608/F008231.dat&query=2781&hit=1&index=orf166|conserved&px=1&section=5&ave_thresh=38&_ignoreionsscorebelow=20&report=0&_sigthreshold=0.001&_msresflags=1089&_msresflags2=2&percolate=0&percolate_rt=0))

**101 - 109 571.8326 1141.6506 1141.6495 1 0 K.IEEALLWLR.K**  ([Ions score 67](http://10.139.25.109/mascot/cgi/peptide_view.pl?file=../data/20120608/F008231.dat&query=2782&hit=1&index=orf166|conserved&px=1&section=5&ave_thresh=38&_ignoreionsscorebelow=20&report=0&_sigthreshold=0.001&_msresflags=1089&_msresflags2=2&percolate=0&percolate_rt=0))

10. [orf218|putative](http://10.139.25.109/mascot/cgi/protein_view.pl?file=../data/20120608/F008231.dat&hit=orf218|putative&db_idx=1&px=1&ave_thresh=38&_ignoreionsscorebelow=20&report=0&_sigthreshold=0.001&_msresflags=1089&_msresflags2=2&percolate=0&percolate_rt=0) tail protein possessing endo-beta-N-acetylglucosaminidase activity; putative membrane protein|[vB_BceM_Bc431v3]   **Mass:** 136257   **Score:** 194   **emPAI:** 0.07

Sequence Coverage: **35%**; Matched peptides shown in **Bold Red**

**1** MAGNKQDYII EL**DAKIDGAV AKLNKI**RKMM DDIERIRDKG ADN**NYTASTQ DINKNMRVMK LLTQQYN**QAN EE**LKKLQSQI NKTPKGDKRN EQH**KRVREEQ

**101** KAIRREYDST LGKFREIASF QQKYSK**NFNA TVGQINLPTK** DFERTKEVIT QMVEE**SNKAK NKLDEVVMK**I REVNKLDRRS ESLSRRASAS KYMSFQQSSN

**201** FKKDRNTVDG YHQEKADNIR RMTEMSTSVT SLMKQIKKIE EKPQATKAEM DRKLEMQRTI ESMDKEFESR TELNRVLERT IANMERYNST VQGVTVKPER

**301** GTFKGMAYER APAIGLAITG AVAAALGSLY HQGAS**IDKGM RQDEI**SIGQR IGMDGSQWRE DIRNNALNSG L**KDKLGMSGQ EMIGF**QENYL SKRGFSGMQD

**401** MNDAMQNQAI FSRVSGIGTE DTKSFYNTVY GAGP**VNGKQT KEI**QNAFLGA IKRSGMEGRE KD**QLKALDGI LSGMSDGRSM TNDEIMNTMG LQSALAQTGN**

**501 RSLQGEKGGK MLQSLDQGIR NGI**DNPMVRM VFGQGTKYQG LSGRWELTKR LEKGISDIDN VRDIASFAQS QGATKEDQNM NFHAFARNSL GADITAQQAE

**601** AFMDAYRKGD LNDKNIKDIL KEDASVGNKV SKDKMEEYKK SSAATNNQ**SD ATTEKQAAGI YDMGEAVRKA NGALSGI**HPA AYAAIAALGA LTIAFAAAAT

**701** SFAISSGVRS LASSSFGGKG KRGGRGGGRG GGGGGVTPPI VGGGSGRSST GGSGSGPVAW RRNSPTPNGA NAPKQ**NFFSR MFGG**GSGASS GAVSGGTVAG

**801** ASRGGGFLKG AGKVAGKALL PIAAIMGI**NE IMSAPKEKKG ETTGSVAGGI LGGLAGGAAA GFAAGTIVPG IGNVAGA**IIG GVGGLIGGI**A GAFGGGKIG**S

**901** WFDSDAKKDK QKKEEAKAKA KAKEEKAKAE AKVTNSSSIT GFGQQGGTVA GY**TATGMAMG TTAGVMASNL DPGLTNQVVT PEAG**NGVNST NTQVDRENTN

**1001** TKQRTEVTKT DNLSYERENL NIYERAL**MKA EQLLAQARSQ NGIFGNGNGA GGAGGAGGGM GVTGGGKLQV LSAGQKWQNA SNLQQSD**LGY TEATL**TAADL**

**1101 DNWINSKAPE GSMM**RGMGAT FLKAGQEYGL DPRYLVAHAA EESAWGTSRI AKDKGNFFGI GAFDNSPYSS AYEFKDGGGT AAEKGIMGGA KWISEKYYGK

**1201** GRTTLDK**MHQ AGYATNSDWA SNIASIMGGA PKGTGQAVNA TINVNVK**G**DE SVAKKINNSS EMKKVGNNIA DMLGFYSK**EM VMT

**Start - End Observed Mr(expt) Mr(calc) ppm Miss Sequence**

**13 - 24 608.3326 1214.6506 1214.6506 0 0 L.DAKIDGAVAKLN.K**  Deamidated (NQ) ([Ions score 25](http://10.139.25.109/mascot/cgi/peptide_view.pl?file=../data/20120608/F008231.dat&query=3711&hit=1&index=orf218|putative&px=1&section=5&ave_thresh=38&_ignoreionsscorebelow=20&report=0&_sigthreshold=0.001&_msresflags=1089&_msresflags2=2&percolate=0&percolate_rt=0))

**16 - 26 571.8421 1141.6696 1141.6706 -1 0 K.IDGAVAKLNKI.R**  Deamidated (NQ) ([Ions score 20](http://10.139.25.109/mascot/cgi/peptide_view.pl?file=../data/20120608/F008231.dat&query=2784&hit=2&index=orf218|putative&px=1&section=5&ave_thresh=38&_ignoreionsscorebelow=20&report=0&_sigthreshold=0.001&_msresflags=1089&_msresflags2=2&percolate=0&percolate_rt=0))

**44 - 67 970.7817 2909.3234 2909.3422 -6 0 N.NYTASTQDINKNMRVMKLLTQQYN.Q**  2 Oxidation (M) ([Ions score 22](http://10.139.25.109/mascot/cgi/peptide_view.pl?file=../data/20120608/F008231.dat&query=11908&hit=2&index=orf218|putative&px=1&section=5&ave_thresh=38&_ignoreionsscorebelow=20&report=0&_sigthreshold=0.001&_msresflags=1089&_msresflags2=2&percolate=0&percolate_rt=0))

**52 - 61 624.3574 1246.7002 1246.6889 9 0 D.INKNMRVMKL.L**  Deamidated (NQ) ([Ions score 28](http://10.139.25.109/mascot/cgi/peptide_view.pl?file=../data/20120608/F008231.dat&query=4101&hit=1&index=orf218|putative&px=1&section=5&ave_thresh=38&_ignoreionsscorebelow=20&report=0&_sigthreshold=0.001&_msresflags=1089&_msresflags2=2&percolate=0&percolate_rt=0))

**52 - 61 624.3574 1246.7003 1246.6889 9 0 D.INKNMRVMKL.L**  Deamidated (NQ) ([Ions score 28](http://10.139.25.109/mascot/cgi/peptide_view.pl?file=../data/20120608/F008231.dat&query=4102&hit=1&index=orf218|putative&px=1&section=5&ave_thresh=38&_ignoreionsscorebelow=20&report=0&_sigthreshold=0.001&_msresflags=1089&_msresflags2=2&percolate=0&percolate_rt=0))

**73 - 81 536.8239 1071.6333 1071.6288 4 0 E.LKKLQSQIN.K**  Deamidated (NQ) ([Ions score 21](http://10.139.25.109/mascot/cgi/peptide_view.pl?file=../data/20120608/F008231.dat&query=1920&hit=1&index=orf218|putative&px=1&section=5&ave_thresh=38&_ignoreionsscorebelow=20&report=0&_sigthreshold=0.001&_msresflags=1089&_msresflags2=2&percolate=0&percolate_rt=0))

**76 - 93 1062.5437 2123.0728 2123.0607 6 0 K.LQSQINKTPKGDKRNEQH.K**  3 Deamidated (NQ) ([Ions score 37](http://10.139.25.109/mascot/cgi/peptide_view.pl?file=../data/20120608/F008231.dat&query=10291&hit=1&index=orf218|putative&px=1&section=5&ave_thresh=38&_ignoreionsscorebelow=20&report=0&_sigthreshold=0.001&_msresflags=1089&_msresflags2=2&percolate=0&percolate_rt=0))

**127 - 140 506.2733 1515.7979 1515.8045 -4 0 K.NFNATVGQINLPTK.D**  ([Ions score 21](http://10.139.25.109/mascot/cgi/peptide_view.pl?file=../data/20120608/F008231.dat&query=6397&hit=1&index=orf218|putative&px=1&section=5&ave_thresh=38&_ignoreionsscorebelow=20&report=0&_sigthreshold=0.001&_msresflags=1089&_msresflags2=2&percolate=0&percolate_rt=0))

**156 - 168 739.3747 1476.7348 1476.7493 -10 0 E.SNKAKNKLDEVVM.K**  2 Deamidated (NQ) ([Ions score 22](http://10.139.25.109/mascot/cgi/peptide_view.pl?file=../data/20120608/F008231.dat&query=6114&hit=1&index=orf218|putative&px=1&section=5&ave_thresh=38&_ignoreionsscorebelow=20&report=0&_sigthreshold=0.001&_msresflags=1089&_msresflags2=2&percolate=0&percolate_rt=0))

**161 - 169 538.2957 1074.5769 1074.5743 2 0 K.NKLDEVVMK.I**  ([Ions score 23](http://10.139.25.109/mascot/cgi/peptide_view.pl?file=../data/20120608/F008231.dat&query=1966&hit=1&index=orf218|putative&px=1&section=5&ave_thresh=38&_ignoreionsscorebelow=20&report=0&_sigthreshold=0.001&_msresflags=1089&_msresflags2=2&percolate=0&percolate_rt=0))

**336 - 345 602.8068 1203.5991 1203.5918 6 0 S.IDKGMRQDEI.S**  ([Ions score 20](http://10.139.25.109/mascot/cgi/peptide_view.pl?file=../data/20120608/F008231.dat&query=3598&hit=1&index=orf218|putative&px=1&section=5&ave_thresh=38&_ignoreionsscorebelow=20&report=0&_sigthreshold=0.001&_msresflags=1089&_msresflags2=2&percolate=0&percolate_rt=0))

**372 - 385 514.2500 1539.7282 1539.7425 -9 0 L.KDKLGMSGQEMIGF.Q**  ([Ions score 24](http://10.139.25.109/mascot/cgi/peptide_view.pl?file=../data/20120608/F008231.dat&query=6591&hit=1&index=orf218|putative&px=1&section=5&ave_thresh=38&_ignoreionsscorebelow=20&report=0&_sigthreshold=0.001&_msresflags=1089&_msresflags2=2&percolate=0&percolate_rt=0))

**435 - 443 509.7737 1017.5329 1017.5342 -1 0 P.VNGKQTKEI.Q**  2 Deamidated (NQ) ([Ions score 23](http://10.139.25.109/mascot/cgi/peptide_view.pl?file=../data/20120608/F008231.dat&query=1347&hit=1&index=orf218|putative&px=1&section=5&ave_thresh=38&_ignoreionsscorebelow=20&report=0&_sigthreshold=0.001&_msresflags=1089&_msresflags2=2&percolate=0&percolate_rt=0))

**435 - 443 509.7749 1017.5352 1017.5342 1 0 P.VNGKQTKEI.Q**  2 Deamidated (NQ) ([Ions score 23](http://10.139.25.109/mascot/cgi/peptide_view.pl?file=../data/20120608/F008231.dat&query=1348&hit=1&index=orf218|putative&px=1&section=5&ave_thresh=38&_ignoreionsscorebelow=20&report=0&_sigthreshold=0.001&_msresflags=1089&_msresflags2=2&percolate=0&percolate_rt=0))

**463 - 497 1244.8953 3731.6641 3731.6885 -7 0 D.QLKALDGILSGMSDGRSMTNDEIMNTMGLQSALAQ.T** 2Ox(M)([Ions score 22](http://10.139.25.109/mascot/cgi/peptide_view.pl?file=../data/20120608/F008231.dat&query=12241&hit=1&index=orf218|putative&px=1&section=5&ave_thresh=38&_ignoreionsscorebelow=20&report=0&_sigthreshold=0.001&_msresflags=1089&_msresflags2=2&percolate=0&percolate_rt=0))

**484 - 513 1071.5034 3211.4884 3211.5046 -5 0 D.EIMNTMGLQSALAQTGNRSLQGEKGGKMLQ.S**([Ions score 33](http://10.139.25.109/mascot/cgi/peptide_view.pl?file=../data/20120608/F008231.dat&query=12070&hit=1&index=orf218|putative&px=1&section=5&ave_thresh=38&_ignoreionsscorebelow=20&report=0&_sigthreshold=0.001&_msresflags=1089&_msresflags2=2&percolate=0&percolate_rt=0))

**486 - 495 541.2538 1080.4930 1080.4944 -1 0 I.MNTMGLQSAL.A**  Oxidation (M) ([Ions score 22](http://10.139.25.109/mascot/cgi/peptide_view.pl?file=../data/20120608/F008231.dat&query=2039&hit=1&index=orf218|putative&px=1&section=5&ave_thresh=38&_ignoreionsscorebelow=20&report=0&_sigthreshold=0.001&_msresflags=1089&_msresflags2=2&percolate=0&percolate_rt=0))

**503 - 520 659.0159 1974.0258 1974.0204 3 0 S.LQGEKGGKMLQSLDQGIR.N**  Oxidation (M) ([Ions score 21](http://10.139.25.109/mascot/cgi/peptide_view.pl?file=../data/20120608/F008231.dat&query=9442&hit=3&index=orf218|putative&px=1&section=5&ave_thresh=38&_ignoreionsscorebelow=20&report=0&_sigthreshold=0.001&_msresflags=1089&_msresflags2=2&percolate=0&percolate_rt=0))

**513 - 523 601.3123 1200.6101 1200.6098 0 0 L.QSLDQGIRNGI.D**  Deamidated (NQ) ([Ions score 22](http://10.139.25.109/mascot/cgi/peptide_view.pl?file=../data/20120608/F008231.dat&query=3561&hit=1&index=orf218|putative&px=1&section=5&ave_thresh=38&_ignoreionsscorebelow=20&report=0&_sigthreshold=0.001&_msresflags=1089&_msresflags2=2&percolate=0&percolate_rt=0))

**649 - 663 801.3544 1600.6942 1600.6926 1 0 Q.SDATTEKQAAGIYDM.G**  Deamidated (NQ) ([Ions score 23](http://10.139.25.109/mascot/cgi/peptide_view.pl?file=../data/20120608/F008231.dat&query=7093&hit=1&index=orf218|putative&px=1&section=5&ave_thresh=38&_ignoreionsscorebelow=20&report=0&_sigthreshold=0.001&_msresflags=1089&_msresflags2=2&percolate=0&percolate_rt=0))

**649 - 663 801.3563 1600.6980 1600.6926 3 0 Q.SDATTEKQAAGIYDM.G**  Deamidated (NQ) ([Ions score 21](http://10.139.25.109/mascot/cgi/peptide_view.pl?file=../data/20120608/F008231.dat&query=7094&hit=2&index=orf218|putative&px=1&section=5&ave_thresh=38&_ignoreionsscorebelow=20&report=0&_sigthreshold=0.001&_msresflags=1089&_msresflags2=2&percolate=0&percolate_rt=0))

**663 - 677 737.8855 1473.7564 1473.7609 -3 0 D.MGEAVRKANGALSGI.H**  Deamidated (NQ) ([Ions score 26](http://10.139.25.109/mascot/cgi/peptide_view.pl?file=../data/20120608/F008231.dat&query=6100&hit=1&index=orf218|putative&px=1&section=5&ave_thresh=38&_ignoreionsscorebelow=20&report=0&_sigthreshold=0.001&_msresflags=1089&_msresflags2=2&percolate=0&percolate_rt=0))

**776 - 784 539.7451 1077.4756 1077.4702 5 0 Q.NFFSRMFGG.G**  Oxidation (M) ([Ions score 23](http://10.139.25.109/mascot/cgi/peptide_view.pl?file=../data/20120608/F008231.dat&query=2006&hit=1&index=orf218|putative&px=1&section=5&ave_thresh=38&_ignoreionsscorebelow=20&report=0&_sigthreshold=0.001&_msresflags=1089&_msresflags2=2&percolate=0&percolate_rt=0))

**829 - 858 934.1600 2799.4581 2799.4436 5 0 I.NEIMSAPKEKKGETTGSVAGGILGGLAGGA.A**  ([Ions score 22](http://10.139.25.109/mascot/cgi/peptide_view.pl?file=../data/20120608/F008231.dat&query=11835&hit=1&index=orf218|putative&px=1&section=5&ave_thresh=38&_ignoreionsscorebelow=20&report=0&_sigthreshold=0.001&_msresflags=1089&_msresflags2=2&percolate=0&percolate_rt=0))

**848 - 877 610.0848 2436.3079 2436.3125 2 0 A.GGILGGLAGGAAAGFAAGTIVPGIGNVAGA.I**  ([Ions score 27](http://10.139.25.109/mascot/cgi/peptide_view.pl?file=../data/20120608/F008231.dat&query=11321&hit=3&index=orf218|putative&px=1&section=5&ave_thresh=38&_ignoreionsscorebelow=20&report=0&_sigthreshold=0.001&_msresflags=1089&_msresflags2=2&percolate=0&percolate_rt=0))

**861 - 874 636.8514 1271.6882 1271.6874 1 0 A.GFAAGTIVPGIGNV.A**  ([Ions score 20](http://10.139.25.109/mascot/cgi/peptide_view.pl?file=../data/20120608/F008231.dat&query=4378&hit=1&index=orf218|putative&px=1&section=5&ave_thresh=38&_ignoreionsscorebelow=20&report=0&_sigthreshold=0.001&_msresflags=1089&_msresflags2=2&percolate=0&percolate_rt=0))

**890 - 899 417.7231 833.4317 833.4396 -9 0 I.AGAFGGGKIG.S**  ([Ions score 23](http://10.139.25.109/mascot/cgi/peptide_view.pl?file=../data/20120608/F008231.dat&query=430&hit=1&index=orf218|putative&px=1&section=5&ave_thresh=38&_ignoreionsscorebelow=20&report=0&_sigthreshold=0.001&_msresflags=1089&_msresflags2=2&percolate=0&percolate_rt=0))

**953 - 984 1033.1420 3096.4042 3096.3938 3 0 Y.TATGMAMGTTAGVMASNLDPGLTNQVVTPEAG.N**  2 Ox(M) ([Ions score 23](http://10.139.25.109/mascot/cgi/peptide_view.pl?file=../data/20120608/F008231.dat&query=12010&hit=1&index=orf218|putative&px=1&section=5&ave_thresh=38&_ignoreionsscorebelow=20&report=0&_sigthreshold=0.001&_msresflags=1089&_msresflags2=2&percolate=0&percolate_rt=0))

**1028 - 1036 516.7739 1031.5333 1031.5321 1 0 L.MKAEQLLAQ.A**  Deamidated (NQ) ([Ions score 26](http://10.139.25.109/mascot/cgi/peptide_view.pl?file=../data/20120608/F008231.dat&query=1485&hit=2&index=orf218|putative&px=1&section=5&ave_thresh=38&_ignoreionsscorebelow=20&report=0&_sigthreshold=0.001&_msresflags=1089&_msresflags2=2&percolate=0&percolate_rt=0))

**1030 - 1038 500.2832 998.5519 998.5509 1 0 K.AEQLLAQAR.S**  ([Ions score 60](http://10.139.25.109/mascot/cgi/peptide_view.pl?file=../data/20120608/F008231.dat&query=1122&hit=1&index=orf218|putative&px=1&section=5&ave_thresh=38&_ignoreionsscorebelow=20&report=0&_sigthreshold=0.001&_msresflags=1089&_msresflags2=2&percolate=0&percolate_rt=0))

**1030 - 1038 500.2834 998.5522 998.5509 1 0 K.AEQLLAQAR.S**  ([Ions score 52](http://10.139.25.109/mascot/cgi/peptide_view.pl?file=../data/20120608/F008231.dat&query=1123&hit=1&index=orf218|putative&px=1&section=5&ave_thresh=38&_ignoreionsscorebelow=20&report=0&_sigthreshold=0.001&_msresflags=1089&_msresflags2=2&percolate=0&percolate_rt=0))

**1033 - 1064 699.3195 2793.2490 2793.2624 -5 0 Q.LLAQARSQNGIFGNGNGAGGAGGAGGGMGVTG.G**  4 Deamidated (NQ); Oxidation (M) ([Ions score 29](http://10.139.25.109/mascot/cgi/peptide_view.pl?file=../data/20120608/F008231.dat&query=11831&hit=1&index=orf218|putative&px=1&section=5&ave_thresh=38&_ignoreionsscorebelow=20&report=0&_sigthreshold=0.001&_msresflags=1089&_msresflags2=2&percolate=0&percolate_rt=0))

**1036 - 1071 1059.4972 3175.4698 3175.4477 7 0 A.QARSQNGIFGNGNGAGGAGGAGGGMGVTGGGKLQVL.S**  5 Deamidated (NQ); Gln->pyro-Glu (N-term Q); Oxidation (M) ([Ions score 26](http://10.139.25.109/mascot/cgi/peptide_view.pl?file=../data/20120608/F008231.dat&query=12052&hit=1&index=orf218|putative&px=1&section=5&ave_thresh=38&_ignoreionsscorebelow=20&report=0&_sigthreshold=0.001&_msresflags=1089&_msresflags2=2&percolate=0&percolate_rt=0))

**1038 - 1066 804.0134 2409.0183 2409.0364 -8 0 A.RSQNGIFGNGNGAGGAGGAGGGMGVTGGG.K**  2 Deamidated (NQ); Oxidation (M) ([Ions score 22](http://10.139.25.109/mascot/cgi/peptide_view.pl?file=../data/20120608/F008231.dat&query=11247&hit=1&index=orf218|putative&px=1&section=5&ave_thresh=38&_ignoreionsscorebelow=20&report=0&_sigthreshold=0.001&_msresflags=1089&_msresflags2=2&percolate=0&percolate_rt=0))

**1038 - 1066 1205.5277 2409.0408 2409.0364 2 0 A.RSQNGIFGNGNGAGGAGGAGGGMGVTGGG.K**  2 Deamidated (NQ); Oxidation (M) ([Ions score 24](http://10.139.25.109/mascot/cgi/peptide_view.pl?file=../data/20120608/F008231.dat&query=11258&hit=1&index=orf218|putative&px=1&section=5&ave_thresh=38&_ignoreionsscorebelow=20&report=0&_sigthreshold=0.001&_msresflags=1089&_msresflags2=2&percolate=0&percolate_rt=0))

**1040 - 1064 1018.4217 2034.8288 2034.8338 -2 0 S.QNGIFGNGNGAGGAGGAGGGMGVTG.G**  2 Deamidated (NQ); Gln->pyro-Glu (N-term Q); Oxidation (M) ([Ions score 24](http://10.139.25.109/mascot/cgi/peptide_view.pl?file=../data/20120608/F008231.dat&query=9781&hit=1&index=orf218|putative&px=1&section=5&ave_thresh=38&_ignoreionsscorebelow=20&report=0&_sigthreshold=0.001&_msresflags=1089&_msresflags2=2&percolate=0&percolate_rt=0))

**1040 - 1076 1075.1676 3222.4810 3222.4735 2 0 S.QNGIFGNGNGAGGAGGAGGGMGVTGGGKLQVLSAGQK.W**  6 Deamidated (NQ); Oxidation (M) ([Ions score 24](http://10.139.25.109/mascot/cgi/peptide_view.pl?file=../data/20120608/F008231.dat&query=12080&hit=1&index=orf218|putative&px=1&section=5&ave_thresh=38&_ignoreionsscorebelow=20&report=0&_sigthreshold=0.001&_msresflags=1089&_msresflags2=2&percolate=0&percolate_rt=0))

**1052 - 1070 773.8931 1545.7717 1545.7570 10 0 G.GAGGAGGGMGVTGGGKLQV.L**  Oxidation (M) ([Ions score 23](http://10.139.25.109/mascot/cgi/peptide_view.pl?file=../data/20120608/F008231.dat&query=6644&hit=1&index=orf218|putative&px=1&section=5&ave_thresh=38&_ignoreionsscorebelow=20&report=0&_sigthreshold=0.001&_msresflags=1089&_msresflags2=2&percolate=0&percolate_rt=0))

**1070 - 1087 658.9923 1973.9551 1973.9443 5 0 Q.VLSAGQKWQNASNLQQSD.L**  Deamidated (NQ) ([Ions score 20](http://10.139.25.109/mascot/cgi/peptide_view.pl?file=../data/20120608/F008231.dat&query=9440&hit=1&index=orf218|putative&px=1&section=5&ave_thresh=38&_ignoreionsscorebelow=20&report=0&_sigthreshold=0.001&_msresflags=1089&_msresflags2=2&percolate=0&percolate_rt=0))

**1096 - 1114 1025.9678 2049.9210 2049.9135 4 0 L.TAADLDNWINSKAPEGSMM.R**  ([Ions score 32](http://10.139.25.109/mascot/cgi/peptide_view.pl?file=../data/20120608/F008231.dat&query=9868&hit=1&index=orf218|putative&px=1&section=5&ave_thresh=38&_ignoreionsscorebelow=20&report=0&_sigthreshold=0.001&_msresflags=1089&_msresflags2=2&percolate=0&percolate_rt=0))

**1208 - 1232 860.3996 2578.1770 2578.1580 7 0 K.MHQAGYATNSDWASNIASIMGGAPK.G**  Deamidated(NQ) ([Ions score 73](http://10.139.25.109/mascot/cgi/peptide_view.pl?file=../data/20120608/F008231.dat&query=11557&hit=1&index=orf218|putative&px=1&section=5&ave_thresh=38&_ignoreionsscorebelow=20&report=0&_sigthreshold=0.001&_msresflags=1089&_msresflags2=2&percolate=0&percolate_rt=0))

**1208 - 1232 860.4004 2578.1795 2578.1580 8 0 K.MHQAGYATNSDWASNIASIMGGAPK.G**  Deamidated(NQ) ([Ions score 36](http://10.139.25.109/mascot/cgi/peptide_view.pl?file=../data/20120608/F008231.dat&query=11558&hit=1&index=orf218|putative&px=1&section=5&ave_thresh=38&_ignoreionsscorebelow=20&report=0&_sigthreshold=0.001&_msresflags=1089&_msresflags2=2&percolate=0&percolate_rt=0))

**1233 - 1247 744.8814 1487.7483 1487.7467 1 0 K.GTGQAVNATINVNVK.G**  3 Deamidated (NQ) ([Ions score 34](http://10.139.25.109/mascot/cgi/peptide_view.pl?file=../data/20120608/F008231.dat&query=6182&hit=5&index=orf218|putative&px=1&section=5&ave_thresh=38&_ignoreionsscorebelow=20&report=0&_sigthreshold=0.001&_msresflags=1089&_msresflags2=2&percolate=0&percolate_rt=0))

**1233 - 1247 744.8815 1487.7485 1487.7467 1 0 K.GTGQAVNATINVNVK.G**  3 Deamidated (NQ) ([Ions score 28](http://10.139.25.109/mascot/cgi/peptide_view.pl?file=../data/20120608/F008231.dat&query=6183&hit=5&index=orf218|putative&px=1&section=5&ave_thresh=38&_ignoreionsscorebelow=20&report=0&_sigthreshold=0.001&_msresflags=1089&_msresflags2=2&percolate=0&percolate_rt=0))

**1249 - 1265 636.9875 1907.9406 1907.9509 -5 0 G.DESVAKKINNSSEMKKV.G**  2 Deamidated (NQ) ([Ions score 23](http://10.139.25.109/mascot/cgi/peptide_view.pl?file=../data/20120608/F008231.dat&query=9114&hit=1&index=orf218|putative&px=1&section=5&ave_thresh=38&_ignoreionsscorebelow=20&report=0&_sigthreshold=0.001&_msresflags=1089&_msresflags2=2&percolate=0&percolate_rt=0))

**1265 - 1278 764.8740 1527.7334 1527.7392 -4 0 K.VGNNIADMLGFYSK.E**  ([Ions score 75](http://10.139.25.109/mascot/cgi/peptide_view.pl?file=../data/20120608/F008231.dat&query=6495&hit=1&index=orf218|putative&px=1&section=5&ave_thresh=38&_ignoreionsscorebelow=20&report=0&_sigthreshold=0.001&_msresflags=1089&_msresflags2=2&percolate=0&percolate_rt=0))

**1265 - 1278 764.8769 1527.7393 1527.7392 0 0 K.VGNNIADMLGFYSK.E**  ([Ions score 80](http://10.139.25.109/mascot/cgi/peptide_view.pl?file=../data/20120608/F008231.dat&query=6496&hit=1&index=orf218|putative&px=1&section=5&ave_thresh=38&_ignoreionsscorebelow=20&report=0&_sigthreshold=0.001&_msresflags=1089&_msresflags2=2&percolate=0&percolate_rt=0))

11. [orf046|hypothetical](http://10.139.25.109/mascot/cgi/protein_view.pl?file=../data/20120608/F008231.dat&hit=orf046|hypothetical&db_idx=1&px=1&ave_thresh=38&_ignoreionsscorebelow=20&report=0&_sigthreshold=0.001&_msresflags=1089&_msresflags2=2&percolate=0&percolate_rt=0) protein|[vB_BceM_Bc431v3]   **Mass:** 13598  **Score:** 155  **emPAI:** 1.43

Sequence Coverage: **39%**; Matched peptides shown in **Bold Red**

**1** **MTNTNTYNGW ANRETWLVNL HFGETLSSY**I VEQATDGAID LTEDEGTIRT EIEGNCSEYL DMILEEELNG LGSFLTDYLD LGRIEWDEIA EVIYSDDIKP

**101** M**IAELEAEEN EEEGEGEEE**

**Start - End Observed Mr(expt) Mr(calc) ppm Miss Sequence**

**1 - 13 771.8422 1541.6699 1541.6681 1 0 -.MTNTNTYNGWANR.E**  ([Ions score 93](http://10.139.25.109/mascot/cgi/peptide_view.pl?file=../data/20120608/F008231.dat&query=6608&hit=1&index=orf046|hypothetical&px=1&section=5&ave_thresh=38&_ignoreionsscorebelow=20&report=0&_sigthreshold=0.001&_msresflags=1089&_msresflags2=2&percolate=0&percolate_rt=0))

**2 - 13 706.3225 1410.6305 1410.6276 2 0 M.TNTNTYNGWANR.E**  ([Ions score 51](http://10.139.25.109/mascot/cgi/peptide_view.pl?file=../data/20120608/F008231.dat&query=5648&hit=1&index=orf046|hypothetical&px=1&section=5&ave_thresh=38&_ignoreionsscorebelow=20&report=0&_sigthreshold=0.001&_msresflags=1089&_msresflags2=2&percolate=0&percolate_rt=0))

**11 - 16 388.6879 775.3612 775.3613 0 0 W.ANRETW.L**  ([Ions score 22](http://10.139.25.109/mascot/cgi/peptide_view.pl?file=../data/20120608/F008231.dat&query=196&hit=1&index=orf046|hypothetical&px=1&section=5&ave_thresh=38&_ignoreionsscorebelow=20&report=0&_sigthreshold=0.001&_msresflags=1089&_msresflags2=2&percolate=0&percolate_rt=0))

**17 - 29 740.3782 1478.7418 1478.7405 1 0 W.LVNLHFGETLSSY.I**  ([Ions score 51](http://10.139.25.109/mascot/cgi/peptide_view.pl?file=../data/20120608/F008231.dat&query=6122&hit=1&index=orf046|hypothetical&px=1&section=5&ave_thresh=38&_ignoreionsscorebelow=20&report=0&_sigthreshold=0.001&_msresflags=1089&_msresflags2=2&percolate=0&percolate_rt=0))

**17 - 29 740.3783 1478.7420 1478.7405 1 0 W.LVNLHFGETLSSY.I**  ([Ions score 51](http://10.139.25.109/mascot/cgi/peptide_view.pl?file=../data/20120608/F008231.dat&query=6123&hit=1&index=orf046|hypothetical&px=1&section=5&ave_thresh=38&_ignoreionsscorebelow=20&report=0&_sigthreshold=0.001&_msresflags=1089&_msresflags2=2&percolate=0&percolate_rt=0))

**102 - 119 1017.9105 2033.8064 2033.8072 0 0 M.IAELEAEENEEEGEGEEE.-**  ([Ions score 54](http://10.139.25.109/mascot/cgi/peptide_view.pl?file=../data/20120608/F008231.dat&query=9756&hit=1&index=orf046|hypothetical&px=1&section=5&ave_thresh=38&_ignoreionsscorebelow=20&report=0&_sigthreshold=0.001&_msresflags=1089&_msresflags2=2&percolate=0&percolate_rt=0))

12. [orf233|hypothetical](http://10.139.25.109/mascot/cgi/protein_view.pl?file=../data/20120608/F008231.dat&hit=orf233|hypothetical&db_idx=1&px=1&ave_thresh=38&_ignoreionsscorebelow=20&report=0&_sigthreshold=0.001&_msresflags=1089&_msresflags2=2&percolate=0&percolate_rt=0) protein|[vB_BceM_Bc431v3]  **Mass:** 33012  **Score:** 153  **emPAI:** 0.61

Sequence Coverage: **29%;** Matched peptides shown in **Bold Red**

**1** MSAKQTFAK**L TEDLEQLEK**S DKEKEVTIEE PKAPIEEPEV VEPAKEEPVV EEPVKEEEPK EEPKVEDEEP AKAEEPEEVE KSKKDEDEDE DKEKEKSPKD

**101** KKDKKDKDKD KKEDKEEVK**K SEDSLDSADI LKAFEAVVK**S NGNLHEKVAG LEK**TLATILE ALSKSQEVTE EVTEEPTTEE AEVTEVEEEV AK**SEQPVEEE

**201** EELEGKAVEF VSKSNGVPEV QVTEE**AEEEV EVEPFNPQNH VDE**ITRYYAE KSSTLSPGAK DNLRSAVHRI KRGQPTDNDV KLAEQIVNFY GN

**Start - End Observed Mr(expt) Mr(calc) ppm Miss Sequence**

**10 - 19 609.3160 1216.6175 1216.6187 -1 0 K.LTEDLEQLEK.S**  ([Ions score 46](http://10.139.25.109/mascot/cgi/peptide_view.pl?file=../data/20120608/F008231.dat&query=3741&hit=1&index=orf233|hypothetical&px=1&section=5&ave_thresh=38&_ignoreionsscorebelow=20&report=0&_sigthreshold=0.001&_msresflags=1089&_msresflags2=2&percolate=0&percolate_rt=0))

**10 - 19 609.3187 1216.6229 1216.6187 3 0 K.LTEDLEQLEK.S**  ([Ions score 58](http://10.139.25.109/mascot/cgi/peptide_view.pl?file=../data/20120608/F008231.dat&query=3743&hit=1&index=orf233|hypothetical&px=1&section=5&ave_thresh=38&_ignoreionsscorebelow=20&report=0&_sigthreshold=0.001&_msresflags=1089&_msresflags2=2&percolate=0&percolate_rt=0))

**120 - 132 710.8620 1419.7094 1419.7093 0 0 K.KSEDSLDSADILK.A**  ([Ions score 65](http://10.139.25.109/mascot/cgi/peptide_view.pl?file=../data/20120608/F008231.dat&query=5709&hit=1&index=orf233|hypothetical&px=1&section=5&ave_thresh=38&_ignoreionsscorebelow=20&report=0&_sigthreshold=0.001&_msresflags=1089&_msresflags2=2&percolate=0&percolate_rt=0))

**120 - 132 474.2441 1419.7104 1419.7093 1 0 K.KSEDSLDSADILK.A**  ([Ions score 30](http://10.139.25.109/mascot/cgi/peptide_view.pl?file=../data/20120608/F008231.dat&query=5710&hit=1&index=orf233|hypothetical&px=1&section=5&ave_thresh=38&_ignoreionsscorebelow=20&report=0&_sigthreshold=0.001&_msresflags=1089&_msresflags2=2&percolate=0&percolate_rt=0))

**121 - 132 646.8139 1291.6132 1291.6143 -1 0 K.SEDSLDSADILK.A**  ([Ions score 58](http://10.139.25.109/mascot/cgi/peptide_view.pl?file=../data/20120608/F008231.dat&query=4626&hit=1&index=orf233|hypothetical&px=1&section=5&ave_thresh=38&_ignoreionsscorebelow=20&report=0&_sigthreshold=0.001&_msresflags=1089&_msresflags2=2&percolate=0&percolate_rt=0))

**121 - 134 755.8681 1509.7216 1509.7198 1 0 K.SEDSLDSADILKAF.E**  ([Ions score 35](http://10.139.25.109/mascot/cgi/peptide_view.pl?file=../data/20120608/F008231.dat&query=6342&hit=1&index=orf233|hypothetical&px=1&section=5&ave_thresh=38&_ignoreionsscorebelow=20&report=0&_sigthreshold=0.001&_msresflags=1089&_msresflags2=2&percolate=0&percolate_rt=0))

**133 - 139 382.2211 762.4276 762.4276 0 0 K.AFEAVVK.S**  ([Ions score 33](http://10.139.25.109/mascot/cgi/peptide_view.pl?file=../data/20120608/F008231.dat&query=154&hit=1&index=orf233|hypothetical&px=1&section=5&ave_thresh=38&_ignoreionsscorebelow=20&report=0&_sigthreshold=0.001&_msresflags=1089&_msresflags2=2&percolate=0&percolate_rt=0))

**154 - 164 580.3494 1158.6842 1158.6860 -2 0 K.TLATILEALSK.S**  ([Ions score 42](http://10.139.25.109/mascot/cgi/peptide_view.pl?file=../data/20120608/F008231.dat&query=3028&hit=1&index=orf233|hypothetical&px=1&section=5&ave_thresh=38&_ignoreionsscorebelow=20&report=0&_sigthreshold=0.001&_msresflags=1089&_msresflags2=2&percolate=0&percolate_rt=0))

**154 - 164 580.3513 1158.6881 1158.6860 2 0 K.TLATILEALSK.S**  ([Ions score 48](http://10.139.25.109/mascot/cgi/peptide_view.pl?file=../data/20120608/F008231.dat&query=3029&hit=1&index=orf233|hypothetical&px=1&section=5&ave_thresh=38&_ignoreionsscorebelow=20&report=0&_sigthreshold=0.001&_msresflags=1089&_msresflags2=2&percolate=0&percolate_rt=0))

**165 - 192 1050.8130 3149.4155 3149.4147 0 0 K.SQEVTEEVTEEPTTEEAEVTEVEEEVAK.S** [Ions score 55](http://10.139.25.109/mascot/cgi/peptide_view.pl?file=../data/20120608/F008231.dat&query=12041&hit=1&index=orf233|hypothetical&px=1&section=5&ave_thresh=38&_ignoreionsscorebelow=20&report=0&_sigthreshold=0.001&_msresflags=1089&_msresflags2=2&percolate=0&percolate_rt=0))

**226 - 243 1055.9664 2109.9182 2109.9127 3 0 E.AEEEVEVEPFNPQNHVDE.I**  ([Ions score 30](http://10.139.25.109/mascot/cgi/peptide_view.pl?file=../data/20120608/F008231.dat&query=10219&hit=1&index=orf233|hypothetical&px=1&section=5&ave_thresh=38&_ignoreionsscorebelow=20&report=0&_sigthreshold=0.001&_msresflags=1089&_msresflags2=2&percolate=0&percolate_rt=0))

**230 - 243 826.8791 1651.7437 1651.7478 -2 0 E.VEVEPFNPQNHVDE.I**  ([Ions score 33](http://10.139.25.109/mascot/cgi/peptide_view.pl?file=../data/20120608/F008231.dat&query=7490&hit=1&index=orf233|hypothetical&px=1&section=5&ave_thresh=38&_ignoreionsscorebelow=20&report=0&_sigthreshold=0.001&_msresflags=1089&_msresflags2=2&percolate=0&percolate_rt=0))

**230 - 243 826.8803 1651.7459 1651.7478 -1 0 E.VEVEPFNPQNHVDE.I**  ([Ions score 37](http://10.139.25.109/mascot/cgi/peptide_view.pl?file=../data/20120608/F008231.dat&query=7491&hit=1&index=orf233|hypothetical&px=1&section=5&ave_thresh=38&_ignoreionsscorebelow=20&report=0&_sigthreshold=0.001&_msresflags=1089&_msresflags2=2&percolate=0&percolate_rt=0))

**230 - 243 826.8829 1651.7512 1651.7478 2 0 E.VEVEPFNPQNHVDE.I**  ([Ions score 28](http://10.139.25.109/mascot/cgi/peptide_view.pl?file=../data/20120608/F008231.dat&query=7493&hit=1&index=orf233|hypothetical&px=1&section=5&ave_thresh=38&_ignoreionsscorebelow=20&report=0&_sigthreshold=0.001&_msresflags=1089&_msresflags2=2&percolate=0&percolate_rt=0))

13. [orf126|hypothetical](http://10.139.25.109/mascot/cgi/protein_view.pl?file=../data/20120608/F008231.dat&hit=orf126|hypothetical&db_idx=1&px=1&ave_thresh=38&_ignoreionsscorebelow=20&report=0&_sigthreshold=0.001&_msresflags=1089&_msresflags2=2&percolate=0&percolate_rt=0) protein|[vB_BceM_Bc431v3]   **Mass:** 21207  **Score:** 126  **emPAI:** 0.55

Sequence Coverage: **37%**; Matched peptides shown in **Bold Red**

**1** MTVKQLSLVK **YAGEYFCTLA DYTETRSTEG YSDSASVK**SA VRTFVVKSDA TKYISFRGEA QLKNIIQENK DNPHFHEEDF RGTRMGIIAW **DMLEPLNNRF**

**101** KENKEYKK**AF AQFMK**EANDY IEGQQSK**ESS DANETNDPTE SR**AIVIRTLR SELNRVDKEI EVRQINR**EKI LQAIN**ALESL EVE

**Start - End Observed Mr(expt) Mr(calc) ppm Miss Sequence**

**11 - 26 980.4251 1958.8357 1958.8356 0 0 K.YAGEYFCTLADYTETR.S**  ([Ions score 79](http://10.139.25.109/mascot/cgi/peptide_view.pl?file=../data/20120608/F008231.dat&query=9379&hit=1&index=orf126|hypothetical&px=1&section=5&ave_thresh=38&_ignoreionsscorebelow=20&report=0&_sigthreshold=0.001&_msresflags=1089&_msresflags2=2&percolate=0&percolate_rt=0))

**27 - 38 615.7788 1229.5430 1229.5412 2 0 R.STEGYSDSASVK.S**  ([Ions score 43](http://10.139.25.109/mascot/cgi/peptide_view.pl?file=../data/20120608/F008231.dat&query=3913&hit=1&index=orf126|hypothetical&px=1&section=5&ave_thresh=38&_ignoreionsscorebelow=20&report=0&_sigthreshold=0.001&_msresflags=1089&_msresflags2=2&percolate=0&percolate_rt=0))

**91 - 100 624.8044 1247.5942 1247.5968 -2 0 W.DMLEPLNNRF.K**  ([Ions score 34](http://10.139.25.109/mascot/cgi/peptide_view.pl?file=../data/20120608/F008231.dat&query=4108&hit=1&index=orf126|hypothetical&px=1&section=5&ave_thresh=38&_ignoreionsscorebelow=20&report=0&_sigthreshold=0.001&_msresflags=1089&_msresflags2=2&percolate=0&percolate_rt=0))

**109 - 115 421.7150 841.4154 841.4156 -0 0 K.AFAQFMK.E**  ([Ions score 27](http://10.139.25.109/mascot/cgi/peptide_view.pl?file=../data/20120608/F008231.dat&query=462&hit=1&index=orf126|hypothetical&px=1&section=5&ave_thresh=38&_ignoreionsscorebelow=20&report=0&_sigthreshold=0.001&_msresflags=1089&_msresflags2=2&percolate=0&percolate_rt=0))

**128 - 142 826.3371 1650.6596 1650.6605 -1 0 K.ESSDANETNDPTESR.A**  ([Ions score 70](http://10.139.25.109/mascot/cgi/peptide_view.pl?file=../data/20120608/F008231.dat&query=7479&hit=1&index=orf126|hypothetical&px=1&section=5&ave_thresh=38&_ignoreionsscorebelow=20&report=0&_sigthreshold=0.001&_msresflags=1089&_msresflags2=2&percolate=0&percolate_rt=0))

**168 - 175 465.2684 928.5222 928.5229 -1 0 R.EKILQAIN.A**  Deamidated (NQ) ([Ions score 27](http://10.139.25.109/mascot/cgi/peptide_view.pl?file=../data/20120608/F008231.dat&query=825&hit=1&index=orf126|hypothetical&px=1&section=5&ave_thresh=38&_ignoreionsscorebelow=20&report=0&_sigthreshold=0.001&_msresflags=1089&_msresflags2=2&percolate=0&percolate_rt=0))

14. [orf215|phage](http://10.139.25.109/mascot/cgi/protein_view.pl?file=../data/20120608/F008231.dat&hit=orf215|phage&db_idx=1&px=1&ave_thresh=38&_ignoreionsscorebelow=20&report=0&_sigthreshold=0.001&_msresflags=1089&_msresflags2=2&percolate=0&percolate_rt=0) minor structural protein, putative tail fiber|[vB_BceM_Bc431v3] **Mass:** 75797 **Score:** 124  **emPAI:** 0.09

Sequence Coverage: **14%**; Matched peptides shown in **Bold Red**

**1** MEDFDYTPLS SMRFQAQLGS EVKRMYKEGE SVIKLSLARV TKVNYKYN**TV EVMTTLHKNS TAKN**PSDNGR YSAR**LPVVFG GQTPDGK**VYG SNTIVTVGSL

**101** VLIGFLEGNK DHPIVLNIYG DADNQSMLTR TTMTGGDESD EAVQRELWQL FTLYPSMTYQ NIDGRGNKEV TFSGKSFLYI TDTDPGNEYV QDGQFDYMDL

**201** PSSRYANGEL IEPVSPQSPT VLYVHQGVYD NHRVTFFLKS DGTLRVGSRH RNGKGITYQE MKTDGSFSIV QKHDTTDPEE ISKKFSKFEI SENGDVTIQS

**301** IDHKLSITKD GVLIDGKPIG SGGGGGDLE**I IKDLQE**KIEG VTTQVTMVNG KLEFKIDKVE IEIDLDSLRQ EQQKMLDSIR EKLDGLTSAL AAMKDYTIPA

**401** FEDGTVTTDE KNKVNSLLAT VKNEKAKLDE KYSQVISDPF LPATYKD**LLG IAKGNL**DSRH QALLNTIEIV MLDGVITPDE RIAVTQAFDG YNQSIE**AMDV**

**501 AFKQAMDSIL EARI**REAQEN AMKYRDTEMR KIGSQITQLA DSITAKVSSE QLSKEIEDVR SEMATKEEQK EIKDTLEQAQ K**DIDEAVTNL P**YR**IELGSTN**

**601 GLIFKNNNID TVLYAR**VYKG KDEITSQIPK EQFIWKRVSD DAEGDTAWDT AHKNIGSSFR ASKDDVPMRA TFSCDLDI

**Start - End Observed Mr(expt) Mr(calc) ppm Miss Sequence**

**49 - 64 887.4594 1772.9043 1772.9091 -3 0 N.TVEVMTTLHKNSTAKN.P**  ([Ions score 21](http://10.139.25.109/mascot/cgi/peptide_view.pl?file=../data/20120608/F008231.dat&query=8320&hit=1&index=orf215|phage&px=1&section=5&ave_thresh=38&_ignoreionsscorebelow=20&report=0&_sigthreshold=0.001&_msresflags=1089&_msresflags2=2&percolate=0&percolate_rt=0))

**75 - 87 657.8601 1313.7055 1313.6980 6 0 R.LPVVFGGQTPDGK.V**  ([Ions score 27](http://10.139.25.109/mascot/cgi/peptide_view.pl?file=../data/20120608/F008231.dat&query=4865&hit=1&index=orf215|phage&px=1&section=5&ave_thresh=38&_ignoreionsscorebelow=20&report=0&_sigthreshold=0.001&_msresflags=1089&_msresflags2=2&percolate=0&percolate_rt=0))

**330 - 336 429.7505 857.4865 857.4858 1 0 E.IIKDLQE.K**  ([Ions score 21](http://10.139.25.109/mascot/cgi/peptide_view.pl?file=../data/20120608/F008231.dat&query=534&hit=1&index=orf215|phage&px=1&section=5&ave_thresh=38&_ignoreionsscorebelow=20&report=0&_sigthreshold=0.001&_msresflags=1089&_msresflags2=2&percolate=0&percolate_rt=0))

**448 - 456 450.2815 898.5485 898.5487 0 0 D.LLGIAKGNL.D**  Deamidated (NQ) ([Ions score 24](http://10.139.25.109/mascot/cgi/peptide_view.pl?file=../data/20120608/F008231.dat&query=694&hit=1&index=orf215|phage&px=1&section=5&ave_thresh=38&_ignoreionsscorebelow=20&report=0&_sigthreshold=0.001&_msresflags=1089&_msresflags2=2&percolate=0&percolate_rt=0))

**497 - 514 1005.5073 2009.0000 2008.9961 2 0 E.AMDVAFKQAMDSILEARI.R**  Deamidated (NQ) ([Ions score 22](http://10.139.25.109/mascot/cgi/peptide_view.pl?file=../data/20120608/F008231.dat&query=9602&hit=1&index=orf215|phage&px=1&section=5&ave_thresh=38&_ignoreionsscorebelow=20&report=0&_sigthreshold=0.001&_msresflags=1089&_msresflags2=2&percolate=0&percolate_rt=0))

**497 - 514 1013.5069 2024.9992 2024.9911 4 0 E.AMDVAFKQAMDSILEARI.R**  Oxidation (M) ([Ions score 22](http://10.139.25.109/mascot/cgi/peptide_view.pl?file=../data/20120608/F008231.dat&query=9696&hit=1&index=orf215|phage&px=1&section=5&ave_thresh=38&_ignoreionsscorebelow=20&report=0&_sigthreshold=0.001&_msresflags=1089&_msresflags2=2&percolate=0&percolate_rt=0))

**582 - 591 544.2570 1086.4994 1086.5081 -8 0 K.DIDEAVTNLP.Y**  Deamidated (NQ) ([Ions score 24](http://10.139.25.109/mascot/cgi/peptide_view.pl?file=../data/20120608/F008231.dat&query=2114&hit=1&index=orf215|phage&px=1&section=5&ave_thresh=38&_ignoreionsscorebelow=20&report=0&_sigthreshold=0.001&_msresflags=1089&_msresflags2=2&percolate=0&percolate_rt=0))

**594 - 605 646.3669 1290.7192 1290.7183 1 0 R.IELGSTNGLIFK.N**  ([Ions score 43](http://10.139.25.109/mascot/cgi/peptide_view.pl?file=../data/20120608/F008231.dat&query=4621&hit=1&index=orf215|phage&px=1&section=5&ave_thresh=38&_ignoreionsscorebelow=20&report=0&_sigthreshold=0.001&_msresflags=1089&_msresflags2=2&percolate=0&percolate_rt=0))

**594 - 605 646.3683 1290.7221 1290.7183 3 0 R.IELGSTNGLIFK.N**  ([Ions score 67](http://10.139.25.109/mascot/cgi/peptide_view.pl?file=../data/20120608/F008231.dat&query=4622&hit=1&index=orf215|phage&px=1&section=5&ave_thresh=38&_ignoreionsscorebelow=20&report=0&_sigthreshold=0.001&_msresflags=1089&_msresflags2=2&percolate=0&percolate_rt=0))

**599 - 611 732.8718 1463.7290 1463.7256 2 0 S.TNGLIFKNNNIDT.V**  Deamidated (NQ) ([Ions score 25](http://10.139.25.109/mascot/cgi/peptide_view.pl?file=../data/20120608/F008231.dat&query=6006&hit=1&index=orf215|phage&px=1&section=5&ave_thresh=38&_ignoreionsscorebelow=20&report=0&_sigthreshold=0.001&_msresflags=1089&_msresflags2=2&percolate=0&percolate_rt=0))

**606 - 616 646.8326 1291.6506 1291.6520 -1 0 K.NNNIDTVLYAR.V**  ([Ions score 62](http://10.139.25.109/mascot/cgi/peptide_view.pl?file=../data/20120608/F008231.dat&query=4636&hit=1&index=orf215|phage&px=1&section=5&ave_thresh=38&_ignoreionsscorebelow=20&report=0&_sigthreshold=0.001&_msresflags=1089&_msresflags2=2&percolate=0&percolate_rt=0))

**606 - 616 646.8328 1291.6511 1291.6520 -1 0 K.NNNIDTVLYAR.V**  ([Ions score 61](http://10.139.25.109/mascot/cgi/peptide_view.pl?file=../data/20120608/F008231.dat&query=4637&hit=1&index=orf215|phage&px=1&section=5&ave_thresh=38&_ignoreionsscorebelow=20&report=0&_sigthreshold=0.001&_msresflags=1089&_msresflags2=2&percolate=0&percolate_rt=0))

15. [orf003|conserved](http://10.139.25.109/mascot/cgi/protein_view.pl?file=../data/20120608/F008231.dat&hit=orf003|conserved&db_idx=1&px=1&ave_thresh=38&_ignoreionsscorebelow=20&report=0&_sigthreshold=0.001&_msresflags=1089&_msresflags2=2&percolate=0&percolate_rt=0) hypothetical membrane protein|[vB_BceM_Bc431v3]  **Mass:** 12260  **Score:** 106  **emPAI:** 0.64

Sequence Coverage: **46%**; Matched peptides shown in **Bold Red**

**1** **MNNQQVLLKL QE**IESTLQDQ EQSTVELKTV VDELRGIVK**D IDKNMAISEE K**QSHLFYR**IE HLEQELEELE EKGEK**GTDRQ **QKLIENAL**MV ILGGLISYIF

**101** SLASKH

**Start - End Observed Mr(expt) Mr(calc) ppm Miss Sequence**

**1 - 9 544.3002 1086.5858 1086.5855 0 0 -.MNNQQVLLK.L**  ([Ions score 47](http://10.139.25.109/mascot/cgi/peptide_view.pl?file=../data/20120608/F008231.dat&query=2117&hit=1&index=orf003|conserved&px=1&section=5&ave_thresh=38&_ignoreionsscorebelow=20&report=0&_sigthreshold=0.001&_msresflags=1089&_msresflags2=2&percolate=0&percolate_rt=0))

**1 - 9 544.3007 1086.5869 1086.5855 1 0 -.MNNQQVLLK.L**  ([Ions score 51](http://10.139.25.109/mascot/cgi/peptide_view.pl?file=../data/20120608/F008231.dat&query=2118&hit=1&index=orf003|conserved&px=1&section=5&ave_thresh=38&_ignoreionsscorebelow=20&report=0&_sigthreshold=0.001&_msresflags=1089&_msresflags2=2&percolate=0&percolate_rt=0))

**4 - 12 550.3269 1098.6392 1098.6284 10 0 N.QQVLLKLQE.I**  Deamidated (NQ) ([Ions score 28](http://10.139.25.109/mascot/cgi/peptide_view.pl?file=../data/20120608/F008231.dat&query=2278&hit=4&index=orf003|conserved&px=1&section=5&ave_thresh=38&_ignoreionsscorebelow=20&report=0&_sigthreshold=0.001&_msresflags=1089&_msresflags2=2&percolate=0&percolate_rt=0))

**40 - 51 696.8381 1391.6617 1391.6602 1 0 K.DIDKNMAISEEK.Q**  ([Ions score 54](http://10.139.25.109/mascot/cgi/peptide_view.pl?file=../data/20120608/F008231.dat&query=5522&hit=1&index=orf003|conserved&px=1&section=5&ave_thresh=38&_ignoreionsscorebelow=20&report=0&_sigthreshold=0.001&_msresflags=1089&_msresflags2=2&percolate=0&percolate_rt=0))

**40 - 51 696.8387 1391.6628 1391.6602 2 0 K.DIDKNMAISEEK.Q**  ([Ions score 69](http://10.139.25.109/mascot/cgi/peptide_view.pl?file=../data/20120608/F008231.dat&query=5523&hit=1&index=orf003|conserved&px=1&section=5&ave_thresh=38&_ignoreionsscorebelow=20&report=0&_sigthreshold=0.001&_msresflags=1089&_msresflags2=2&percolate=0&percolate_rt=0))

**40 - 51 464.8950 1391.6631 1391.6602 2 0 K.DIDKNMAISEEK.Q**  ([Ions score 28](http://10.139.25.109/mascot/cgi/peptide_view.pl?file=../data/20120608/F008231.dat&query=5524&hit=1&index=orf003|conserved&px=1&section=5&ave_thresh=38&_ignoreionsscorebelow=20&report=0&_sigthreshold=0.001&_msresflags=1089&_msresflags2=2&percolate=0&percolate_rt=0))

**59 - 75 521.5119 2082.0185 2082.0004 9 0 R.IEHLEQELEELEEKGEK.G**  Deamidated (NQ) ([Ions score 27](http://10.139.25.109/mascot/cgi/peptide_view.pl?file=../data/20120608/F008231.dat&query=10058&hit=1&index=orf003|conserved&px=1&section=5&ave_thresh=38&_ignoreionsscorebelow=20&report=0&_sigthreshold=0.001&_msresflags=1089&_msresflags2=2&percolate=0&percolate_rt=0))

**81 - 88 465.2684 928.5222 928.5229 -1 0 Q.QKLIENAL.M**  Deamidated (NQ) ([Ions score 27](http://10.139.25.109/mascot/cgi/peptide_view.pl?file=../data/20120608/F008231.dat&query=825&hit=3&index=orf003|conserved&px=1&section=5&ave_thresh=38&_ignoreionsscorebelow=20&report=0&_sigthreshold=0.001&_msresflags=1089&_msresflags2=2&percolate=0&percolate_rt=0))

16. [orf205|conserved](http://10.139.25.109/mascot/cgi/protein_view.pl?file=../data/20120608/F008231.dat&hit=orf205|conserved&db_idx=1&px=1&ave_thresh=38&_ignoreionsscorebelow=20&report=0&_sigthreshold=0.001&_msresflags=1089&_msresflags2=2&percolate=0&percolate_rt=0) hypothetical protein, putative baseplate protein|[vB_BceM_Bc431v3] **Mass:** 38896 **Score:**102 **emPAI:**0.28

Sequence Coverage: **23%;** Matched peptides shown in **Bold Red**

**1** MRYKQMTEIY GRLVDHTITN TNKINDF**SIG SAVRAIY**EAT **AREIEQLYIL** TEENIREAIA TGVYSSFGFQ RKPAQRAYGK VQLVFHNAVQ QTLPLPRGTR

**101** FTSSLSDYTM TYETVEDYYI PQGTVTAEVQ IYCTITGEIG NVPN**NVINIM MTPLANIKTV TNAQAFQTGQ DEE**PLEELKS RFRSYIESLS KGTIPALEYG

**201** TRSVIEISGV WIDEQTGIVY VYAHDRNGDL PDVVRDKVIA T**LQNYRAAGI PV**VVRPVTRK AVNIDVTIVL TDKTAITKAL QDKIVAEISR YLNNMQTSQS

**301** VILSDLSSVI KGIDRR**LIYD ITFNDPKANV IVAGNEVVR**A GTVKVTLT

**Start - End Observed Mr(expt) Mr(calc) ppm Miss Sequence**

**28 - 37 518.7915 1035.5684 1035.5713 -3 0 F.SIGSAVRAIY.E**  ([Ions score 28](http://10.139.25.109/mascot/cgi/peptide_view.pl?file=../data/20120608/F008231.dat&query=1532&hit=1&index=orf205|conserved&px=1&section=5&ave_thresh=38&_ignoreionsscorebelow=20&report=0&_sigthreshold=0.001&_msresflags=1089&_msresflags2=2&percolate=0&percolate_rt=0))

**41 - 50 624.3574 1246.7002 1246.6921 6 0 T.AREIEQLYIL.T**  ([Ions score 20](http://10.139.25.109/mascot/cgi/peptide_view.pl?file=../data/20120608/F008231.dat&query=4101&hit=4&index=orf205|conserved&px=1&section=5&ave_thresh=38&_ignoreionsscorebelow=20&report=0&_sigthreshold=0.001&_msresflags=1089&_msresflags2=2&percolate=0&percolate_rt=0))

**42 - 47 394.7107 787.4068 787.4075 -1 0 A.REIEQL.Y**  Deamidated (NQ) ([Ions score 20](http://10.139.25.109/mascot/cgi/peptide_view.pl?file=../data/20120608/F008231.dat&query=231&hit=4&index=orf205|conserved&px=1&section=5&ave_thresh=38&_ignoreionsscorebelow=20&report=0&_sigthreshold=0.001&_msresflags=1089&_msresflags2=2&percolate=0&percolate_rt=0))

**42 - 47 394.7111 787.4077 787.4075 0 0 A.REIEQL.Y**  Deamidated (NQ) ([Ions score 24](http://10.139.25.109/mascot/cgi/peptide_view.pl?file=../data/20120608/F008231.dat&query=232&hit=4&index=orf205|conserved&px=1&section=5&ave_thresh=38&_ignoreionsscorebelow=20&report=0&_sigthreshold=0.001&_msresflags=1089&_msresflags2=2&percolate=0&percolate_rt=0))

**145 - 153 525.2590 1048.5035 1048.4933 10 0 N.NVINIMMTP.L** Deamidated (NQ); Oxidation (M) ([Ions score 23](http://10.139.25.109/mascot/cgi/peptide_view.pl?file=../data/20120608/F008231.dat&query=1674&hit=1&index=orf205|conserved&px=1&section=5&ave_thresh=38&_ignoreionsscorebelow=20&report=0&_sigthreshold=0.001&_msresflags=1089&_msresflags2=2&percolate=0&percolate_rt=0))

**148 - 162 838.9311 1675.8476 1675.8637 -10 0 I.NIMMTPLANIKTVTN.A**  Oxidation (M) ([Ions score 24](http://10.139.25.109/mascot/cgi/peptide_view.pl?file=../data/20120608/F008231.dat&query=7688&hit=1&index=orf205|conserved&px=1&section=5&ave_thresh=38&_ignoreionsscorebelow=20&report=0&_sigthreshold=0.001&_msresflags=1089&_msresflags2=2&percolate=0&percolate_rt=0))

**151 - 157 380.2036 758.3927 758.3996 -9 0 M.MTPLANI.K**  ([Ions score 24](http://10.139.25.109/mascot/cgi/peptide_view.pl?file=../data/20120608/F008231.dat&query=140&hit=2&index=orf205|conserved&px=1&section=5&ave_thresh=38&_ignoreionsscorebelow=20&report=0&_sigthreshold=0.001&_msresflags=1089&_msresflags2=2&percolate=0&percolate_rt=0))

**158 - 173 883.9095 1765.8044 1765.8119 -4 0 I.KTVTNAQAFQTGQDEE.P**  ([Ions score 43](http://10.139.25.109/mascot/cgi/peptide_view.pl?file=../data/20120608/F008231.dat&query=8268&hit=1&index=orf205|conserved&px=1&section=5&ave_thresh=38&_ignoreionsscorebelow=20&report=0&_sigthreshold=0.001&_msresflags=1089&_msresflags2=2&percolate=0&percolate_rt=0))

**158 - 173 589.6105 1765.8098 1765.8119 -1 0 I.KTVTNAQAFQTGQDEE.P**  ([Ions score 30](http://10.139.25.109/mascot/cgi/peptide_view.pl?file=../data/20120608/F008231.dat&query=8269&hit=1&index=orf205|conserved&px=1&section=5&ave_thresh=38&_ignoreionsscorebelow=20&report=0&_sigthreshold=0.001&_msresflags=1089&_msresflags2=2&percolate=0&percolate_rt=0))

**242 - 252 601.3385 1200.6624 1200.6615 1 0 T.LQNYRAAGIPV.V**  ([Ions score 22](http://10.139.25.109/mascot/cgi/peptide_view.pl?file=../data/20120608/F008231.dat&query=3570&hit=3&index=orf205|conserved&px=1&section=5&ave_thresh=38&_ignoreionsscorebelow=20&report=0&_sigthreshold=0.001&_msresflags=1089&_msresflags2=2&percolate=0&percolate_rt=0))

**317 - 327 669.8490 1337.6834 1337.6867 -2 0 R.LIYDITFNDPK.A**  ([Ions score 52](http://10.139.25.109/mascot/cgi/peptide_view.pl?file=../data/20120608/F008231.dat&query=5091&hit=1&index=orf205|conserved&px=1&section=5&ave_thresh=38&_ignoreionsscorebelow=20&report=0&_sigthreshold=0.001&_msresflags=1089&_msresflags2=2&percolate=0&percolate_rt=0))

**317 - 327 669.8525 1337.6904 1337.6867 3 0 R.LIYDITFNDPK.A**  ([Ions score 42](http://10.139.25.109/mascot/cgi/peptide_view.pl?file=../data/20120608/F008231.dat&query=5092&hit=1&index=orf205|conserved&px=1&section=5&ave_thresh=38&_ignoreionsscorebelow=20&report=0&_sigthreshold=0.001&_msresflags=1089&_msresflags2=2&percolate=0&percolate_rt=0))

**328 - 339 620.8534 1239.6922 1239.6935 -1 0 K.ANVIVAGNEVVR.A**  ([Ions score 63](http://10.139.25.109/mascot/cgi/peptide_view.pl?file=../data/20120608/F008231.dat&query=4024&hit=1&index=orf205|conserved&px=1&section=5&ave_thresh=38&_ignoreionsscorebelow=20&report=0&_sigthreshold=0.001&_msresflags=1089&_msresflags2=2&percolate=0&percolate_rt=0))

**328 - 339 620.8535 1239.6925 1239.6935 -1 0 K.ANVIVAGNEVVR.A**  ([Ions score 49](http://10.139.25.109/mascot/cgi/peptide_view.pl?file=../data/20120608/F008231.dat&query=4025&hit=1&index=orf205|conserved&px=1&section=5&ave_thresh=38&_ignoreionsscorebelow=20&report=0&_sigthreshold=0.001&_msresflags=1089&_msresflags2=2&percolate=0&percolate_rt=0))

17. [orf197|DNA](http://10.139.25.109/mascot/cgi/protein_view.pl?file=../data/20120608/F008231.dat&hit=orf197|DNA&db_idx=1&px=1&ave_thresh=38&_ignoreionsscorebelow=20&report=0&_sigthreshold=0.001&_msresflags=1089&_msresflags2=2&percolate=0&percolate_rt=0) helicase I|[vB_BceM_Bc431v3]    **Mass:** 55468    **Score:** 101  **emPAI:** 0.26

Sequence Coverage: **21%**; Matched peptides shown in **Bold Red**

**1** **MESPIMTQIL R**K**AIENPIFA K**EVLAVAPLT VFEGSPAYTE LASIVKRY**YQ TNNKPLTEDA LL**TLTEEKLD RMKK**DALTQQ DYFGK**VHYLY **EVRNSGDNEV**

**101 IDEKIEEY**IR QKMSIDLLTK AATNLKNKEF LEKLPDEFKK ILMLNISGKR NEIINVLDDA EYKRTSLSTL FQNMIPTGFK DIDHLNGGGL AKGELGLIVA

**201** ASGTGKTLIL TNLATNYTKN GYNVLFIALE ELENRMILKF E**QSLLRQNKS TILTGS**VLNN EQFDKRQAFI QQHRKHFGNL YFARYSPQAV TPAKIEQLIS

**301** D**LMIREGVQV** DAVVVDYPEL LRNPRSSGNE ADDGGKLFEE MRRIAQDYNV VMWTAAQMNR TAYSALVRTA EHMEGSHRKK NAAELVLTVN QTPEEYQAGF

**401** IRLYADKVRN PPEGQYNRML GFKVVGSAQT VRDFESEQER KEHQYVLEAA DEAREAMFKS **KRKDGKDNTP KIDYAGEI**NQ ALHSMRG

**Start - End Observed Mr(expt) Mr(calc) ppm Miss Sequence**

**1 - 10 581.7955 1161.5765 1161.5773 -1 0 -.MESPIMTQIL.R**  ([Ions score 34](http://10.139.25.109/mascot/cgi/peptide_view.pl?file=../data/20120608/F008231.dat&query=3059&hit=1&index=orf197|DNA&px=1&section=5&ave_thresh=38&_ignoreionsscorebelow=20&report=0&_sigthreshold=0.001&_msresflags=1089&_msresflags2=2&percolate=0&percolate_rt=0))

**1 - 11 659.8490 1317.6834 1317.6785 4 0 -.MESPIMTQILR.K**  ([Ions score 76](http://10.139.25.109/mascot/cgi/peptide_view.pl?file=../data/20120608/F008231.dat&query=4913&hit=1&index=orf197|DNA&px=1&section=5&ave_thresh=38&_ignoreionsscorebelow=20&report=0&_sigthreshold=0.001&_msresflags=1089&_msresflags2=2&percolate=0&percolate_rt=0))

**13 - 21 501.7832 1001.5518 1001.5545 -3 0 K.AIENPIFAK.E**  ([Ions score 32](http://10.139.25.109/mascot/cgi/peptide_view.pl?file=../data/20120608/F008231.dat&query=1176&hit=1&index=orf197|DNA&px=1&section=5&ave_thresh=38&_ignoreionsscorebelow=20&report=0&_sigthreshold=0.001&_msresflags=1089&_msresflags2=2&percolate=0&percolate_rt=0))

**13 - 21 501.7842 1001.5537 1001.5545 -1 0 K.AIENPIFAK.E**  ([Ions score 25](http://10.139.25.109/mascot/cgi/peptide_view.pl?file=../data/20120608/F008231.dat&query=1177&hit=1&index=orf197|DNA&px=1&section=5&ave_thresh=38&_ignoreionsscorebelow=20&report=0&_sigthreshold=0.001&_msresflags=1089&_msresflags2=2&percolate=0&percolate_rt=0))

**49 - 62 810.4188 1618.8231 1618.8202 2 0 Y.YQTNNKPLTEDALL.T**  ([Ions score 42](http://10.139.25.109/mascot/cgi/peptide_view.pl?file=../data/20120608/F008231.dat&query=7219&hit=1&index=orf197|DNA&px=1&section=5&ave_thresh=38&_ignoreionsscorebelow=20&report=0&_sigthreshold=0.001&_msresflags=1089&_msresflags2=2&percolate=0&percolate_rt=0))

**75 - 85 643.3074 1284.6003 1284.5986 1 0 K.DALTQQDYFGK.V**  ([Ions score 51](http://10.139.25.109/mascot/cgi/peptide_view.pl?file=../data/20120608/F008231.dat&query=4535&hit=1&index=orf197|DNA&px=1&section=5&ave_thresh=38&_ignoreionsscorebelow=20&report=0&_sigthreshold=0.001&_msresflags=1089&_msresflags2=2&percolate=0&percolate_rt=0))

**91 - 108 713.3353 2136.9840 2136.9811 1 0 Y.EVRNSGDNEVIDEKIEEY.I**  ([Ions score 46](http://10.139.25.109/mascot/cgi/peptide_view.pl?file=../data/20120608/F008231.dat&query=10369&hit=1&index=orf197|DNA&px=1&section=5&ave_thresh=38&_ignoreionsscorebelow=20&report=0&_sigthreshold=0.001&_msresflags=1089&_msresflags2=2&percolate=0&percolate_rt=0))

**242 - 253 701.8936 1401.7725 1401.7827 -7 0 E.QSLLRQNKSTIL.T**  2 Deamidated (NQ) ([Ions score 21](http://10.139.25.109/mascot/cgi/peptide_view.pl?file=../data/20120608/F008231.dat&query=5597&hit=2&index=orf197|DNA&px=1&section=5&ave_thresh=38&_ignoreionsscorebelow=20&report=0&_sigthreshold=0.001&_msresflags=1089&_msresflags2=2&percolate=0&percolate_rt=0))

**242 - 256 549.6376 1645.8911 1645.8999 -5 0 E.QSLLRQNKSTILTGS.V**  Deamidated (NQ) ([Ions score 26](http://10.139.25.109/mascot/cgi/peptide_view.pl?file=../data/20120608/F008231.dat&query=7430&hit=1&index=orf197|DNA&px=1&section=5&ave_thresh=38&_ignoreionsscorebelow=20&report=0&_sigthreshold=0.001&_msresflags=1089&_msresflags2=2&percolate=0&percolate_rt=0))

**242 - 256 824.4517 1646.8888 1646.8839 3 0 E.QSLLRQNKSTILTGS.V**  2 Deamidated (NQ) ([Ions score 24](http://10.139.25.109/mascot/cgi/peptide_view.pl?file=../data/20120608/F008231.dat&query=7439&hit=1&index=orf197|DNA&px=1&section=5&ave_thresh=38&_ignoreionsscorebelow=20&report=0&_sigthreshold=0.001&_msresflags=1089&_msresflags2=2&percolate=0&percolate_rt=0))

**302 - 310 522.8008 1043.5870 1043.5797 7 0 D.LMIREGVQV.D**  ([Ions score 30](http://10.139.25.109/mascot/cgi/peptide_view.pl?file=../data/20120608/F008231.dat&query=1617&hit=1&index=orf197|DNA&px=1&section=5&ave_thresh=38&_ignoreionsscorebelow=20&report=0&_sigthreshold=0.001&_msresflags=1089&_msresflags2=2&percolate=0&percolate_rt=0))

**461 - 478 1025.0249 2048.0352 2048.0538 -9 0 S.KRKDGKDNTPKIDYAGEI.N**  Deamidated (NQ) ([Ions score 21](http://10.139.25.109/mascot/cgi/peptide_view.pl?file=../data/20120608/F008231.dat&query=9864&hit=1&index=orf197|DNA&px=1&section=5&ave_thresh=38&_ignoreionsscorebelow=20&report=0&_sigthreshold=0.001&_msresflags=1089&_msresflags2=2&percolate=0&percolate_rt=0))

18. [orf203|putative](http://10.139.25.109/mascot/cgi/protein_view.pl?file=../data/20120608/F008231.dat&hit=orf203|putative&db_idx=1&px=1&ave_thresh=38&_ignoreionsscorebelow=20&report=0&_sigthreshold=0.001&_msresflags=1089&_msresflags2=2&percolate=0&percolate_rt=0) tail protein|[vB_BceM_Bc431v3]   **Mass:** 129489   **Score:** 96  **emPAI:** 0.10

Sequence Coverage: **13%**; Matched peptides shown in **Bold Red**

**1** MADIIDLSGM PYNDRFDSKK GRSKALFRSD RPLQQAELNE IQSIAEDNLK RLGDRVFSDG NIQTGMAFTF DNAETKTKIT VEDGLLYLAG KIRPFHKQTI

**101** PFTGVGRENI GVKVVQKIVT YNDDPTLLDQ TQNAPSYLSP GADRLEEQVV LTYNDDSTTM IYRFDDGKLF IEPNRPEFSG IIEMIAQRDK ETLGSYQAEG

**201** FNMWTEKGRT PETIDVVIDA GIAYVNGYRI HKPTATRVAV KKSKDFRTII QEGSTYKASK GKVTVGSIFV KQVNNV**VGRT DSPAGGVQL**S KGVLDGRDPL

**301** PAQYTNIDAS KLTVYVGAKV YVQG**KDYALV QDSGIQ**YIDW KGTLNGEEPT PGTTYFLTFE YDRVMKAGTD YKVVSTPLGD TIPGSTTDVD FNVAGGVKPK

**401** DNGTIRVDYD YYLSREDI**VT LDVTGNFT**VV EGQPDREGLT KQPENRDPLT LKIGNVHVFP FSDFAEAKNT AVMRLRMEDL QRMKTRLENV EYNQAMILLE

**501** KQATKTQDPL TLRGVFADPF TDFSKMDSAI STVAFSFDDA TITIPTKTPD DQKVKPKFME NESVANSWGR LVTAPFKEIK EISQPLATEA WNVNPYMVYN

**601** KQGVLK**LTPE TDNWIDEQR**V TLYEEDHVTT NLNRWWMHQG EGDPGG**IVSD WNKELIDKTQ L**EGGIQWNES SIGWREKQEG SFWSSAQTTR **NEVIEYMRQI**

**701 EVGFSATNLK** SNENNLFLTF DGNRVAVKPT GATAPGSDAG TVR**SNAQGEA SGTFMIPTGV RTGTREATLQ NANNQATATF T**AQGSAKITT DTITRTRVTF

**801** NLYDPLAQSF AFPQARVITS VGVYFGSKST **KDNIIMQVRG LSEGGLPNRT** IYAERVLTPD KVIVSADASK ETKIALDDPL MVQPGESYCI VFITDSADYT

**901** MWCAKMGQKT LGDNPQTVIS NPYVNGVLFS SSNAVSWTVH QETDMKFNIY TAEFEEEGII EFDTMKNIDS NGILLMASYL TPANTGCIWE VKVVNASDVG

**1001** TVSIDSVPWM PLVNYAGIET PFVVGLAKLR ARFKSNRYIS PMLVLDDLLF VNFVSATKGE YVSKTVD**QSV APFNQITLS**Y DSAAPAGTRV KPYYSLDQGA

**1101** TWKEFTKAPT TTKRSAEFTR YTYVERQAGS AVEISIKYKL VLEGDNRFVR PRVRQLTGMT TDAI

**Start - End Observed Mr(expt) Mr(calc) ppm Miss Sequence**

**277 - 289 629.3264 1256.6383 1256.6361 2 0 V.VGRTDSPAGGVQL.S**  Deamidated (NQ) ([Ions score 31](http://10.139.25.109/mascot/cgi/peptide_view.pl?file=../data/20120608/F008231.dat&query=4218&hit=1&index=orf203|putative&px=1&section=5&ave_thresh=38&_ignoreionsscorebelow=20&report=0&_sigthreshold=0.001&_msresflags=1089&_msresflags2=2&percolate=0&percolate_rt=0))

**325 - 336 669.8228 1337.6311 1337.6351 -3 0 G.KDYALVQDSGIQ.Y**  2 Deamidated (NQ) ([Ions score 30](http://10.139.25.109/mascot/cgi/peptide_view.pl?file=../data/20120608/F008231.dat&query=5089&hit=1&index=orf203|putative&px=1&section=5&ave_thresh=38&_ignoreionsscorebelow=20&report=0&_sigthreshold=0.001&_msresflags=1089&_msresflags2=2&percolate=0&percolate_rt=0))

**329 - 335 366.2004 730.3861 730.3861 0 0 A.LVQDSGI.Q**  ([Ions score 22](http://10.139.25.109/mascot/cgi/peptide_view.pl?file=../data/20120608/F008231.dat&query=70&hit=2&index=orf203|putative&px=1&section=5&ave_thresh=38&_ignoreionsscorebelow=20&report=0&_sigthreshold=0.001&_msresflags=1089&_msresflags2=2&percolate=0&percolate_rt=0))

**419 - 428 533.7743 1065.5340 1065.5343 0 0 I.VTLDVTGNFT.V**  ([Ions score 23](http://10.139.25.109/mascot/cgi/peptide_view.pl?file=../data/20120608/F008231.dat&query=1856&hit=1&index=orf203|putative&px=1&section=5&ave_thresh=38&_ignoreionsscorebelow=20&report=0&_sigthreshold=0.001&_msresflags=1089&_msresflags2=2&percolate=0&percolate_rt=0))

**607 - 619 808.8798 1615.7451 1615.7478 -2 0 K.LTPETDNWIDEQR.V**  ([Ions score 36](http://10.139.25.109/mascot/cgi/peptide_view.pl?file=../data/20120608/F008231.dat&query=7200&hit=1&index=orf203|putative&px=1&section=5&ave_thresh=38&_ignoreionsscorebelow=20&report=0&_sigthreshold=0.001&_msresflags=1089&_msresflags2=2&percolate=0&percolate_rt=0))

**607 - 619 808.8845 1615.7544 1615.7478 4 0 K.LTPETDNWIDEQR.V**  ([Ions score 53](http://10.139.25.109/mascot/cgi/peptide_view.pl?file=../data/20120608/F008231.dat&query=7201&hit=1&index=orf203|putative&px=1&section=5&ave_thresh=38&_ignoreionsscorebelow=20&report=0&_sigthreshold=0.001&_msresflags=1089&_msresflags2=2&percolate=0&percolate_rt=0))

**647 - 661 601.6612 1801.9617 1801.9462 9 0 G.IVSDWNKELIDKTQL.E**  Deamidated (NQ) ([Ions score 20](http://10.139.25.109/mascot/cgi/peptide_view.pl?file=../data/20120608/F008231.dat&query=8536&hit=1&index=orf203|putative&px=1&section=5&ave_thresh=38&_ignoreionsscorebelow=20&report=0&_sigthreshold=0.001&_msresflags=1089&_msresflags2=2&percolate=0&percolate_rt=0))

**691 - 698 527.2555 1052.4965 1052.4960 0 0 R.NEVIEYMR.Q**  ([Ions score 41](http://10.139.25.109/mascot/cgi/peptide_view.pl?file=../data/20120608/F008231.dat&query=1713&hit=1&index=orf203|putative&px=1&section=5&ave_thresh=38&_ignoreionsscorebelow=20&report=0&_sigthreshold=0.001&_msresflags=1089&_msresflags2=2&percolate=0&percolate_rt=0))

**691 - 698 527.2559 1052.4972 1052.4960 1 0 R.NEVIEYMR.Q**  ([Ions score 36](http://10.139.25.109/mascot/cgi/peptide_view.pl?file=../data/20120608/F008231.dat&query=1714&hit=1&index=orf203|putative&px=1&section=5&ave_thresh=38&_ignoreionsscorebelow=20&report=0&_sigthreshold=0.001&_msresflags=1089&_msresflags2=2&percolate=0&percolate_rt=0))

**699 - 710 653.8541 1305.6937 1305.6929 1 0 R.QIEVGFSATNLK.S**  ([Ions score 54](http://10.139.25.109/mascot/cgi/peptide_view.pl?file=../data/20120608/F008231.dat&query=4792&hit=1&index=orf203|putative&px=1&section=5&ave_thresh=38&_ignoreionsscorebelow=20&report=0&_sigthreshold=0.001&_msresflags=1089&_msresflags2=2&percolate=0&percolate_rt=0))

**744 - 761 911.9348 1821.8551 1821.8680 -7 0 R.SNAQGEASGTFMIPTGVR.T**  ([Ions score 59](http://10.139.25.109/mascot/cgi/peptide_view.pl?file=../data/20120608/F008231.dat&query=8644&hit=1&index=orf203|putative&px=1&section=5&ave_thresh=38&_ignoreionsscorebelow=20&report=0&_sigthreshold=0.001&_msresflags=1089&_msresflags2=2&percolate=0&percolate_rt=0))

**744 - 761 911.9440 1821.8735 1821.8680 3 0 R.SNAQGEASGTFMIPTGVR.T**  ([Ions score 41](http://10.139.25.109/mascot/cgi/peptide_view.pl?file=../data/20120608/F008231.dat&query=8646&hit=1&index=orf203|putative&px=1&section=5&ave_thresh=38&_ignoreionsscorebelow=20&report=0&_sigthreshold=0.001&_msresflags=1089&_msresflags2=2&percolate=0&percolate_rt=0))

**750 - 781 1115.8734 3344.5984 3344.5943 1 0 E.ASGTFMIPTGVRTGTREATLQNANNQATATFT.A**  Ox(M) ([Ions score 26](http://10.139.25.109/mascot/cgi/peptide_view.pl?file=../data/20120608/F008231.dat&query=12128&hit=1&index=orf203|putative&px=1&section=5&ave_thresh=38&_ignoreionsscorebelow=20&report=0&_sigthreshold=0.001&_msresflags=1089&_msresflags2=2&percolate=0&percolate_rt=0))

**831 - 848 648.0017 1940.9831 1940.9989 -8 0 T.KDNIIMQVRGLSEGGLPN.R**  Deamidated (NQ) ([Ions score 22](http://10.139.25.109/mascot/cgi/peptide_view.pl?file=../data/20120608/F008231.dat&query=9290&hit=1&index=orf203|putative&px=1&section=5&ave_thresh=38&_ignoreionsscorebelow=20&report=0&_sigthreshold=0.001&_msresflags=1089&_msresflags2=2&percolate=0&percolate_rt=0))

**831 - 848 648.3352 1941.9838 1941.9829 0 0 T.KDNIIMQVRGLSEGGLPN.R**  2 Deamidated (NQ) ([Ions score 36](http://10.139.25.109/mascot/cgi/peptide_view.pl?file=../data/20120608/F008231.dat&query=9296&hit=1&index=orf203|putative&px=1&section=5&ave_thresh=38&_ignoreionsscorebelow=20&report=0&_sigthreshold=0.001&_msresflags=1089&_msresflags2=2&percolate=0&percolate_rt=0))

**833 - 850 653.0071 1955.9994 1956.0098 -5 0 D.NIIMQVRGLSEGGLPNRT.I**  2 Deamidated (NQ) ([Ions score 29](http://10.139.25.109/mascot/cgi/peptide_view.pl?file=../data/20120608/F008231.dat&query=9363&hit=1&index=orf203|putative&px=1&section=5&ave_thresh=38&_ignoreionsscorebelow=20&report=0&_sigthreshold=0.001&_msresflags=1089&_msresflags2=2&percolate=0&percolate_rt=0))

**1068 - 1079 653.3399 1304.6652 1304.6612 3 0 D.QSVAPFNQITLS.Y**  Deamidated (NQ) ([Ions score 22](http://10.139.25.109/mascot/cgi/peptide_view.pl?file=../data/20120608/F008231.dat&query=4785&hit=1&index=orf203|putative&px=1&section=5&ave_thresh=38&_ignoreionsscorebelow=20&report=0&_sigthreshold=0.001&_msresflags=1089&_msresflags2=2&percolate=0&percolate_rt=0))

19. [orf207|conserved](http://10.139.25.109/mascot/cgi/protein_view.pl?file=../data/20120608/F008231.dat&hit=orf207|conserved&db_idx=1&px=1&ave_thresh=38&_ignoreionsscorebelow=20&report=0&_sigthreshold=0.001&_msresflags=1089&_msresflags2=2&percolate=0&percolate_rt=0) hypothetical membrane protein|[vB_BceM_Bc431v3] **Mass:** 20064  **Score:** 94  **emPAI:** 0.59

Sequence Coverage: **45%**; Matched peptides shown in **Bold Red**

**1** MTIRKPLDLI R**FVSSVPVLP DGTIPLNEMG TTVQ**YTSTLY TPSFSVSALA R**LTLEDIQR**N KIELI**NVPLD PRTIVSQVMN SDLATYNPR**V YVLVCAVVLE

**101** SFALLY**NLEE RNTNIQY**VTK KDILKMK**QNV NYIADYFGTE R**KYRTMIETL RDIDISIGYL ENQVESAMNK WVVR

**Start - End Observed Mr(expt) Mr(calc) ppm Miss Sequence**

**12 - 33 758.3901 2272.1486 2272.1661 -8 0 R.FVSSVPVLPDGTIPLNEMGTTV.Q**  ([Ions score 23](http://10.139.25.109/mascot/cgi/peptide_view.pl?file=../data/20120608/F008231.dat&query=10879&hit=1&index=orf207|conserved&px=1&section=5&ave_thresh=38&_ignoreionsscorebelow=20&report=0&_sigthreshold=0.001&_msresflags=1089&_msresflags2=2&percolate=0&percolate_rt=0))

**23 - 34 652.8231 1303.6317 1303.6330 -1 0 G.TIPLNEMGTTVQ.Y**  Deamidated (NQ) ([Ions score 22](http://10.139.25.109/mascot/cgi/peptide_view.pl?file=../data/20120608/F008231.dat&query=4764&hit=1&index=orf207|conserved&px=1&section=5&ave_thresh=38&_ignoreionsscorebelow=20&report=0&_sigthreshold=0.001&_msresflags=1089&_msresflags2=2&percolate=0&percolate_rt=0))

**52 - 59 494.2781 986.5415 986.5396 2 0 R.LTLEDIQR.N**  ([Ions score 46](http://10.139.25.109/mascot/cgi/peptide_view.pl?file=../data/20120608/F008231.dat&query=1051&hit=1&index=orf207|conserved&px=1&section=5&ave_thresh=38&_ignoreionsscorebelow=20&report=0&_sigthreshold=0.001&_msresflags=1089&_msresflags2=2&percolate=0&percolate_rt=0))

**66 - 87 821.7460 2462.2163 2462.2111 2 0 I.NVPLDPRTIVSQVMNSDLATYN.P**  Oxidation (M) ([Ions score 25](http://10.139.25.109/mascot/cgi/peptide_view.pl?file=../data/20120608/F008231.dat&query=11369&hit=1&index=orf207|conserved&px=1&section=5&ave_thresh=38&_ignoreionsscorebelow=20&report=0&_sigthreshold=0.001&_msresflags=1089&_msresflags2=2&percolate=0&percolate_rt=0))

**73 - 89 955.4792 1908.9438 1908.9251 10 0 R.TIVSQVMNSDLATYNPR.V**  Deamidated (NQ) ([Ions score 46](http://10.139.25.109/mascot/cgi/peptide_view.pl?file=../data/20120608/F008231.dat&query=9116&hit=1&index=orf207|conserved&px=1&section=5&ave_thresh=38&_ignoreionsscorebelow=20&report=0&_sigthreshold=0.001&_msresflags=1089&_msresflags2=2&percolate=0&percolate_rt=0))

**107 - 117 697.3405 1392.6665 1392.6633 2 0 Y.NLEERNTNIQY.V**  ([Ions score 39](http://10.139.25.109/mascot/cgi/peptide_view.pl?file=../data/20120608/F008231.dat&query=5536&hit=1&index=orf207|conserved&px=1&section=5&ave_thresh=38&_ignoreionsscorebelow=20&report=0&_sigthreshold=0.001&_msresflags=1089&_msresflags2=2&percolate=0&percolate_rt=0))

**128 - 141 845.3940 1688.7734 1688.7794 -4 0 K.QNVNYIADYFGTER.K**  ([Ions score 59](http://10.139.25.109/mascot/cgi/peptide_view.pl?file=../data/20120608/F008231.dat&query=7791&hit=1&index=orf207|conserved&px=1&section=5&ave_thresh=38&_ignoreionsscorebelow=20&report=0&_sigthreshold=0.001&_msresflags=1089&_msresflags2=2&percolate=0&percolate_rt=0))

**128 - 141 845.3998 1688.7850 1688.7794 3 0 K.QNVNYIADYFGTER.K**  ([Ions score 60](http://10.139.25.109/mascot/cgi/peptide_view.pl?file=../data/20120608/F008231.dat&query=7792&hit=1&index=orf207|conserved&px=1&section=5&ave_thresh=38&_ignoreionsscorebelow=20&report=0&_sigthreshold=0.001&_msresflags=1089&_msresflags2=2&percolate=0&percolate_rt=0))

20. [orf058|hypothetical](http://10.139.25.109/mascot/cgi/protein_view.pl?file=../data/20120608/F008231.dat&hit=orf058|hypothetical&db_idx=1&px=1&ave_thresh=38&_ignoreionsscorebelow=20&report=0&_sigthreshold=0.001&_msresflags=1089&_msresflags2=2&percolate=0&percolate_rt=0)protein|[vB_BceM_Bc431v3]    **Mass:** 15272  **Score:** 84   **emPAI:** 0.82

Sequence Coverage: **41%**; Matched peptides shown in **Bold Red**

**1** MTEVEKKEVE KKEEVLEVEL EPIVGHLVVK **GGLHSEFLNA TTSAIKKYTK QGYEVETQYQ MVLDPNSGEI IHSAY**LVGKL KETKPEEPEL VTVHIPHVSQ

**101** EALSKVEEWV ESYWGTGHMP NGIY**NTIMDL IDSTK**

**Start - End Observed Mr(expt) Mr(calc) ppm Miss Sequence**

**31 - 46 549.2886 1644.8441 1644.8471 -2 0 K.GGLHSEFLNATTSAIK.K**  ([Ions score 20](http://10.139.25.109/mascot/cgi/peptide_view.pl?file=../data/20120608/F008231.dat&query=7418&hit=1&index=orf058|hypothetical&px=1&section=5&ave_thresh=38&_ignoreionsscorebelow=20&report=0&_sigthreshold=0.001&_msresflags=1089&_msresflags2=2&percolate=0&percolate_rt=0))

**37 - 54 689.0231 2064.0474 2064.0415 3 0 E.FLNATTSAIKKYTKQGYE.V**  2 Deamidated (NQ) ([Ions score 20](http://10.139.25.109/mascot/cgi/peptide_view.pl?file=../data/20120608/F008231.dat&query=9950&hit=1&index=orf058|hypothetical&px=1&section=5&ave_thresh=38&_ignoreionsscorebelow=20&report=0&_sigthreshold=0.001&_msresflags=1089&_msresflags2=2&percolate=0&percolate_rt=0))

**37 - 54 689.0258 2064.0555 2064.0415 7 0 E.FLNATTSAIKKYTKQGYE.V**  2 Deamidated (NQ) ([Ions score 24](http://10.139.25.109/mascot/cgi/peptide_view.pl?file=../data/20120608/F008231.dat&query=9951&hit=1&index=orf058|hypothetical&px=1&section=5&ave_thresh=38&_ignoreionsscorebelow=20&report=0&_sigthreshold=0.001&_msresflags=1089&_msresflags2=2&percolate=0&percolate_rt=0))

**49 - 59 673.3170 1344.6194 1344.6198 0 0 Y.TKQGYEVETQY.Q**  ([Ions score 49](http://10.139.25.109/mascot/cgi/peptide_view.pl?file=../data/20120608/F008231.dat&query=5158&hit=1&index=orf058|hypothetical&px=1&section=5&ave_thresh=38&_ignoreionsscorebelow=20&report=0&_sigthreshold=0.001&_msresflags=1089&_msresflags2=2&percolate=0&percolate_rt=0))

**49 - 59 673.3179 1344.6212 1344.6198 1 0 Y.TKQGYEVETQY.Q**  ([Ions score 49](http://10.139.25.109/mascot/cgi/peptide_view.pl?file=../data/20120608/F008231.dat&query=5159&hit=1&index=orf058|hypothetical&px=1&section=5&ave_thresh=38&_ignoreionsscorebelow=20&report=0&_sigthreshold=0.001&_msresflags=1089&_msresflags2=2&percolate=0&percolate_rt=0))

**60 - 75 887.9283 1773.8420 1773.8243 10 0 Y.QMVLDPNSGEIIHSAY.L**  Deamidated (NQ) ([Ions score 45](http://10.139.25.109/mascot/cgi/peptide_view.pl?file=../data/20120608/F008231.dat&query=8323&hit=1&index=orf058|hypothetical&px=1&section=5&ave_thresh=38&_ignoreionsscorebelow=20&report=0&_sigthreshold=0.001&_msresflags=1089&_msresflags2=2&percolate=0&percolate_rt=0))

**125 - 135 625.8186 1249.6226 1249.6224 0 0 Y.NTIMDLIDSTK.-**  ([Ions score 26](http://10.139.25.109/mascot/cgi/peptide_view.pl?file=../data/20120608/F008231.dat&query=4132&hit=1&index=orf058|hypothetical&px=1&section=5&ave_thresh=38&_ignoreionsscorebelow=20&report=0&_sigthreshold=0.001&_msresflags=1089&_msresflags2=2&percolate=0&percolate_rt=0))

**125 - 135 625.8187 1249.6228 1249.6224 0 0 Y.NTIMDLIDSTK.-**  ([Ions score 55](http://10.139.25.109/mascot/cgi/peptide_view.pl?file=../data/20120608/F008231.dat&query=4133&hit=1&index=orf058|hypothetical&px=1&section=5&ave_thresh=38&_ignoreionsscorebelow=20&report=0&_sigthreshold=0.001&_msresflags=1089&_msresflags2=2&percolate=0&percolate_rt=0))

21. [orf206|conserved](http://10.139.25.109/mascot/cgi/protein_view.pl?file=../data/20120608/F008231.dat&hit=orf206|conserved&db_idx=1&px=1&ave_thresh=38&_ignoreionsscorebelow=20&report=0&_sigthreshold=0.001&_msresflags=1089&_msresflags2=2&percolate=0&percolate_rt=0) hypothetical protein, putative baseplate protein|[vB_BceM_Bc431v3] **Mass:**28089 **Score:**83 **emPAI:** 0.25

Sequence Coverage: **17%**; Matched peptides shown in **Bold Red**

**1** MAKFKRRIIA EGDTM**QAIAQ QELGDVSRWI ELVRF**NDLRH PY**IVDTVAEK LTNPN**HLLTI GDTLLIEMSE NSQNELMNAL KRTTDFDKEE LYALALGKDL

**101** DVLPIPKPFG KAGWDSDILE MKDDGRGDIA TIRGVENLKQ SLFIRLITPL GSYIGYPRYG SKVHEYL**GRK NTEENAAL**LD IEIERTLRTD GRVRTVEKVG

**201** HVIDGNSYST TFKVFSIAME EAFLLALSGE LGKEGSLVLQ DNFVDNMIR

**Start - End Observed Mr(expt) Mr(calc) ppm Miss Sequence**

**16 - 29 800.9089 1599.8033 1599.8005 2 0 M.QAIAQQELGDVSRW.I**  ([Ions score 72](http://10.139.25.109/mascot/cgi/peptide_view.pl?file=../data/20120608/F008231.dat&query=7086&hit=1&index=orf206|conserved&px=1&section=5&ave_thresh=38&_ignoreionsscorebelow=20&report=0&_sigthreshold=0.001&_msresflags=1089&_msresflags2=2&percolate=0&percolate_rt=0))

**30 - 35 388.7368 775.4590 775.4592 0 0 W.IELVRF.N**  ([Ions score 27](http://10.139.25.109/mascot/cgi/peptide_view.pl?file=../data/20120608/F008231.dat&query=199&hit=1&index=orf206|conserved&px=1&section=5&ave_thresh=38&_ignoreionsscorebelow=20&report=0&_sigthreshold=0.001&_msresflags=1089&_msresflags2=2&percolate=0&percolate_rt=0))

**43 - 51 494.2891 986.5636 986.5648 -1 0 Y.IVDTVAEKL.T**  ([Ions score 47](http://10.139.25.109/mascot/cgi/peptide_view.pl?file=../data/20120608/F008231.dat&query=1053&hit=1&index=orf206|conserved&px=1&section=5&ave_thresh=38&_ignoreionsscorebelow=20&report=0&_sigthreshold=0.001&_msresflags=1089&_msresflags2=2&percolate=0&percolate_rt=0))

**46 - 55 543.7976 1085.5807 1085.5717 8 0 D.TVAEKLTNPN.H**  ([Ions score 20](http://10.139.25.109/mascot/cgi/peptide_view.pl?file=../data/20120608/F008231.dat&query=2104&hit=1&index=orf206|conserved&px=1&section=5&ave_thresh=38&_ignoreionsscorebelow=20&report=0&_sigthreshold=0.001&_msresflags=1089&_msresflags2=2&percolate=0&percolate_rt=0))

**168 - 178 602.7987 1203.5829 1203.5731 8 0 L.GRKNTEENAAL.L**  2 Deamidated (NQ) ([Ions score 20](http://10.139.25.109/mascot/cgi/peptide_view.pl?file=../data/20120608/F008231.dat&query=3596&hit=3&index=orf206|conserved&px=1&section=5&ave_thresh=38&_ignoreionsscorebelow=20&report=0&_sigthreshold=0.001&_msresflags=1089&_msresflags2=2&percolate=0&percolate_rt=0))

22. [orf214|conserved](http://10.139.25.109/mascot/cgi/protein_view.pl?file=../data/20120608/F008231.dat&hit=orf214|conserved&db_idx=1&px=1&ave_thresh=38&_ignoreionsscorebelow=20&report=0&_sigthreshold=0.001&_msresflags=1089&_msresflags2=2&percolate=0&percolate_rt=0) hypothetical protein|[vB_BceM_Bc431v3]    **Mass:** 49079  **Score:** 81  **emPAI:** 0.30

Sequence Coverage: **41%**; Matched peptides shown in **Bold Red**

**1** MVVR**ATGQIT LSDLNDAK**QL VLYLNSNYKT QIYDPNGTTY NPNFTSSNLV ITPELYVA**GG NGGNMLPSAA IK**SLFWYEGS QTVTPLAETG AGTTPSGLSY

**101** TIPTGAVATT AKPL**TIKSNL TATTSQMF**TC VITYLDPDLQ METTIKANVD **IVKIVNGAAG T**NGTDAYYLN LWAPGGDAIR NSNGNLTLKA DMY**KGAGSVT**

**201 PTAF**QWYIQD STATVGGGGD ADGGAGWRR**I NNVADPTAAP TLALSPNASS QLTPATYYVK** YTWCGLSGET IGSAQAQ**LAV TTGNELKVTI PAFATNVTMA**

**301 KVYIGTASGV** LF**YAGDITTS AGNLIVKRF**D NTAEPIPTAS STSMNVAQIT IRNWAIPGVK GFKCVTSVSG TSTKFTAVIV VRDFQDPLVV **NIIGTNVFKN**

**401 GQGSITLNA**Q LIQAGLVISN TGYTFGWSLY KPDGNLIK**TY PTVTTDQITV PSTDVDATAN LVVDASK**

**Start - End Observed Mr(expt) Mr(calc) ppm Miss Sequence**

**5 - 18 723.8766 1445.7386 1445.7362 2 0 R.ATGQITLSDLNDAK.Q**  ([Ions score 68](http://10.139.25.109/mascot/cgi/peptide_view.pl?file=../data/20120608/F008231.dat&query=5899&hit=1&index=orf214|conserved&px=1&section=5&ave_thresh=38&_ignoreionsscorebelow=20&report=0&_sigthreshold=0.001&_msresflags=1089&_msresflags2=2&percolate=0&percolate_rt=0))

**59 - 72 651.8329 1301.6513 1301.6398 9 0 A.GGNGGNMLPSAAIK.S**  Oxidation (M) ([Ions score 26](http://10.139.25.109/mascot/cgi/peptide_view.pl?file=../data/20120608/F008231.dat&query=4748&hit=1&index=orf214|conserved&px=1&section=5&ave_thresh=38&_ignoreionsscorebelow=20&report=0&_sigthreshold=0.001&_msresflags=1089&_msresflags2=2&percolate=0&percolate_rt=0))

**115 - 128 771.8964 1541.7783 1541.7759 2 0 L.TIKSNLTATTSQMF.T**  ([Ions score 30](http://10.139.25.109/mascot/cgi/peptide_view.pl?file=../data/20120608/F008231.dat&query=6612&hit=1&index=orf214|conserved&px=1&section=5&ave_thresh=38&_ignoreionsscorebelow=20&report=0&_sigthreshold=0.001&_msresflags=1089&_msresflags2=2&percolate=0&percolate_rt=0))

**151 - 161 521.8118 1041.6090 1041.6182 -9 0 D.IVKIVNGAAGT.N**  ([Ions score 22](http://10.139.25.109/mascot/cgi/peptide_view.pl?file=../data/20120608/F008231.dat&query=1603&hit=1&index=orf214|conserved&px=1&section=5&ave_thresh=38&_ignoreionsscorebelow=20&report=0&_sigthreshold=0.001&_msresflags=1089&_msresflags2=2&percolate=0&percolate_rt=0))

**152 - 158 351.2132 700.4118 700.4119 0 0 I.VKIVNGA.A**  Deamidated (NQ) ([Ions score 21](http://10.139.25.109/mascot/cgi/peptide_view.pl?file=../data/20120608/F008231.dat&query=3&hit=1&index=orf214|conserved&px=1&section=5&ave_thresh=38&_ignoreionsscorebelow=20&report=0&_sigthreshold=0.001&_msresflags=1089&_msresflags2=2&percolate=0&percolate_rt=0))

**194 - 204 518.2766 1034.5385 1034.5397 -1 0 Y.KGAGSVTPTAF.Q**  ([Ions score 22](http://10.139.25.109/mascot/cgi/peptide_view.pl?file=../data/20120608/F008231.dat&query=1516&hit=1&index=orf214|conserved&px=1&section=5&ave_thresh=38&_ignoreionsscorebelow=20&report=0&_sigthreshold=0.001&_msresflags=1089&_msresflags2=2&percolate=0&percolate_rt=0))

**230 - 260 1063.5552 3187.6421 3187.6401 1 0 R.INNVADPTAAPTLALSPNASSQLTPATYYVK.Y**  [Ions score 39](http://10.139.25.109/mascot/cgi/peptide_view.pl?file=../data/20120608/F008231.dat&query=12057&hit=1&index=orf214|conserved&px=1&section=5&ave_thresh=38&_ignoreionsscorebelow=20&report=0&_sigthreshold=0.001&_msresflags=1089&_msresflags2=2&percolate=0&percolate_rt=0))

**278 - 292 509.9641 1526.8704 1526.8556 10 0 Q.LAVTTGNELKVTIPA.F**  Deamidated (NQ) ([Ions score 24](http://10.139.25.109/mascot/cgi/peptide_view.pl?file=../data/20120608/F008231.dat&query=6489&hit=1&index=orf214|conserved&px=1&section=5&ave_thresh=38&_ignoreionsscorebelow=20&report=0&_sigthreshold=0.001&_msresflags=1089&_msresflags2=2&percolate=0&percolate_rt=0))

**288 - 301 732.3976 1462.7806 1462.7854 -3 0 K.VTIPAFATNVTMAK.V**  ([Ions score 39](http://10.139.25.109/mascot/cgi/peptide_view.pl?file=../data/20120608/F008231.dat&query=6003&hit=1&index=orf214|conserved&px=1&section=5&ave_thresh=38&_ignoreionsscorebelow=20&report=0&_sigthreshold=0.001&_msresflags=1089&_msresflags2=2&percolate=0&percolate_rt=0))

**288 - 301 732.4002 1462.7859 1462.7854 0 0 K.VTIPAFATNVTMAK.V**  ([Ions score 45](http://10.139.25.109/mascot/cgi/peptide_view.pl?file=../data/20120608/F008231.dat&query=6004&hit=1&index=orf214|conserved&px=1&section=5&ave_thresh=38&_ignoreionsscorebelow=20&report=0&_sigthreshold=0.001&_msresflags=1089&_msresflags2=2&percolate=0&percolate_rt=0))

**296 - 310 509.9241 1526.7505 1526.7650 -10 0 T.NVTMAKVYIGTASGV.L**  Oxidation (M) ([Ions score 26](http://10.139.25.109/mascot/cgi/peptide_view.pl?file=../data/20120608/F008231.dat&query=6485&hit=1&index=orf214|conserved&px=1&section=5&ave_thresh=38&_ignoreionsscorebelow=20&report=0&_sigthreshold=0.001&_msresflags=1089&_msresflags2=2&percolate=0&percolate_rt=0))

**313 - 329 609.3333 1824.9781 1824.9734 3 0 F.YAGDITTSAGNLIVKRF.D**  ([Ions score 32](http://10.139.25.109/mascot/cgi/peptide_view.pl?file=../data/20120608/F008231.dat&query=8685&hit=1&index=orf214|conserved&px=1&section=5&ave_thresh=38&_ignoreionsscorebelow=20&report=0&_sigthreshold=0.001&_msresflags=1089&_msresflags2=2&percolate=0&percolate_rt=0))

**313 - 329 609.6646 1825.9720 1825.9574 8 0 F.YAGDITTSAGNLIVKRF.D**  Deamidated (NQ) ([Ions score 40](http://10.139.25.109/mascot/cgi/peptide_view.pl?file=../data/20120608/F008231.dat&query=8690&hit=1&index=orf214|conserved&px=1&section=5&ave_thresh=38&_ignoreionsscorebelow=20&report=0&_sigthreshold=0.001&_msresflags=1089&_msresflags2=2&percolate=0&percolate_rt=0))

**391 - 402 653.3391 1304.6637 1304.6725 -7 0 V.NIIGTNVFKNGQ.G**  Deamidated (NQ) ([Ions score 20](http://10.139.25.109/mascot/cgi/peptide_view.pl?file=../data/20120608/F008231.dat&query=4782&hit=1&index=orf214|conserved&px=1&section=5&ave_thresh=38&_ignoreionsscorebelow=20&report=0&_sigthreshold=0.001&_msresflags=1089&_msresflags2=2&percolate=0&percolate_rt=0))

**391 - 402 653.3399 1304.6652 1304.6725 -6 0 V.NIIGTNVFKNGQ.G**  Deamidated (NQ) ([Ions score 20](http://10.139.25.109/mascot/cgi/peptide_view.pl?file=../data/20120608/F008231.dat&query=4785&hit=2&index=orf214|conserved&px=1&section=5&ave_thresh=38&_ignoreionsscorebelow=20&report=0&_sigthreshold=0.001&_msresflags=1089&_msresflags2=2&percolate=0&percolate_rt=0))

**400 - 409 488.2420 974.4694 974.4669 3 0 K.NGQGSITLNA.Q**  Deamidated (NQ) ([Ions score 21](http://10.139.25.109/mascot/cgi/peptide_view.pl?file=../data/20120608/F008231.dat&query=1002&hit=1&index=orf214|conserved&px=1&section=5&ave_thresh=38&_ignoreionsscorebelow=20&report=0&_sigthreshold=0.001&_msresflags=1089&_msresflags2=2&percolate=0&percolate_rt=0))

**439 - 467 1008.1783 3021.5144 3021.5030 4 0 K.TYPTVTTDQITVPSTDVDATANLVVDASK.-**  ([Ions score 30](http://10.139.25.109/mascot/cgi/peptide_view.pl?file=../data/20120608/F008231.dat&query=11959&hit=1&index=orf214|conserved&px=1&section=5&ave_thresh=38&_ignoreionsscorebelow=20&report=0&_sigthreshold=0.001&_msresflags=1089&_msresflags2=2&percolate=0&percolate_rt=0))

23. [orf235|phage](http://10.139.25.109/mascot/cgi/protein_view.pl?file=../data/20120608/F008231.dat&hit=orf235|phage&db_idx=1&px=1&ave_thresh=38&_ignoreionsscorebelow=20&report=0&_sigthreshold=0.001&_msresflags=1089&_msresflags2=2&percolate=0&percolate_rt=0) prohead protease|[vB_BceM_Bc431v3]   **Mass:** 29924    **Score:** 52  **emPAI:** 0.11

Sequence Coverage: **12%**; Matched peptides shown in **Bold Red**

**1** MQAVNPITGK VNLFVPIDIE ESISKSNEDT SSKSWCLRGY ATTPDLDLQD DIIDPRGIDI SHFITHGYLN YEHFQGEEYK VGVPTEGTHV DDVGLFVEGK

**101** LYKDNPYAK**S IWNLANSIQK** SGIDRKIGFS IEGFAKARDK ADPRIIKSTY ITNVAVTTSP ANPNAVWDAF MKSW**QVGYAI TPEESVGVAA ISPDSLA**RSL

**201** YNLSWSLKEE DESKFKDVWG EVGNYLDAME RYTPESAILF LQISKGYSRV EAKEKLEQLS QQAKQNI

**Start - End Observed Mr(expt) Mr(calc) ppm Miss Sequence**

**110 - 120 637.3442 1272.6739 1272.6826 -7 0 K.SIWNLANSIQK.S**  ([Ions score 39](http://10.139.25.109/mascot/cgi/peptide_view.pl?file=../data/20120608/F008231.dat&query=4390&hit=1&index=orf235|phage&px=1&section=5&ave_thresh=38&_ignoreionsscorebelow=20&report=0&_sigthreshold=0.001&_msresflags=1089&_msresflags2=2&percolate=0&percolate_rt=0))

**110 - 120 637.3458 1272.6771 1272.6826 -4 0 K.SIWNLANSIQK.S**  ([Ions score 50](http://10.139.25.109/mascot/cgi/peptide_view.pl?file=../data/20120608/F008231.dat&query=4392&hit=1&index=orf235|phage&px=1&section=5&ave_thresh=38&_ignoreionsscorebelow=20&report=0&_sigthreshold=0.001&_msresflags=1089&_msresflags2=2&percolate=0&percolate_rt=0))

**175 - 197 759.0508 2274.1305 2274.1267 2 0 W.QVGYAITPEESVGVAAISPDSLA.R**  ([Ions score 23](http://10.139.25.109/mascot/cgi/peptide_view.pl?file=../data/20120608/F008231.dat&query=10887&hit=1&index=orf235|phage&px=1&section=5&ave_thresh=38&_ignoreionsscorebelow=20&report=0&_sigthreshold=0.001&_msresflags=1089&_msresflags2=2&percolate=0&percolate_rt=0))

___________________________________________________________________________________________________________________________________________
