# Supplementary material for: Genome sequence and analysis of a broad-host range lytic bacteriophage that infects the Bacillus cereus group
Source: Virol J. 2013 Feb 7;10:48. doi: 10.1186/1743-422X-10-48 (PMC3601020; doi:10.1186/1743-422X-10-48)
Supplement: Additional file 3: Table S3 — Potential promoters and rho-independent terminators in vB_BceM_Bc431v3 with WebLogo of the consensus. [file 1743-422X-10-48-S3.doc]

**Additional file 3, Table S3:** Potential promoters and rho-independent terminators in phage Bc431v3 with the conserved -10 and -35 residues in capital letters

1. SigmaA Promoters

| **Name** | **Location** | **Sequence** |
| --- | --- | --- |
| P_orf017_ | 8743..8772 | ctttttcttttaatttattgTTGACAtatagaatatgtacaagcTATAcTat |
| P_orf019_ | 10469..10498 | aaaaagcagtcaatttcttgTTGACtgcttttttgtgtctaTGaTATAgTag |
| P_orf024_ | 14054..14156 | tgtaacttttaaaaaagttgTTGACtataatacaacaatggataTATAcTaa |
| P_orf046_ | 22846..22948 | aaaattctttttaaaatgttgTTGACtttaagacaacaagaTGagATAATga |
| P_orf049_ | 24191..24163 | aaaattctttttaaaatgttgTTGACtttaagacaacaatgaatTATAcTga |
| P_orf050_ | 24743..24845 | aaaattctttttaaaatgttgTTGACtttaagacaacaagaTGagATAATga |
| P_orf051_ | 25497-25469 | taaattctttttaaaatgttgTTGACtttaagacaacaaacaatTATAcTga |
| P_orf052_ | 26062..26164 | aaattatttttggttatattgTTGACtttagggcaatatatctaTAgtATag |
| P_orf056_ | 27848..27820 | catttattttctgtatagttgTTGACtttaaggaaacaatcctgTATtATat |
| P_orf075_ | 34011..34113 | ttttatttttttaatttattgTTGACgattggacaacctctaTGtTATtcTa |
| P_orf091_ | 43340..43369 | ttttaaaaagttattgactgTAgACAacatgatagtatgatgaaTATAAgaa |
| P_orf114_ | 56891..56993 | caaaaattttttaaatagttgTTGACtataagaaaacaacaTGaTAagATaa |
| P_orf119_ | 59131..59107 | ttttttttttgtttattttgTTGACAgtgggacaacctataTGaTATtcTtt |
| P_orf143_ | 71405..71507 | agaatatgaataaaaaagttgTTGACttaaagacaacatcaTGgTAaAtTag |
| P_orf146_ | 72611..72713 | aaattaattttaataaagttgTTGACttatagaaaacaacaTGgTAaAtTaa |
| P_orf156_ | 75134..75236 | attttttttttataaatgttgTTGACtttaggaaaacatgtaTGgTAaAtTa |
| P_orf161A_ | 78240..78342 | ctaaattatgctagttttttgTTGACtgttacgaaacatgaTGaTAgAATga |
| P_orf169_ | 84368..84342 | tataatttctgtaactttttgTTGACAcacagaataggatagTgTAatATac |
| P_orf171_ | 87673..87775 | ttttttgtgtctattatcttgTTGACttatagtgtatgatatagTActATta |
| P_orf173_ | 89472..89574 | ttttttctttgtaatttattgTTGACttatagacaaagttacagTAatATta |
| P_orf181_ | 92735..92837 | atataagttgtaaaaaagttgTTGACtaaaagacaacctacaTGcTATAgTa |
| P_orf182_ | 93518..93492 | cttctttttttttgtataacttgTTGACttttaggaaacaaTGaTATAAgat |
| P_orf190_ | 99747..99719 | ttagcagagtggcaataatgaTTGACAgtaactaatctgtaTGcTAgAATac |
| P_orf194_ | 103822..103794 | acggactaaccctttttgtcgTTGACAtaacacgctctaaaTGaTAcAATga |
| P_orf198_ | 108580..108553 | agtatggaatagaatttagataTTGAaAtcgttatagtatgTGtTATAcTgt |
| P_orf201_ | 110862..110891 - | tattatttaaatatatttaaTTaACActaatatataatataaataATAATta |
| P_orf221_ | 139069..139041 | ctatttttttttattttattgTTGACtattggaaaaccttataaTATAcTaa |

B. WebLogo of the putative promoters, with the exception of P_orf091_, P_orf198_ and P_orf201_.


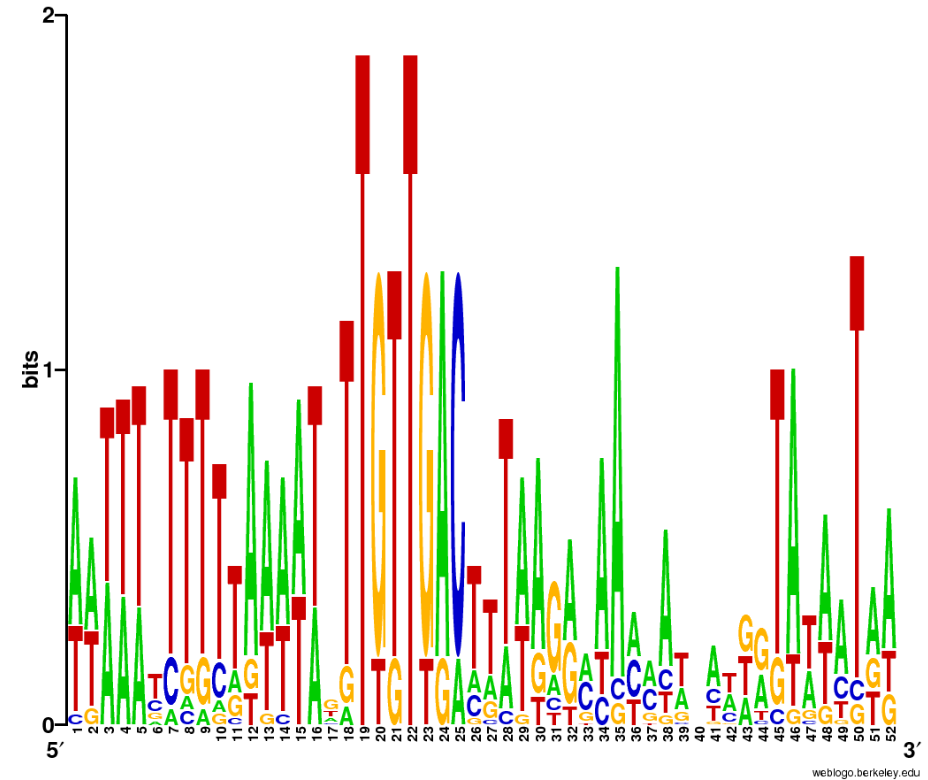


C.Terminators

| **Name** | **Location** | **Sequence** | **ΔG (kcal/mol)*** |
| --- | --- | --- | --- |
| T_orf001_ | 918..948 | ggagtaggggaaacccactcctttttcttat | -15.90 |
| T_orf002_ | 946..925 | gggaaacccactcctttttctt | - 3.90 |
| T_orf012_ | 6528..6558 | ccgactctaaatagggtcggttttctttttt | -11.50 |
| T_orf013_ | 7142..7174 | gagtagcaattgctactctttttttttgtttat | -10.90 |
| T_orf016_ | 8702..8744 | agagactacatttagtagtctctttttcttttaatttattgtt | -12.30 |
| T_orf018_ | 10454..10482 | gcagtcaatttcttgttgactgctttttt | - 13.0 |
| T_orf023_ | 14038..14071 | AACTAGGTTATGACACCTAGTTATTTTTTTTTT | - 9.0 |
| T_orf042_ | 21118..21146 | ggactctttaattagagtcctttttgttt | - 11.10 |
| T_orf043_ | 21663..21628 | gatttcctatgatattaggaagtctttttttttatt | - 11.30 |
| T_orf053_ | 26454..26418 | CTCACTGTACAATATATGCAGTGAGTTTTTCTATTAT | - 11.30 |
| T_orf058_ | 28292..28256 | GGATTGGTAGCTTTCCCCATAGGCCGCCGATCCTTTT | - 16.90 |
| T_orf097_ | 44887..44862 | gggagtccattacgggttcccttttt | - 11.50 |
| T_orf106_ | 51992..51962 | cttccgatattttttcggaagtttttttttt | - 9.30 |
| T_orf110_ | 54452..54428 | gagcgtttatgcgctcttttttttt | - 9.20 |
| T_orf122_ | 60727..60690 | ggagaactagggactacccctagtttttctttatattt | - 17.00 |
| T_orf126_ | 63131..63097 | gggttgcattttatggtgcaaccctttattatatt | - 15.60 |
| T_orf129_ | 65475..65448 | gagaatgctataacaggcattctctttt | - 12.80 |
| T_orf157_ | 75263..75217 | ggaaggctctgtaatagagtctttctttttttttattttttttttat | - 12.40 |
| T_orf170_ | 84334..84302 | gagaagagacaagcagtttcttctctttagttt | - 13.50 |
| T_orf172_ | 87778..87753 | gagaggtgtaacaacctctctttttt | - 11.70 |
| T_orf176_ | 90786..90758 | gaacaggtttccgtaacctgttctttttt | -12.50 |
| T_orf183_ | 93557..93529 | gaagaggaaatttcctcttcttttttttt | - 11.50 |
| T_orf195_ | 103854..103827 | gggttaggtctacggactaacccttttt | - 13.80 |
| T_orf202_ | 111167..111139 | gaggtgcgtatcacacctctttttaattt | - 8.50 |
| T_orf209_ | 119146..119115 | gccgaggtttccctcggcttttcttatattat | - 13.50 |
| T_orf214_ | 127207..127178 | ggaacttagcgataagttccttttattttt | - 9.80 |
| T_orf219_ | 136934..136904 | gggggagagggaaacctcttctcctattttt | - 20.60 |
| T_orf221_ | 138076..138051 | gctaccacactaagtggtagcttttt | - 13.70 |
| T_orf222_ | 139114..139068 | gacacggataataaaaattcgtgtctatttttttttattttattgtt | - 11.00 |
| T_orf223_ | 139914..139878 | gtaccctctatccactctgagggtacttttctatttt | - 13.50 |
| T_orf224_ | 142322..142300 | gacccgagagggtctttttattt | - 11.00 |
| T_orf232_ | 148247..148212 | gggacagacgaaaattctgtccctttttattttatt | - 13.30 |
| T_orf233_ | 149835..149798 | catagacgggttcctcctcctagcccgtctgtgtttat | - 17.50 |
| T_orf239_ | 154779..154748 | GACTCCTCGTCTCCTTTCGAGGGGTCTTTTTT | - 15.50 |

Capitalized terminators discovered using ARNold; those in small letters by TransTerm. * Calculated using MFOLD (<http://mfold.rna.albany.edu/?q=mfold/RNA-Folding-Form>)
