# Supplementary material for: Genome sequence and analysis of a broad-host range lytic bacteriophage that infects the Bacillus cereus group
Source: Virol J. 2013 Feb 7;10:48. doi: 10.1186/1743-422X-10-48 (PMC3601020; doi:10.1186/1743-422X-10-48)
Supplement: Additional file 4: Table S4 — tRNAs discovered in the sequence of Bc341v3 using tRNAScan-SE. [file 1743-422X-10-48-S4.doc]

**Additional file 4, Table S4**. tRNAs discovered in the sequence of vB_BceM_Bc341v3 using tRNAScan-SE.

| **tRNA** | **Location** | **Amino acid** | **Anticodon** | **Cove Score** |
| --- | --- | --- | --- | --- |
| 1 | 155941..155870 | Met | CAT | 63.0 |
| 2 | 156027..155946 | Trp | CCA | 29.1 |
| 3 | 156177..156093 | Leu | TAG | 49.2 |
| 4 | 156262..156186 | Ile | GAT | 76.1 |
| 5 | 156352..156266 | Leu | TAA | 64.8 |
| 6 | 156352..156266 | Tyr | GTA | 35.8 |
| 7 | 156519..156445 | Phe | GAA | 37.9 |
| 8 | 156692..156621 | Pro | TGG | 28.2 |
| 9 | 156774..156701 | His | GTG | 55.9 |
| 10 | 156926..156853 | Gln | TTG | 54.1 |
| 11 | 157094..157005 | Ser | TGA | 48.5 |
| 12 | 157226..157153 | Arg | TCT | 61.9 |
| 13 | 157420..157348 | Ile | TAT | 49.7 |
| 14 | 157494..157423 | Asp | GTC | 64.7 |
| 15 | 157575..157499 | Glu | TTC | 72.1 |
| 16 | 157659..157585 | Asp | GTC | 54.3 |
| 17 | 157741..157665 | Thr | TGT | 86.9 |
| 18 | 157821..157746 | Gly | TCC | 50.2 |
| 19 | 158085..158011 | Asn | GTT | 83.6 |
| 20 | 158203..158131 | Cys | GCA | 61.7 |
| 21 | 158301..158212 | Ser | GCT | 51.2 |
|  |  |  |  |  |
