# Supplementary material for: Genome sequence and analysis of a broad-host range lytic bacteriophage that infects the Bacillus cereus group
Source: Virol J. 2013 Feb 7;10:48. doi: 10.1186/1743-422X-10-48 (PMC3601020; doi:10.1186/1743-422X-10-48)
Supplement: Additional file 5: Table S5 — Comparison of codon usage in Bc431v3 genes with that of Bacillus cereus Q1. Those codons which are 50% more common in the phage are coloured orange. [file 1743-422X-10-48-S5.doc]

| **Additional file 5, Table S5.** Comparison of codon usage in vB_BceM_Bc431v3 genes with that of *Bacillus cereus* Q1. Those codons which are 50% more common in the phage are coloured orange. | | | | | |
| --- | --- | --- | --- | --- | --- |
| **Amino acid** | **Codon** | **Fraction (Host)** | **Fraction (Phage)** | **Codon usage (Phage/host)** | **Phage tRNA present** |
| Ala | GCG | 0.20 | 0.13 | 0.65 |  |
| Ala | GCA | 0.43 | 0.44 | 1.02 |  |
| Ala | GCT | 0.31 | 0.34 | 1.10 |  |
| Ala | GCC | 0.06 | 0.09 | 1.50 |  |
| Arg | CGG | 0.04 | 0.06 | 1.50 |  |
| Arg | CGA | 0.15 | 0.16 | 1.07 |  |
| Arg | CGT | 0.37 | 0.18 | 0.49 |  |
| Arg | CGC | 0.13 | 0.05 | 0.38 |  |
| Arg | AGG | 0.07 | 0.17 | 2.43 |  |
| Arg | AGA | 0.25 | 0.38 | 1.52 | Yes |
| Asn | AAT | 0.71 | 0.44 | 0.62 |  |
| Asn | AAC | 0.29 | 0.56 | 1.93 | Yes |
| Asp | GAT | 0.81 | 0.54 | 0.67 |  |
| Asp | GAC | 0.19 | 0.46 | 2.42 | Yes |
| Cys | TGT | 0.74 | 0.60 | 0.81 |  |
| Cys | TGC | 0.26 | 0.40 | 1.54 | Yes |
| Gln | CAG | 0.18 | 0.35 | 1.94 |  |
| Gln | CAA | 0.82 | 0.65 | 0.79 | Yes |
| Glu | GAG | 0.25 | 0.40 | 1.6 |  |
| Glu | GAA | 0.75 | 0.60 | 0.80 | Yes |
| Gly | GGG | 0.14 | 0.14 | 1.00 |  |
| Gly | GGA | 0.36 | 0.38 | 1.06 |  |
| Gly | GGT | 0.37 | 0.40 | 1.08 |  |
| Gly | GGC | 0.13 | 0.08 | 0.62 |  |
| His | CAT | 0.77 | 0.55 | 0.71 |  |
| His | CAC | 0.23 | 0.45 | 1.96 | Yes |
| Ile | ATA | 0.21 | 0.25 | 1.19 | Yes |
| Ile | ATT | 0.63 | 0.43 | 0.68 |  |
| Ile | ATC | 0.16 | 0.32 | 2.00 | Yes |
| Leu | CTG | 0.03 | 0.09 | 3.00 |  |
| Leu | CTA | 0.11 | 0.25 | 2.27 | Yes |
| Leu | CTT | 0.19 | 0.18 | 0.95 |  |
| Leu | CTC | 0.05 | 0.07 | 1.40 |  |
| Leu | TTG | 0.10 | 0.14 | 1.40 |  |
| Leu | TTA | 0.52 | 0.28 | 0.54 | Yes |
| Lys | AAG | 0.24 | 0.44 | 1.83 |  |
| Lys | AAA | 0.76 | 0.56 | 0.74 |  |
| Met | ATG | 1.00 | 1.00 | 1.00 | Yes |
| Phe | TTT | 0.70 | 0.41 | 0.59 |  |
| Phe | TTC | 0.30 | 0.59 | 1.97 | Yes |
| Pro | CCG | 0.22 | 0.20 | 0.91 |  |
| Pro | CCA | 0.48 | 0.31 | 0.65 | Yes |
| Pro | CCT | 0.27 | 0.41 | 1.52 |  |
| Pro | CCC | 0.03 | 0.09 | 3.00 |  |
| Ser | TCG | 0.08 | 0.10 | 1.25 |  |
| Ser | TCA | 0.25 | 0.18 | 0.72 | Yes |
| Ser | TCT | 0.27 | 0.18 | 0.67 |  |
| Ser | TCC | 0.06 | 0.08 | 1.33 |  |
| Ser | AGT | 0.25 | 0.28 | 1.12 |  |
| Ser | AGC | 0.10 | 0.17 | 1.70 | Yes |
| Thr | ACG | 0.24 | 0.22 | 0.92 |  |
| Thr | ACA | 0.49 | 0.39 | 0.80 | Yes |
| Thr | ACT | 0.22 | 0.27 | 1.23 |  |
| Thr | ACC | 0.05 | 0.11 | 2.20 |  |
| Trp | TGG | 1.00 | 1.00 | 1.00 |  |
| Tyr | TAT | 0.75 | 0.46 | 0.61 |  |
| Tyr | TAC | 0.25 | 0.54 | 2.16 | Yes |
| Val | GTG | 0.15 | 0.15 | 1.00 |  |
| Val | GTA | 0.42 | 0.41 | 0.98 |  |
| Val | GTT | 0.35 | 0.33 | 0.94 |  |
| Val | GTC | 0.08 | 0.10 | 1.25 |  |
| Stop | TGA | 0.15 | 0.31 | 2.07 |  |
| Stop | TAG | 0.16 | 0.25 | 1.56 |  |
| Stop | TAA | 0.69 | 0.44 | 0.64 |  |
